# Chemical Genetics with SP600125 Reveals That Mps1 Protein Kinase Works as a Regulatory Element in Post-embryonic Development of the *Arabidopsis thaliana* Root System—An Insight into Plant Cell Cycle Control

**DOI:** 10.1021/acsomega.5c07325

**Published:** 2025-10-27

**Authors:** Emanuel Victor Nogueira Gotardo, Eduardo Alves Gamosa de Oliveira, Lucas Zanchetta Passamani, Izabela Silva dos Santos, Geraldo de Amaral Gravina, Claudete Santa-Catarina, Vanildo Silveira, Antônia Elenir Amâncio Oliveira, Marco Antonio Lopes Cruz

**Affiliations:** † Laboratório de Biotecnologia Vegetal, 28125 Universidade Federal do Rio de Janeiro, Av. Amaro Reinaldo dos Santos Silva, 764, São José do Barreto, Macaé, Rio de Janeiro 27965-045, Brazil; ‡ Laboratório de Química de Função de Proteínas e Peptídeos, Centro de Biociências e Biotecnologia, 28109 Universidade Estadual do Norte Fluminense Darcy Ribeiro, Av. Alberto Lamego 2000, P5, sala 224, Campos dos Goytacazes 28013-602, Brazil; § Laboratório de Biotecnologia, Centro de Biociências e Biotecnologia, Universidade Estadual do Norte Fluminense Darcy Ribeiro, Av. Alberto Lamego 2000, Campos dos Goytacazes 28013-602, Brazil; ∥ Laboratório de Engenharia Agrícola, Centro de Ciências e Tecnologias Agropecuárias, Universidade Estadual do Norte Fluminense Darcy Ribeiro, Av. Alberto Lamego 2000, Campos dos Goytacazes 28013-602, Brazil; ⊥ Laboratório de Biologia Celular e Tecidual, Centro de Biociências e Biotecnologia, Universidade Estadual do Norte Fluminense Darcy Ribeiro, Av. Alberto Lamego, 2000, Campos dos Goytacazes 28013-602, Brazil; # Programa de Pós-graduação em Produtos Bioativos e Biociências, Universidade Federal do Rio de Janeiro, Avenida Aluízio da Silva Gomes, 50, Bloco C, Sala 106, Bairro da Glória, Macaé 27930-560, Brazil; ¶ Programa de Pós-graduação em Biociências e Biotecnologia, Universidade Estadual do Norte Fluminense Darcy Ribeiro, Av. Alberto Lamego 2000, P5, sala 224, Campos dos Goytacazes 28013-602, Brazil; ○ Unidade de Biologia Integrativa, Setor de Genômica e Proteômica, UENF, Av. Alberto Lamego 2000, P5, sala 224, Campos dos Goytacazes, Rio de Janeiro 28013-602, Brazil

## Abstract

The “spindle assembly checkpoint” (SAC) is a regulatory pathway that monitors the correct anchoring of the mitotic spindle microtubules to chromosomes during the metaphase–anaphase transition. The protein kinase monopolar spindle 1 (Mps1) is a key SAC component and is considered a promising target for antitumor drugs. This has led to the development of different inhibitory molecules that have helped to elucidate the Mps1 functions in the cell cycle. However, in plants, the catalytic mechanisms and roles of Mps1 during cell proliferation remain unknown. Here, we show that *Arabidopsis thaliana*’s Mps1 (AtMps1) has a similar catalytic structure to that observed in humans (*Homo sapiens*’ Mps1—HsMps1) and that its inhibition by SP600125 hinders postgerminative development. Further, our computational docking studies strongly suggest that both HsMps1 and AtMps1 interact with SP600125 in a similar manner and that plant proteins have topologically conserved protein–protein interaction motifs in their kinase domains. Furthermore, using *A. thaliana* as an experimental model demonstrates that Mps1 activity is essential for cell proliferation and postgerminative development and that SP600125 effects are reversible. Our experiments open new possibilities for understanding the mechanisms of the Mps1 protein using plants as experimental models. They also show that chemical genetics is a robust alternative for studies of plant development.

## Graphical abstract

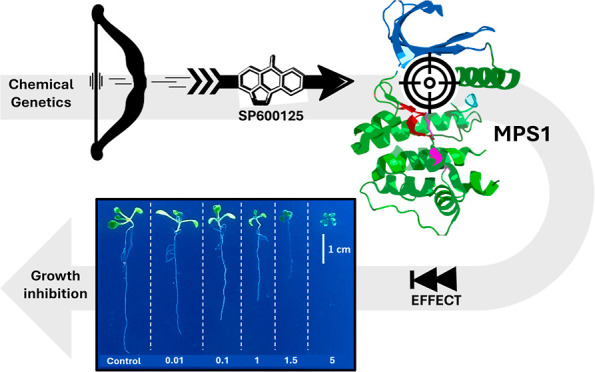

## Introduction

### Development and Cell Cycle

Plant development is strongly influenced by changes in the environmental conditions. Variations in light intensity, temperature changes, water availability, nutrient supply, and gravity can cause significant changes in plant development.^1,2^ A plant’s response to external stimuli results in several inter- and intracellular events that can directly affect the cell cycle.^3^ This directly impacts not only the biochemical signaling mechanisms but also the morphology of plants. Didactically, the cell cycle can be divided into four phases. The replication of genetic material is separated from the segregation of duplicated chromosomes. The G1 phase separates DNA replication (the S phase) from chromosome segregation (the M phase), also called mitosis. The G2 phase separates the S phase from the following M phase.^5^ During mitosis, the replication and segregation of chromosomes (karyokinesis), plasma membrane, and organelles occur, characterizing cytokinesis.^4^ The interruption phases allow operational control of the cycle, ensuring that the previous phases have been verified and completed.^3,5^ Although plants present special features in the cell cycle, such as cytokinesis,^6^ most of the protein components involved in the control of cell division are conserved in eukaryotes.^5,7^ Understanding the molecular mechanisms that govern the development of the eukaryotic cell cycle can contribute to an understanding of the mechanisms used by plants to promote their cellular homeostasis in response to environmental changes.

### Spindle Assembly Checkpoint

The different CDK/cyclin protein complexes control macro events in phase changes in the eukaryote cell cycle.^8,9^ However, during the G1/S, G2/M, and metaphase–anaphase transitions, there are specific molecular mechanisms called “checkpoints” responsible for monitoring, controlling, and rectifying these transitions.^10^ Also in mitosis, the metaphase–anaphase transition is the last monitoring point before cell division is completed. This event is marked by the segregation of sister chromatids and subsequent migration to the daughter cells. Errors in this process can lead to incorrect distribution of chromosomes and make daughter cells unviable. Chromosome segregation is finely monitored and controlled by a mechanism called the spindle assembly checkpoint (SAC). The main function of the SAC is to prevent premature progression to anaphase.^11,12^ Only when all chromosomes are correctly anchored and aligned are the signals triggered to complete cell division.^13^ Different steps of the SAC are executed by different protein complexes. For example, the anaphase-promoting complex/cyclosome^14^ (APC/C) and the complex formed by the proteins separase and securin.^15^ The proteins Mad2, Bub3, BubR1, and Cdc20 form the MCC complex (mitotic checkpoint complex), whose function is to inhibit Cdc20, a cofactor of the APC/C complex.^16^ This inhibition prevents the ubiquitination of securin and type B cyclins, which prevents their degradation by the proteasome. In turn, the proteolytic action of separase on the cohesin protein is inhibited by securin,^17^ and this keeps sister chromatids together.^18^ The effect of the sequential action of these complexes is the transition from metaphase to anaphase, and disturbances in the functioning of this mechanism can generate cells with different numbers of chromosomes.^19^ Different components of the SAC pathway have been identified in plant species, including Cdc20, Mad2, BubR1, and Bub3,^20^ components of the Aurora family,^21^ the APC/C complex,^22^ and Mps1.^23^ The results of these studies show that the respective structures of these components are conserved in plants, when compared to other eukaryotic models.^20,23^ Recent studies point to the STZ family of transcription factors as a potential link between stress response (biotic and abiotic) and cell cycle in *Arabidopsis thaliana*.^101^ However, how plants integrate this signal into fine control of the cell cycle remains unknown.

### Mps1 Protein in Plants

The Mps1 protein was first described^99^ by Winey et al. (1991) and was later characterized as a dual-activity kinase in humans.^24^ Initially, Mps1 was identified as important for the duplication of the mitotic spindle pole body.^25^ Later, its function in the mitotic SAC, which monitors the efficiency of chromosome segregation, was suggested.^26–28^ Subsequent studies on the structure and function of HsMps1 showed a complex phosphoregulation mechanism,^29–32^ increasing the possibilities of modulations in its different functions. In *A. thaliana*, the Mps1 protein is encoded by the AT1G77720 gene and contains 777 amino acids. Its kinase domain has the classical canonical conformation of a catalytic domain,^33^ formed by 293 amino acids, with a smaller N-terminal region composed of 5 β-sheets and a larger C-terminal region with 6 α-helix. This structure is similar to the HsMps1 kinase domain.^23,30^ In AtMps1, motifs related to nuclear localization and export occur.^23^ This suggests that AtMps1 can act in both the cytoplasm and the nucleus, as has been shown in humans.^34^ AtMps1 has the main characteristics of the Mps1 kinase family, such as the DFG motif (D568, F569, and G570), conserved in HsMps1. This motif is important in maintaining the conformation of the catalytic loop observed in the kinase domain of AtMps1. In HsMps1, the highly flexible glycine of this motif is essential for the conformation of the catalytic loop.^30^ In the region of the loop connecting the N- and C-terminal lobes, E499 is oriented in a way that suggests a function equivalent to that of E603 in HsMps1. However, very important is the occurrence of three threonine residues (T579, T580, and T590), whose equivalents in HsMps1 (T675, T676, and T686) were described as essential for autophosphorylation in the activation loop.^31,35^ The occurrence of these residues in the same region indicates structural similarity and strongly suggests functional similarity between HsMps1 and AtMps1. Furthermore, the structure of the catalytic and activation loop of AtMps1 appears to be conserved in different plant species.^23^ Interestingly, the protein kinase inhibitor SP600125 tested in HsMps1 ^36,37^ inhibited the postembryonic development of *A. thaliana* (Columbia-0) in a dose-dependent manner. This effect was also observed in seedlings induced with 5.0 μM indole acetic acid (IAA). The phenotype developed by these plants (characterized by an increase in lateral roots) was reversed with the addition of the inhibitor at a concentration of 1.0 μM IAA, suggesting the action of AtMps1 downstream of IAA signaling.^23^ It was also shown that the Mps1 inhibition by SP600125 affects the development of embryogenic cell cultures of *Araucaria augustifolia*.^38^

### Lateral Root Generation

In plants, at the end of embryogenesis, a complete miniature plant emerges with two groups of cells called meristems (apical and root). Through cycles of cell division and differentiation, the meristems can produce new organs such as leaves, flowers, stems (apical meristem), and roots (root meristem). In general, this is the process for the development of an adult plant.^3,7^ This peculiarity establishes the need to integrate a set of mechanisms for body modeling, development processes, and cell proliferation that can respond to a variable environment.^2^ Roots are the primary system for obtaining water, micro and macro nutrients, and sometimes have secondary functions such as storage of photosynthesized nutrients, synthesis of phytohormones, or clonal propagation.^39,40^ Thus, the root system directly influences the ability to obtain resources from the soil. Different endogenous and exogenous factors, biotic and abiotic, can affect this system and consequently influence plant development.^39,40^ The primary root arises during embryogenesis. However, the lateral (or secondary) root originates in postembryonic development in a group of pericycle cells stationed in G2.^41^ In this process, IAA acts to activate molecular mechanisms related to successive cell division cycles that will culminate in the emergence of a new root.^41–43^ Although great advances have been made in understanding these mechanisms,^42–46^ precise knowledge of how some pericycle cells respond to cell cycle activation and give rise to new roots remains unknown. Studies in plants have characterized and revealed the importance of the “core cell cycle”.^47–51^ However, several genes expressed in different phases of the cycle^52^ still have unknown functions. In this context and considering that blocking AtMps1 activity reduces lateral root formation, even with the addition of exogenous IAA,^23^ elucidating the role of AtMps1 in the cell cycle will help to understand how this process influences the formation of the *A. thaliana* root system. Here, we used chemical genetics with protein kinase inhibitor SP600125 to show the importance of the AtMps1 protein not only in cell division in *A. thaliana* but also in the mechanism of lateral root formation.

## Results and Discussion

### Mps1 Plant Interaction with SP600125

Chemical genetics is defined as the use of small molecules to disturb the function of a protein or biological system to explore the outcome.^53^ Because of its multiple functions in mitosis, HsMps1 has been proposed as a promising target for antitumor drugs and different inhibitors have been developed^54,55^ and used for its functional and structural characterization.^37^ SP600125 (anthrapyrazolone) is an ATP-competitive inhibitor. It was first described as a JNK inhibitor^56^ and later as an Mps1 protein kinase inhibitor.^57^ Our computational analysis suggests that AtMps1 SP600125 forms two hydrogen bonds between the N1 atom of the inhibitor and the NH group at the backbone of the Gly 501 residue (Gly 605 in HsMps1), and the N2 atom forms one hydrogen bond with the carbonyl oxygen of Glu 499 (Glu 603 in HsMps1). The theoretical binding distance was 2.72 Å from Gly 501 and 2.77 Å from Glu 499 (Figure 1A).

**Figure 1:**
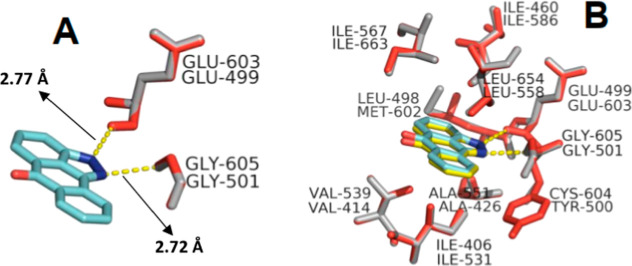
Overlap between HsMps1 (gray) and AtMps1 (red) catalytic site residues in interaction with SP600125. (A) The yellow dashed lines show hydrogen bonds, and arrows indicate the theoretical distance obtained. (B) Residues that form other interactions with SP600125 and contribute to the catalytic site’s hydrophobicity.

These values are close to those obtained in HsMps1 ^36^ (i.e., 2.91 Å and 2.82 Å, respectively). Molecular docking also revealed that the inhibitor is accommodated in the ATP binding site, stabilized by a strong hydrophobic component formed by the residues Ile 406, Val 414, Ala 426, Ile 460, Leu 498, Tyr 500, Leu 558, and Ile 567 (Figure 1B). These residues are equivalent to Ile 531, Val 539, Ala 551, Ile 586, Met 602, Cys 604, Leu 654, and Ile 663 of HsMps1.^36^ Active-site residues are conserved in the analyzed plant species (Table S1). Due to evolutionary well-conserved proteins, *A. thaliana* was proposed as an experimental model for biological mechanisms related to human health,^58^ and in this context, we include AtMps1. For example, in HsMps1, the residues Ile 531, Ile 598, Cys 604, and Ser 611 have been described as important in the resistance mechanisms to inhibitors.^59^ The corresponding residues Ile 406, Ile 494, Cys 500, and His 507 are conserved at the same positions in AtMps1. Two residues (Ile 531 and Ile 598) are conserved in other plant species (Table S1). A mutation replacing a cysteine with a tyrosine at position 604 adversely affects the interaction with some inhibitors.^59^ This change also occurs in other analyzed species (Table S1). This information provides new insights into conserved structural elements important for Mps1 interaction with different inhibitors.

### Post-translational Modifications in Plant Mps1

Motif identification^60^ combined with protein–protein interaction prediction^61^ provides a robust strategy in searching for new links in signaling pathways in plants. Here, we show that AtMps1 has interaction motifs with different proteins involved in post-translational modifications, such as phosphorylation and ubiquitination (Figure 2), and can interact with proteins related to the stress response and cell cycle.

**Figure 2:**
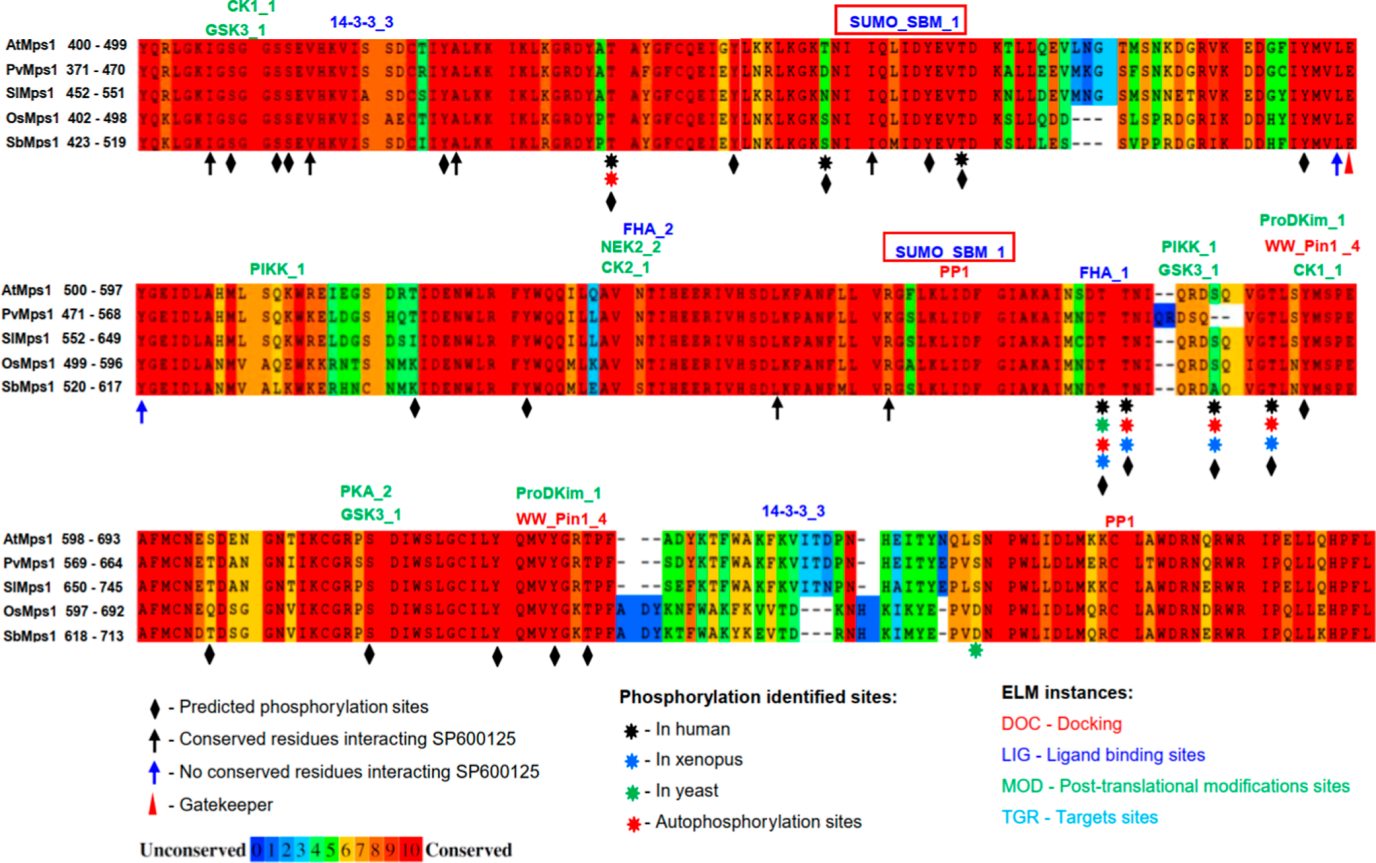
Mps1 kinase domain sequence alignment from five plant species: *Arabidopsis thaliana* (AtMps1), *Phaseolus vulgaris* (PvMps1), *Solanum lycopersicum* (SlMps1), *Oryza sativa* (OsMps1), and *Sorghum bicolor* (SbMps1). Red squares highlight well-conserved ubiquitination motifs and predicted phosphorylation sites.

Multisite phosphorylation in a protein expands its ability to interact with different enzymes or substrates in events such as labeling degradation, subcellular localization, enzymatic activity, and protein–protein interaction.^62,63^ Our results suggest that AtMps1 can be regulated by this mechanism (Figure 2 and Table S3), in the same way as for Mps1 in other eukaryotes. For example, in response to DNA damage, HsMps1 interacts with CHK2, which has an FHA domain.^64^ In plants, kinases with the FHA domain participate in cell cycle regulation, particularly in response to DNA damage.^65^ We showed that AtMps1 has the potential to interact with the FHA domain (Figure 2). Phosphoregulation requires phosphatase activity, and Mps1 is regulated in this way in different eukaryotes,^66,67^ including downregulation in SAC by PP1 phosphatase.^68^ In plants, PP1 acts on cell division, differentiation, cell cycle control, and other signaling pathways.^69^ The presence of the PP1_1 motif in AtMps1 suggests that it is a PP1 target. Ubiquitination and proteasomes from the “ubiquitin-proteasome system” (UPS) regulate different stages of mitosis in eukaryotes.^70,71^ In plants, UPS also acts in the abiotic stress response.^72^ In humans, degradation mediated by ubiquitination regulates the activity of Mps1.^73^ Small ubiquitin-related modifier (SUMO) promotes ubiquitination of proteins with subnuclear localization. In plants, “sumoylation” occurs in response to developmental hormones and biotic/abiotic stress.^74^ Sumoylation of HsMps1 regulates its subnuclear localization and function in SAC.^75^ AtMps1 has SUMO interaction motifs, suggesting that it is regulated by posttranslational modification. Cleavage of ubiquitin from its substrate (“deubiquitination”) negatively regulates UPS.^76^ USP7 is a deubiquitination enzyme, and its interaction motif (i.e., USP7_1) occurs in AtMps1 (Figure 2), suggesting that ubiquitination is a reversible mechanism for AtMps1 regulation. Thus, combining phosphorylation and ubiquitination, AtMps1 could act as a “convergence point” of molecular signals from different pathways, biotic and abiotic stress related to cell cycle control.

### Effect of SP600125 in *A. thaliana* Postgerminative Development

The detection of CYCB1;1 D-box GUS expression^77^ shows that the cell cycle progresses until the G2/M transition, even in plants treated with 1.0 μM SP600125. GUS activity was observed in leaf primordia and at the base of young leaves, suggesting a relationship between cell cycle, cell differentiation, and organogenesis in plants^78^ (Figure 3C,D).

**Figure 3:**
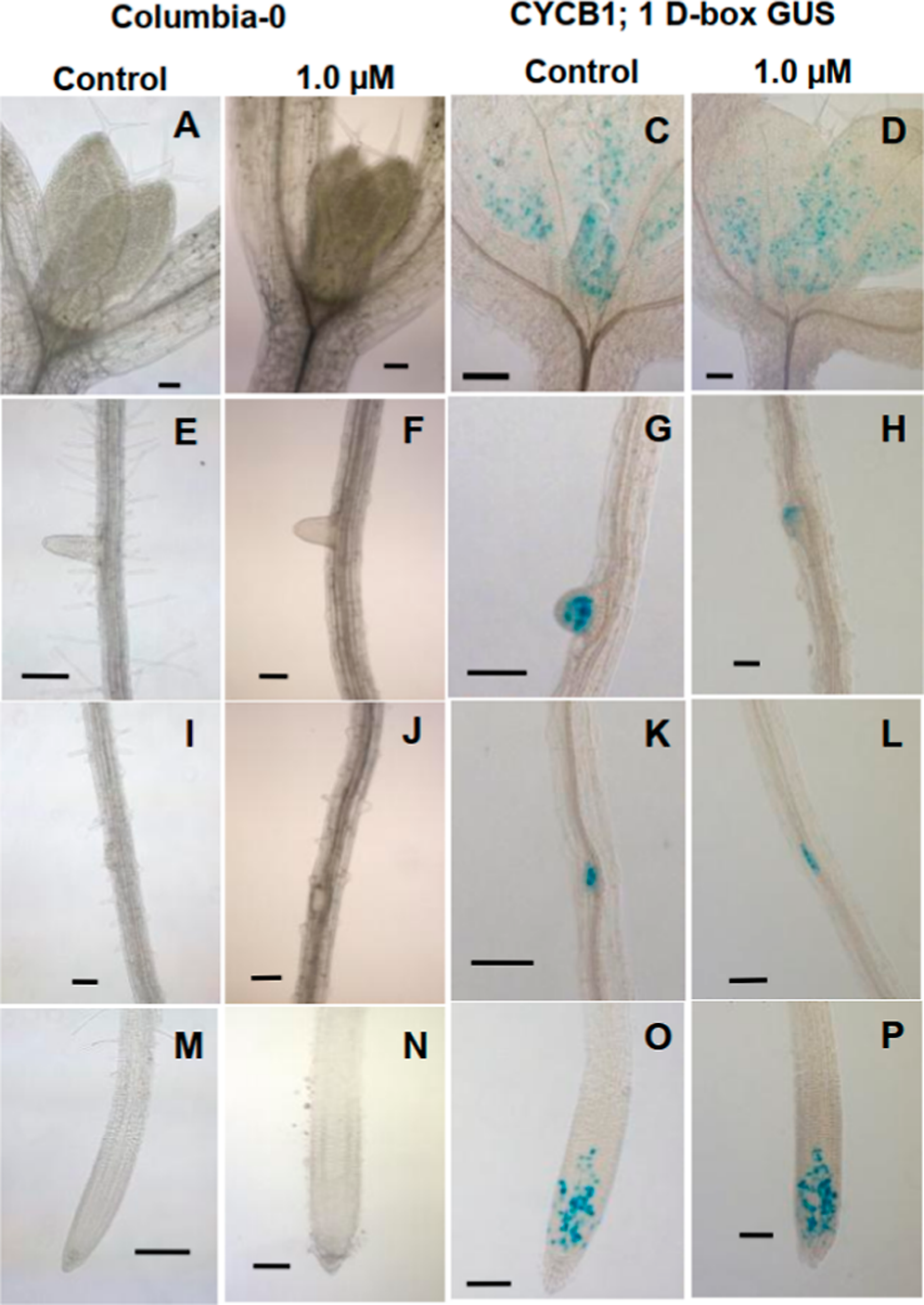
The effect of SP600125 on postgerminative development of different plant species. (A–P) Light microscopy of shoot meristems, lateral roots, lateral root primordia, and root apical meristems of *A. thaliana* plants after 7 days of growth (bars = 100 μm).

However, the same was not observed in the leaves and other mature tissues. We observed GUS activity in lateral roots (Figure 3G), root primordia (Figure 3H), pericycle cells (Figure 3H), and root meristematic proximal zone (Figure 3O,P). We did not observe GUS activity in the transition regions (TZ) or in the elongation and differentiation zones (ZED). In humans, Mps1 arrests the SAC pathway during the metaphase–anaphase transition, and it was recently shown that SP600125 affects cellular growth and morphology in somatic embryogenesis in *Araucaria angustifolia* embryogenic cell suspension cultures, indicating an important role of Mps1 in this process.^38^ In *A. thaliana*, lateral roots originate from pericycle cells, which are stationed in G2.^79,80^ This mechanism is triggered by increased levels of Aux/IAA in these cells.^43^ However, the molecular mechanisms that connect these two events remain unknown. SP600125 reduces root primordia formation and prevents the action of Aux/IAA, indicating that AtMps1 acts downstream of IAA signaling.^23^ We showed that root development can be reversed by removing the inhibitor (Figure 4A–D), demonstrating one of the best experimental possibilities of chemical genetics: reversibility. This can be seen in label-free proteomic analysis in samples taken from roots (120 h) and treated with 1.0 μM SP600125. In total, 1665 coexpressed proteins from different functional groups were identified (Figure S1A–C), and just over 10% do not recover their relative levels (113 up- and 65 downregulated proteins, respectively) when compared to the control (Table S4). A fluorometric assay with purified Mps1 kinase domain was performed to confirm that SP600125 inhibits the protein. Four concentrations of the inhibitor were used: 0.01, 0.1, 1.0, and 10.0 μM. This analysis demonstrates the high specificity of the inhibitor and its in vitro activity (Figure S2). Thus, our results show that SP600125 can be used in studies of Mps1 protein function and postgerminative development of *A. thaliana*. We also show that Mps1 acts on different tissues of *A. thaliana* that have cell cycle activity, suggesting a role in the integration of different signaling pathways for perception of a variable environment (Figure S1D).

**Figure 4:**
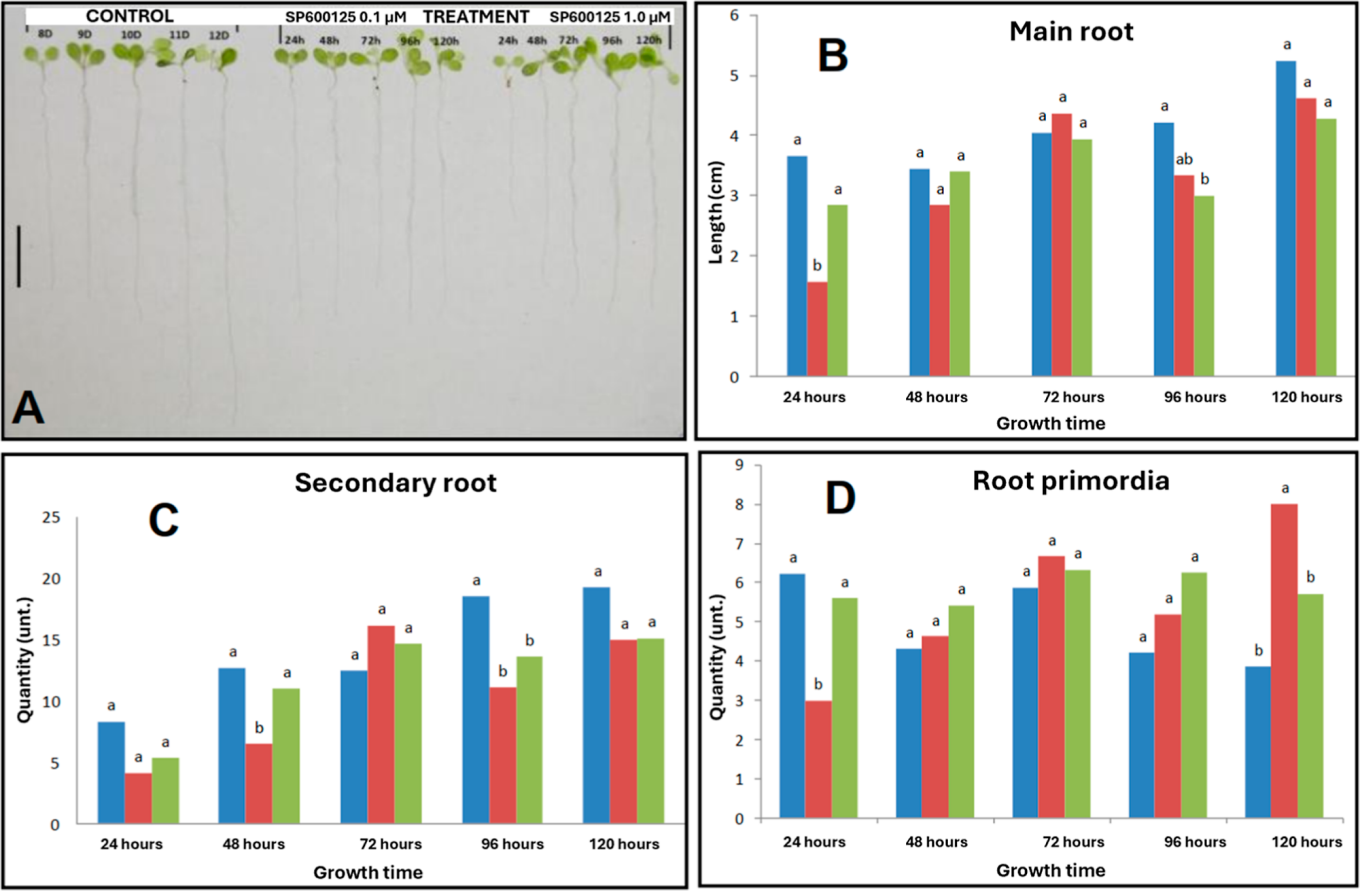
Potential reversibility evaluation of the growth inhibition effect caused by SP600125. (A) Overall plant morphology (bar = 1 cm). (B) Main root length. (C) Number of secondary roots and (D) number of root primordia. Color bars indicate plant treatments: blue = control; red = 0.1 μM SP600125; green = 1.0 μM SP600125.

### Molecular Docking Predictions in the AtMps1 Catalytic Site

Besides SP600125, two other inhibitors were used in silico to determine the interaction profiles with the AtMps1 kinase domain: Reversine and MPS1-IN-2. They have, respectively, 8 and 12 more carbons in their structure than SP600125 (C_14_H_8_N_2_O). Some amine groups (NH) can be observed in both molecules, and particularly, MPS1-IN-2 exhibited two more hydroxyl groups (OH) than SP600125 (Figure 5A–C).

**Figure 5:**
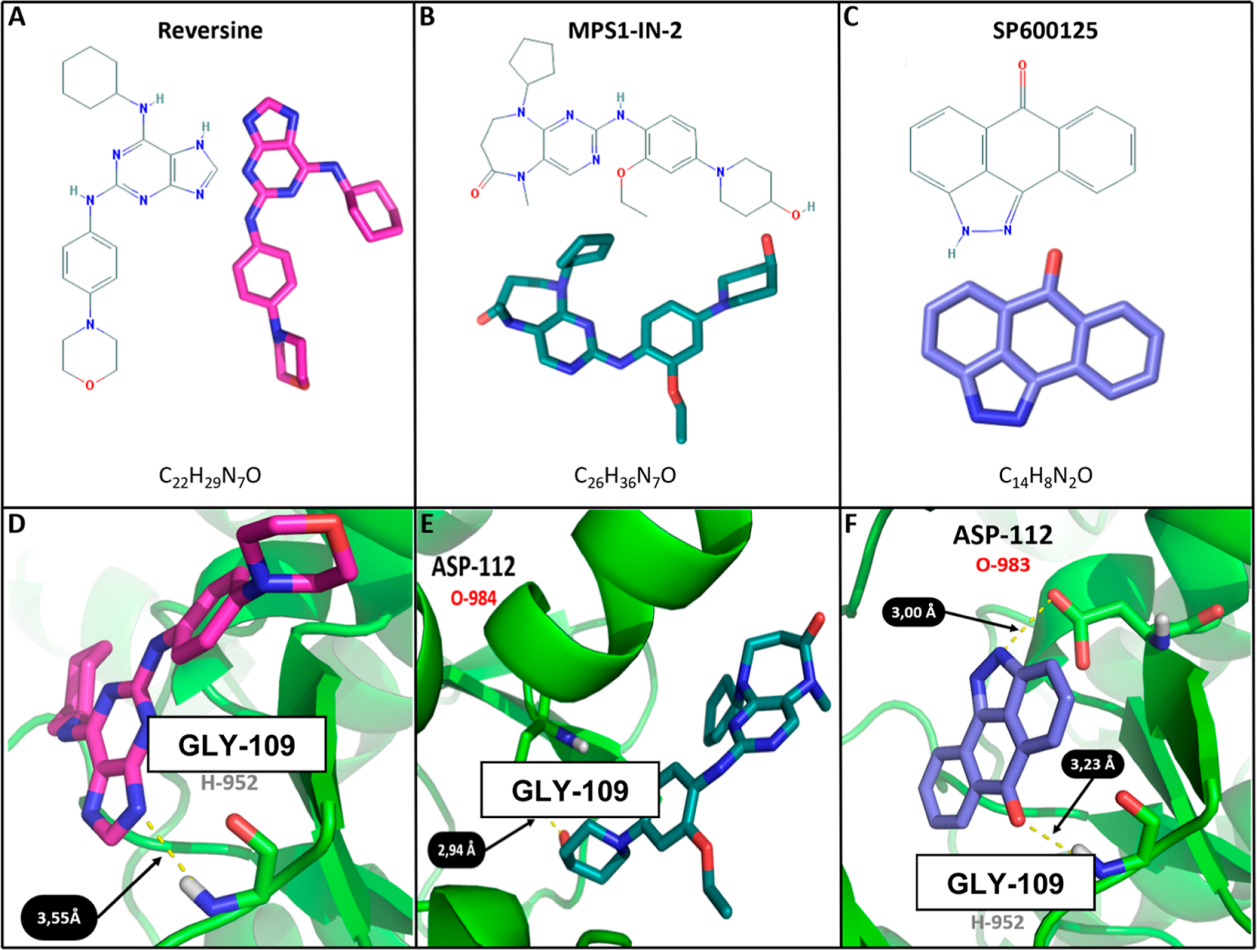
Chemical structure and molecular docking of three different Mps1 inhibitors. (A–C) Chemical and conformational structures of Reversine, Mps1-IN-2, SP600125, and their atomic details. (D–F) Poses and hydrogen bonds (yellow dashed lines) found in docking simulation between the three ligands and *A. thaliana*’s Mps1 catalytic site. Nitrogen and oxygen atoms are represented in dark blue and red, respectively. Hydrogens are represented in gray and carbon atoms are in colors.

SP600125 demonstrated a more robust ability to form bonds with the conserved residues Gly 109 and Asp 112 in the AtMps1 catalytic site. It was able to form two hydrogen bonds, 3.23 Å from the hydrogen-952 (H-952) in Gly 109 and 3.0 Å from the oxygen-983 (O-983) in ASP112 (Figure 5F). The same quantity of hydrogen bonds was not observed with Reversine and Mps1-IN-2, but intriguingly, they formed, respectively, a single hydrogen bond with the H-952 in Gly 109, and with the O-983 in Asp 112. The bond distance was sensibly greater in Reversine (3.55 Å) and shorter in MPS-IN-2 (2.94 Å), in comparison to SP600125 (Figure 5D,E). Further analysis has shown that the three inhibitors were able to form other interactions with residues in the AtMps1 catalytic site, such as Pi–Pi, Pi-sigma, Pi-alkyl, Pi-anion, and Pi-stacked (Figure S6). These interactions involved some conserved residues: Ile 14, Val 22, Ala 34, Glu 107, Tyr 108, Gly 109, Glu 110, Ile 111, Asp 112, His 115, Leu 166, and Ile 175, common in dual-specificity kinases—DSK,^81^ the protein family of which Mps1 is a member. Basically, these residues are responsible for making a favorable chemical environment at the catalytic site for substrate docking.

### Morphological Effects of Inhibitors in the *A. thaliana* Postgerminative Development

As observed in molecular docking, the postgerminative experiments conducted with *A. thaliana* seedlings also demonstrated a more pronounced effect of SP600125 in their development (Figure 6).

**Figure 6:**
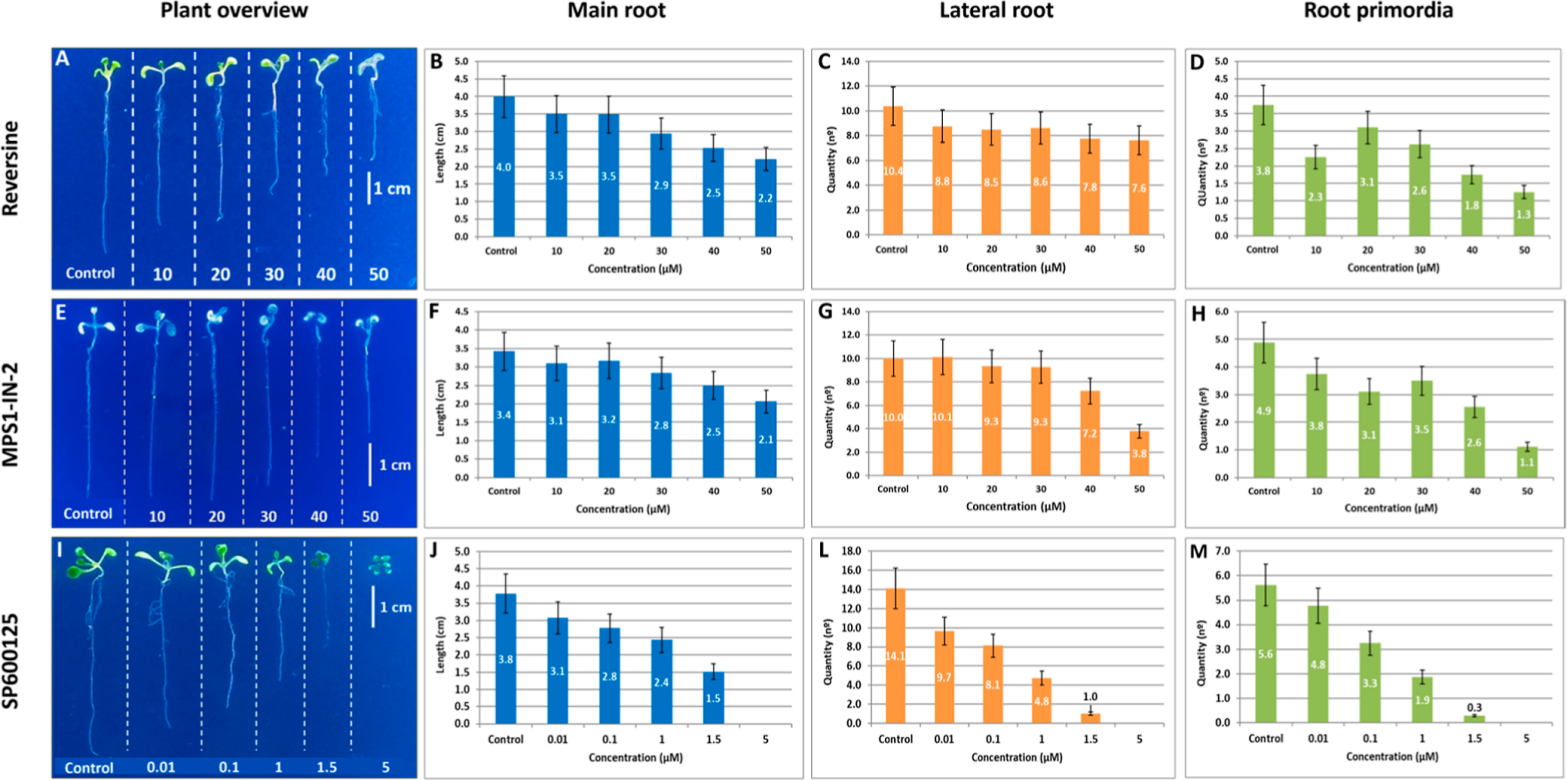
Effect of Mps1 inhibition in postgerminative development of *A. thaliana*. (A,E,I) Overview of *A. thaliana* seedlings after 7 days of development. All values indicate the arithmetic mean of each feature expressed. Blue, orange, and green columns indicate, respectively, the length of the main root (cm), the quantity of lateral roots (total count), and the quantity of root primordia (total count) for each inhibitor’s concentration tested.

This inhibitor was able to affect vegetative growth more effectively, even using 10× lower concentrations than those used in both Reversine and MPS1-IN-2. Plants treated with 1.0 μM SP600125 produced approximately 1.8× fewer lateral roots than plants treated with 10 μM Reversine, and 2.1× fewer than plants treated with 10 μM MPS1-IN-2. SP600125 also demonstrated a sublethal concentration at 5.0 μM. This permitted initial leaves to grow but not roots, while the greater concentration used in Reversine and MPS1-IN-2 (50 μM) treatments permitted plants to produce, respectively: 2.2 and 2.1 cm of main root; 7.6 and 3.8 of lateral roots (total count); 1.3 and 1.1 of roots primordia (total count), thus letting plants survive, differently from those treated with SP600125 in sublethal concentration. In terms of plant root absorption, larger roots mean greater capability of water and nutrient absorption. In comparison with the control, seedlings treated with 1.5 μM SP600125 have shown a difference of 2.3 cm between their main root length (Max 3.8 – Min 1.5 = 2.3 cm). It means an average loss of 60.5% in root absorption capability. Using the same calculations for Reversine and MPS1-IN-2, it gives an average loss of 45.0% and 38.2% respectively. All these data suggest that SP600125 has more affinity for the AtMps1 kinase domain than Reversine and MPS1-IN-2, causing a more pronounced effect in plant growth.

## Conclusion

The JNK protein was first identified by its ability to phosphorylate the transcription factor c-Jun in mammalian cells, and it is encoded by three genes called JNK1, JNK2, and JNK3 ^82^ (Barr & Bogoyevitch, 2001). JNKs are components of the MAPK signaling pathway^82,83^ (Barr & Bogoyevitch, 2001; Dunn et al., 2002) and are related to the response to different types of stress,^84,85^ such as UV irradiation, hyperosmolarity, heat shock, pathogen response (intra and extracellular), hormones, cytokines, DNA damage, and oxidative stress.^102–104^ In eukaryotes, MAP kinase signaling pathways are evolutionarily conserved,^86^ and in plants, they are present in a greater diversity of components.^87^ The Mps1 protein plays a central role in cell division control, as demonstrated by studies in different eukaryotes, such as *Saccharomyces cerevisiae,* ^25^ *Xenopus laevis,* ^28^ *Danio rerio,* ^88^ *Homo sapiens,* ^89^ and *Drosophila melanogaster*.^90^ Due to its importance in cell division control, Mps1 has become an important target for molecular studies, aiming at antitumor drug development.^91–96^ In addition, different inhibitors have also been used to functionally characterize this kinase in cell division control mechanisms.^97,100^ Here, we combined chemical genetics, molecular docking, proteomics, and in vitro activity to demonstrate the importance of AtMps1 in the postgerminative development of *A. thaliana*. We show that the inhibitor SP600125, initially characterized as an inhibitor of JNK in cell culture^56^ and later tested as an inhibitor of human Mps1,^57^ is predicted to form a complex with AtMps1 in a manner similar to that of HsMps1.^36^ Furthermore, the kinase activity of AtMps1 is also inhibited by SP600125 in vitro, as occurs with HsMps1. We show that the postgermination effects of SP600125 occur by blocking the cell cycle at the G2/M transition, where AtMps1 is expressed.^52^ We also showed that by using SP600125, we can delay postgermination development of *A. thaliana* seedlings and then resume normal growth when removing the inhibitor. We do not exclude the possibility of inhibition of some MAPK (JNK) family members by SP600125 in *A. thaliana*. However, as reported by Schmidt et al. 2005,^57^ the cell division arrest effects are quite significant. Furthermore, disarrangement of biotic and abiotic stress response can disrupt and even arrest cell cycle progression in plants^98^ and thus act before the G2/M phase. In this context, detection of CYCB1;1 expression shows that division progression occurs up to the G2/M transition. This suggests that if SP600125 inhibited any member of the stress signaling pathways, this inhibition did not affect the cell cycle progression. Further, it was already established in a previous study^23^ that AtMps1 is not an MAPK nor an MAPK paralog but indeed an HsMps1 ortholog. Further studies are needed to evaluate the effects of SP600125 on MAPK pathways in response to different types of stress (biotic and abiotic) and to determine whether these effects can block these signaling pathways in plant cells. Our findings not only contribute to advances in knowledge about the control of cell division in plants but also present a new use for the tested inhibitor, namely, using it as a reversible growth controller. In the future, by combining studies of protein structure and function and chemical synthesis, we may initiate a new generation of selective growth controllers that act at specific points of cell cycle control in different plant species.

## Materials and Methods

### Plant Materials, Culture Media, and Growth Conditions

All experiments were conducted with *A. thaliana* seeds (Columbia-0). Transgenic *A. thaliana* expressing CYCB1;1/D-box/GUS was kindly supplied by Dr. Paulo Ferreira and Dr. Adriana Hemerly (Plant Molecular Biology Laboratory IBqM/UFRJ). *A. thaliana* seeds were sown in six-well plates with 2 mL of liquid 1/2 MS culture medium, prepared as follows: MS salts (Sigma-Aldrich) supplemented with 5 g/L sucrose and 0.25 g/L MES. The pH was adjusted to 5.8 with KOH 1 M. *A. thaliana* plants were kept under a 16/8 h photoperiod at 22 °C.

### *A. thaliana* GUS Staining

After the inhibition assay, transgenic *A. thaliana* seedlings expressing CYCB1;1/D-box/GUS were recovered from the six-well plates, washed, and submitted to a GUS staining method as follows: first all seedlings were washed with distilled water and then kept in the staining solution (1 M Na_2_HPO_4_, 1 M NaH_2_PO_4_, 0.5 M EDTA, 10% Triton X-100, 50 mM K_3_Fe­(CN)_6_, and 100 mM X-Gluc, pH 7.0) for 24 h at 37 °C. After the incubation period was over, the staining solution was substituted for a fixing solution (3:1 ethanol and acetic acid), and the seedlings were stored at 4 °C overnight. Following storage, the material was then incubated in 50% lactic acid to remove any residual coloring and improve GUS visualization under the microscope.

### SP600125 Reversibility in the *A. thaliana* Assessment Experiment

This experiment was designed to evaluate whether inhibition caused by SP600125 is reversible compared to that of plants having the same time to develop. The reversibility of the inhibitory effect caused by SP600125 was evaluated in a germination experiment with *A. thaliana* seeds. For this experiment, seeds were germinated in liquid 1/2 MS culture medium with 0.1 and 1 μM SP600125 for 7 days. After this period, the inhibitor was removed by washing the seedlings three times with fresh culture medium. After inhibitor removal, seedlings kept under the same growth conditions were collected in intervals of 24 h up to 168 h (7 days + 24 h, and so on) and conserved in fresh fixative solution (0.05 M sodium cacodylate, 4% formaldehyde, glutaraldehyde 2.5%, pH 7.2). Control seedlings were collected after 8 days in 24 h intervals up to 12 days of growth. Three morphological parameters were evaluated: main root length (cm), number of secondary roots, and number of root primordia. Root length was measured with the aid of ImageJ v1.45s and SmartRoot v4.1 plugin. Root length and root primordia were counted under light microscopy using an Olympus BX51 setup with an Olympus DP71 image capture system. Statistical analysis was conducted using a Lilefors test coupled with a Cochran test at the level of 5% significance (*P* < 0.05) using the SAS statistical analysis package.

### Mps1 In Vitro Inhibition

To confirm that SP600125 inhibits Mps1, we used a purified kinase domain (supplied by Agrisera) and measured its activity with a Universal Fluorometric Kinase Assay Kit (Sigma-Aldrich/MAK173, St. Louis, MO, USA) under inhibition. 10 ng of purified Mps1 kinase domain was mixed in a reaction buffer (50 m M246 Tris–HCl, pH 7.4, 0.2 M NaCl, 10 mM MgCl_2_, 1 mM DTT, and 100 μM ATP) with increasing concentrations of SP600125 (0.01, 0.1, 1, and 10 μM). Dimethyl sulfoxide 0.04% (v/v) was used as a control. Final reaction volume, including all reagents supplied by the kit, was 50 μL. All reactions were distributed in black 96-well microplates and incubated for 15 min, and then the fluorescence intensity (λ_ex_ = 544 nm/ λ_em_ = 590 nm) was monitored in a Hidex Plate Chameleon V 425–106 multilabel counter in 15 min intervals up to 1 h. All reactions were performed in duplicate (as recommended by the manufacturer), and average intensity values were considered.

### Molecule Features

Inhibitor features are as follows: Reversine (IC_50_: 40 nM|molecular weight: 393.495 g/mol), Mps1-IN-2 (IC_50_: 140 nM|molecular weight: 480.613 g/mol), and SP600125 (IC_50_: 692 nM|molecular weight: 220.231 g/mol).

### Molecular Modeling and Docking Calculations

Tridimensional structures of Mps1 kinase domain from *A. thaliana* were obtained with Modeler v9.14 using 4 templates, PDB ID: 2ZMD/2ZMC, 3HMN, and 3DBQ.^2–4^ Molecular docking calculations with SP600125 were then performed by using AutoDockTools v1.5.65. For the docking calculations, the grid center was positioned on the GLY501 residue inside the active-site pocket using the O_2_ atom as the starting position for the ligand (SP600126 molecule). The 2D docking interaction maps were built with BIOVIA|Discovery Studio Visualizer v16.1. All 3D solutions were evaluated, and figures were generated with PyMOL v1.3 (Schrödinger, LLC) with the AutoDock plugin.^6^

### Proteomic Analysis from *A. thaliana* Plants Submitted to a Reversibility Assay

#### Reversibility Experiment and Protein Extraction

Proteomic analysis was performed to further investigate the effects of SP600125 on the postgerminative development of *A. thaliana* seedlings. In this experiment, *A. thaliana* seeds were germinated in 1/2 MS culture medium (see growth conditions above) containing 0.1 and 1 μM SP600125 and kept growing for 5 days. After this first development period, all the seedlings were washed with fresh culture medium free of SP600125 and then allowed to grow for five more days under the same conditions (at 22 °C, 16/8 h photoperiod). Each treatment (including control groups) consisted of three replicates for a total of 900 seedlings (300 seedlings for each replicate). After the growth period, all seedlings were recovered. Roots from all seedlings were collected, frozen in liquid nitrogen, ground to a fine powder, and stored at −20 °C. Approximately 300 mg fresh weight (FW) of each sample was submitted to protein extraction.^7^ All samples were homogenized in 1 mL of extraction buffer (7 M urea, 2 M thiourea, 2% Triton X-100, 1% DTT, 1 mM PMSF, 5 μM pepstatin), incubated for 30 min on ice, and centrifuged at 16,000*g* for 20 min at 4 °C. The supernatant was recovered and quantified using the 2-D Quant Kit (GE Healthcare, Piscataway, NJ, USA).

#### Protein Digestion

Protein digestion was conducted as described.^8^ Before the trypsin digestion step, 100 μg protein aliquots of each biological sample were desalted on 5000 MWCO Vivaspin 500 membranes (GE Healthcare) using 50 mM ammonium bicarbonate (Sigma-Aldrich) (pH 8.5) as a buffer. Each sample was incubated at 80 °C for 15 min in 25 μL of 0.2% RapiGest (Waters, Milford, CT, USA). After this first step, 100 mM DTT was added, and samples were homogenized and then incubated for 30 min at 60 °C under agitation. Following this step, 300 mM iodoacetamine was added and the samples were allowed to rest for 30 min at room temperature in the dark. The digestion step was performed overnight at 37 °C by adding 50 ng/μL (Promega, V5111, Madison, WI, USA) of trypsin to each sample. After digestion, RapiGest precipitation was performed for 90 min at 37 °C after the addition of 5% (v/v) trifluoroacetic acid (TFA). All samples were centrifuged for 30 min at 16,000*g* and then transferred to Total Recovery Vials (Waters, Manchester, UK).

#### Mass Spectrometry Analysis

ESI-LC-MS/MS analysis was performed in a nanoAquity UPLC instrument connected to a Synapt G2-Si HDMS mass spectrometer (Waters, Manchester, UK). The normalization step was performed based on stoichiometric measurements of total ion counts of scouting runs in MSE mode. For separation, all samples were loaded into a C18 trap column for 3 min and then transferred to the nanoAquity HSS T3 reversed-phase column at 60 °C. The elution step was performed with a binary gradient consisting of 2 mobile phases (mobile phase A: water and 0.1% formic acid and B: acetonitrile and 0.1% formic acid). Elution started at 7% (solution B) for 3 min and then ramped up to 40% (solution B) for 90.09 min and then to 85% up to 98.09 min. The gradient was decreased to 7% (solution B) at the 100.09 min mark and then kept at 7% for the rest of the run until the end at 108.09 min. Mass spectrometry was performed in V mode (positive resolution) at 35,000 fwhm with ion mobility and data-independent acquisition (DIA) (HDMSE). The wave velocity was 600 m/s, and the transfer collision mode escalated from 19 to 45 V in high-energy mode. Cone and capillary voltages were set to 30 and 2800 V, respectively, at 70 °C. The time of flight (TOF) scan time was set to 0.5 s in continuum mode with a mass range of 50–2000 Da. Human [Glu1]-fibrinopeptide B (100 fmol/ μL) was used as a standard for calibration purposes, and lock-mass acquisition was performed every 30 s.

#### Bioinformatics Analysis of MS Data

Progenesis Qi for Proteomics V2.0 was used for spectra processing and database searching conditions using the following parameters: one missed cleavage, minimum fragment ion per peptide equal to 1, minimum fragment ion per protein equal to 3, minimum peptide per protein equal to 2, fixed modifications of carbaminomethyl (C) and variable modifications of oxidation (M) and phosphoryl (STY) and a default false discovery rate (FDR) value at 4% maximum, score greater than 5, and maximum mass errors of 10 ppm. To ensure the quality of results after progenesis analysis, only proteins present in 3 out of 3 biological samples were included in the final list. Functional annotation of gene ontology terms was performed with Blast2Go v3.0.

## Protein Accession IDs

- PDB ID: 2ZMD
- PDB ID: 2ZMC
- PDB ID: 3HMN
- PDB ID: 3DBQ

## Supplementary Material



## Notes

Gene Ontology analysis from *A. thaliana* seedlings after SP600125 inhibition recovery (Figures S1 A–C); proposed Mps1 signaling pathway­(Figure S1D ; Mps1 inhibition assay with SP600125 (Figure S2); Mps1 active-site residues interaction maps (Figure S3 A–C); catalytic site residue comparison between different plants Mps1 (Table S1); annotated Mps1 protein–protein interactions (Table S2); and *A. thaliana* Mps1 phosphorylated residues computational prediction (Table S3) (PDF)
