# Supplementary material for: Chemical Genetics with SP600125 Reveals That Mps1 Protein Kinase Works as a Regulatory Element in Post-embryonic Development of the Arabidopsis thaliana Root System—An Insight into Plant Cell Cycle Control
Source: ACS Omega. 2025 Oct 27;10(43):51900–11. doi: 10.1021/acsomega.5c07325 (PMC12593080; doi:10.1021/acsomega.5c07325)
Supplement: Supplementary file 1 [file ao5c07325_si_001.pdf]

Chemical genetics with SP600125 reveals that Mps1 protein kinase works as a regulatory element in post embryonic development of *Arabidopsis thaliana* root system - an insight into plant cell cycle control

Emanuel Victor Nogueira Gotardo [a], Eduardo Alves Gamosa de Oliveira [b], Lucas Zancheta Passamani [c], Izabela Silva dos Santos [a], Geraldo do Amaral Gravina [d], Claudete Santa-Catarina [e], Vanildo Silveira [c], Antônia Elenir Amâncio Oliveira [b], Marco Antônio Lopes Cruz [a]*

[a] Laboratório de Biotecnologia Vegetal, Universidade Federal do Rio de Janeiro. Av. Amaro Reinaldo dos Santos Silva, 764, São José do Barreto, Macaé, Rio de Janeiro, 27965-045, Brasil.

[b] Laboratório de Química de Função de Proteínas e Peptídeos, Centro de Biociências e Biotecnologia, Universidade Estadual do Norte Fluminense Darcy Ribeiro. Av. Alberto Lamego 2000, P5, sala 224, Campos dos Goytacazes, 28013-602, Brasil.

[c] Laboratório de Biotecnologia, Centro de Biociências e Biotecnologia, Universidade Estadual do Norte Fluminense Darcy Ribeiro. Av. Alberto Lamego 2000, Campos dos Goytacazes, 28013-602, Brazil.1

[d] Laboratório de Engenharia Agrícola, Centro de Ciências e Tecnologias Agropecuárias, Universidade Estadual do Norte Fluminense Darcy Ribeiro. Av. Alberto Lamego 2000, Campos dos Goytacazes, 28013-602, Brasil.

[e] Laboratório de Biologia Celular e Tecidual, Centro de Biociências e Biotecnologia, Universidade Estadual do Norte Fluminense Darcy Ribeiro. Av. Alberto Lamego, 2000, Campos dos Goytacazes, 28013-602, Brasil.

*** E-mail:** Marco Antônio Lopes Cruz (marcocruz@macae.ufrj.br);

Key words: Mps1, Spindle Assembly Checkpoint (SAC), SP600125, Plant cell cycle

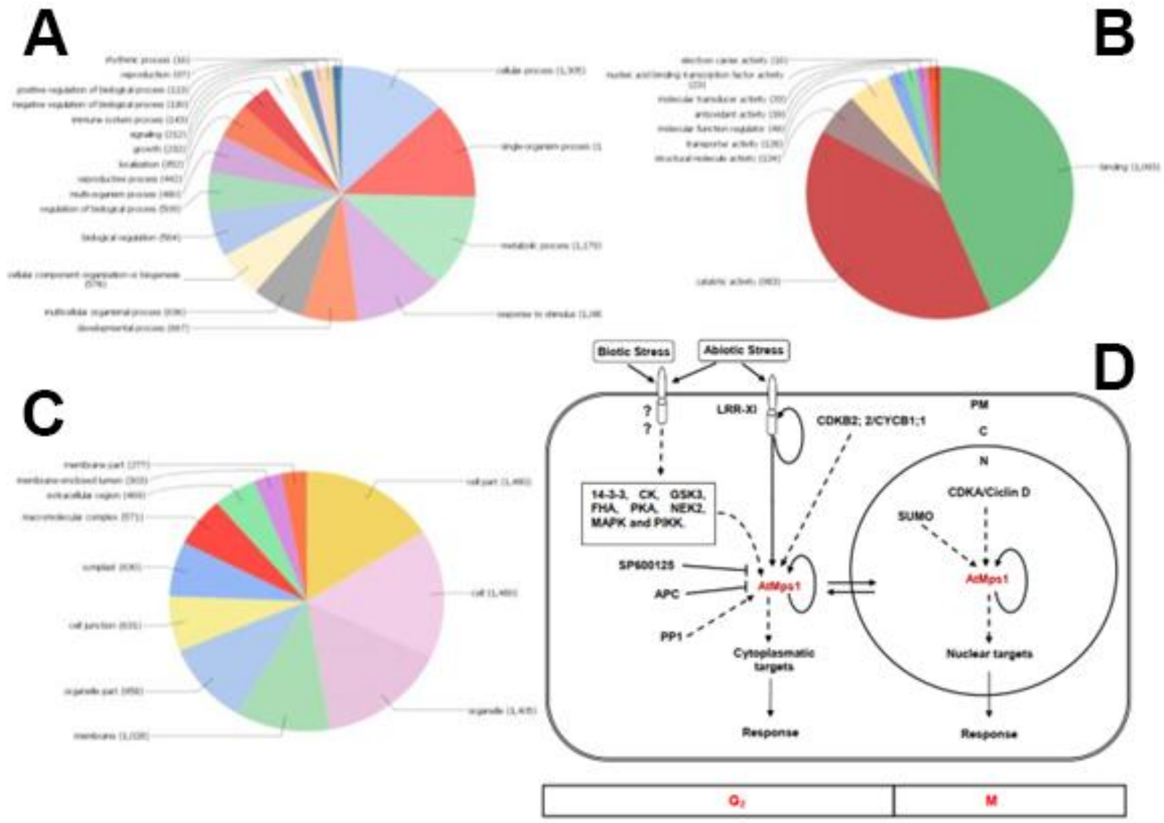

Figure S1 - (A) Biological processes with neutral regulation after proteomic analysis of *Arabidopsis thaliana* roots submitted to reversibility experiment. (B) Molecular functions of different proteins identified from *A. thaliana* root after reversibility experiments. (C) Cellular localization of different proteins identified from *A. thaliana* roots after reversibility experiments. (D) Proposed model for AtMps1 role(s) over cell cycle regulation.

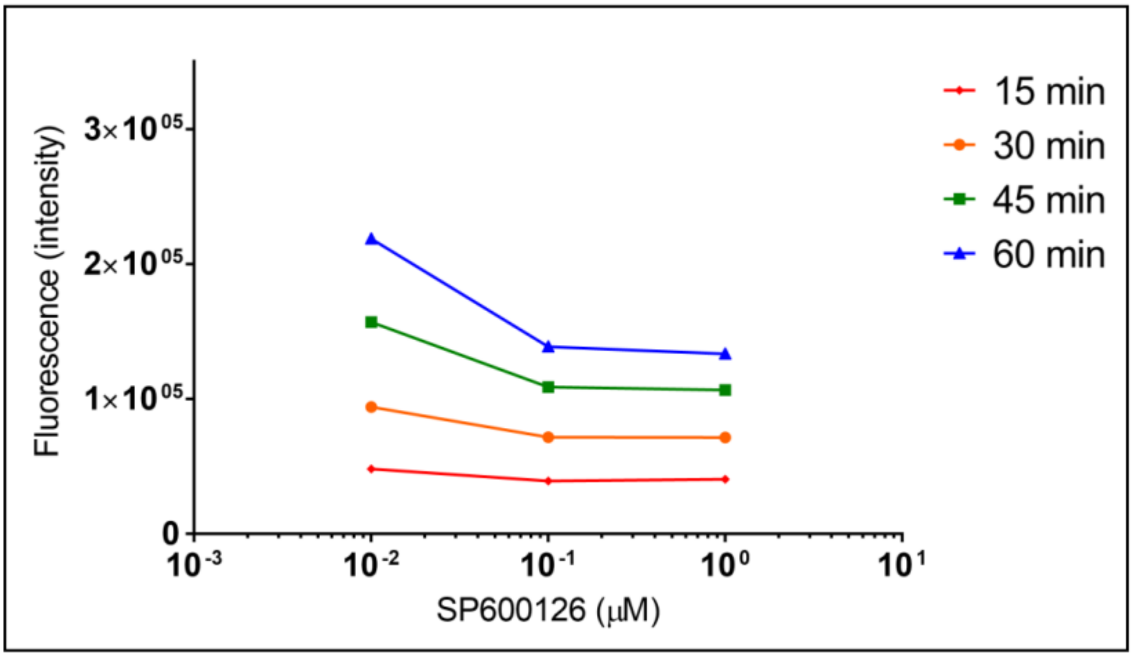

Figure S2 – Fluorometric assay conducted with Mps1. It was used 10 ng of purified kinase domain of Arabidopsis thaliana Mps1 (AtMps1) with Universal Fluorimetric Kinase Assay Kit (Sigma-Aldrich/MAK173, St. Louis, MO, USA) under inhibition and with increasing concentrations of SP600125.

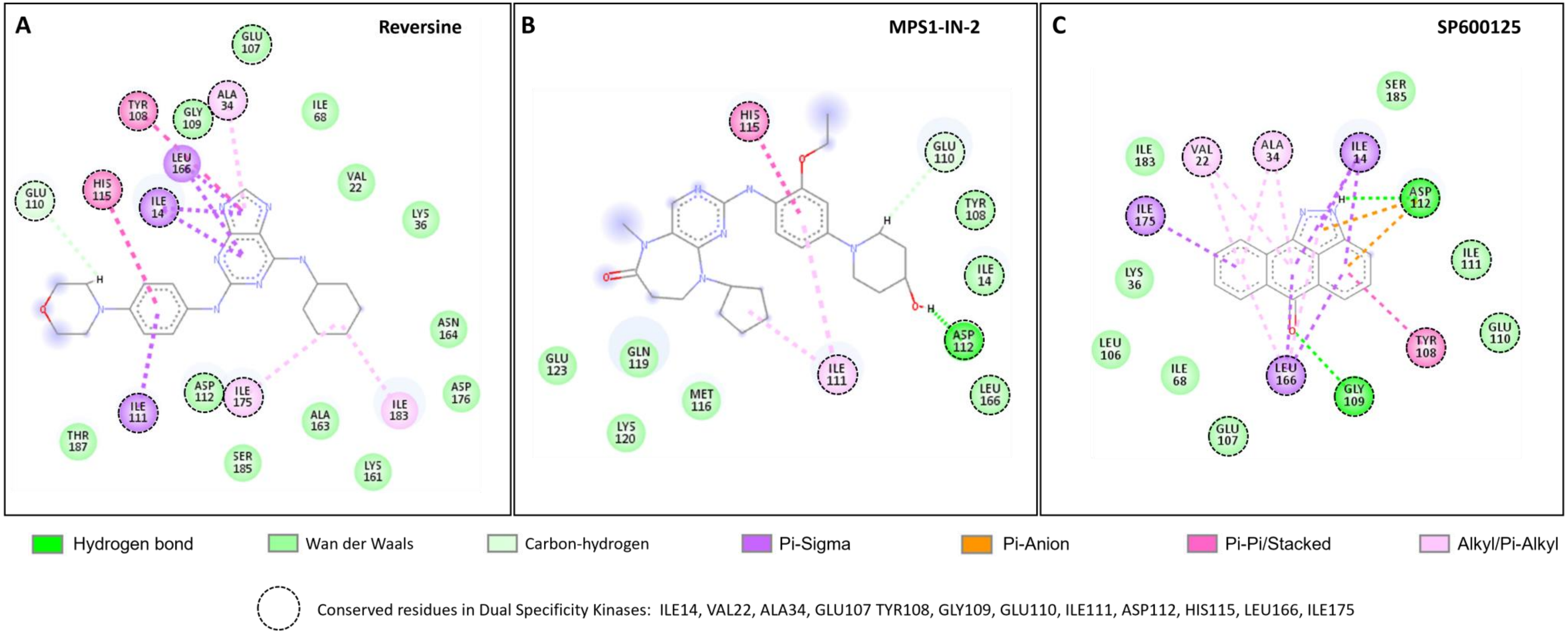

Figure S3 – Hydrogen bonds and other interactions formed between conserved residues in AtMps1 catalytic site and the three inhibitors (A-C). Dashed lines represent bonds, and their absence represents interactions.

ARABIDOPSIS	SORGHUM	TOMATO	HUMAN
ILE 406	ILE 429	ILE 458	ILE 531
VAL 414	VAL 437	VAL 466	VAL 539
ALA 426	ALA 449	ALA 478	ALA 551
ILE 460	ILE 483	ILE 512	ILE 586
LEU 498	LEU 518	LEU 550	MET 602
TYR 500	TYR 520	TYR 552	CYS 604
LEU 558	MET 578	LEU 641	LEU 654
ILE 567	ILE 587	ILE 650	ILE 663
GLU 499	GLU 519	GLU 551	GLU 603
GLY 501	GLY 520	GLY 553	GLY 605

Table S1 – Conserved residues in Mps1 catalytic site from human and three plant species. Correspondent residues in each organism are horizontally represented. Green cells represent the residues that form hydrogen bonds with the inhibitor SP600125. Yellow cells highlight different residues in HsMps1 in comparison with plant Mps1.

	Biological functions	Accession	Protein	Ref.
**1**	Cell cycle/SAC	AT1G78770	APC6	18
**2**	Cell cycle/SAC	AT2G20000	HOBBIT	25
**3**	Cell cycle/SAC	AT2G25880	AUR2	29
**4**	Cell cycle/SAC	AT3G25980	MAD2	7
**5**	Cell cycle/SAC	AT3G48750	CDKA;1	32
**6**	Cell cycle/mitosis	AT2G27960	CKS1	11
**7**	Cell cycle/mitosis	AT2G27970	CKS2	30
**8**	Cell cycle/cytokinesis	AT2G45490	AUR3	29
**9**	Cell cycle/ meiosis	AT5G19400	SMG7	6
**10**	Cell cycle/ meiosis	AT5G20850	RAD51	15
**11**	Cell cycle/ meiosis	AT5G45550	MOB1A	13
**12**	Abiotic stress	AT1G12270	HOP1	12
**13**	Abiotic stress	AT5G64200	SC35	28
**14**	Abiotic stress	AT1G62740	HOP2	12
**15**	Abiotic stress	AT2G41380	----	24
**16**	Abiotic stress	AT2G44150	SDG7	14
**17**	Abiotic stress	AT2G44420	----	----
**18**	Abiotic stress	AT3G44110	DJA3	22
**19**	Abiotic stress	AT5G22060	ATJ2	3
**20**	Abiotic stress / Nutritional stress	AT1G72180	LRR-RLK XI/CEPR2	9, 26
**21**	Abiotic stress /RNA splicing	AT4G02430	SR34B	34
**22**	Abiotic stress /Biotic stress	AT5G52640	HSP83	8
**23**	Abiotic stress /Biotic stress	AT5G56030	HSP90	2
**24**	Biotic stress	AT4G19050	NB-ARC domain	19
**25**	Biotic stress/cell cycle	AT5G45190	Cyclin-like	10
**26**	DNA repair	AT3G50360	CENTRIN2	16
**27**	Nucleoporin	AT3G56900	ALADIN	27
**28**	Cytoskeletal	AT1G04820	TOR2	23
**29**	Cytoskeletal	AT4G14960	TUA6	1
**30**	Cytoskeletal	AT2G36200	BIMC	33
**31**	Metabolic process	AT4G38880	ATASE3	31
**32**	Metabolic process	AT5G08380	AGAL1	5
**33**	Metabolic process/ Embryo development	AT3G02280	TAH18	4
**34**	Development/Germination	AT5G53180	PTB2	21
**35**	Vesicle transport/Vascular tissues	AT5G13300	VAN3/SCF	17, 20
**36**	**----**	AT1G31870	----	----

Table S2 – Annotation Mps1 protein-protein interactions based on available literature.

References - Table S2

1. Abe, T., & Hashimoto, T. (2005). Altered microtubule dynamics by expression of modified α-tubulin protein causes right-handed helical growth in transgenic Arabidopsis plants. Plant Cell, 43, 191-204.

2. Bao, F., Huang, X., Zhu, C., Zhang, X., Li, X., & Yang, S. (2014). Arabidopsis HSP90 protein modulates RPP4-mediated temperature-dependent cell death and defense responses. New Phytologist, 202(4), 1320–1334.

3. Bechtold, U., Richard, O., Zamboni, A., Gapper, C., Geisler, M., Pogson, B., Karpinski, S., & Mullineaux, P. M. (2008). Impact of chloroplastic- and extracellular-sourced ROS on high light-responsive gene expression in Arabidopsis. Journal of Experimental Botany, 59(2), 121–133.

4. Bernard, D. G., Netz, D. J. A., Lagny, T. J., Pierik, a J., & Balk, J. (2013). Requirements of the cytosolic iron-sulfur cluster assembly pathway in Arabidopsis. Philosophical Transactions of the Royal Society B: Biological Sciences, 368(1622), 20120259–20120259.

5. Boudart, G., Jamet, E., Rossignol, M., Lafitte, C., Borderies, G., Jauneau, A., Esquerré-Tugayé, M.-T., & Pont-Lezica, R. (2005). Cell wall proteins in apoplastic fluids of Arabidopsis thaliana rosettes: Identification by mass spectrometry and bioinformatics.

6. Bulankova, P., Riehs-Kearnan, N., Nowack, M. K., Schnittger, A., & Riha, K. (2010). Meiotic Progression in Arabidopsis Is Governed by Complex Regulatory Interactions between SMG7, TDM1, and the Meiosis I-Specific Cyclin TAM. THE PLANT CELL ONLINE, 22(11), 3791–3803.

7. Caillaud, M. C., Paganelli, L., Lecomte, P., Deslandes, L., Quentin, M., Pecrix, Y., le Bris, M., Marfaing, N., Abad, P., & Favery, B. (2009). Spindle assembly checkpoint protein dynamics reveal conserved and unsuspected roles in plant cell division. PLoS ONE, 4(8).

8. Cha, J. Y., Ahn, G., Kim, J. Y., Kang, S. bin, Kim, M. R., Su'udi, M., Kim, W. Y., & Son, D. (2013). Structural and functional differences of cytosolic 90-kDa heat-shock proteins (Hsp90s) in Arabidopsis thaliana. Plant Physiology and Biochemistry, 70, 368–373.

9. ten Hove, C. A., Bochdanovits, Z., Jansweijer, V. M. A., Koning, F. G., Berke, L., Sanchez-Perez, G. F., Scheres, B., & Heidstra, R. (2011). Probing the roles of LRR RLK genes in Arabidopsis thaliana roots using a custom T-DNA insertion set. Plant Molecular Biology, 76(1–2), 69–83.

10. Cui, X., Fan, B., Scholz, J., & Chen, Z. (2007). Roles of Arabidopsis cyclin-dependent kinase C complexes in cauliflower mosaic virus infection, plant growth, and development. Plant Cell, 19(4), 1388–1402.

11. de Veylder, L., Segers, G., Glab, N., Casteels, P., van Montagu, M., & Inzé, D. (1997). The arabidopsis Cks1At protein binds the cyclin-dependent kinases Cdc2aAt and Cdc2bAt. FEBS Letters, 412(3), 446–452.

12. Fellerer, C., Schweiger, R., Schöngruber, K., Soll, J., & Schwenkert, S. (2011). Cytosolic HSP90 Cochaperones HOP and FKBP Interact with Freshly Synthesized Chloroplast Preproteins of Arabidopsis. Molecular Plant, 4(6), 1133–1145.

13. Galla, G., Zenoni, S., Marconi, G., Marino, G., Botton, A., Pinosa, F., Citterio, S., Ruperti, B., Palme, K., Albertini, E., Pezzotti, M., Mau, M., Sharbel, T. F., de Storme, N., Geelen, D., & Barcaccia, G. (2011). Sporophytic and gametophytic functions of the cell cycle-associated Mob1 gene in Arabidopsis thaliana L. Gene, 484(1–2), 1–12.

14. Lee, J., Yun, J.-Y., Zhao, W., Shen, W.-H., & Amasino, R. M. (2015). A methyltransferase required for proper timing of the vernalization response in Arabidopsis. Proceedings of the National Academy of Sciences, 112(7), 2269–2274.

15. Li, W., Chen, C., Markmann-Mulisch, U., Timofejeva, L., Schmelzer, E., Ma, H., & Reiss, B. (2004). The Arabidopsis AtRAD51 gene is dispensable for vegetative development but required for meiosis. Proceedings of the National Academy of Sciences, 101(29), 10596–10601.

16. Liang, L., Flury, S., Kalck, V., Hohn, B., & Molinier, J. (2006). CENTRIN2 Interacts with the Arabidopsis Homolog of the Human XPC Protein (AtRAD4) and Contributes to Efficient Synthesis-dependent Repair of Bulky DNA Lesions. Plant Molecular Biology, 61(1–2), 345–356.

17. Koizumi, K., Naramoto, S., Sawa, S., Yahara, N., Ueda, T., Nakano, A., Sugiyama, M., & Fukuda, H. (2005). VAN3 ARF-GAP-mediated vesicle transport is involved in leaf vascular network formation. Development, 132(7), 1699–1711.

18. Marrocco, K., Thomann, A., Parmentier, Y., Genschik, P., & Criqui, M. C. (2009). The APC/C E3 ligase remains active in most post-mitotic Arabidopsis cells and is required for proper vasculature development and organization. Development, 136(9), 1475–1485.

19. Meyers, B. C., Kozik, A., Griego, A., Kuang, H., & Michelmore, R. W. (2003). Genome-Wide Analysis of NBS-LRR–Encoding Genes in Arabidopsis[W]. The Plant Cell, 15(4), 809–834.

20. Naramoto, S., Sawa, S., Koizumi, K., Uemura, T., Ueda, T., Friml, J., Nakano, A., & Fukuda, H. (2009). Phosphoinositide-dependent regulation of VAN3 ARF-GAP localization and activity essential for vascular tissue continuity in plants. Development, 136(9), 1529–1538.

21. Ruhl, C., Stauffer, E., Kahles, A., Wagner, G., Drechsel, G., Ratsch, G., & Wachter, A. (2012). Polypyrimidine Tract Binding Protein Homologs from Arabidopsis Are Key Regulators of Alternative Splicing with Implications in Fundamental Developmental Processes. The Plant Cell, 24(11), 4360–4375.

22. Salas-Muñoz, S., Rodríguez-Hernández, A. A., Ortega-Amaro, M. A., Salazar-Badillo, F. B., & Jiménez-Bremont, J. F. (2016). Arabidopsis AtDjA3 Null Mutant Shows Increased Sensitivity to Abscisic Acid, Salt, and Osmotic Stress in Germination and Post-germination Stages. Frontiers in Plant Science, 7(February), 1–11.

23. Sambade, A., Findlay, K., Schaffner, A. R., Lloyd, C. W., & Buschmann, H. (2014). Actin-Dependent and -Independent Functions of Cortical Microtubules in the Differentiation of Arabidopsis Leaf Trichomes. The Plant Cell, 26(4), 1629–1644.

24. Sarry, J., Kuhn, L., Ducruix, C., Lafaye, A., Junot, C., Hugouvieux, V., Jourdain, A., Bastien, O., Fievet, J. B., Vailhen, D., Amekraz, B., Moulin, C., Ezan, E., Garin, J., & Bourguignon, J. (2006). The early responses of Arabidopsis thaliana cells to cadmium exposure explored by protein and metabolite profiling analyses. PROTEOMICS, 6(7), 2180–2198.

25. Serralbo, O., Perez-Perez, J. M., Heidstra, R., & Scheres, B. (2006). Non-cell-autonomous rescue of anaphase-promoting complex function revealed by mosaic analysis of HOBBIT, an Arabidopsis CDC27 homolog. Proceedings of the National Academy of Sciences, 103(35), 13250–13255.

26. Tabata, R., Sumida, K., Yoshii, T., Ohyama, K., Shinohara, H., & Matsubayashi, Y. (2014). Perception of root-derived peptides by shoot LRR-RKs mediates systemic N-demand signaling. Science, 346(6207), 343–346.

27. Tamura, K., Fukao, Y., Iwamoto, M., Haraguchi, T., & Hara-Nishimura, I. (2010). Identification and Characterization of Nuclear Pore Complex Components in Arabidopsis thaliana. The Plant Cell, 22(12), 4084–4097

28. Tanabe, N., Yoshimura, K., Kimura, A., Yabuta, Y., & Shigeoka, S. (2007). Differential Expression of Alternatively Spliced mRNAs of Arabidopsis SR Protein Homologs, atSR30 and atSR45a, in Response to Environmental Stress. Plant and Cell Physiology, 48(7), 1036–1049.

29. van Damme, D., de Rybel, B., Gudesblat, G., Demidov, D., Grunewald, W., de Smet, I., Houben, A., Beeckman, T., & Russinova, E. (2011). Arabidopsis α Aurora Kinases Function in Formative Cell Division Plane Orientation. The Plant Cell, 23(11), 4013–4024.

30. van Leene, J., Hollunder, J., Eeckhout, D., Persiau, G., van de Slijke, E., Stals, H., van Isterdael, G., Verkest, A., Neirynck, S., Buffel, Y., de Bodt, S., Maere, S., Laukens, K., Pharazyn, A., Ferreira, P. C. G., Eloy, N., Renne, C., Meyer, C., Faure, J.-D., … de Jaeger, G. (2010). Targeted interactomics reveals a complex core cell cycle machinery in Arabidopsis thaliana. Molecular Systems Biology, 6(397), 397.

31. Hung, W.-F., Chen, L.-J., Boldt, R., Sun, C.-W., & Li, H. (2004). Characterization of Arabidopsis Glutamine Phosphoribosyl Pyrophosphate Amidotransferase-Deficient Mutants. Plant Physiology, 135(3), 1314–1323.

32. Yang, K., Wang, H., Xue, S., Qu, X., Zou, J., & Le, J. (2014). Requirement for A-type cyclin-dependent kinase and cyclins for the terminal division in the stomatal lineage of Arabidopsis. Journal of Experimental Botany, 65(9), 2449–2461.

33. Lee, Y. R. J., & Liu, B. (2004). Cytoskeletal motors in Arabidopsis. Sixty-one kinesins and seventeen myosins. In Plant Physiology (Vol. 136, Issue 4, pp. 3877–3883). American Society of Plant Biologists.

34. Zhang, W., Du, B., Liu, D., & Qi, X. (2014). Splicing factor SR34b mutation reduces cadmium tolerance in Arabidopsis by regulating iron-regulated transporter 1 gene. Biochemical and Biophysical Research Communications, 455(3–4), 312–317.

Domain	Residue Position	Residue Substrate	Phosohorylation prediction			Kinase prediction		
			PlantPhos	DISPHOS	NetSurfP	KinasePhos 2.0	GPS Polo	Reference
N-terminal	27	T ●	†	•	E	GRK/PKB	•	
	28	T ●	†	•	E	CDK/PKC/GSK3	†	1
	29	T ●	†	†	E	•	•	6
	31	S ●	†	†	E	AKT1/PKG	•	1, 9
	62	S/T ●	•	•	E	•	•	1
	68	S/T ●	†	†	E	PKA/PKG	•	1
	125	T/S ●	†	•	E	•	•	13
	136	S/T ●	†	•	E	ATM	•	1
	167	S/T ●	•	•	E	ATM	•	1
	175	T/S ●	•	•	E	•	•	1
	210	S/T ●	†	†	E	ATM/CK1/Plk1	†	1, 3, 4, 16, 17
	232	S/T	†	•	E	GRK/PKB	•	---
	235	S		•	E	PKC/PKA/PKG	•	2
	239	S/T	†	•	E	ATM/ Aurora/Plk1	•	2
	243	T	†	•	E	CK2/PDK	•	----
	249	S ●	•	•	E	Cdc2/ATM	•	1
	250	S ●	†	•	B	ATM/ Plk1/GSK3	•	1, 3, 16, 19
	251	S ●	†	•	E	ATM/ PKB/GSK3	•	1, 3, 4, 6, 7, 16
	259	T	•	•	E	GRK/PKB	•	3, 8, 16, 17,19
	262	S/Y	†	†	E	ATM/CK1	•	---
	270	S	†	•	E	Plk1/ATM/GSK3	•	3, 8
	303	T/S ●	•	•	E	ATM	†	1
	310	S	†	†	E	ATM/Plk1/MAPK/CDK	•	----
	324	S ●	•	•	E	CKII/CKI	†	1
	330 ●	T/S	†	•	E	CK2/PDK/ GRK/PKB	•	3, 8
	339	S/T ●	†	†	E	ATM/ Aurora	•	7, 13
	359	S	†	†	E	ATM/ GSK3/Aurora/Plk1	†	---
	387	Y	†	•	E	FGFR1/AK2/TK1	•	---
	400	Y	†	•	B	FGFR1/JAK2/TK1	•	---
Kinase Domain	408	S	†	•	E	ATM/GSK3/PKB1/Plk1	•	---
	411	S	†	†	E	ATM/Aurora/Plk1	•	---
	412	S	†	•	B	ATM/Aurora/CDK/Plk1	•	---
	425	Y	†	•	B	PDGFR/ZAP70/FGFR1	•	---
	439 ●	T ●	•	•	E	GRK/PKB	•	3, 12, 15, 16
	449	Y	†	•	B	FGFR1/PDGFR/ZAP70	•	---
	465	Y	†	•	B	FGFR1/PDGFR/ZAP70	•	---
	468 ●	T	†	•	E	GRK/PKB	•	16
	495	Y	†	•	B	FGFR1/PDGFR/ZAP70	•	---
	522	T	†	•	E	CK2/PDK	†	---
	531	Y	•	•	B	Ret/FGFR1/PDGFR/ZAP70	•	---
	541	T	•	•	B	CK2/PDK/ GRK/PKB	•	---
	550	S	•	•	B	ATM/Aurora/PKC/PKB	•	---
	579 ●	T ●	•	•	E	GRK/PKB	•	1, 12,15, 16
	580 ●	T ●●	†	•	E	CDK/ PKC/PKB	†	7, 8, 13, 15,18

Table S3 – AtMps1 phosphorylated residues and protein kinases prediction.

Labels – green: conserved and aligned residues in other plant species (monocots, dicots or both); red: experimentally confirmed residues in hMps1; ● autophosphorylation sites in hMps1; ● phosphorylation sites in Xenopus laevis; ● phosphorylation sites in yeast; ●no prediction in the selected tool; † positive prediction in the selected tool. Sequences utilized: *Populus trichocarpa* (gi|224063138); *Ricinus communis*: (gi|255545510); *Vitis vinifera* (gi|225459469); *Oryza sativa* (gi|22450594 and gi|125545426); *Sorghum bicolor* (Sb01g010730.1); *Zea mays* (GRMZM2G143160_T01); *Medicago truncatula* (Medtr3g098080 and Medtr5g065090.1); *Phaseolus vulgaris* (Phvulv091012681m); *Carica papaya* (evm.model.supercontig_36.11); *Citrus cinensis* (orange1.1g003052m); *Eucalyptus grandis* (Eucgr.F03751.1); *Manihot esculenta* (assava4.1_001761m); *Glycine Max* (Glyma14g33400.1, Glyma14g33400.2, Glyma06g11500.3, Glyma06g11500.4, Glyma04g43190.1 and Glyma13g02620.1)

References Table S3

1. Breitkreutz, A., Choi, H., Sharom, J. R., Boucher, L., Neduva, V., Larsen, B., Lin, Z. Y., Breitkreutz, B. J., Stark, C., Liu, G., Ahn, J., Dewar-Darch, D., Reguly, T., Tang, X., Almeida, R., Qin, Z. S., Pawson, T., Gingras, A. C., Nesvizhskii, A. I., & Tyers, M. (2010). A Global Protein Kinase and Phosphatase Interaction Network in Yeast. Science, 328(5981), 1043–1046.

2. Daub, H., Olsen, J. v, Bairlein, M., Gnad, F., Oppermann, F. S., Körner, R., Greff, Z., Kéri, G., Stemmann, O., & Mann, M. (2008). Kinase-selective enrichment enables quantitative phosphoproteomics of the kinome across the cell cycle. Molecular Cell, 31(3), 438–448.

3. Dou, Z., von Schubert, C., Körner, R., Santamaria, A., Elowe, S., & Nigg, E. a. (2011). Quantitative mass spectrometry analysis reveals similar substrate consensus motif for human Mps1 kinase and Plk1. PLoS ONE, 6(4).

4. Dulla, K., Daub, H., Hornberger, R., Nigg, E. a, & Korner, R. (2010). Quantitative Site-specific Phosphorylation Dynamics of Human Protein Kinases during Mitotic Progression. Molecular & Cellular Proteomics, 9(6), 1167–1181.

5. Iakoucheva, L. M., Radivojac, P., Brown, C. J., O'Connor, T. R., Sikes, J. G., Obradovic, Z., & Dunker, K. A. (2004). The importance of intrinsic disorder for protein phosphorylation. Nucleic Acids Research, 32(3), 1037–1049.

6. Jaspersen, S. L., Huneycutt, B. J., Giddings, T. H., Resing, K. a., Ahn, N. G., & Winey, M. (2004). Cdc28/Cdk1 Regulates Spindle Pole Body Duplication through Phosphorylation of Spc42 and Mps1. Developmental Cell, 7(2), 263–274.

7. Jelluma, N., Brenkman, A. B., McLeod, I., Yates, J. R., Cleveland, D. W., Medema, R. H., & Kops, G. J. P. L. (2008). Chromosomal Instability by Inefficient Mps1 Auto-Activation Due to a Weakened Mitotic Checkpoint and Lagging Chromosomes. PLoS ONE, 3(6), e2415.

8. Kang, J., Chen, Y., Zhao, Y., & Yu, H. (2007). Autophosphorylation-dependent activation of human Mps1 is required for the spindle checkpoint. Proc. Natl. Acad. Sci. U S A, 104, 20232-7.

9. Keck, J. M., Jones, M. H., Wong, C. C. L., Binkley, J., Chen, D., Jaspersen, S. L., Holinger, E. P., Xu, T., Niepel, M., Rout, M. P., Vogel, J., Sidow, A., Yates, J. R., & Winey, M. (2011). A Cell Cycle Phosphoproteome of the Yeast Centrosome. Science, 332(6037), 1557–1561.

10. Lee, T. Y., Bretaña, N. A., & Lu, C. T. (2011). PlantPhos: using maximal dependence decomposition to identify plant phosphorylation sites with substrate site specificity. BMC bioinformatics, 12.

11. Liu, Z., Ren, J., Cao, J., He, J., Yao, X., Jin, C., & Xue, Y. (2013). Systematic analysis of the Plk-mediated phosphoregulation in eukaryotes. Briefings in Bioinformatics, 14(3), 344–360

12. Mattison, C. P., Old, W. M., Steiner, E., Huneycutt, B. J., Resing, K. A., Ahn, N. G., & Winey, M. (2007). Mps1 Activation Loop Autophosphorylation Enhances Kinase Activity. Journal of Biological Chemistry, 282(42), 30553–30561

13. Morin, V., Prieto, S., Melines, S., Hem, S., Rossignol, M., Lorca, T., Espeut, J., Morin, N., & Abrieu, A. (2012). CDK-Dependent Potentiation of MPS1 Kinase Activity Is Essential to the Mitotic Checkpoint. Current Biology, 22(4), 289–295.

14. Petersen, B., Petersen, T. N., Andersen, P., Nielsen, M., & Lundegaard, C. (2009). A generic method for assignment of reliability scores applied to solvent accessibility predictions. BMC Struct Biol 9.

15. Tyler, R. K., Chu, M. L. H., Johnson, H., McKenzie, E. A., Gaskell, S. J., & Eyers, P. A. (2009). Phosphoregulation of human Mps1 kinase. Biochemical Journal, 417(1), 173–184.

16. von Schubert, C., Cubizolles, F., Bracher, J. M., Sliedrecht, T., Kops, G. J. P. L., & Nigg, E. A. (2015). Plk1 and Mps1 Cooperatively Regulate the Spindle Assembly Checkpoint in Human Cells. Cell Reports, 12(1), 66–78.

17. Wang, X., Yu, H., Xu, L., Zhu, T., Zheng, F., Fu, C., Wang, Z., & Dou, Z. (2014). Dynamic Autophosphorylation of Mps1 Kinase Is Required for Faithful Mitotic Progression. PLoS ONE, 9(9), e104723.

18. Wang, W., Yang, Y., Gao, Y., Xu, Q., Wang, F., Zhu, S., Old, W., Resing, K., Ahn, N., Lei, M., & Liu, X. (2009). Structural and mechanistic insights into Mps1 kinase activation. Journal of Cellular and Molecular Medicine, 13(8b), 1679–1694.

19. Xu, Q., Zhu, S., Wang, W., Zhang, X., Old, W., Ahn, N., & Liu, X. (2009). Regulation of Kinetochore Recruitment of Two Essential Mitotic Spindle Checkpoint Proteins by Mps1 Phosphorylation. Molecular Biology of the Cell, 20(1), 10–20.

20. Wong, Y.-H., Lee, T.-Y., Liang, H.-K., Huang, C.-M., Wang, T.-Y., Yang, Y.-H., Chu, C.-H., Huang, H.-D., Ko, M.-T., & Hwang, J.-K. (2007). KinasePhos 2.0: a web server for identifying protein kinase-specific phosphorylation sites based on sequences and coupling patterns. Nucleic Acids Research, 35(suppl_2), W588–W594.

Table S4 - Protein detection and annotation derived from the proteomics of inhibition experiment. The table also indicates protein fold change for each sample treatment.

Accession	Peptide count	Unique peptides	Confidence score	Anova (p)	(-)LOG10_ANOVA	Max fold change	Highest mean condition	Lowest mean condition
AT1G0524(	16	11	170,0471	1,87E-05	-4,7274	9,206043	DMSO	Control
AT5G2630(	5	1	40,5897	3,25E-05	-4,48795	3,747844	Control	1mg
AT1G2536(	2	1	23,3624	5,67E-05	-4,2464	40,57327	1mg	DMSO
AT5G1921(	4	1	27,3639	5,80E-05	-4,23645	23,65429	Control	1mg
AT1G5113(	1	1	6,1817	6,64E-05	-4,17763	2,125089	DMSO	Control
AT3G5801(	2	1	21,0796	6,94E-05	-4,15887	4,763374	DMSO	1mg
AT3G1678(	1	1	5,9294	7,11E-05	-4,14812	Infinity	DMSO	Control
AT4G2607(	1	1	5,0967	7,90E-05	-4,10234	Infinity	DMSO	1mg
AT3G0562(	2	1	21,4143	0,000123011	-3,91006	40,64345	Control	1mg
AT5G1530(	1	1	5,0218	0,000140866	-3,85119	6,973895	Control	1mg
AT1G0278(	3	3	32,9241	0,00024331	-3,61384	28,92596	DMSO	Control
AT3G4996(	4	2	27,7861	0,000261104	-3,58319	4,081642	DMSO	1mg
AT3G5327(	2	1	11,5997	0,00031262	-3,50498	18,69206	DMSO	1mg
AT3G1624(	3	2	26,3997	0,00034257	-3,46525	2,627417	DMSO	1mg
AT3G1349(	3	1	16,7144	0,000378133	-3,42236	3,094625	1mg	DMSO
AT5G2398(	4	2	22,0453	0,000440977	-3,35558	2,624188	DMSO	Control
AT1G8024(	5	4	28,2843	0,000479004	-3,31966	1,78162	DMSO	1mg
AT2G4259(	5	3	48,5815	0,000534669	-3,27192	1,525744	DMSO	1mg
AT3G1071(	3	1	17,2842	0,000563881	-3,24881	1,268482	1mg	Control
AT4G0227(	3	3	18,9467	0,000573719	-3,2413	5,860905	DMSO	1mg
AT3G6268(	5	5	41,2983	0,000581132	-3,23573	2,618912	DMSO	1mg
AT2G2363(	10	3	64,2835	0,000608984	-3,21539	2,075946	Control	1mg
AT5G4981(	20	11	121,0619	0,000634758	-3,19739	1,86519	Control	1mg
AT2G3807(	4	1	26,152	0,000652215	-3,18561	1,863101	Control	1mg
AT1G0820(	16	5	111,0121	0,000664755	-3,17734	2,013143	Control	1mg
AT3G1976(	18	13	139,9403	0,000815492	-3,08858	1,434354	DMSO	1mg
AT1G1786(	4	4	27,0385	0,000840476	-3,07547	15,33402	DMSO	1mg
AT3G0191(	4	2	27,6294	0,000926607	-3,0331	1,312809	1mg	Control
AT1G7073(	21	6	155,7595	0,000990829	-3,004	1,654376	Control	1mg
AT1G2829(	6	4	78,2912	0,001019026	-2,99181	1,821612	DMSO	Control
AT4G1400(	3	2	16,1442	0,00108189	-2,96582	4,769418	1mg	Control
AT3G4856(	6	3	32,8004	0,001260692	-2,89939	1,2587	Control	1mg
AT5G2190(	2	2	15,5065	0,001269644	-2,89632	5,318612	DMSO	1mg
AT1G7992(	33	1	264,323	0,001270104	-2,89616	1,739474	DMSO	Control
AT1G2113(	3	2	15,5752	0,001408217	-2,85133	1,986028	DMSO	1mg
AT4G2847(	20	4	130,2121	0,001429263	-2,84489	1,984247	Control	1mg
AT5G6444(	2	1	10,875	0,001461773	-2,83512	22,01114	DMSO	1mg
AT1G2855(	7	3	42,306	0,001507179	-2,82184	1,807061	1mg	Control

AT2G2045(	2	1	11,2454	0,001568497	-2,80452	5,67997	DMSO	Control
AT2G0770	3	3	22,5191	0,001569481	-2,80424	1,484891	DMSO	1mg
AT1G6743(	2	2	13,3496	0,001583194	-2,80047	3,844049	DMSO	Control
AT3G5146(	3	3	17,2091	0,001607668	-2,7938	1,95018	DMSO	1mg
AT4G1739(	4	3	52,646	0,00170318	-2,76874	6,371912	DMSO	Control
AT4G2670(	2	1	10,8589	0,001868096	-2,7286	1,387679	1mg	Control
AT4G1750	1	1	5,3307	0,001921427	-2,71638	18,10225	Control	1mg
AT1G6962(	2	2	12,3002	0,001922862	-2,71605	2,195418	DMSO	1mg
AT1G1205(	2	2	10,7215	0,001952695	-2,70937	4,296948	DMSO	1mg
AT2G2501(	1	1	11,1596	0,002105535	-2,67664	1,418063	1mg	Control
AT2G3167(	4	3	23,6009	0,002155783	-2,66639	19,45949	Control	1mg
AT1G5207(	10	6	64,297	0,002161318	-2,66528	1,270532	DMSO	1mg
AT1G6984(	6	4	44,8702	0,002222961	-2,65307	1,422936	1mg	Control
AT4G3337(	2	1	10,327	0,002233001	-2,65111	2,505843	Control	1mg
AT1G0269(	2	1	12,2139	0,002281712	-2,64174	60,86827	1mg	Control
AT1G1774	8	3	76,9165	0,002282577	-2,64157	1,382448	Control	1mg
AT5G4487(	3	2	16,6751	0,002345793	-2,62971	4,49318	DMSO	1mg
AT5G2764(	14	10	94,236	0,002353881	-2,62822	1,561088	Control	1mg
AT2G4461(	3	1	18,9719	0,002367458	-2,62572	1,807275	1mg	Control
AT5G4402(	7	4	56,1525	0,002392552	-2,62114	5,103292	DMSO	Control
AT1G4188(	1	1	6,0159	0,00239327	-2,62101	Infinity	DMSO	Control
AT2G1845(	18	5	130,1198	0,002484534	-2,60476	1,31041	Control	DMSO
AT1G2002(	1	1	5,068	0,002549174	-2,5936	1,98594	1mg	Control
AT4G3305(	3	1	16,0285	0,002617992	-2,58203	2,05157	1mg	Control
AT3G5725(	1	1	5,055	0,0026997	-2,56868	4,83976	DMSO	1mg
AT1G2691(	7	2	56,9226	0,002748456	-2,56091	1,523301	1mg	Control
AT2G3442(	1	1	5,8004	0,002777326	-2,55637	3,932398	1mg	Control
AT3G1387(	17	2	113,5673	0,002812526	-2,5509	9,710114	Control	1mg
AT2G4066(	4	2	27,378	0,002898502	-2,53783	1,847793	DMSO	1mg
AT5G6562(	10	3	61,3127	0,002914273	-2,53547	1,470491	1mg	Control
AT4G3467(	14	8	107,4684	0,003102643	-2,50827	2,116254	DMSO	1mg
AT1G4784(	6	4	34,2132	0,003134684	-2,50381	1,676167	1mg	Control
AT1G1084(	5	3	28,9717	0,003180719	-2,49747	1,851003	Control	1mg
AT5G6112(	2	2	17,2925	0,003265056	-2,48611	3,621469	DMSO	Control
AT4G1626(	18	12	207,2594	0,003319456	-2,47893	3,922286	1mg	Control
AT2G0445(	2	2	10,3606	0,003320054	-2,47885	1,769489	Control	1mg
AT3G1311(	2	2	10,8331	0,003365225	-2,47299	2,398837	Control	1mg
AT3G5512(	4	4	25,1545	0,003420098	-2,46596	2,239851	1mg	Control
AT2G2703(	7	2	59,6421	0,003452199	-2,4619	5,768105	1mg	DMSO
AT2G3839(	11	5	82,3453	0,003454288	-2,46164	2,342713	1mg	Control
AT3G4531(	8	3	50,1618	0,003459787	-2,46095	1,38153	1mg	Control
AT4G3368(	9	5	79,8663	0,003602263	-2,44342	1,722931	DMSO	Control
AT5G3820(	2	2	11,6622	0,003961797	-2,40211	1,223336	1mg	Control
AT4G2089(	25	2	305,4396	0,004119968	-2,38511	1,388439	DMSO	1mg
AT4G3998(	13	6	75,904	0,004237965	-2,37284	1,413436	1mg	Control
AT4G2601(	9	5	63,7436	0,004280764	-2,36848	1,768548	DMSO	Control
AT5G5573(	5	2	32,9123	0,004305378	-2,36599	1,87193	1mg	Control
AT5G0830(	9	2	106,3433	0,004322895	-2,36423	2,233103	1mg	DMSO
AT5G4218(	3	2	18,55	0,004408203	-2,35574	17,43149	Control	1mg
AT1G6419(	21	6	198,5806	0,00457607	-2,33951	1,198508	Control	DMSO

AT1G4885(	15	10	106,2137	0,004589045	-2,33828	1,266173	DMSO	Control
AT1G5410(	12	7	72,7895	0,004601745	-2,33708	1,301252	DMSO	1mg
AT1G7712(	10	8	67,4476	0,004633521	-2,33409	2,16606	1mg	Control
AT5G1733(	26	14	246,1403	0,00473846	-2,32436	1,773784	DMSO	1mg
AT3G4530(	6	5	42,0495	0,004813871	-2,31751	2,22586	Control	1mg
AT5G4854(	1	1	5,3331	0,00482261	-2,31672	1,68514	Control	1mg
AT2G2042(	18	14	162,6344	0,005237254	-2,2809	1,370171	DMSO	1mg
AT3G1882(	8	2	60,2355	0,005275061	-2,27777	4,149044	Control	1mg
AT5G6036(	10	3	79,2092	0,005339693	-2,27248	2,44068	Control	1mg
AT1G6802(	11	3	65,3687	0,005408298	-2,26694	1,474576	Control	1mg
AT5G2029(	8	4	75,1635	0,005449275	-2,26366	2,893279	DMSO	1mg
AT2G1948(	7	3	40,9864	0,00566243	-2,247	1,717527	Control	1mg
AT1G1827(	32	22	209,7258	0,006099229	-2,21473	1,247882	Control	1mg
AT4G1810(	2	2	18,8529	0,006483527	-2,18819	6,828305	DMSO	Control
AT3G1422(	4	3	34,0956	0,006517704	-2,18591	2,049707	DMSO	Control
AT3G4810(	1	1	5,6913	0,00679505	-2,16781	1,445411	Control	DMSO
AT1G5512(	3	1	18,6278	0,006990262	-2,15551	1,780994	Control	1mg
AT3G5326(	10	3	69,6297	0,007009412	-2,15432	4,145967	DMSO	1mg
AT1G6280(	8	3	47,6753	0,007054378	-2,15154	1,661368	1mg	Control
AT4G3908(	14	6	88,2644	0,007150674	-2,14565	1,656602	DMSO	1mg
AT5G0414(	14	2	96,8164	0,007186935	-2,14346	1,710798	Control	1mg
AT2G2955(	26	4	287,0462	0,007194283	-2,14301	1,302969	DMSO	1mg
AT5G6692(	5	2	28,1271	0,007224516	-2,14119	3,246658	1mg	Control
AT1G6626(	1	1	6,3117	0,007240116	-2,14025	1,664089	DMSO	Control
AT2G0126(	1	1	5,5547	0,007446297	-2,12806	2,504159	Control	1mg
AT5G1461(	2	1	12,2302	0,007501273	-2,12486	10,74497	DMSO	1mg
AT3G1509(	3	3	15,776	0,007760269	-2,11012	2,700803	Control	1mg
AT1G7886(	21	5	187,4552	0,007762489	-2,11	1,963186	Control	1mg
AT5G0997ℇ	2	2	12,7397	0,007801065	-2,10785	1,431843	1mg	DMSO
AT2G2398(	2	2	10,4838	0,008066165	-2,09333	1,485103	DMSO	1mg
AT3G0890(	13	4	124,0725	0,008106697	-2,09116	1,910667	1mg	DMSO
AT2G4145:	1	1	5,6163	0,008235032	-2,08433	1,472212	1mg	Control
AT5G5410(	2	2	10,1911	0,008306169	-2,0806	2,262811	DMSO	1mg
AT1G7563(	2	2	22,386	0,008348161	-2,07841	1,734653	DMSO	1mg
AT3G0739(	5	4	29,471	0,008394202	-2,07602	1,432789	1mg	Control
AT2G4217(	9	5	69,0569	0,00852525	-2,06929	1,922252	Control	1mg
AT3G1312(	1	1	5,8146	0,008845912	-2,05326	12,74903	Control	1mg
AT2G3977(	7	5	51,9988	0,008852466	-2,05294	1,509653	DMSO	1mg
AT5G4133(	2	1	11,1693	0,009032173	-2,04421	6,464974	DMSO	1mg
AT3G5296(	9	7	65,4654	0,009230611	-2,03477	1,212292	Control	DMSO
AT3G4565(	2	1	15,8585	0,009351419	-2,02912	1,995801	1mg	Control
AT4G1777(	5	2	26,859	0,009532564	-2,02079	1,826701	1mg	Control
AT2G2058(	31	13	231,4065	0,009613955	-2,0171	1,516482	DMSO	1mg
AT5G4121(	1	1	5,2663	0,009655102	-2,01524	2,591796	Control	1mg
AT5G5924(	5	1	40,4467	0,009780934	-2,00962	2,935786	DMSO	1mg
AT2G2225(	8	6	52,6057	0,009818964	-2,00793	1,449637	Control	1mg
AT4G2840(	3	1	18,3405	0,010068717	-1,99703	2,23378	1mg	Control
AT1G5236(	20	6	124,0711	0,010110088	-1,99525	1,943458	Control	1mg
AT1G6597(	4	2	49,0451	0,010178919	-1,9923	2,493857	Control	1mg
AT2G1336(	1	1	5,6998	0,010245777	-1,98946	1,411617	Control	DMSO

AT3G0894(	8	3	43,8037	0,010254535	-1,98908	8,134704	Control	1mg
AT1G7326(	9	8	87,7496	0,010264252	-1,98867	2,021164	1mg	DMSO
AT1G1635(	8	6	53,0949	0,01054349	-1,97702	1,320658	Control	1mg
AT3G2702(	1	1	5,771	0,010848543	-1,96463	1,258362	1mg	Control
AT5G3941(	5	4	40,879	0,011046742	-1,95677	2,694608	Control	1mg
AT3G1258(	26	3	314,1415	0,011136721	-1,95324	2,225575	Control	1mg
AT1G2648(	8	4	59,3134	0,01137236	-1,94415	1,856323	DMSO	1mg
AT5G1459(	18	10	156,2039	0,011754381	-1,9298	1,415489	Control	1mg
AT3G4793(	2	1	10,3111	0,011863311	-1,92579	2,155223	1mg	Control
AT2G4410(	18	9	138,1634	0,011980832	-1,92151	2,630166	Control	1mg
AT1G0256(	4	3	21,7347	0,012040345	-1,91936	1,311558	1mg	DMSO
AT2G2772(	6	3	61,5215	0,012137021	-1,91589	1,802906	1mg	Control
AT5G4333(	19	5	238,939	0,012789801	-1,89314	1,905151	Control	1mg
AT3G1439(	17	3	125,3486	0,013119234	-1,88209	3,389975	DMSO	Control
AT3G0871(	1	1	5,6065	0,013154184	-1,88094	2,154644	1mg	DMSO
AT5G6740(	8	5	55,6488	0,013311257	-1,87578	2,914546	DMSO	Control
ATCG0049(	31	16	301,7287	0,013585262	-1,86693	2,607816	1mg	Control
AT3G0119(	7	3	43,4286	0,013842693	-1,85878	4,326937	DMSO	Control
AT5G4393(	3	2	16,5242	0,013932134	-1,85598	1,917923	1mg	Control
AT4G1278(	3	1	22,1247	0,013966429	-1,85491	2,076849	1mg	Control
AT1G2241(	16	9	101,2051	0,014025914	-1,85307	1,232633	DMSO	1mg
AT3G1034(	5	4	27,1845	0,014218701	-1,84714	2,149275	Control	1mg
AT5G6615(	3	1	24,7953	0,014221	-1,84707	2,209676	Control	1mg
AT4G2912(	6	1	48,7689	0,014265904	-1,8457	1,702326	Control	DMSO
AT3G6077(	1	1	5,5292	0,01444013	-1,84043	2,274469	DMSO	Control
AT4G3909(	4	2	37,6561	0,014515565	-1,83817	1,625638	DMSO	1mg
AT1G2657(	14	4	114,6571	0,01473395	-1,83168	2,940467	1mg	DMSO
AT5G1840(	4	3	37,7485	0,014834658	-1,82872	1,5853	DMSO	1mg
AT5G1923(	1	1	10,7366	0,014841399	-1,82853	2,042425	Control	1mg
AT1G2988(	18	10	103,0508	0,015087636	-1,82138	1,66207	DMSO	Control
AT5G2626(	18	9	195,6943	0,015297832	-1,81537	1,184671	1mg	Control
AT5G5269(	1	1	6,1204	0,015475253	-1,81036	3,882225	DMSO	1mg
AT4G1343(	19	16	134,4454	0,01557234	-1,80765	1,248319	DMSO	Control
AT1G1483(	4	2	21,8281	0,015629603	-1,80605	1,944222	Control	1mg
AT1G4183(	6	2	50,8818	0,015886809	-1,79896	1,690802	DMSO	Control
AT3G4887(	19	4	104,2506	0,016003107	-1,7958	1,385594	Control	1mg
AT2G1802(	7	1	85,6556	0,016455731	-1,78368	2,994361	DMSO	1mg
AT3G1781(	15	11	124,9411	0,016666195	-1,77816	1,507687	DMSO	1mg
AT3G0858(	31	8	359,8977	0,016898481	-1,77215	1,68319	DMSO	1mg
AT3G1030(	2	1	10,3618	0,016983293	-1,76998	19,37286	Control	1mg
AT1G7969(	3	3	22,1028	0,017057903	-1,76807	2,769528	Control	1mg
AT3G1154(	1	1	5,7608	0,017370611	-1,76018	1,547712	1mg	Control
AT3G2360(	4	1	40,817	0,017406804	-1,75928	2,357258	1mg	Control
AT2G4252(	14	3	92,9167	0,01780798	-1,74939	1,825498	DMSO	Control
AT1G0550(	3	2	17,2213	0,017826966	-1,74892	1,763018	1mg	DMSO
AT5G4232(	5	2	33,7009	0,018012493	-1,74443	1,672699	1mg	Control
AT4G1048(	5	2	44,2661	0,0181264	-1,74169	1,297135	Control	1mg
AT3G2399(	39	17	370,4402	0,018571184	-1,73116	1,214097	DMSO	Control
AT4G2085(	34	22	229,5583	0,018627289	-1,72985	1,314015	Control	1mg
AT2G1475(	1	1	5,547	0,018650171	-1,72932	1,545714	Control	1mg

AT1G7083(	6	1	40,5519	0,018708891	-1,72795	4,119155	DMSO	1mg
AT3G0859(	28	11	253,9393	0,018777196	-1,72637	1,324321	DMSO	Control
AT3G4899(	19	15	163,8136	0,018994205	-1,72138	1,183686	Control	DMSO
AT3G1494(	40	10	304,1341	0,019144444	-1,71796	1,705265	Control	1mg
AT4G2267(	10	7	61,8569	0,019437349	-1,71136	1,444825	Control	1mg
AT5G0443(	9	6	62,4344	0,019450899	-1,71106	1,221341	Control	1mg
AT3G0460(	3	1	16,2713	0,019468335	-1,71067	Infinity	DMSO	1mg
AT3G0571(	2	2	17,9513	0,019480757	-1,71039	1,825648	1mg	Control
AT2G0592(	15	11	114,3963	0,0195882	-1,70801	1,418565	DMSO	Control
AT5G5183(	9	4	70,0676	0,019770114	-1,70399	1,733194	Control	1mg
AT4G3325(	6	4	47,74	0,019840359	-1,70245	1,643886	DMSO	1mg
AT1G6856(	18	17	108,354	0,020026752	-1,69839	1,788138	DMSO	1mg
AT1G1184(	14	9	124,8193	0,020047724	-1,69793	1,446	DMSO	1mg
AT5G6543(	20	6	155,7927	0,020166263	-1,69537	2,265009	Control	1mg
AT2G2452(	14	6	99,4996	0,020497277	-1,6883	1,225407	Control	1mg
AT4G2745(	5	2	29,1273	0,020954219	-1,67873	2,774887	Control	1mg
AT5G5650(	21	6	150,0807	0,02098618	-1,67807	1,466891	1mg	Control
AT1G6420(	5	3	30,5866	0,021047192	-1,67681	3,208261	1mg	Control
AT5G1120(	18	1	160,0851	0,021121299	-1,67528	2,663304	DMSO	Control
AT3G0968(	3	2	21,4527	0,02114344	-1,67482	3,673084	DMSO	Control
AT2G0705(	2	1	11,4296	0,021304882	-1,67152	1,849151	1mg	Control
AT3G2417(	15	12	107,0544	0,021358507	-1,67043	1,33106	Control	1mg
AT1G4523:	4	2	20,5038	0,021636643	-1,66481	1,439847	Control	DMSO
AT1G5434(	17	8	171,7776	0,022681559	-1,64433	3,056466	Control	1mg
AT1G6068(	9	2	52,6969	0,022793036	-1,6422	2,341844	DMSO	1mg
AT3G4410(	2	1	13,8889	0,022879481	-1,64055	1,363693	Control	DMSO
AT4G2149(	1	1	5,2567	0,023042469	-1,63747	2,255126	1mg	DMSO
AT5G3332(	4	4	22,8532	0,023103861	-1,63632	1,436917	Control	1mg
AT4G2630(	9	4	57,0386	0,023411481	-1,63057	1,627061	DMSO	1mg
AT3G1429(	5	1	49,7387	0,023537993	-1,62823	3,188829	1mg	DMSO
AT5G0709(	9	7	66,2427	0,023580437	-1,62745	2,299	DMSO	Control
AT5G5663(	5	2	28,5782	0,023584325	-1,62738	1,638511	DMSO	1mg
AT4G0404(	12	2	73,8545	0,023805582	-1,62332	5,12848	Control	DMSO
AT1G0130(	1	1	5,9325	0,023868723	-1,62217	3,610691	DMSO	Control
AT1G7233(	8	3	60,111	0,024195085	-1,61627	8,564925	1mg	Control
AT2G1817(	2	1	10,9607	0,024212527	-1,61596	5,512671	1mg	Control
AT5G1976(	18	16	161,595	0,024253059	-1,61523	1,198461	DMSO	1mg
AT5G6628(	4	1	23,2251	0,024348235	-1,61353	4,652609	1mg	DMSO
AT3G1644(	5	2	49,9829	0,024656293	-1,60807	1,701905	Control	1mg
AT3G0159(	1	1	5,0837	0,024946554	-1,60299	1,797322	Control	1mg
AT3G5561(	2	1	11,5246	0,025010769	-1,60187	1,620492	DMSO	Control
AT1G1306(	9	7	72,0581	0,025022935	-1,60166	1,235171	Control	1mg
AT1G5206(	6	2	36,7947	0,025270477	-1,59739	1,818656	Control	1mg
AT4G0208(	12	3	108,0737	0,025300183	-1,59688	2,045736	DMSO	Control
AT5G4893(	2	1	10,8341	0,025406354	-1,59506	1,821921	Control	DMSO
AT5G6594(	2	1	10,3675	0,025549868	-1,59261	2,984721	DMSO	1mg
AT3G1392(	28	2	340,695	0,025714229	-1,58983	1,509525	DMSO	1mg
AT5G0179(	1	1	5,4566	0,026058163	-1,58406	8,460048	1mg	Control
AT1G6596(	15	4	118,8908	0,026384007	-1,57866	1,591854	1mg	Control
AT4G2841(	1	1	12,892	0,026652959	-1,57425	1,936785	Control	1mg

AT5G1398(	14	11	81,4772	0,02671197	-1,57329	1,251383	Control	1mg
AT1G3035(	2	2	10,4235	0,026721472	-1,57314	3,994284	DMSO	1mg
AT5G4438(	7	4	47,7066	0,02750669	-1,56056	1,91939	DMSO	Control
AT1G2110(	3	1	16,9671	0,02756569	-1,55963	1,844703	1mg	DMSO
AT1G5990(	15	4	106,477	0,027931601	-1,5539	1,344656	Control	1mg
AT4G0932(	10	8	126,4657	0,027999624	-1,55285	1,267878	1mg	DMSO
AT2G4084(	5	4	33,5913	0,0280469	-1,55212	1,50832	DMSO	1mg
AT1G1569(	8	8	61,7816	0,028357337	-1,54733	1,610938	DMSO	1mg
AT1G6627(	29	12	345,6336	0,028543221	-1,5445	3,298146	Control	1mg
AT2G4347(	1	1	5,8372	0,028772069	-1,54103	2,584948	Control	DMSO
AT2G4415(	1	1	5,2013	0,029180327	-1,53491	2,140019	Control	DMSO
AT1G2390(	8	6	48,4537	0,029248122	-1,5339	1,486789	Control	1mg
AT5G4577(	1	1	5,2054	0,029415772	-1,53142	1,946134	1mg	Control
AT2G2061(	14	2	88,9915	0,029431199	-1,53119	1,364957	1mg	DMSO
AT1G7609(	5	2	32,8925	0,029651319	-1,52796	2,643784	DMSO	Control
AT5G6410(	15	11	118,0618	0,029704359	-1,52718	1,674857	DMSO	Control
AT1G7806(	4	2	22,5719	0,030254944	-1,5192	1,558717	1mg	Control
AT4G2369(	9	5	64,9105	0,03025615	-1,51919	1,332195	DMSO	1mg
AT1G6440(	3	1	30,9609	0,030587979	-1,51445	2,90045	1mg	Control
AT1G4317(	13	9	114,3086	0,030616836	-1,51404	3,993736	DMSO	Control
AT3G5116(	8	6	54,4217	0,030788168	-1,51162	1,416224	Control	DMSO
AT1G6314(	1	1	5,6072	0,030908513	-1,50992	9,759091	1mg	DMSO
AT1G0908(	18	9	122,9041	0,031015403	-1,50842	1,494656	Control	1mg
AT1G5241(	2	1	13,7946	0,031093415	-1,50733	11,95392	Control	1mg
AT4G0491(	22	18	142,1555	0,031109702	-1,5071	1,2882	DMSO	1mg
AT4G2667(	4	3	26,9128	0,031265722	-1,50493	1,753298	Control	1mg
ATMG0016	2	2	12,5071	0,031276466	-1,50478	1,567309	Control	1mg
AT4G2922(	5	1	28,5404	0,031458942	-1,50226	1,58989	Control	DMSO
AT1G8060(	5	3	30,0972	0,031737791	-1,49842	1,300874	Control	DMSO
AT5G3559(	13	5	78,0201	0,031780618	-1,49784	1,140415	1mg	DMSO
AT2G3334(	3	1	16,7913	0,032134659	-1,49303	1,424463	Control	1mg
AT3G6045(	3	3	24,3265	0,032263069	-1,49129	2,983403	DMSO	1mg
AT5G5991(	4	4	30,9119	0,032497634	-1,48815	3,317543	DMSO	1mg
AT2G4751(	15	9	129,1937	0,032680982	-1,4857	1,227675	Control	DMSO
AT3G0981(	8	2	56,6954	0,032798014	-1,48415	1,358148	Control	DMSO
ATCG0048(	12	5	91,5604	0,032865559	-1,48326	2,198733	Control	1mg
AT2G3169(	2	1	10,786	0,032879474	-1,48308	1,710251	DMSO	1mg
AT5G6079(	5	5	29,586	0,033393887	-1,47633	1,441945	DMSO	Control
AT5G1738(	13	10	90,4118	0,034061259	-1,46774	1,702787	Control	1mg
AT4G3619!	3	3	22,9776	0,034085572	-1,46743	1,409029	DMSO	Control
AT4G1389(	4	2	37,5889	0,034122237	-1,46696	3,977482	DMSO	Control
AT1G5385(	5	1	50,4573	0,034624311	-1,46062	2,532639	1mg	DMSO
AT1G7975(	12	9	83,6587	0,034790685	-1,45854	1,854028	Control	1mg
AT1G1356(	1	1	6,0344	0,034978368	-1,4562	1,611435	DMSO	Control
AT3G1506(	5	1	32,7127	0,035299533	-1,45223	2,521879	Control	1mg
AT5G2588(	12	2	109,0691	0,035926499	-1,44459	5,956808	Control	DMSO
AT4G3199(	18	13	150,9688	0,035964481	-1,44413	1,281663	Control	DMSO
AT3G5485(	5	2	32,846	0,036303411	-1,44005	2,213733	DMSO	1mg
AT3G0252(	15	1	118,8217	0,036414496	-1,43873	4,094631	1mg	Control
AT1G5168(	4	2	22,9898	0,036463049	-1,43815	1,288101	1mg	Control

AT1G0646(	1	1	5,7465	0,03669868	-1,43535	6,909091	Control	1mg
AT5G1225(	24	4	277,3153	0,03679699	-1,43419	1,510581	DMSO	1mg
AT1G0441(	22	10	324,5033	0,03692549	-1,43267	1,629841	DMSO	1mg
AT4G3170(	4	1	24,2124	0,037705592	-1,42359	1,908221	DMSO	Control
AT2G1763(	14	6	100,0588	0,037828598	-1,42218	1,395529	1mg	Control
AT1G7999(	18	10	111,3162	0,038096948	-1,41911	1,139513	Control	1mg
AT2G2217(	7	2	89,6	0,038275348	-1,41708	1,787384	DMSO	1mg
AT5G4946(	22	4	201,6459	0,038719935	-1,41207	1,306542	Control	1mg
AT1G7751(	18	7	128,9713	0,038818657	-1,41096	1,2806	1mg	Control
AT3G4869(	1	1	5,3674	0,038958091	-1,4094	1,499036	Control	1mg
AT1G0233!	5	1	38,3936	0,038981728	-1,40914	3,788135	1mg	Control
AT4G3423(	14	9	89,6354	0,039045559	-1,40843	1,227101	Control	DMSO
AT1G2745(	10	2	83,0607	0,039152341	-1,40724	5,297218	Control	1mg
AT3G0223(	28	8	298,4348	0,039404469	-1,40445	1,329359	1mg	DMSO
AT4G3696(	3	2	16,0044	0,039480087	-1,40362	2,610842	Control	1mg
AT5G5702(	4	4	28,9332	0,039517302	-1,40321	1,278773	Control	1mg
AT1G7640(	9	5	55,7786	0,039684205	-1,40138	1,304525	DMSO	Control
AT4G1884(	2	1	11,8214	0,039916017	-1,39885	3,744585	DMSO	1mg
AT1G6067(	1	1	5,5425	0,040020853	-1,39771	5,772596	1mg	Control
AT3G0553(	16	7	105,6983	0,040025942	-1,39766	1,520217	1mg	Control
AT3G5997(	28	17	253,3435	0,04008589	-1,39701	1,402953	DMSO	1mg
AT3G5425(	8	3	50,3752	0,040093524	-1,39693	1,561189	Control	1mg
AT5G0369(	13	3	155,519	0,040379036	-1,39384	1,568017	Control	1mg
AT2G1986(	10	5	56,3231	0,040544348	-1,39207	2,03629	1mg	DMSO
AT1G0956(	5	3	47,2449	0,040709365	-1,39031	1,638622	Control	DMSO
AT1G6202(	42	10	320,6634	0,040902342	-1,38825	1,300589	DMSO	1mg
AT2G2975(	3	3	16,1743	0,040964656	-1,38759	1,944919	Control	1mg
AT1G0481(	13	2	101,3697	0,041282431	-1,38423	1,611326	Control	DMSO
AT2G4629(	1	1	5,6103	0,042039081	-1,37635	3,208265	Control	DMSO
AT4G3716(	6	4	43,0023	0,042112607	-1,37559	2,060533	DMSO	Control
AT2G4581(	2	1	11,1572	0,042327319	-1,37338	1,38694	Control	1mg
AT5G0816(	1	1	5,0661	0,042390606	-1,37273	2,114694	DMSO	Control
AT1G5149(	7	3	50,6401	0,042561299	-1,37099	7,172788	Control	1mg
AT1G6099(	3	1	26,8759	0,042593458	-1,37066	1,428227	Control	1mg
AT4G0495(	2	2	11,5719	0,042606665	-1,37052	2,586823	DMSO	1mg
AT2G1873(	3	2	16,371	0,042966826	-1,36687	1,207526	1mg	DMSO
AT1G2380(	8	5	54,2182	0,043151731	-1,365	1,591272	1mg	DMSO
AT1G2244(	10	8	63,8028	0,043334588	-1,36317	1,442203	Control	1mg
AT1G5614!	3	1	20,584	0,043444354	-1,36207	1,718818	Control	1mg
AT1G5989(	5	3	31,9732	0,043927737	-1,35726	1,260314	Control	1mg
AT5G4039(	13	7	80,1743	0,043975598	-1,35679	1,346901	1mg	Control
AT3G2483(	2	1	11,1023	0,044015411	-1,3564	14,39601	DMSO	1mg
AT5G3894(	4	1	48,499	0,044215836	-1,35442	2,082812	1mg	Control
AT2G1754(	3	2	16,4872	0,044569557	-1,35096	1,881265	DMSO	1mg
AT4G3191(	2	2	11,1211	0,045078566	-1,34603	1,507722	DMSO	Control
AT2G3537(	2	1	16,8605	0,04551908	-1,34181	5,192284	1mg	Control
AT2G1726!	10	4	74,0683	0,045849127	-1,33867	1,670836	1mg	Control
AT5G6064(	11	10	82,6563	0,04612209	-1,33609	1,434138	1mg	Control
AT3G4752(	19	15	204,2285	0,046183735	-1,33551	1,383032	1mg	Control
AT4G3510(	7	4	83,3108	0,04633444	-1,3341	1,388703	Control	1mg

AT2G2476	3	2	17,5528	0,047200648	-1,32605	1,355786	Control	1mg
AT3G1239(	7	5	63,4653	0,047340338	-1,32477	1,379144	Control	1mg
AT3G0605(	7	5	59,8472	0,047845084	-1,32016	1,503556	1mg	Control
AT3G5646(	2	1	10,484	0,047858686	-1,32004	2,609366	DMSO	1mg
AT2G4375(	9	6	57,5462	0,04791329	-1,31954	1,260477	Control	DMSO
AT4G3309(	34	18	237,129	0,048017539	-1,3186	1,170687	DMSO	1mg
AT1G5427(	28	5	324,2237	0,048418174	-1,31499	1,465117	1mg	Control
AT5G3974(	7	6	80,3025	0,048446861	-1,31473	1,644551	Control	1mg
AT1G2731(	3	3	21,1682	0,048514095	-1,31413	1,43406	1mg	DMSO
AT3G5318(	9	3	61,0807	0,048973563	-1,31004	3,184004	1mg	Control
AT5G3247(	1	1	5,6238	0,049273448	-1,30739	2,386753	Control	1mg
AT5G6429(	2	2	11,1281	0,049627249	-1,30428	1,468351	DMSO	Control
AT5G2610(	1	1	5,3671	0,049628237	-1,30427	1,533312	1mg	Control
AT1G6773(	9	7	56,3354	0,049947632	-1,30149	1,315471	Control	1mg
AT4G1721(	1	1	5,8537	0,050186598	-1,29941	3,817197	DMSO	Control
AT2G2125(	1	1	5,6328	0,050292733	-1,29849	1,922776	Control	DMSO
AT5G6527(	3	1	24,2274	0,050488066	-1,29681	1,779386	1mg	Control
AT3G0894	7	3	38,3928	0,050653498	-1,29539	1,852675	DMSO	Control
AT4G3613(	9	2	87,724	0,050707349	-1,29493	2,050406	DMSO	Control
AT3G2360(	4	1	35,0418	0,050741512	-1,29464	4,402378	DMSO	1mg
AT2G2771(	9	6	83,9495	0,050919151	-1,29312	1,59278	1mg	Control
AT3G4677(	1	1	5,2628	0,051487748	-1,2883	1,497683	Control	DMSO
AT4G1666(	6	2	34,6129	0,051534084	-1,28791	1,415912	DMSO	1mg
AT5G1999(	10	7	66,9117	0,051723173	-1,28631	1,281969	Control	DMSO
AT5G4412(	8	3	54,7667	0,05213216	-1,28289	6,294082	DMSO	1mg
AT2G3804(	15	7	84,222	0,052170572	-1,28257	1,488425	DMSO	Control
AT3G2918(	3	2	28,4954	0,052396951	-1,28069	1,558837	Control	1mg
AT1G2797(	7	6	53,2091	0,052556091	-1,27938	1,659521	Control	1mg
AT3G5311(	4	2	21,4536	0,052562138	-1,27933	1,995963	Control	1mg
AT1G2915(	15	12	124,9378	0,053075636	-1,2751	1,297791	DMSO	1mg
AT1G2941(	2	2	21,9082	0,054124467	-1,26661	1,811799	Control	1mg
AT5G4432(	9	3	55,7884	0,054329003	-1,26497	1,897703	Control	DMSO
AT1G7470(	2	1	11,2142	0,054779442	-1,26138	2,104069	Control	DMSO
AT2G4765(	13	1	80,7053	0,055293881	-1,25732	8,964425	DMSO	1mg
AT5G2235(	2	1	10,2022	0,055494735	-1,25575	4,380231	DMSO	1mg
AT4G3315(	9	4	53,5479	0,055581525	-1,25507	1,821888	Control	DMSO
AT1G1826	1	1	5,2643	0,055606817	-1,25487	9,505343	Control	1mg
AT3G5701(	1	1	6,0864	0,055695041	-1,25418	2,373046	1mg	DMSO
AT5G5289(	1	1	5,254	0,055737847	-1,25385	2,699523	Control	1mg
AT4G1416(	17	11	117,5353	0,056112799	-1,25094	1,303415	DMSO	1mg
AT1G8005(	3	1	20,8768	0,056334126	-1,24923	6,83892	Control	1mg
AT3G5528(	2	1	13,576	0,056483814	-1,24808	19,32634	DMSO	1mg
AT3G1982(	16	9	116,1896	0,057059504	-1,24367	1,573683	1mg	Control
AT1G5619(	13	4	118,8386	0,057136092	-1,24309	1,135873	1mg	Control
AT4G1032(	25	18	156,6476	0,057562739	-1,23986	1,361726	Control	1mg
AT2G3704(	11	4	76,9102	0,057688581	-1,23891	1,317265	DMSO	1mg
AT3G5119(	4	1	42,0458	0,057795075	-1,23811	1,76438	Control	DMSO
AT5G1951(	10	7	120,0269	0,057847552	-1,23772	1,565173	DMSO	1mg
AT1G1647(	9	5	73,746	0,058315903	-1,23421	1,424754	Control	1mg
AT3G3018(	2	2	10,527	0,058368147	-1,23382	2,165373	DMSO	Control

AT5G1077(	5	5	29,3189	0,058467907	-1,23308	1,529771	DMSO	Control
AT3G5184(	7	6	42,9851	0,058607724	-1,23205	1,559635	1mg	Control
AT5G3485(	10	8	76,9183	0,058933048	-1,22964	1,570029	DMSO	1mg
AT3G2548(	3	3	22,0518	0,060057217	-1,22143	4,642468	Control	1mg
AT1G4890(	3	2	17,1323	0,060295742	-1,21971	1,492384	DMSO	1mg
AT5G1607(	19	2	173,5016	0,060324756	-1,2195	1,528264	Control	DMSO
AT4G1189(	3	2	15,7754	0,060364642	-1,21922	2,465562	DMSO	1mg
AT4G1726(	6	3	42,5043	0,060431305	-1,21874	1,7582	1mg	Control
AT1G0180(	2	2	17,7776	0,060672315	-1,21701	2,775953	1mg	DMSO
AT1G3023(	11	5	111,5399	0,060737605	-1,21654	2,123463	DMSO	Control
AT2G0416(	2	1	10,7547	0,060978716	-1,21482	2,301854	Control	1mg
AT3G1948(	11	5	87,1906	0,06105555	-1,21427	1,651345	Control	1mg
AT5G0210(	8	3	48,7021	0,061530074	-1,21091	5,339396	1mg	DMSO
AT2G2360(	1	1	5,9812	0,061560135	-1,2107	3,219909	1mg	Control
AT3G0391(	8	1	65,7448	0,062832412	-1,20182	3,517915	DMSO	1mg
AT3G1664(	9	8	126,0092	0,062921356	-1,2012	1,26696	DMSO	1mg
AT1G3118(	11	4	74,5417	0,063122212	-1,19982	1,245714	Control	1mg
AT4G3787(	19	9	124,4663	0,063130281	-1,19976	1,270941	DMSO	1mg
AT4G2852(	4	4	25,3807	0,063369496	-1,19812	10,85695	DMSO	Control
AT5G2033(	2	2	10,7637	0,063588944	-1,19662	1,731887	DMSO	1mg
AT3G5358(	8	5	51,035	0,063899199	-1,1945	1,771596	1mg	Control
AT3G1640(	30	7	357,0018	0,06390035	-1,1945	1,371256	DMSO	1mg
AT4G1122(	2	2	12,384	0,063922015	-1,19435	2,033869	DMSO	Control
AT4G2967(	1	1	5,22	0,06452374	-1,19028	1,86071	DMSO	1mg
AT2G3658(	26	6	208,448	0,064525999	-1,19027	1,381273	DMSO	Control
AT1G4319(	3	2	17,4062	0,064618483	-1,18964	1,79693	1mg	Control
AT2G2133(	3	1	18,9943	0,064952828	-1,1874	2,125775	1mg	Control
AT3G2869(	1	1	5,4145	0,065191033	-1,18581	1,582287	1mg	Control
AT2G2166(	9	5	67,1104	0,066240318	-1,17888	2,263743	1mg	Control
AT1G7360(	8	1	60,9594	0,066465153	-1,17741	1,627987	1mg	Control
AT3G1886(	8	4	49,838	0,066678308	-1,17602	1,828756	DMSO	1mg
AT4G3441;	2	1	11,2565	0,06673245	-1,17566	4,154211	1mg	Control
AT4G3053(	3	1	25,9901	0,066924695	-1,17441	1,940999	Control	1mg
AT5G1120(	18	1	161,2069	0,067211472	-1,17256	1,878519	DMSO	1mg
AT4G2000(	2	2	17,7537	0,067748072	-1,1691	1,499049	Control	1mg
AT5G6096(	2	2	11,0796	0,067777312	-1,16892	2,011684	1mg	Control
AT1G2172(	5	4	47,8328	0,067839793	-1,16852	1,355186	1mg	Control
AT3G2743(	1	1	6,3866	0,067997457	-1,16751	1,477466	1mg	Control
AT4G2563(	11	3	73,5565	0,068470075	-1,1645	1,528629	DMSO	Control
AT4G3336(	10	8	69,9103	0,069080875	-1,16064	1,438748	DMSO	1mg
AT1G2994(	4	2	25,3702	0,069268066	-1,15947	1,788231	Control	1mg
AT1G2001(	23	4	260,5679	0,070180368	-1,15378	1,346829	DMSO	1mg
AT1G5401(	14	7	125,0038	0,07028721	-1,15312	1,338055	DMSO	1mg
AT4G3301(	26	6	199,8183	0,070776053	-1,15011	1,591231	Control	1mg
AT1G1067(	12	4	89,275	0,07179199	-1,14392	1,52785	Control	1mg
AT5G5078(	3	1	17,2125	0,071903238	-1,14325	2,661617	DMSO	1mg
AT3G1393(	14	11	105,1516	0,071939223	-1,14303	1,402633	DMSO	1mg
AT2G4522(	11	8	78,5004	0,072780037	-1,13799	1,19583	Control	1mg
AT1G6620(	16	3	175,2733	0,072979579	-1,1368	1,580561	DMSO	1mg
AT3G1596(	2	1	10,9866	0,073224744	-1,13534	1,423607	1mg	Control

AT1G1759(	1	1	6,4775	0,073466772	-1,13391	23,69523	Control	DMSO
AT5G5337(	3	1	18,3675	0,073516242	-1,13362	10,14165	1mg	DMSO
AT3G6082(	6	6	48,866	0,074216382	-1,1295	1,553937	Control	1mg
AT2G4625(	2	1	11,0626	0,074358576	-1,12867	4,992255	1mg	Control
AT2G4418(	1	1	5,5843	0,075106688	-1,12432	5,05047	DMSO	1mg
AT1G6075(	4	1	22,3341	0,075169729	-1,12396	16,25382	DMSO	1mg
AT4G2083(	2	1	11,283	0,075543472	-1,1218	1,681681	DMSO	Control
AT5G1978(	24	2	326,4368	0,075620851	-1,12136	1,947598	1mg	Control
AT2G3959(	5	2	42,368	0,075856499	-1,12001	1,543769	Control	1mg
AT5G4991(	16	1	111,0059	0,075889835	-1,11982	1,588333	Control	1mg
AT2G0478(	6	6	77,6765	0,076102084	-1,1186	1,418409	DMSO	1mg
AT4G2968(	6	6	43,0572	0,076359972	-1,11713	1,546024	Control	1mg
AT2G4628(	7	6	47,4775	0,076520435	-1,11622	1,480083	DMSO	1mg
AT3G6013(	3	2	17,5511	0,076821224	-1,11452	1,419838	Control	1mg
AT5G6736(	3	3	16,773	0,076990995	-1,11356	1,524562	1mg	Control
AT4G2428(	21	3	144,4827	0,077379335	-1,11138	1,429098	1mg	Control
AT1G0526(	14	11	132,4719	0,077588421	-1,1102	1,321921	DMSO	1mg
AT3G1191(	4	1	25,4206	0,077610726	-1,11008	4,067069	1mg	Control
AT5G1588(	1	1	5,9466	0,07786167	-1,10868	1,292936	1mg	Control
AT1G1158(	21	15	231,7085	0,078144965	-1,1071	1,559874	1mg	Control
AT1G0631(	3	1	16,4426	0,078412365	-1,10562	3,470683	Control	1mg
AT1G7934(	9	6	54,3066	0,078418967	-1,10558	1,412074	DMSO	1mg
AT5G2785(	8	7	69,0584	0,079866898	-1,09763	2,211649	DMSO	1mg
AT5G0955(	12	4	93,5737	0,080149744	-1,0961	2,671143	Control	1mg
AT3G1813(	18	5	165,0445	0,080319781	-1,09518	1,43038	DMSO	Control
AT3G1173(	4	1	34,1772	0,080605772	-1,09363	1,409091	1mg	DMSO
AT1G4847(	5	2	41,5762	0,080841314	-1,09237	1,390133	Control	DMSO
AT3G1642(	22	9	305,4277	0,080929496	-1,09189	1,236557	DMSO	1mg
AT4G2346(	14	1	100,8921	0,081035207	-1,09133	1,724498	Control	1mg
AT3G1278(	16	9	143,9884	0,081086454	-1,09105	1,264048	1mg	Control
AT3G2037(	21	16	203,0008	0,081314391	-1,08983	1,406197	DMSO	Control
AT3G2380(	1	1	5,7481	0,081812801	-1,08718	2,140002	1mg	DMSO
AT5G1474(	2	2	12,0039	0,083237119	-1,07968	1,818805	Control	1mg
AT3G0944(	38	13	423,6273	0,083373493	-1,07897	1,232173	Control	1mg
AT3G1178(	1	1	5,738	0,08356606	-1,07797	1,218311	Control	DMSO
AT2G0266(	3	2	27,8824	0,084076956	-1,07532	2,425961	DMSO	1mg
AT4G3706(	2	1	11,7088	0,084957455	-1,0708	3,65264	Control	DMSO
AT1G3327(	1	1	5,374	0,085189595	-1,06961	1,551826	DMSO	Control
AT3G1038(	3	3	15,6224	0,085214343	-1,06949	4,323727	Control	1mg
AT4G3526(	13	3	112,5954	0,0854848	-1,06811	1,360156	1mg	Control
AT3G0129(	14	9	127,8418	0,085508639	-1,06799	1,379757	DMSO	1mg
AT5G5385(	4	3	21,1128	0,085674307	-1,06715	1,674953	DMSO	1mg
AT1G6300(	17	11	151,4366	0,0857613	-1,06671	1,230731	DMSO	Control
AT5G0640(	5	2	31,4711	0,085773242	-1,06665	1,400687	Control	DMSO
AT4G0110(	2	1	10,2943	0,086431245	-1,06333	3,247685	DMSO	1mg
AT2G4089(	3	3	17,0718	0,087005593	-1,06045	1,425174	1mg	Control
AT3G0672(	10	3	67,2468	0,087395257	-1,05851	1,618141	1mg	Control
AT4G3365(	7	4	38,1875	0,087551799	-1,05773	1,391094	DMSO	1mg
AT4G2797(	1	1	12,3452	0,087685209	-1,05707	6,237233	DMSO	Control
AT2G4453(	2	1	16,7156	0,088307168	-1,054	1,996668	1mg	DMSO

AT5G5749(	3	2	27,8785	0,088612815	-1,0525	3,922214	1mg	Control
AT3G5857(	9	2	61,9741	0,089112487	-1,05006	1,618257	1mg	Control
AT4G0674(	1	1	6,1884	0,089122991	-1,05001	2,058353	DMSO	1mg
AT5G1782(	16	13	148,7719	0,089476044	-1,04829	1,846769	DMSO	1mg
AT3G5976(	15	12	95,1745	0,092115992	-1,03566	1,348194	Control	DMSO
AT5G0598(	3	2	16,9778	0,092587638	-1,03345	2,921888	1mg	Control
AT3G2596(	9	1	55,1609	0,092851597	-1,03221	2,086378	Control	1mg
AT4G3966(	17	14	142,1286	0,092867104	-1,03214	1,285611	DMSO	Control
AT1G8046(	23	22	183,759	0,093256281	-1,03032	1,27617	Control	1mg
AT5G6384(	11	7	61,6006	0,093339479	-1,02993	1,17052	1mg	Control
AT5G3553(	13	1	151,419	0,093372551	-1,02978	2,073533	Control	1mg
AT2G4652(	6	4	37,1728	0,093656424	-1,02846	1,897242	1mg	Control
AT5G0735(	32	13	217,3383	0,094181479	-1,02603	1,406287	DMSO	Control
AT1G7084(	8	4	55,5716	0,094284836	-1,02556	1,389442	1mg	Control
AT3G2580(	16	5	103,2252	0,094291038	-1,02553	1,461923	DMSO	Control
AT4G3445(	44	35	379,4935	0,094526295	-1,02445	1,0928	DMSO	1mg
AT1G7077(	14	12	89,5731	0,09454238	-1,02437	1,265195	DMSO	Control
AT1G1531(	2	1	10,8945	0,094746853	-1,02344	2,220697	Control	1mg
AT3G4352(	1	1	5,9167	0,094940644	-1,02255	3,708993	1mg	Control
AT3G1499(	24	21	259,8398	0,095589961	-1,01959	1,318397	Control	1mg
AT3G4781(	1	1	5,819	0,096354983	-1,01613	3,711867	Control	1mg
AT5G6151(	7	6	45,3477	0,096470163	-1,01561	1,839462	DMSO	1mg
AT1G2451(	15	11	99,5183	0,096971921	-1,01335	1,2301	DMSO	Control
AT2G3292(	9	8	63,7764	0,09781779	-1,00958	1,137393	DMSO	Control
AT1G5240(	7	3	65,958	0,098523649	-1,00646	1,409895	1mg	Control
AT1G5176(	2	1	11,1612	0,0988255	-1,00513	3,702779	DMSO	1mg
AT4G2949(	2	1	10,5463	0,100165659	-0,99928	3,228456	1mg	Control
AT1G2274(	7	2	47,4769	0,100824305	-0,99643	3,508852	Control	1mg
AT2G3998(	2	2	10,2921	0,101732343	-0,99254	1,463221	Control	1mg
AT1G0767(	6	4	32,7056	0,102151547	-0,99076	1,477865	DMSO	Control
AT3G2394(	17	10	115,5176	0,103339025	-0,98574	1,420676	Control	1mg
AT3G6144(	7	3	43,3805	0,103452543	-0,98526	1,628204	1mg	DMSO
AT4G2942(	2	1	10,8723	0,103796015	-0,98382	1,743229	1mg	Control
AT1G0883(	4	3	37,9545	0,104950638	-0,97901	1,991088	1mg	DMSO
AT2G3139(	21	8	219,7264	0,105093972	-0,97842	1,410084	1mg	Control
AT4G3487(	12	6	158,418	0,105538454	-0,97659	1,225989	1mg	Control
AT5G5765!	6	4	40,3388	0,105830211	-0,97539	1,450111	Control	DMSO
AT1G3443(	18	10	135,2402	0,106489679	-0,97269	1,235037	Control	1mg
AT5G3621(	2	1	11,0344	0,106536762	-0,9725	1,716792	1mg	Control
AT5G1592(	2	1	12,0054	0,106595181	-0,97226	1,327186	Control	1mg
AT5G6389(	6	4	41,3235	0,106702068	-0,97183	1,279491	1mg	Control
AT3G1794(	4	4	24,1736	0,106975633	-0,97072	1,331925	DMSO	Control
AT3G4217(	5	3	34,5999	0,107245134	-0,96962	1,557925	DMSO	1mg
AT1G3244(	8	2	63,4273	0,107963654	-0,96672	4,754794	Control	1mg
AT1G7273(	20	4	254,0324	0,108165979	-0,96591	1,214167	1mg	DMSO
AT4G0244(	1	1	5,3654	0,108629135	-0,96405	4,61878	Control	1mg
AT1G3012(	10	3	77,5837	0,108912747	-0,96292	1,323159	Control	1mg
AT1G8066(	10	1	63,6548	0,1090429	-0,9624	1,355991	1mg	DMSO
AT1G7492(	10	7	66,105	0,109192346	-0,96181	1,428788	Control	DMSO
AT4G1615!	9	3	52,8588	0,109243254	-0,96161	1,459574	Control	1mg

AT3G5193(	2	1	10,2022	0,109379703	-0,96106	8,538921	DMSO	1mg
AT2G1685(	3	1	43,8602	0,109891184	-0,95904	1,385477	Control	DMSO
AT3G1041(	4	2	21,7225	0,110215792	-0,95776	1,557247	DMSO	1mg
AT2G0465(	3	2	20,7408	0,111559322	-0,95249	2,002579	Control	1mg
AT4G0258(	4	4	30,2256	0,111898718	-0,95117	1,531243	DMSO	Control
ATCG0012(	9	5	70,1025	0,11205793	-0,95056	1,297763	Control	1mg
AT3G0685(	2	1	17,3792	0,112062862	-0,95054	2,080073	1mg	Control
AT5G1762(	2	1	11,3099	0,112637527	-0,94832	11,93636	Control	1mg
AT4G2901(	12	10	77,6197	0,112732587	-0,94795	1,213552	Control	1mg
AT4G1376(	1	1	5,6406	0,112902137	-0,9473	5,057224	1mg	DMSO
AT1G3476(	7	2	59,2176	0,113078926	-0,94662	1,598155	1mg	Control
AT5G1420(	11	7	77,3737	0,113115543	-0,94648	1,509574	DMSO	Control
AT3G5678(	2	2	11,108	0,113317781	-0,9457	1,407524	Control	DMSO
AT1G1292(	7	4	38,815	0,113460187	-0,94516	1,468067	1mg	Control
AT4G2392(	2	1	15,6105	0,114106879	-0,94269	2,034224	Control	DMSO
AT3G2728(	2	1	11,5477	0,114329413	-0,94184	5,48959	Control	1mg
AT2G4174(	9	5	59,7275	0,114571494	-0,94092	1,392632	Control	1mg
AT1G3558(	16	10	137,2636	0,114962178	-0,93945	1,383761	Control	1mg
AT1G4533:	3	3	15,5846	0,115293513	-0,9382	1,90575	1mg	DMSO
AT5G0329(	9	3	75,388	0,115381658	-0,93786	1,277478	Control	DMSO
AT5G5829(	17	10	110,0459	0,115393189	-0,93782	1,286428	Control	1mg
AT3G0686(	26	19	164,0775	0,115551426	-0,93722	1,212718	DMSO	1mg
AT3G6024!	1	1	6,9617	0,115810102	-0,93625	3,508901	DMSO	Control
AT1G6409(	1	1	5,9384	0,116103725	-0,93515	1,731418	DMSO	Control
AT2G0403(	17	9	99,2141	0,116301642	-0,93441	1,200774	DMSO	1mg
AT1G2175(	35	27	322,6048	0,116336859	-0,93428	1,205525	DMSO	Control
AT1G2144(	3	2	16,5781	0,118334851	-0,92689	1,863699	1mg	Control
AT3G5609(	4	2	21,9849	0,118828283	-0,92508	1,554365	1mg	DMSO
AT1G6078(	3	2	21,4201	0,118876571	-0,9249	1,387368	DMSO	1mg
AT3G0414(	4	4	21,9598	0,118901064	-0,92481	1,370333	Control	1mg
AT5G1565(	29	10	316,8375	0,118928073	-0,92472	1,235274	1mg	DMSO
AT2G4416(	23	13	204,0086	0,119940422	-0,92103	1,247126	DMSO	1mg
AT1G7868(	8	3	48,1735	0,120772267	-0,91803	1,666515	DMSO	Control
AT1G7085(	17	9	157,4213	0,121511825	-0,91538	1,797651	DMSO	1mg
AT1G5987(	31	12	185,8151	0,122307347	-0,91255	1,57074	1mg	Control
AT1G7533(	3	3	16,8029	0,122359112	-0,91236	1,758409	Control	1mg
AT5G5028(	4	3	27,6613	0,122395228	-0,91224	3,247078	Control	1mg
AT3G5323(	34	3	307,1082	0,122757676	-0,91095	1,423522	Control	DMSO
AT3G0777(	22	13	157,6566	0,123158055	-0,90954	1,223393	DMSO	1mg
AT3G5124(	1	1	5,4582	0,123312233	-0,90899	2,58445	DMSO	1mg
AT3G1431(	18	9	152,6578	0,123433518	-0,90857	1,452166	1mg	Control
AT3G1379(	16	8	96,3492	0,123453621	-0,9085	1,353427	DMSO	1mg
AT3G1127(	8	6	65,1039	0,1237845	-0,90733	1,898135	Control	1mg
AT1G5607(	65	31	790,3207	0,124482314	-0,90489	1,510809	DMSO	1mg
AT4G1336(	2	1	11,3872	0,124535214	-0,90471	2,290072	Control	DMSO
AT3G5021(	1	1	5,4917	0,12470773	-0,90411	2,219252	Control	1mg
AT4G2419(	42	28	337,2012	0,125749906	-0,90049	1,181223	DMSO	Control
AT3G5861(	29	22	267,7883	0,125750895	-0,90049	1,32124	Control	1mg
AT2G4040(	2	1	16,9671	0,12797265	-0,89288	2,519807	1mg	Control
AT5G4609(	1	1	6,2238	0,12819438	-0,89213	2,101775	DMSO	Control

AT2G3304(	13	13	136,0389	0,128348007	-0,89161	1,482794	1mg	Control
AT3G1573(	40	25	383,4243	0,128588655	-0,8908	1,292582	DMSO	1mg
AT1G1344(	30	1	419,7268	0,128902497	-0,88974	2,285861	DMSO	1mg
AT1G3491(	1	1	5,6525	0,129211054	-0,8887	1,79685	Control	DMSO
AT3G0851(	3	3	21,9646	0,129778628	-0,8868	1,477381	DMSO	Control
AT1G0964(	14	3	132,4501	0,13039471	-0,88474	1,165889	1mg	DMSO
AT3G6236(	13	9	71,0839	0,130682505	-0,88378	1,29127	DMSO	Control
AT1G7060(	3	2	22,4536	0,1309257	-0,88298	3,11477	DMSO	Control
AT1G1095(	2	2	10,7833	0,131101247	-0,88239	2,922984	DMSO	Control
AT1G7944(	9	7	52,9541	0,131452875	-0,88123	1,210723	1mg	DMSO
AT4G1378(	3	3	16,5575	0,131573029	-0,88083	2,006878	1mg	Control
AT1G1854(	2	2	11,7314	0,131673929	-0,8805	3,630491	DMSO	Control
AT1G5506(	10	3	82,5315	0,131678002	-0,88049	1,440341	1mg	Control
AT5G4126(	2	1	11,0464	0,132229149	-0,87867	1,468653	Control	1mg
AT1G1186(	17	12	120,9411	0,132243176	-0,87863	1,292196	Control	1mg
AT3G2340(	2	2	10,4357	0,132484577	-0,87783	1,432834	1mg	Control
AT1G0110(	2	2	11,7487	0,132603589	-0,87744	3,902061	1mg	DMSO
AT3G6021(	1	1	5,0861	0,133683884	-0,87392	1,342176	Control	DMSO
AT3G4433(	7	6	39,5171	0,133845955	-0,87339	1,206112	1mg	Control
AT3G5543(	3	3	21,9442	0,135041908	-0,86953	1,313404	Control	1mg
AT5G2851(	5	2	53,3541	0,135097391	-0,86935	1,345426	Control	1mg
AT2G4029(	5	5	31,6792	0,135135216	-0,86923	1,872432	Control	DMSO
AT5G1605(	15	1	119,0497	0,135210561	-0,86899	4,514133	DMSO	Control
AT1G1200(	22	12	161,532	0,135321342	-0,86863	1,440802	DMSO	Control
AT1G7655(	24	11	174,825	0,135701684	-0,86741	1,4826	Control	1mg
AT1G4863(	18	5	167,7792	0,135917582	-0,86672	1,592745	DMSO	1mg
AT1G1898(	1	1	5,9981	0,1360302	-0,86636	2,143457	DMSO	1mg
AT1G3616(	10	6	57,457	0,136221503	-0,86575	1,12523	Control	DMSO
AT1G6479(	21	17	126,1026	0,136928529	-0,86351	1,854911	1mg	DMSO
AT2G2141(	12	3	80,7085	0,137629816	-0,86129	2,250495	1mg	Control
AT1G6474(	14	1	178,3571	0,137738574	-0,86094	1,378996	Control	DMSO
AT1G7616(	13	9	86,7511	0,137896856	-0,86045	1,278189	1mg	Control
AT2G3718(	9	3	83,0497	0,138563633	-0,85835	1,359577	Control	1mg
AT2G1784(	9	8	64,7341	0,138669137	-0,85802	1,463449	DMSO	1mg
AT4G3418(	5	3	36,3744	0,138703219	-0,85791	1,32497	DMSO	1mg
AT5G5224(	1	1	5,5586	0,138823133	-0,85754	3,359747	DMSO	Control
AT2G1957(	2	1	12,906	0,139026625	-0,8569	1,339999	1mg	DMSO
AT3G0984(	42	12	391,6135	0,139055946	-0,85681	1,297443	1mg	Control
AT4G0352(	2	1	11,2254	0,139101478	-0,85667	2,008782	1mg	DMSO
AT1G1850(	4	4	22,0308	0,139508974	-0,8554	1,757669	1mg	Control
AT2G2212!	4	3	21,6316	0,140682121	-0,85176	2,665854	1mg	Control
AT1G1521(	19	5	118,161	0,140830419	-0,8513	1,24214	Control	1mg
AT3G6090(	8	2	64,8497	0,141740691	-0,84851	2,735327	1mg	Control
AT1G8049(	6	4	34,112	0,143045181	-0,84453	1,109589	Control	DMSO
AT1G6260(	1	1	5,4367	0,143055578	-0,8445	2,121025	Control	1mg
AT3G0853(	81	12	698,5925	0,143382505	-0,8435	1,482681	DMSO	Control
AT5G4225(	1	1	5,2494	0,143771739	-0,84233	1,879095	DMSO	Control
AT5G5443(	3	3	16,2825	0,143855144	-0,84207	1,2823	Control	1mg
AT1G7403(	19	13	165,6455	0,143984138	-0,84169	1,2039	1mg	Control
AT4G3457(	1	1	6,5084	0,144068715	-0,84143	8,801656	Control	1mg

AT5G5496(	11	7	67,7571	0,144485743	-0,84018	1,770537	1mg	DMSO
AT5G1152(	15	9	117,1554	0,145231936	-0,83794	1,176222	DMSO	1mg
AT5G1177(	7	6	49,4625	0,145258346	-0,83786	1,398446	1mg	Control
AT2G3049(	25	21	203,9729	0,146427588	-0,83438	1,179225	DMSO	1mg
AT1G6264(	9	6	59,9018	0,1477411	-0,8305	1,407607	DMSO	Control
AT1G1284(	20	16	156,7624	0,148375441	-0,82864	1,257703	1mg	Control
AT1G6394(	23	20	191,1003	0,148831431	-0,82731	1,189988	1mg	Control
AT5G6016(	8	6	58,8599	0,148991372	-0,82684	1,284844	1mg	DMSO
AT4G1404(	3	1	19,5337	0,149268542	-0,82603	2,548607	DMSO	Control
AT2G2139(	51	18	414,3479	0,149283241	-0,82599	1,31563	DMSO	1mg
AT2G3870(	14	7	83,9342	0,149691858	-0,8248	1,306959	DMSO	1mg
AT5G4276(	2	1	11,0544	0,150399566	-0,82275	3,494831	DMSO	1mg
AT3G1782(	17	8	146,6686	0,150672425	-0,82197	1,473823	DMSO	1mg
AT1G5358(	11	6	84,7075	0,151031641	-0,82093	1,462951	Control	1mg
AT4G3449(	6	5	46,9241	0,15129977	-0,82016	2,303054	Control	1mg
AT3G5501(	5	3	27,3453	0,151434536	-0,81978	1,866333	DMSO	Control
AT3G2366(	11	4	76,4236	0,151579422	-0,81936	1,71469	1mg	DMSO
AT3G0204(	1	1	5,8992	0,151721375	-0,81895	1,42297	Control	1mg
AT4G3351(	5	1	28,6029	0,152610111	-0,81642	1,380569	1mg	DMSO
AT2G2598(	15	10	101,9337	0,153053085	-0,81516	1,480304	Control	1mg
AT2G2248(	6	3	33,9094	0,153104446	-0,81501	1,60416	DMSO	Control
AT4G0293(	23	15	212,5891	0,15325061	-0,8146	1,222963	1mg	Control
AT4G3130(	10	9	96,9912	0,154187833	-0,81195	1,202689	1mg	Control
AT1G7885(	27	10	248,7202	0,154676696	-0,81058	1,303764	1mg	Control
AT5G3594(	11	3	65,5063	0,154691103	-0,81053	1,3899	DMSO	1mg
AT5G2632(	10	3	78,5175	0,154931525	-0,80986	1,227553	1mg	Control
AT1G7353(	1	1	10,1326	0,155942524	-0,80704	2,548677	Control	DMSO
AT5G1016(	9	5	55,9495	0,156279324	-0,8061	1,131562	Control	DMSO
AT1G0581(	2	1	13,1253	0,156377217	-0,80583	1,506543	Control	DMSO
AT5G2683(	19	12	130,4661	0,157152362	-0,80368	1,328546	1mg	DMSO
AT2G1711(	4	3	21,2704	0,158211635	-0,80076	1,258494	1mg	Control
AT4G0320!	3	1	15,8814	0,159729937	-0,79661	1,294588	Control	1mg
AT4G1676(	13	9	82,6936	0,159943472	-0,79603	1,285082	Control	1mg
AT2G4260(	21	2	175,1586	0,160283707	-0,79511	1,3552	DMSO	1mg
AT3G1878(	31	1	387,6056	0,160699953	-0,79398	1,425234	1mg	Control
AT1G7456(	2	1	11,4799	0,16131402	-0,79233	3,812956	DMSO	Control
AT3G2432(	5	3	28,002	0,161336903	-0,79227	1,562194	DMSO	1mg
ATMG0028	6	2	40,7721	0,161392116	-0,79212	1,871545	1mg	DMSO
AT5G5481(	5	4	34,2067	0,161412255	-0,79206	1,34056	1mg	Control
AT1G8099(	2	1	10,7795	0,161964256	-0,79058	3,355542	1mg	Control
AT5G4575(	7	1	45,018	0,16199777	-0,79049	2,591859	Control	DMSO
AT2G3713(	3	2	24,1873	0,163287156	-0,78705	1,112269	1mg	Control
AT1G1224(	10	6	73,147	0,163541831	-0,78637	1,141933	Control	DMSO
AT1G4886(	11	4	64,9875	0,163557478	-0,78633	1,312996	1mg	Control
AT5G4965(	2	2	10,522	0,163623269	-0,78615	2,348516	DMSO	1mg
AT4G2433(	9	6	57,4138	0,163928019	-0,78535	1,210458	DMSO	Control
AT4G2612(	5	4	30,9409	0,163943003	-0,78531	3,101815	Control	1mg
AT3G5399(	4	2	24,6087	0,164177737	-0,78469	1,641106	Control	DMSO
AT3G6283(	12	3	71,4832	0,164242865	-0,78451	2,556273	Control	1mg
AT4G0137(	4	3	29,1389	0,164605493	-0,78356	1,292546	Control	DMSO

AT3G6165(	1	1	5,2208	0,164927258	-0,78271	3,153794	1mg	Control
AT5G1777(	11	10	98,7893	0,165059197	-0,78236	1,357959	DMSO	1mg
AT1G3123(	5	5	26,7255	0,165597254	-0,78095	1,414224	1mg	Control
AT5G4728(	1	1	5,4184	0,165820632	-0,78036	12,31174	DMSO	1mg
AT5G1706(	4	1	23,979	0,16634707	-0,77898	3,392468	Control	1mg
AT4G1242(	10	5	65,1489	0,166725867	-0,778	1,265902	DMSO	1mg
AT1G1630(	18	2	149,3352	0,166776905	-0,77786	1,52329	DMSO	Control
AT3G6105(	11	7	70,4511	0,168565069	-0,77323	1,509626	Control	1mg
AT4G2768(	4	2	27,9087	0,168710949	-0,77286	1,483036	DMSO	1mg
AT4G1252(	3	2	27,9989	0,168878368	-0,77243	4,471397	DMSO	Control
AT4G2690(	9	6	59,59	0,169068591	-0,77194	1,348395	1mg	Control
AT1G2412(	2	1	16,6838	0,169830114	-0,76999	1,713121	Control	1mg
AT2G1713(	12	5	100,2576	0,170834932	-0,76742	1,559287	1mg	Control
AT3G4889(	1	1	6,1744	0,170943662	-0,76715	2,437069	1mg	Control
AT3G6346(	20	12	141,6086	0,17112292	-0,76669	1,212747	DMSO	1mg
AT1G5633(	10	2	104,705	0,171375498	-0,76605	1,551092	1mg	Control
AT5G1470(	2	2	10,4765	0,171993998	-0,76449	1,332722	Control	DMSO
AT4G1880(	6	1	39,7174	0,172146989	-0,7641	3,6455	1mg	Control
AT2G2786(	14	2	93,5086	0,173498827	-0,7607	2,284104	DMSO	1mg
AT5G0828(	3	2	16,9485	0,173763029	-0,76004	1,416321	Control	DMSO
AT4G1717(	11	7	85,8553	0,174439873	-0,75835	1,127943	DMSO	Control
AT4G1941(	16	1	127,5082	0,174862589	-0,7573	1,351569	1mg	Control
AT1G7237(	16	9	174,9597	0,175104659	-0,7567	1,223317	Control	1mg
AT5G2083(	6	2	46,4825	0,175330657	-0,75614	1,426969	1mg	Control
AT2G4380(	6	3	51,0106	0,175404594	-0,75596	1,266423	1mg	Control
AT5G2095(	22	17	180,2154	0,176067645	-0,75432	1,295323	DMSO	1mg
ATCG0050(	10	6	83,1839	0,176403678	-0,75349	1,417756	DMSO	Control
AT2G2116(	3	2	33,5789	0,176486681	-0,75329	1,623508	DMSO	Control
AT1G4500(	7	1	49,8267	0,176796459	-0,75253	1,354737	DMSO	1mg
AT5G4167(	28	12	242,5779	0,177260913	-0,75139	1,167109	Control	DMSO
AT3G4620(	2	1	11,418	0,177271779	-0,75136	1,954603	Control	1mg
AT4G3017(	21	13	223,6407	0,17733165	-0,75121	1,183817	1mg	Control
AT3G0288(	19	13	134,2215	0,177572145	-0,75063	1,431034	1mg	DMSO
AT1G7857(	19	6	122,2491	0,178273559	-0,74891	1,347487	Control	1mg
AT2G3097(	15	9	115,8172	0,179639423	-0,7456	1,316247	Control	1mg
AT4G1753(	9	4	74,1948	0,17974958	-0,74533	1,452073	1mg	Control
AT1G2931(	7	5	45,4778	0,17978674	-0,74524	1,155384	Control	1mg
AT2G3717(	11	3	139,6488	0,180062518	-0,74458	1,519435	1mg	Control
AT5G0646(	4	2	30,3847	0,180175097	-0,74431	1,24943	1mg	DMSO
AT1G4860(	17	7	137,6656	0,181780833	-0,74045	1,241663	DMSO	1mg
AT1G6709(	1	1	5,3034	0,182400873	-0,73897	1,362473	1mg	Control
AT3G6106(	2	2	11,2084	0,182824794	-0,73796	1,266561	1mg	Control
AT3G5729(	1	1	5,7611	0,183078864	-0,73736	1,99049	Control	1mg
AT4G0185(	27	10	301,8905	0,18308589	-0,73735	1,755799	DMSO	1mg
AT3G0774(	2	1	11,1369	0,183538092	-0,73627	1,152459	Control	1mg
AT5G4413(	2	1	11,5034	0,184304674	-0,73446	2,473354	1mg	Control
AT3G2000(	5	4	34,6852	0,184604803	-0,73376	1,27983	Control	1mg
AT5G1731(	20	1	139,5586	0,185210463	-0,73233	2,198344	DMSO	Control
AT5G6066(	7	2	84,5229	0,186515126	-0,72929	1,618446	1mg	Control
AT3G2719(	6	2	33,1258	0,187274477	-0,72752	1,257073	Control	1mg

AT3G6034(	2	1	15,0585	0,188487123	-0,72472	1,407911	1mg	Control
AT3G1113(	83	12	731,1837	0,188563628	-0,72454	1,33551	DMSO	Control
AT2G3519(	1	1	11,2546	0,190716708	-0,71961	1,506176	1mg	Control
AT4G0161(	5	3	36,9618	0,190954235	-0,71907	1,54945	1mg	Control
AT2G3307(	11	4	95,3114	0,19124461	-0,71841	1,532267	Control	DMSO
AT3G0979(	15	7	111,8252	0,192489504	-0,71559	1,117538	1mg	Control
AT3G5302(	2	1	10,6889	0,192661378	-0,71521	1,722569	1mg	Control
AT1G5198(	23	8	218,8567	0,193310424	-0,71374	1,476417	DMSO	Control
AT5G5292(	19	9	115,963	0,19340819	-0,71353	1,133192	1mg	Control
AT4G3405(	12	9	116,1724	0,194422015	-0,71125	1,449035	Control	1mg
AT4G3092(	13	5	104,015	0,194893499	-0,7102	1,465483	1mg	Control
AT5G6144(	3	1	16,6582	0,195115223	-0,70971	2,1378	1mg	Control
AT3G2644(	2	1	17,1572	0,195901845	-0,70796	10,89197	DMSO	1mg
AT5G1188(	19	2	139,0643	0,196301405	-0,70708	1,712803	DMSO	Control
AT3G2246(	7	1	49,8692	0,196874247	-0,70581	1,266858	DMSO	1mg
AT4G3583(	38	20	371,7425	0,197602109	-0,70421	1,149013	DMSO	Control
AT4G3928(	3	2	16,9489	0,19821595	-0,70286	1,211454	1mg	Control
AT4G3969(	5	5	28,2797	0,198539448	-0,70215	1,436034	DMSO	Control
AT3G2205(	3	3	15,9996	0,198557015	-0,70211	1,25357	Control	1mg
AT4G1680?	2	2	10,7129	0,198653583	-0,7019	4,463966	1mg	Control
AT2G3797(	4	2	22,1633	0,199078906	-0,70097	1,83506	Control	1mg
AT4G2653(	7	3	68,2795	0,199285497	-0,70052	3,235215	DMSO	1mg
AT5G2441(	2	1	11,1306	0,199666189	-0,6997	1,446835	1mg	DMSO
AT2G3629(	3	1	30,1132	0,200269938	-0,69838	2,444388	1mg	Control
AT1G3628(	2	1	10,4334	0,200540316	-0,6978	1,610778	Control	DMSO
AT5G2884(	3	3	23,8904	0,202436626	-0,69371	1,230586	Control	1mg
AT1G0921(	20	9	166,3999	0,202627549	-0,6933	1,365825	DMSO	1mg
AT4G2962(	1	1	11,2138	0,204420107	-0,68948	2,120295	DMSO	Control
AT1G7417(	4	1	28,2784	0,204495158	-0,68932	2,189934	1mg	DMSO
AT2G3931(	7	3	43,465	0,20471591	-0,68885	1,456323	DMSO	Control
AT4G1836(	3	3	17,0723	0,205078637	-0,68808	1,404214	DMSO	Control
AT3G1646(	37	27	461,2427	0,205721333	-0,68672	1,213042	DMSO	1mg
AT5G4897(	3	3	16,5201	0,206074677	-0,68598	1,227636	Control	DMSO
AT5G1092(	8	6	50,055	0,20614693	-0,68582	1,277776	1mg	DMSO
AT1G0918(	6	1	55,6132	0,206429812	-0,68523	5,145969	DMSO	1mg
AT1G0857(	1	1	5,3399	0,206654685	-0,68475	1,905049	Control	1mg
AT5G2096(	13	6	73,3363	0,207522289	-0,68294	1,282236	1mg	Control
AT5G5672(	9	7	69,895	0,208559743	-0,68077	1,639446	Control	1mg
AT4G1272(	2	2	16,6642	0,208737797	-0,6804	1,40275	DMSO	Control
AT5G2738(	11	8	67,902	0,209355909	-0,67911	1,266732	Control	1mg
AT5G2377(	1	1	5,7908	0,209427285	-0,67897	3,484665	DMSO	1mg
AT5G2550(	1	1	5,1107	0,209512329	-0,67879	18,25892	1mg	Control
AT3G4330(	18	12	97,6259	0,210103777	-0,67757	1,298358	DMSO	1mg
AT1G0943(	9	2	68,5199	0,210161621	-0,67745	1,316813	1mg	DMSO
AT1G3191(	2	1	11,9518	0,21027858	-0,6772	2,250084	Control	DMSO
AT5G2063(	5	4	36,5408	0,211017643	-0,67568	1,67348	DMSO	Control
AT1G7994(	2	2	10,9905	0,211738197	-0,6742	1,650614	DMSO	Control
AT2G4579(	9	7	63,4503	0,212057414	-0,67355	1,167982	Control	1mg
AT2G3512(	4	3	25,7292	0,212267863	-0,67312	1,22584	DMSO	Control
AT1G0910(	14	4	93,1832	0,213021255	-0,67158	1,339517	Control	DMSO

AT1G7255(	6	5	39,8065	0,213195002	-0,67122	1,531493	1mg	Control
AT5G6178(	31	16	200,4082	0,213700313	-0,67019	1,191854	1mg	Control
AT5G6356(	3	3	22,0199	0,215150552	-0,66726	1,217205	1mg	Control
AT2G4479(	7	7	99,4433	0,215711821	-0,66613	1,332701	1mg	DMSO
AT1G1954(	1	1	5,1067	0,216573591	-0,66439	3,859079	DMSO	1mg
AT5G4298(	8	4	71,0989	0,218668725	-0,66021	1,266139	1mg	DMSO
AT3G1941(	3	2	16,6998	0,219202467	-0,65915	1,32563	Control	1mg
AT2G3179(	2	2	16,0814	0,22030205	-0,65698	2,86438	DMSO	1mg
AT5G4202(	44	4	488,1387	0,220333445	-0,65692	1,596155	DMSO	Control
AT5G4772(	5	4	28,0739	0,220794875	-0,65601	1,158819	Control	1mg
AT3G2233(	4	2	22,1505	0,221450799	-0,65472	1,747316	Control	1mg
AT3G1134(	1	1	6,0994	0,221512025	-0,6546	1,445051	1mg	DMSO
AT3G4854(	1	1	11,7342	0,22319465	-0,65132	2,239787	1mg	Control
AT5G5668(	2	2	10,2329	0,223435544	-0,65085	1,872093	DMSO	1mg
AT1G5645(	7	6	54,3648	0,224607183	-0,64858	1,715389	Control	1mg
AT1G0323(	20	11	207,2797	0,224702859	-0,64839	1,19531	DMSO	1mg
AT1G7110(	3	3	15,9783	0,224889586	-0,64803	2,523899	Control	1mg
AT5G0496(	3	2	19,0799	0,226220597	-0,64547	1,329327	DMSO	1mg
AT5G3563(	15	9	132,059	0,226575523	-0,64479	1,314564	DMSO	Control
AT5G0101(	3	1	16,6861	0,226619359	-0,6447	1,590936	1mg	DMSO
AT4G2904(	6	1	47,9138	0,228009086	-0,64205	1,288574	1mg	Control
AT5G0250(	40	9	485,7336	0,228010225	-0,64205	1,361702	DMSO	1mg
AT5G4411(	10	3	65,9345	0,228059241	-0,64195	1,490492	DMSO	1mg
AT1G2976(	2	2	15,9976	0,228325058	-0,64145	1,353053	Control	DMSO
AT5G1549(	24	7	188,2819	0,229105386	-0,63996	1,395542	Control	1mg
AT5G6270(	28	6	324,5929	0,229160503	-0,63986	1,30033	DMSO	Control
AT1G4514!	7	5	50,7034	0,229836257	-0,63858	1,335167	1mg	Control
AT4G3563(	17	7	127,1932	0,230741757	-0,63687	1,393667	1mg	DMSO
AT3G0297(	1	1	0	0,233199691	-0,63227	2,485652	DMSO	Control
AT5G2754(	8	4	49,2901	0,233831258	-0,6311	1,5144	1mg	Control
AT5G1342(	23	17	203,9465	0,234249397	-0,63032	1,13054	DMSO	1mg
AT4G0516(	10	8	56,4178	0,234744936	-0,6294	1,243859	1mg	DMSO
AT4G2343(	1	1	5,1765	0,23479334	-0,62931	1,857132	1mg	Control
AT1G7402(	11	9	76,9164	0,235204892	-0,62855	1,227621	1mg	Control
AT1G2253(	38	28	301,9736	0,236123313	-0,62686	1,200763	DMSO	Control
AT1G5422(	6	3	47,4486	0,236814137	-0,62559	1,299968	DMSO	Control
AT3G0494(	7	5	51,6367	0,236845355	-0,62554	1,242204	Control	1mg
AT3G0963(	23	5	229,1804	0,237013479	-0,62523	1,431425	Control	1mg
AT4G3523(	2	1	11,5595	0,237142384	-0,62499	1,818454	1mg	Control
AT1G0902(	2	2	25,3306	0,237144716	-0,62499	1,194266	1mg	Control
AT1G2319(	23	8	191,2579	0,238261756	-0,62295	1,224319	1mg	DMSO
AT3G4872(	2	1	10,8373	0,238283951	-0,62291	3,127415	Control	1mg
AT4G0900(	19	5	154,1334	0,238463773	-0,62258	1,101021	DMSO	Control
AT5G5416(	19	17	185,705	0,238917989	-0,62175	1,192291	DMSO	1mg
AT2G2117(	12	9	110,3024	0,238944119	-0,6217	1,087389	DMSO	1mg
AT5G2089(	23	15	198,1051	0,239072724	-0,62147	1,13182	1mg	Control
AT3G5851(	13	5	95,4056	0,239203644	-0,62123	1,317031	Control	1mg
AT5G6756(	5	2	43,1948	0,239325318	-0,62101	1,757129	DMSO	Control
AT5G1385(	5	1	48,9995	0,239382103	-0,62091	1,193303	Control	DMSO
AT4G0246(	5	2	26,6455	0,242332973	-0,61559	1,864341	DMSO	1mg

AT1G7268(	3	1	15,9167	0,242488254	-0,61531	1,455999	Control	DMSO
AT5G1025(	2	1	10,4577	0,242781431	-0,61478	2,213664	Control	DMSO
AT2G3206(	4	2	30,2105	0,244263695	-0,61214	1,642265	Control	1mg
AT4G1541!	1	1	5,3536	0,244728481	-0,61132	2,466588	1mg	DMSO
AT1G7426(	20	12	128,7152	0,244756741	-0,61127	1,124931	1mg	Control
AT1G3107(	3	2	17,343	0,245041728	-0,61076	1,366328	1mg	DMSO
AT5G5901(	3	2	16,2313	0,245236795	-0,61041	4,018048	1mg	DMSO
AT1G0475(	4	4	23,5792	0,246239472	-0,60864	1,309145	DMSO	1mg
AT1G5581(	3	1	17,2716	0,246396042	-0,60837	1,322931	Control	DMSO
AT2G4180(	16	11	100,2328	0,246422515	-0,60832	1,109337	DMSO	Control
AT1G1808(	21	14	273,5749	0,246548608	-0,6081	1,230731	DMSO	1mg
AT1G0284(	2	1	12,0542	0,246808214	-0,60764	2,513043	DMSO	Control
AT2G4614(	3	2	19,6308	0,246976845	-0,60734	1,134298	Control	DMSO
AT1G6452(	2	1	11,8476	0,247267757	-0,60683	1,460597	Control	1mg
AT1G7031(	8	5	64,6943	0,247330471	-0,60672	1,485368	DMSO	Control
AT4G3851(	27	4	286,9238	0,247332857	-0,60672	1,208685	1mg	Control
AT5G1982(	22	18	150,6031	0,247850227	-0,60581	1,219317	1mg	Control
AT5G2354(	10	8	66,5987	0,248108877	-0,60536	1,154755	1mg	DMSO
AT2G3984(	1	1	5,073	0,248428397	-0,6048	1,822541	DMSO	Control
AT3G0236(	26	14	227,9001	0,248978825	-0,60384	1,127023	Control	DMSO
AT1G5403(	7	5	41,4679	0,249125528	-0,60358	1,281624	Control	DMSO
AT3G1648(	17	5	153,6772	0,249517179	-0,6029	1,085729	1mg	Control
AT1G2820(	1	1	5,7444	0,250006584	-0,60205	1,357639	DMSO	1mg
AT5G0744(	19	13	212,5354	0,250457068	-0,60127	1,119139	Control	1mg
AT4G1989(	2	1	10,6782	0,25060269	-0,60101	1,471208	1mg	Control
AT1G6229(	11	3	74,1046	0,250808248	-0,60066	1,395962	Control	DMSO
AT5G0857(	16	3	113,672	0,252179314	-0,59829	1,827703	DMSO	Control
AT4G3019(	40	8	376,269	0,252504854	-0,59773	1,161009	Control	1mg
AT2G1562(	19	13	130,7813	0,255118148	-0,59326	1,363481	1mg	Control
AT4G3822(	2	2	11,0572	0,255553008	-0,59252	1,418952	1mg	DMSO
AT1G7215(	27	20	219,9585	0,255720643	-0,59223	1,161696	Control	1mg
AT4G0951(	5	1	42,2225	0,255959383	-0,59183	5,199167	DMSO	Control
AT2G4274(	9	6	82,0671	0,256416837	-0,59105	1,789446	1mg	DMSO
AT3G5989(	1	1	5,4569	0,256890176	-0,59025	1,780653	1mg	Control
AT5G5399(	1	1	5,1042	0,257173889	-0,58977	2,081849	Control	DMSO
AT3G5873(	1	1	6,0474	0,257293417	-0,58957	3,455712	DMSO	1mg
AT1G0322(	18	11	269,4021	0,257313016	-0,58954	1,284526	DMSO	Control
AT5G6362(	6	5	34,7444	0,257778822	-0,58875	1,33152	1mg	DMSO
AT3G1323!	2	2	11,2195	0,257879226	-0,58858	2,388167	1mg	Control
AT1G7830(	16	3	129,4616	0,260301355	-0,58452	1,6379	DMSO	Control
AT5G1343(	5	3	46,7884	0,260991956	-0,58337	1,989555	Control	1mg
AT5G6104(	4	1	27,5278	0,261622065	-0,58233	1,82965	1mg	Control
AT4G1246(	2	2	15,7302	0,262427184	-0,58099	1,465997	Control	1mg
AT1G1958(	6	3	54,2642	0,26274083	-0,58047	1,848658	1mg	Control
AT5G2006(	1	1	5,5009	0,262832824	-0,58032	1,29255	DMSO	1mg
AT1G4517(	1	1	5,535	0,262945298	-0,58013	2,02156	Control	1mg
AT2G1790(	1	1	5,9782	0,26314283	-0,57981	1,700269	1mg	Control
AT3G4795(	12	1	74,4527	0,263687385	-0,57891	2,540208	Control	1mg
AT1G0162(	12	3	123,0327	0,264326715	-0,57786	1,279389	Control	DMSO
AT5G0578(	6	2	40,4223	0,265088078	-0,57661	1,268982	DMSO	1mg

AT1G2418(	26	9	216,5913	0,265216067	-0,5764	1,164431	Control	1mg
AT1G5634(	19	7	148,5694	0,265226407	-0,57638	1,720774	DMSO	1mg
AT1G7200(	3	1	18,1933	0,265688701	-0,57563	3,654922	Control	1mg
AT1G2265(	3	1	26,3287	0,266620972	-0,57411	1,192446	1mg	DMSO
AT4G1301(	1	1	5,0851	0,267854674	-0,5721	3,235284	1mg	Control
AT4G3791(	21	12	160,2727	0,268095237	-0,57171	1,149951	DMSO	Control
AT2G2036(	19	14	137,3623	0,268659611	-0,5708	1,34734	Control	DMSO
AT2G2162(	5	5	39,8637	0,269169265	-0,56997	1,630618	Control	DMSO
AT1G8036(	5	3	30,4037	0,26928005	-0,5698	1,267968	Control	1mg
AT1G2428(	11	4	73,5438	0,270138405	-0,56841	3,553072	Control	1mg
AT4G3118(	14	7	89,9041	0,270296539	-0,56816	1,10468	Control	DMSO
ATMG0051	7	6	43,7995	0,27115544	-0,56678	1,191458	DMSO	Control
AT5G1404(	18	15	178,9172	0,271664486	-0,56597	1,158772	DMSO	1mg
AT1G3249(	1	1	5,0193	0,271916116	-0,56557	2,31695	Control	1mg
AT3G0926(	33	20	473,4527	0,272503032	-0,56463	1,493746	DMSO	1mg
AT4G1843(	5	2	33,5307	0,272807368	-0,56414	3,493881	1mg	DMSO
AT4G2293(	1	1	6,4621	0,273165769	-0,56357	2,652557	1mg	DMSO
AT3G2774(	9	6	57,5942	0,273556989	-0,56295	1,204624	Control	1mg
AT3G4939(	1	1	5,0828	0,275008844	-0,56065	3,0086	Control	DMSO
AT3G5352(	10	8	56,7126	0,275047577	-0,56059	1,393729	Control	1mg
AT3G6139(	2	1	10,7495	0,275128569	-0,56046	1,867895	Control	DMSO
AT1G0535(	2	2	10,7315	0,275764545	-0,55946	1,148037	DMSO	Control
AT3G5399(	2	1	12,7066	0,276024179	-0,55905	1,809415	DMSO	Control
AT4G1394(	43	13	578,3476	0,276549086	-0,55823	1,324605	1mg	Control
AT3G0134(	8	6	48,9608	0,276723289	-0,55795	1,425382	1mg	Control
AT3G5522(	2	2	11,7422	0,277883172	-0,55614	1,857973	DMSO	1mg
AT4G2545(	2	1	16,2526	0,278169905	-0,55569	1,234859	DMSO	Control
AT5G0138(	1	1	5,0052	0,278337416	-0,55543	1,638946	Control	1mg
AT3G0884(	3	2	16,6473	0,278587832	-0,55504	1,511185	DMSO	Control
AT3G5705(	3	2	22,574	0,278851769	-0,55463	1,197493	DMSO	1mg
AT1G4930(	6	2	46,1312	0,279400244	-0,55377	1,337204	DMSO	Control
AT5G6437(	2	2	10,9227	0,279403853	-0,55377	1,327055	Control	1mg
AT4G1971(	3	1	16,435	0,279499643	-0,55362	1,280485	DMSO	1mg
AT5G4690(	1	1	5,8644	0,280210176	-0,55252	2,677664	DMSO	1mg
AT5G1944(	6	5	41,2493	0,28194437	-0,54984	1,489378	Control	DMSO
AT5G2628(	18	12	204,7733	0,282416825	-0,54911	1,116471	Control	1mg
AT1G4760(	23	7	262,1855	0,283465335	-0,5475	1,183173	DMSO	Control
AT5G4077(	3	1	17,3039	0,284676667	-0,54565	1,567903	1mg	DMSO
AT5G3740(	1	1	11,7034	0,285219713	-0,54482	1,540785	1mg	Control
AT4G3486(	2	1	18,1837	0,285817741	-0,54391	1,59497	1mg	DMSO
AT3G4885(	6	4	41,6208	0,287007063	-0,54211	1,32077	DMSO	Control
AT4G0057(	8	7	64,8175	0,287370671	-0,54156	1,406713	DMSO	Control
AT5G4076(	14	8	86,7021	0,287723695	-0,54102	1,312884	1mg	Control
AT3G5214(	1	1	5,2603	0,289501118	-0,53835	2,168447	1mg	Control
AT5G1110(	2	2	16,0074	0,289638223	-0,53814	1,879006	Control	1mg
AT5G1659(	9	5	57,9555	0,290238325	-0,53725	1,324356	1mg	Control
AT1G5324(	22	11	246,6741	0,290641273	-0,53664	1,290478	1mg	Control
AT4G3675(	6	5	36,6506	0,290856588	-0,53632	1,236721	Control	1mg
AT5G2265(	6	4	50,3436	0,291156429	-0,53587	1,570959	DMSO	1mg
AT2G3504(	2	2	11,0416	0,292251188	-0,53424	1,758228	Control	1mg

AT5G0474(	6	3	33,3795	0,293981145	-0,53168	1,531815	Control	1mg
AT3G2063(	3	1	16,0052	0,294386922	-0,53108	1,980167	Control	1mg
AT5G3932(	27	7	225,753	0,294999643	-0,53018	1,349623	1mg	Control
AT5G6668(	15	12	94,4184	0,295210453	-0,52987	1,193378	DMSO	Control
AT2G0200(	14	1	119,9059	0,297408159	-0,52665	1,16182	DMSO	Control
AT5G4383(	12	10	94,9746	0,29781797	-0,52605	1,332946	DMSO	1mg
AT3G1806(	9	2	68,0609	0,297977394	-0,52582	1,871254	1mg	Control
AT3G2676(	1	1	5,4228	0,298047791	-0,52571	1,400877	Control	1mg
AT5G2575(	2	1	10,8952	0,298566672	-0,52496	1,206692	DMSO	1mg
AT3G1739(	30	13	320,921	0,299093975	-0,52419	1,358998	DMSO	1mg
AT1G3069(	9	3	51,9139	0,299942175	-0,52296	1,518683	1mg	Control
AT1G0347(	4	3	27,8549	0,30001937	-0,52285	1,338747	1mg	DMSO
AT4G2455(	2	2	10,7589	0,300065172	-0,52278	1,463091	1mg	Control
AT3G1647(	13	9	114,0373	0,300075274	-0,52277	1,175931	DMSO	1mg
AT5G1349(	16	3	167,0906	0,300319411	-0,52242	1,354601	1mg	DMSO
AT3G5375(	24	3	261,2107	0,300512481	-0,52214	1,308708	Control	DMSO
AT3G6203(	5	2	28,8056	0,300626367	-0,52197	1,15968	DMSO	1mg
AT2G4049(	2	1	16,0057	0,301280108	-0,52103	1,728474	DMSO	Control
AT5G0527(	5	4	34,5061	0,301731058	-0,52038	1,268747	DMSO	1mg
AT1G7410(	3	2	17,7378	0,30287562	-0,51874	1,459264	DMSO	Control
AT3G1641(	27	7	293,9262	0,302896227	-0,51871	1,335648	DMSO	1mg
AT2G1695(	4	4	21,6407	0,30299218	-0,51857	1,360321	Control	1mg
AT5G2325(	10	4	105,2347	0,303880577	-0,5173	1,74799	DMSO	Control
AT2G2608(	23	5	178,0308	0,307183483	-0,5126	1,396197	1mg	Control
AT4G2510(	1	1	7,9507	0,307630868	-0,51197	2,226875	DMSO	1mg
AT2G1412(	9	3	51,2205	0,30840691	-0,51088	3,504301	DMSO	1mg
AT3G4873(	4	1	21,2051	0,308555393	-0,51067	2,154209	Control	DMSO
AT1G2307(	2	1	16,812	0,308827586	-0,51028	12,26886	Control	DMSO
AT5G4593(	3	1	16,1978	0,309284753	-0,50964	1,785193	1mg	Control
AT3G5464(	3	3	17,6174	0,309397044	-0,50948	2,815855	DMSO	1mg
AT5G1352(	4	4	22,5073	0,309854822	-0,50884	1,342436	DMSO	1mg
AT1G6179(	5	3	34,3112	0,311584004	-0,50642	1,654997	Control	1mg
AT2G0152(	12	7	134,3918	0,311841333	-0,50607	1,811695	Control	1mg
AT2G2099(	7	1	47,1081	0,313091374	-0,50433	2,464425	DMSO	1mg
AT3G2381(	38	6	438,2166	0,313539482	-0,50371	1,683981	DMSO	1mg
AT1G2853(	3	1	16,6924	0,314841373	-0,50191	1,829832	1mg	Control
AT5G6368(	16	2	108,5423	0,315815626	-0,50057	2,9941	DMSO	1mg
AT4G0904(	1	1	5,1155	0,319108972	-0,49606	2,359337	Control	1mg
AT3G5948(	13	6	116,4298	0,319467364	-0,49557	1,248419	Control	DMSO
AT5G6227(	2	1	11,2725	0,321283355	-0,49311	1,735906	DMSO	Control
AT1G2786(	3	1	17,0778	0,321608185	-0,49267	1,365246	DMSO	1mg
AT3G2871(	10	6	65,2795	0,321990066	-0,49216	1,676214	DMSO	Control
AT5G1247(	3	2	16,4633	0,322629669	-0,4913	1,335374	DMSO	1mg
AT3G0803(	6	4	35,8248	0,322969041	-0,49084	1,345497	DMSO	Control
AT5G4770(	1	1	6,799	0,32391633	-0,48957	1,163892	1mg	Control
AT4G0245(	11	9	108,3136	0,324863557	-0,4883	1,294178	DMSO	Control
AT3G1291(	38	12	406,0124	0,325035918	-0,48807	1,230185	DMSO	Control
AT1G1807(	3	2	18,2734	0,32541548	-0,48756	1,129452	Control	DMSO
AT1G0963(	6	1	38,7341	0,326341741	-0,48633	2,306396	1mg	DMSO
AT5G0944(	1	1	5,1076	0,327635946	-0,48461	1,595665	Control	1mg

AT2G0484[illegible]	3	2	27,2748	0,327658354	-0,48458	1,844317	1mg	Control
AT3G0710[illegible]	13	12	82,9757	0,327833752	-0,48435	1,168738	DMSO	1mg
AT2G3087[illegible]	12	8	107,2938	0,328156979	-0,48392	1,191735	Control	1mg
AT5G6651[illegible]	11	5	99,4112	0,328639805	-0,48328	1,174505	1mg	Control
AT4G2036[illegible]	17	11	146,1045	0,328656364	-0,48326	1,14183	Control	1mg
AT4G3640[illegible]	3	1	16,3551	0,329787943	-0,48177	1,780904	Control	1mg
AT5G4510[illegible]	2	1	10,7433	0,330087973	-0,48137	3,310846	Control	1mg
AT1G5670[illegible]	2	2	11,6568	0,330112777	-0,48134	1,352709	Control	1mg
AT5G5939[illegible]	3	3	15,4838	0,330507535	-0,48082	1,954424	1mg	DMSO
AT3G4834[illegible]	5	3	27,4861	0,331625836	-0,47935	1,16354	DMSO	1mg
AT1G1539[illegible]	1	1	5,7494	0,331920333	-0,47897	1,226686	1mg	Control
AT1G2990[illegible]	24	16	152,3175	0,333399067	-0,47704	1,211089	Control	1mg
AT4G0282[illegible]	2	1	10,3709	0,333442867	-0,47698	2,669966	1mg	DMSO
AT1G0388[illegible]	4	3	22,8962	0,334120502	-0,4761	23,8828	DMSO	Control
AT5G6676[illegible]	22	7	181,0794	0,334492413	-0,47561	1,149325	1mg	Control
AT3G5562[illegible]	3	2	19,7652	0,334593846	-0,47548	1,269947	1mg	Control
AT3G5541[illegible]	23	14	173,6009	0,336138182	-0,47348	1,138119	1mg	DMSO
AT4G3926[illegible]	8	2	57,0341	0,336819613	-0,4726	1,265626	1mg	Control
AT5G5092[illegible]	14	4	83,1631	0,33683894	-0,47258	1,088175	1mg	Control
AT1G6531[illegible]	5	1	28,6764	0,336914614	-0,47248	3,114306	Control	1mg
AT2G1315[illegible]	3	3	22,651	0,337106267	-0,47223	1,340423	1mg	DMSO
AT4G2310[illegible]	6	5	33,1219	0,337465015	-0,47177	1,281056	1mg	DMSO
AT4G1138[illegible]	18	6	130,1841	0,337474481	-0,47176	1,131173	DMSO	Control
AT2G1264[illegible]	2	2	11,3488	0,337578743	-0,47162	1,703819	1mg	Control
AT3G2925[illegible]	10	7	83,3322	0,338157892	-0,47088	1,221901	1mg	Control
AT2G3181[illegible]	5	4	33,2599	0,338566704	-0,47036	1,567835	Control	DMSO
AT4G2480[illegible]	2	1	11,2395	0,338605302	-0,47031	1,348026	1mg	Control
AT5G2072[illegible]	9	7	64,1685	0,338611124	-0,4703	1,189484	1mg	DMSO
AT1G1913[illegible]	2	1	11,2036	0,338817435	-0,47003	1,537366	Control	DMSO
AT5G4306[illegible]	12	9	86,9658	0,34003811	-0,46847	1,105396	DMSO	Control
AT3G0325[illegible]	24	8	208,0779	0,341047582	-0,46719	1,286014	Control	DMSO
AT3G0412[illegible]	29	2	411,6613	0,342037121	-0,46593	1,663723	DMSO	Control
AT1G1734[illegible]	2	1	10,4383	0,343911155	-0,46355	3,079881	Control	DMSO
AT5G3957[illegible]	5	4	32,8246	0,344221684	-0,46316	1,709987	DMSO	Control
AT1G2703[illegible]	3	3	17,6925	0,345323865	-0,46177	1,444309	DMSO	Control
AT1G5549[illegible]	20	2	135,8084	0,345365194	-0,46172	3,040597	1mg	Control
AT2G3011[illegible]	24	17	166,8671	0,345576559	-0,46146	1,192603	DMSO	1mg
AT5G2440[illegible]	2	2	11,053	0,346835166	-0,45988	1,351056	Control	1mg
AT5G6253[illegible]	12	8	77,4188	0,348772007	-0,45746	1,127174	1mg	DMSO
AT3G2079[illegible]	2	2	10,659	0,348877762	-0,45733	1,487999	Control	DMSO
AT3G4319[illegible]	2	1	10,951	0,349056048	-0,4571	2,141906	1mg	Control
AT3G1695[illegible]	12	5	82,3912	0,349648738	-0,45637	1,640241	DMSO	1mg
AT5G3536[illegible]	14	10	104,3345	0,350226742	-0,45565	1,128681	DMSO	1mg
AT4G2474[illegible]	1	1	5,7011	0,350424322	-0,45541	2,229576	DMSO	Control
AT3G1643[illegible]	15	4	154,8139	0,351000218	-0,45469	1,27478	1mg	DMSO
AT1G0372[illegible]	1	1	5,2059	0,351549864	-0,45401	1,342276	DMSO	1mg
AT1G5383[illegible]	9	2	67,2979	0,351628306	-0,45392	1,12611	Control	1mg
AT3G5875[illegible]	4	4	20,9816	0,352479321	-0,45287	1,100636	Control	DMSO
AT3G2450[illegible]	22	15	154,9424	0,352571785	-0,45275	1,11124	Control	1mg
AT3G0142[illegible]	22	15	147,9479	0,3530812	-0,45213	1,127715	Control	DMSO

AT1G2236(	6	5	34,4695	0,354764676	-0,45006	1,368593	DMSO	1mg
AT2G2420(	22	15	204,4139	0,354791875	-0,45003	1,126901	DMSO	Control
AT3G4683(	7	1	47,2172	0,354965223	-0,44981	2,979432	1mg	Control
AT3G4563(	2	1	10,7424	0,355482252	-0,44918	1,998565	1mg	Control
AT1G0417(	18	14	147,8858	0,356378448	-0,44809	1,208074	DMSO	1mg
AT1G6281(	3	3	16,7999	0,357396881	-0,44685	1,306014	1mg	Control
AT2G4711(	9	3	103,535	0,357653718	-0,44654	1,461021	1mg	Control
AT1G6146(	2	2	10,6443	0,359151721	-0,44472	1,425055	DMSO	1mg
AT1G0109(	15	10	97,4682	0,361447012	-0,44196	1,15336	1mg	Control
AT2G1811(	10	4	97,6119	0,361706467	-0,44164	1,167381	DMSO	1mg
AT4G1505:	1	1	5,3205	0,361942831	-0,44136	1,73842	1mg	DMSO
AT1G5171(	3	2	27,4051	0,363001224	-0,44009	2,250534	Control	1mg
AT4G1539(	15	10	117,3997	0,363300798	-0,43973	1,104487	1mg	DMSO
AT3G0220(	8	4	50,0886	0,36335212	-0,43967	1,28332	1mg	Control
AT1G4892(	6	5	41,3697	0,363380256	-0,43964	1,377677	DMSO	Control
AT1G2031(	1	1	5,0765	0,363471411	-0,43953	1,574617	DMSO	1mg
AT1G7041(	8	6	62,7202	0,365049438	-0,43765	1,119955	1mg	DMSO
AT3G0768(	4	2	23,3206	0,365862184	-0,43668	1,480513	DMSO	Control
AT1G1820(	4	1	30,9252	0,367737349	-0,43446	1,450259	Control	1mg
AT1G6123(	3	1	21,9107	0,367834804	-0,43435	1,149064	Control	1mg
AT3G2738(	2	1	11,0813	0,367880898	-0,43429	1,831103	1mg	Control
AT1G6266(	18	13	164,0136	0,368706473	-0,43332	1,074979	Control	1mg
AT3G1386(	18	12	124,4181	0,368883893	-0,43311	1,148759	DMSO	Control
AT3G2137(	10	4	92,1633	0,369476075	-0,43241	1,417776	Control	1mg
AT4G1265(	4	3	30,1346	0,369617114	-0,43225	1,254816	DMSO	1mg
AT3G1945(	20	17	206,5703	0,370023911	-0,43177	1,204605	DMSO	Control
AT4G2970(	2	1	10,4844	0,370294829	-0,43145	3,382787	1mg	Control
AT5G6386(	1	1	6,2503	0,370496927	-0,43122	1,569168	1mg	Control
AT3G1939(	8	4	50,9827	0,370833111	-0,43082	1,195796	1mg	Control
AT5G2002(	9	7	82,9364	0,370848503	-0,4308	1,280326	1mg	DMSO
AT5G5909(	21	13	177,5816	0,3710707	-0,43054	1,167792	DMSO	1mg
AT4G1182(	14	9	88,7506	0,371717536	-0,42979	1,113107	DMSO	1mg
AT4G3973(	6	4	68,6569	0,372396728	-0,42899	1,107845	1mg	Control
AT1G5521(	4	3	23,8069	0,373600248	-0,42759	1,228409	Control	1mg
AT5G0459(	14	13	102,7819	0,374123343	-0,42699	1,086233	1mg	Control
AT1G2410(	1	1	5,5173	0,374852618	-0,42614	2,063122	DMSO	Control
AT5G6073(	1	1	12,4274	0,375646011	-0,42522	1,828487	Control	1mg
AT5G4296(	3	3	16,3458	0,37641141	-0,42434	1,13594	Control	1mg
AT1G5184(	5	4	37,1237	0,376836645	-0,42385	1,421578	Control	1mg
AT4G3586(	7	2	41,1044	0,37731963	-0,42329	1,565335	1mg	DMSO
AT3G4911(	8	1	81,7799	0,378193048	-0,42229	2,326406	1mg	Control
AT5G1480(	7	6	40,3555	0,378686184	-0,42172	1,208051	DMSO	1mg
AT3G1277(	2	1	10,4459	0,378816074	-0,42157	2,500896	DMSO	Control
AT4G1403(	16	11	116,2859	0,379032844	-0,42132	1,153601	Control	DMSO
ATMG0007	8	6	46,9589	0,379421032	-0,42088	1,507674	Control	DMSO
AT5G3688(	13	8	85,2526	0,379440092	-0,42086	1,399088	Control	1mg
AT5G2386(	26	3	306,8169	0,379714768	-0,42054	1,448022	DMSO	1mg
AT5G6195(	3	2	21,185	0,380251564	-0,41993	1,251514	1mg	DMSO
AT2G4382(	3	2	16,5216	0,380430156	-0,41973	2,264236	DMSO	Control
AT1G6522(	6	2	34,373	0,38067857	-0,41944	1,349719	1mg	Control

AT3G6075(	27	16	257,183	0,38071539	-0,4194	1,290568	DMSO	Control
AT2G4713(	5	2	48,8015	0,382034961	-0,4179	1,519177	DMSO	1mg
AT5G4629(	18	16	138,4718	0,382461361	-0,41741	1,30368	DMSO	1mg
AT5G5635(	21	5	139,9734	0,385191834	-0,41432	1,473371	DMSO	1mg
AT2G3207(	2	2	11,457	0,386172615	-0,41322	1,440381	Control	1mg
AT5G2098(	16	4	165,8742	0,386585872	-0,41275	1,141766	Control	DMSO
AT5G2671(	6	4	32,5894	0,387705066	-0,4115	1,309456	Control	1mg
AT2G4406(	14	10	130,4155	0,387743981	-0,41145	1,143112	DMSO	Control
AT3G2672(	11	9	62,4331	0,389129203	-0,40991	1,119605	Control	DMSO
AT1G5960(	3	1	20,3354	0,391735311	-0,40701	1,85816	1mg	DMSO
AT2G3773(	3	3	16,3437	0,391916949	-0,40681	1,444759	DMSO	Control
AT2G0584(	10	1	59,1767	0,392780517	-0,40585	1,410311	1mg	Control
AT2G3459(	8	2	55,4318	0,393323812	-0,40525	1,316482	DMSO	Control
AT3G5631(	9	7	67,6033	0,393926733	-0,40458	1,126142	Control	DMSO
AT1G5136(	1	1	11,3862	0,394101975	-0,40439	1,799321	1mg	DMSO
AT1G2228(	1	1	6,0008	0,394309965	-0,40416	1,722988	Control	1mg
AT5G3853(	1	1	5,1504	0,394673603	-0,40376	2,59827	Control	1mg
AT1G1191(	24	14	228,2708	0,395154961	-0,40323	1,307684	DMSO	Control
AT5G5366(	2	1	14,325	0,395228259	-0,40315	2,447248	1mg	DMSO
AT2G2751(	5	4	44,2153	0,395756497	-0,40257	1,257113	Control	1mg
AT5G3893(	2	1	22,6079	0,395983992	-0,40232	1,940999	1mg	Control
AT1G1634(	3	1	16,9906	0,396166169	-0,40212	1,467419	DMSO	Control
AT3G1639(	12	5	115,2021	0,396267892	-0,40201	1,521915	Control	1mg
AT3G1229(	11	9	73,3872	0,39674073	-0,40149	1,257706	DMSO	Control
AT2G3653(	31	24	398,4328	0,397166949	-0,40103	1,183861	DMSO	Control
AT1G5961(	7	3	37,2034	0,398211576	-0,39989	1,230087	Control	1mg
AT1G3379(	7	1	43,2756	0,398268679	-0,39982	1,71508	1mg	DMSO
AT5G6391(	1	1	5,5116	0,399853848	-0,3981	1,902251	DMSO	Control
AT2G1798(	3	1	16,1347	0,400182383	-0,39774	1,425044	1mg	Control
AT1G2033(	2	1	11,5052	0,400679956	-0,3972	1,52996	DMSO	1mg
AT5G6179(	4	3	29,5325	0,401062124	-0,39679	1,506557	1mg	Control
AT1G7496(	5	4	28,7093	0,401224018	-0,39661	1,623453	DMSO	1mg
AT3G1211(	24	1	278,8251	0,401245168	-0,39659	1,245554	1mg	DMSO
AT1G2745(	9	1	75,6201	0,40261333	-0,39511	1,740226	DMSO	1mg
AT4G1970(	1	1	5,3849	0,403644152	-0,394	1,229689	Control	1mg
AT2G4098(	1	1	12,5672	0,405186705	-0,39234	1,514908	DMSO	Control
AT3G1125(	9	1	97,547	0,406151343	-0,39131	1,640475	1mg	Control
AT3G5756(	5	4	28,1949	0,406608011	-0,39082	1,279984	1mg	Control
AT2G3646(	19	3	233,6664	0,406712604	-0,39071	1,187721	1mg	DMSO
AT1G6698(	8	4	52,9033	0,408441309	-0,38887	1,20601	Control	1mg
AT4G2758!	2	1	10,8874	0,409041314	-0,38823	1,431461	1mg	Control
AT3G1347(	21	1	149,6528	0,410457085	-0,38673	1,857694	Control	DMSO
AT5G1651(	10	7	65,6979	0,411271947	-0,38587	1,706924	Control	DMSO
AT1G4726(	15	10	129,3772	0,411834418	-0,38528	1,151177	1mg	Control
AT1G0250(	26	6	279,5075	0,413093784	-0,38395	1,502734	DMSO	Control
AT1G0552(	5	2	36,1578	0,415009683	-0,38194	1,567955	Control	1mg
AT4G2691(	14	5	103,2298	0,416282705	-0,38061	2,00833	1mg	Control
AT1G1845(	7	5	54,622	0,416710339	-0,38017	1,209329	1mg	Control
AT1G7275(	2	2	11,8236	0,418322453	-0,37849	1,238798	DMSO	Control
AT5G2008(	3	1	17,9598	0,419883277	-0,37687	2,286258	DMSO	Control

AT1G0629(	9	3	59,3331	0,42119021	-0,37552	1,234351	1mg	Control
AT1G7595(	5	3	45,1044	0,421653788	-0,37504	1,365879	1mg	Control
AT5G6612(	1	1	6,0913	0,422291472	-0,37439	1,418583	1mg	Control
AT3G0484(	17	7	145,5993	0,422732884	-0,37393	1,227127	DMSO	Control
AT1G3359(	5	3	33,6936	0,422763779	-0,3739	1,335171	Control	DMSO
AT1G0737(	12	5	73,8332	0,423749003	-0,37289	1,8336	DMSO	Control
AT1G7049(	12	8	125,2727	0,424919489	-0,37169	1,276294	DMSO	Control
AT1G6926(	2	1	23,855	0,425094561	-0,37151	1,590765	1mg	DMSO
AT3G0276(	19	8	130,5101	0,425571422	-0,37103	1,165714	Control	DMSO
AT3G1747(	3	1	22,2116	0,426190474	-0,3704	1,432899	1mg	DMSO
AT2G4184(	13	4	109,9935	0,427129034	-0,36944	1,854262	DMSO	Control
AT5G6642(	26	23	181,902	0,427771062	-0,36879	1,07404	1mg	Control
AT2G3005(	11	8	74,5393	0,428701935	-0,36784	1,187164	DMSO	1mg
AT2G3484(	3	2	15,591	0,430195371	-0,36633	1,285186	DMSO	1mg
AT5G6571(	7	2	38,8411	0,432142526	-0,36437	1,222132	Control	1mg
AT1G0978(	30	16	309,9628	0,432770096	-0,36374	1,191676	DMSO	Control
AT3G0869(	1	1	16,503	0,433144937	-0,36337	1,57055	DMSO	Control
AT4G1129(	10	9	89,4937	0,43337235	-0,36314	1,181844	1mg	Control
AT1G5020(	25	17	173,6778	0,43358214	-0,36293	1,094386	Control	DMSO
AT1G3058(	11	11	89,4649	0,434129435	-0,36238	1,100911	DMSO	1mg
AT3G0930(	9	5	58,1717	0,434659754	-0,36185	1,257602	Control	DMSO
AT3G2039(	11	7	83,4324	0,435568574	-0,36094	1,178426	1mg	DMSO
AT3G5749(	8	3	75,8489	0,436171232	-0,36034	1,268202	DMSO	1mg
AT5G5157(	1	1	5,3383	0,437242236	-0,35928	2,05032	DMSO	1mg
AT1G6598(	8	5	86,0739	0,437706017	-0,35882	1,224708	Control	1mg
AT1G1071(	4	2	21,2046	0,437852557	-0,35867	1,544691	DMSO	1mg
AT5G4779(	3	1	15,5011	0,439773653	-0,35677	1,231695	DMSO	1mg
AT1G7400(	1	1	5,5713	0,440122626	-0,35643	1,782932	Control	DMSO
AT2G2063(	6	2	37,5575	0,440730615	-0,35583	1,209774	1mg	Control
AT5G6469(	1	1	5,0416	0,44365158	-0,35296	1,642309	Control	1mg
AT4G1119(	3	2	18,3403	0,443835039	-0,35278	1,884466	DMSO	1mg
AT5G5203(	1	1	5,5601	0,444246881	-0,35238	1,265877	Control	DMSO
AT4G0508(	1	1	5,0178	0,444729214	-0,3519	1,955101	1mg	DMSO
AT3G1819(	29	17	236,4528	0,445024205	-0,35162	1,185364	1mg	Control
AT3G0477(	10	1	95,3505	0,445801527	-0,35086	1,311774	DMSO	1mg
AT5G4396(	2	2	12,1759	0,446923324	-0,34977	1,135315	Control	DMSO
AT4G2851(	4	1	24,3552	0,446924272	-0,34977	1,40204	DMSO	1mg
AT1G5827(	12	8	93,7663	0,449216466	-0,34754	1,109801	Control	DMSO
AT2G4530(	7	3	43,754	0,450744096	-0,34607	1,320612	1mg	Control
AT3G5059(	5	2	27,7379	0,450991147	-0,34583	1,508757	1mg	Control
AT5G5687(	12	7	68,3174	0,453221882	-0,34369	1,17888	DMSO	1mg
AT1G7953(	22	11	193,0227	0,453730254	-0,3432	1,264278	DMSO	Control
AT3G2177(	6	3	37,3796	0,454173306	-0,34278	1,531798	DMSO	1mg
AT1G2549(	10	4	66,7249	0,454233388	-0,34272	1,405199	Control	1mg
AT1G0792(	25	21	317,6773	0,454518505	-0,34245	1,321311	DMSO	1mg
AT1G4803(	22	8	174,3382	0,455194914	-0,3418	1,19583	DMSO	Control
AT2G3321(	29	10	243,8013	0,456001628	-0,34103	1,052638	Control	DMSO
AT3G5496(	17	14	121,7537	0,456253034	-0,34079	1,18148	DMSO	Control
AT3G5155(	2	1	11,1701	0,457036049	-0,34005	1,854639	Control	1mg
AT3G1800(	13	4	105,8953	0,45721856	-0,33988	1,751709	Control	1mg

AT4G2319(	1	1	5,991	0,457678785	-0,33944	2,134302	DMSO	Control
AT5G5871(	6	5	35,3107	0,457920533	-0,33921	1,219828	Control	DMSO
AT1G2713(	8	5	66,8036	0,458572367	-0,33859	1,150289	Control	DMSO
AT3G0378(	39	13	531,0833	0,458684026	-0,33849	1,149545	DMSO	Control
AT3G0658(	8	5	44,1775	0,458842305	-0,33834	1,158901	Control	1mg
AT2G1255(	5	2	31,0935	0,459531309	-0,33768	1,52267	1mg	Control
AT2G4571(	3	3	22,3088	0,460316848	-0,33694	1,353378	DMSO	Control
AT1G2813(	6	2	33,8969	0,460506619	-0,33676	1,359445	DMSO	1mg
AT3G1406;	16	12	116,815	0,461096174	-0,33621	1,248549	1mg	Control
AT4G3411(	7	4	46,5155	0,46133235	-0,33599	1,117638	DMSO	Control
AT5G5247(	11	2	73,7255	0,461595117	-0,33574	1,370856	DMSO	1mg
AT3G2005(	12	11	83,4483	0,462719686	-0,33468	3,47611	DMSO	Control
AT1G5611(	11	6	64,5738	0,463003123	-0,33442	1,294238	DMSO	Control
AT4G1728(	2	2	10,4515	0,464892465	-0,33265	1,343261	DMSO	1mg
AT5G3751(	24	16	173,3009	0,464958359	-0,33259	1,179157	DMSO	Control
AT2G2545(	7	6	51,5733	0,465935491	-0,33167	1,196323	DMSO	1mg
AT4G0187(	2	2	10,9076	0,466153333	-0,33147	1,535598	Control	1mg
AT4G2969(	5	4	41,254	0,466223139	-0,33141	1,556414	1mg	DMSO
AT3G4988(	2	1	10,8871	0,466921918	-0,33076	1,585639	1mg	Control
AT1G3051(	3	2	16,5475	0,467939257	-0,32981	1,351767	Control	1mg
AT2G1382(	1	1	6,0985	0,468784895	-0,32903	1,103545	Control	1mg
AT1G0114(	4	2	26,1852	0,469286782	-0,32856	2,795523	1mg	Control
AT5G6457(	1	1	5,0238	0,47061901	-0,32733	1,452613	Control	1mg
AT4G2422(	9	5	63,5212	0,470658166	-0,32729	1,217857	1mg	DMSO
AT3G5447(	12	12	103,1702	0,471105173	-0,32688	1,21567	DMSO	Control
AT1G8095(	2	2	11,3231	0,471945564	-0,32611	1,465632	Control	1mg
AT5G4528(	12	8	107,0003	0,472187624	-0,32589	1,501275	1mg	DMSO
AT3G5649(	1	1	6,6212	0,472332426	-0,32575	1,418412	1mg	DMSO
AT5G4208(	11	5	61,0843	0,472485862	-0,32561	1,260988	DMSO	1mg
AT3G0488(	4	2	22,3208	0,472876151	-0,32525	1,355763	Control	DMSO
AT4G2329(	2	1	11,1166	0,473836501	-0,32437	1,170713	DMSO	Control
AT1G1277!	2	1	10,7151	0,475653823	-0,32271	1,221726	1mg	Control
AT3G2098(	6	3	37,7739	0,476059145	-0,32234	1,26339	1mg	Control
AT4G3999(	4	1	30,3004	0,476589346	-0,32186	1,3133	1mg	DMSO
AT5G6569(	13	6	75,2231	0,476618428	-0,32183	1,180206	1mg	DMSO
AT2G2868(	7	5	40,6756	0,476818967	-0,32165	1,449698	DMSO	Control
AT2G0114(	12	7	116,6729	0,476886951	-0,32158	1,28702	Control	1mg
AT5G2206(	1	1	5,3909	0,477868008	-0,32069	2,459911	Control	DMSO
AT1G0622(	9	3	49,3767	0,479147551	-0,31953	1,612385	DMSO	Control
AT1G0934(	1	1	5,2832	0,481431406	-0,31747	1,206614	1mg	DMSO
AT3G4868(	8	1	50,0413	0,482872255	-0,31617	1,950659	1mg	Control
AT2G3769(	6	2	33,9056	0,483484526	-0,31562	1,276502	1mg	Control
AT1G2069:	1	1	5,4051	0,48431204	-0,31487	2,397003	Control	1mg
AT3G1645(	17	13	177,6693	0,484601423	-0,31462	1,111032	Control	DMSO
AT1G5257(	19	4	150,1681	0,484901673	-0,31435	1,152556	DMSO	1mg
AT3G4431(	7	2	53,1371	0,485793051	-0,31355	1,188579	1mg	DMSO
AT2G3688(	24	12	275,6017	0,490199298	-0,30963	1,136895	Control	1mg
AT5G1156(	18	13	112,0379	0,490954697	-0,30896	1,091207	1mg	Control
AT5G2330(	2	2	10,9198	0,492112443	-0,30794	3,018195	DMSO	Control
AT5G6575(	14	3	99,098	0,493023165	-0,30713	1,316492	1mg	DMSO

AT5G5937(	20	1	227,5399	0,493468258	-0,30674	1,590235	Control	1mg
AT5G0423(	3	3	16,911	0,493679895	-0,30655	1,104642	Control	1mg
AT3G4800(	15	11	114,8647	0,493692669	-0,30654	1,124532	Control	DMSO
AT5G1036(	4	1	23,687	0,494183758	-0,30611	6,067781	DMSO	Control
AT3G1194(	14	12	128,3682	0,494649714	-0,3057	1,143327	DMSO	Control
AT3G1610(	5	2	32,0386	0,497596307	-0,30312	1,164432	Control	1mg
AT4G1045(	10	3	75,1082	0,498390384	-0,30243	1,412127	1mg	DMSO
AT3G0405(	11	4	67,0025	0,499312167	-0,30163	1,426921	DMSO	1mg
AT1G1552(	2	1	11,8125	0,499345842	-0,3016	1,142115	1mg	Control
AT5G6557(	3	1	22,3211	0,500564456	-0,30054	1,559132	DMSO	Control
AT5G5197(	17	15	125,4191	0,500719885	-0,30041	1,167092	Control	DMSO
AT3G5440(	3	3	17,9553	0,501217329	-0,29997	1,435399	DMSO	1mg
AT4G2697(	39	29	329,6585	0,502364144	-0,29898	1,172489	DMSO	1mg
AT4G2235(	4	3	21,37	0,502957045	-0,29847	1,164878	DMSO	1mg
AT4G2603(	1	1	5,1973	0,503109091	-0,29834	1,598782	DMSO	Control
AT5G1609(	1	1	6,0858	0,503715738	-0,29781	1,19439	DMSO	Control
AT5G5915(	7	1	46,1639	0,505152531	-0,29658	2,274395	Control	DMSO
AT5G1955(	27	17	249,6358	0,506055316	-0,2958	1,190857	DMSO	Control
AT2G4296(	1	1	11,977	0,506580158	-0,29535	1,137535	1mg	DMSO
AT3G5293(	28	14	427,3198	0,507072849	-0,29493	1,207879	1mg	Control
AT4G1496(	22	1	278,3882	0,507594074	-0,29448	1,192872	1mg	Control
AT1G1550(	3	2	17,078	0,507696305	-0,2944	1,286978	DMSO	1mg
AT1G0427(	3	2	20,9908	0,507792043	-0,29431	1,997487	DMSO	Control
AT3G2296(	19	12	124,3921	0,509516415	-0,29284	1,19164	DMSO	Control
AT2G0522(	1	1	5,7464	0,509972181	-0,29245	1,402205	DMSO	1mg
AT3G5992(	18	10	159,7507	0,510235082	-0,29223	1,218843	DMSO	Control
AT1G2702(	1	1	5,2798	0,511276708	-0,29134	1,482944	1mg	DMSO
AT5G1204(	3	1	16,5487	0,512353281	-0,29043	1,349393	Control	1mg
AT1G5898:	8	2	68,905	0,512579026	-0,29024	1,737268	DMSO	Control
AT3G4205(	18	12	113,451	0,513291391	-0,28964	1,104838	1mg	Control
AT5G5603(	51	3	545,6746	0,51438563	-0,28871	1,168663	Control	DMSO
AT4G3874(	6	2	79,8666	0,514595707	-0,28853	1,42807	1mg	Control
AT1G0974(	5	5	45,8613	0,514980595	-0,28821	1,149784	DMSO	1mg
AT5G2277(	1	1	11,6976	0,516523558	-0,28691	1,694963	DMSO	Control
AT5G0473(	2	2	11,7767	0,517713992	-0,28591	1,250671	Control	1mg
AT3G2553(	18	11	121,2776	0,517981105	-0,28569	1,226434	1mg	Control
AT1G3134(	7	1	71,526	0,518817855	-0,28499	1,651211	Control	DMSO
AT5G1056(	2	2	17,2082	0,521086267	-0,28309	1,108312	Control	DMSO
AT3G4472(	3	1	16,9259	0,522290047	-0,28209	1,283426	Control	DMSO
AT5G2523(	6	1	33,5481	0,52277297	-0,28169	3,196143	1mg	Control
AT3G1724(	23	10	186,8616	0,523317668	-0,28123	1,158522	DMSO	Control
AT1G6643(	2	1	10,7019	0,523505803	-0,28108	1,338629	DMSO	1mg
AT5G3783(	8	5	43,2281	0,526164401	-0,27888	1,117449	DMSO	Control
AT5G3623(	10	4	71,083	0,527561809	-0,27773	1,538593	1mg	DMSO
AT1G0789(	15	12	219,1755	0,52796202	-0,2774	1,204871	Control	1mg
AT1G0469(	2	1	13,6687	0,5281125	-0,27727	1,301678	Control	1mg
AT5G0867(	41	30	509,1099	0,529439286	-0,27618	1,177038	1mg	Control
AT4G2913(	16	9	117,5726	0,529538038	-0,2761	1,201987	DMSO	Control
AT1G3020(	1	1	5,5779	0,53043441	-0,27537	2,188091	DMSO	Control
AT2G3719(	10	3	92,6298	0,530759352	-0,2751	1,222401	DMSO	Control

AT3G5607(	7	3	56,0225	0,530933655	-0,27496	1,19159	1mg	DMSO
AT1G7759(	4	2	32,7439	0,530984563	-0,27492	1,366705	DMSO	1mg
AT2G0571(	47	26	425,4263	0,531308449	-0,27465	1,106149	DMSO	Control
AT2G4210(	21	7	209,6389	0,534018846	-0,27244	1,235594	1mg	Control
AT5G0981(	28	6	342,028	0,534425408	-0,27211	1,274222	DMSO	1mg
AT2G1898(	12	5	95,2069	0,535474079	-0,27126	1,31992	Control	1mg
AT1G6377(	25	15	173,1071	0,537165353	-0,26989	1,121935	DMSO	1mg
AT2G2113(	6	2	60,2807	0,537562497	-0,26957	1,138281	DMSO	1mg
AT4G3707(	14	8	94,8761	0,53865748	-0,26869	1,305988	DMSO	Control
AT3G1074(	18	12	135,4109	0,538681602	-0,26867	1,132116	Control	1mg
AT5G2678(	4	2	22,3796	0,540053403	-0,26756	1,276491	1mg	Control
AT2G1896(	34	4	316,8091	0,541123178	-0,2667	1,206845	Control	DMSO
AT2G4391(	8	7	97,2321	0,541389285	-0,26649	1,182525	Control	1mg
AT3G1183(	24	17	182,1928	0,541973203	-0,26602	1,360752	1mg	DMSO
AT2G4773(	5	3	33,2343	0,545064832	-0,26355	2,17622	DMSO	Control
AT5G5600(	46	4	459,9456	0,546284043	-0,26258	1,218024	Control	DMSO
AT1G2020(	3	1	18,0389	0,547129066	-0,26191	1,405258	Control	DMSO
AT1G4389(	3	2	17,9652	0,547262535	-0,2618	1,641976	DMSO	1mg
AT1G5084(	2	1	10,633	0,549238276	-0,26024	1,471027	Control	DMSO
AT3G0128(	19	13	206,0242	0,549447617	-0,26007	1,091188	1mg	Control
AT1G6593(	34	19	422,2565	0,549728466	-0,25985	1,157334	1mg	Control
AT1G3572(	19	13	150,702	0,550430718	-0,2593	1,577226	DMSO	1mg
AT5G5500(	2	1	10,4442	0,551310643	-0,2586	1,289723	1mg	DMSO
AT5G4881(	2	2	16,8713	0,555220918	-0,25553	1,295174	DMSO	1mg
AT3G2285(	5	2	29,7685	0,555509533	-0,25531	1,28813	1mg	Control
AT2G1994(	7	4	46,5773	0,555576784	-0,25526	1,223084	DMSO	Control
AT1G6535(	8	2	85,2485	0,556017174	-0,25491	1,287773	Control	DMSO
AT1G7933(	5	2	29,3346	0,556064011	-0,25488	1,821126	1mg	DMSO
AT5G5735(	24	6	190,8412	0,558237065	-0,25318	1,133084	Control	DMSO
AT4G2836(	1	1	13,6802	0,558345418	-0,2531	1,103284	DMSO	1mg
AT2G0417(	7	3	58,0379	0,559551543	-0,25216	1,083796	1mg	DMSO
AT4G3158(	2	2	11,9001	0,561514421	-0,25064	1,208035	Control	1mg
AT2G4216(	1	1	5,3602	0,56306893	-0,24944	4,07677	DMSO	1mg
AT2G1645(	3	2	22,3132	0,5631693	-0,24936	1,102288	DMSO	1mg
AT3G1163(	3	2	21,6944	0,563480365	-0,24912	1,219979	Control	DMSO
AT5G1086(	4	3	26,9037	0,563732457	-0,24893	1,255339	DMSO	Control
AT5G2802(	7	5	42,9365	0,56674228	-0,24661	1,092841	1mg	DMSO
AT3G2586(	19	10	149,4427	0,567346473	-0,24615	1,125877	1mg	Control
AT1G5584(	3	2	22,8255	0,569776074	-0,2443	1,158773	Control	DMSO
AT3G5205(	2	1	10,0774	0,571163051	-0,24324	1,145506	1mg	DMSO
AT5G0703(	14	10	100,1576	0,571603224	-0,24291	1,240939	DMSO	1mg
AT1G5048(	20	16	132,0964	0,571912179	-0,24267	1,059795	Control	DMSO
AT5G0227(	6	4	39,9232	0,572225977	-0,24243	1,169505	DMSO	1mg
AT5G2383(	8	7	61,4023	0,57442139	-0,24077	1,125564	1mg	DMSO
AT3G0554(	1	1	5,7949	0,57538326	-0,24004	1,249029	Control	DMSO
AT1G1328(	7	3	50,8212	0,575401449	-0,24003	1,262901	1mg	Control
AT4G1734(	4	1	38,3926	0,575630588	-0,23986	1,17944	DMSO	1mg
AT5G2106(	4	3	22,4596	0,577176089	-0,23869	1,258349	DMSO	1mg
AT1G7987(	2	1	11,6645	0,578892449	-0,2374	1,377382	DMSO	1mg
AT4G2921(	1	1	5,5541	0,581763749	-0,23525	1,328018	1mg	DMSO

AT3G5544(	18	15	241,5355	0,58178093	-0,23524	1,221867	DMSO	Control
AT1G0603(	9	2	78,8983	0,586690579	-0,23159	1,221407	Control	1mg
AT2G3683(	2	1	21,5381	0,587616715	-0,23091	1,228874	1mg	Control
AT2G4547(	13	7	136,1383	0,589018048	-0,22987	1,259593	1mg	Control
AT5G5450(	7	7	71,0421	0,589086845	-0,22982	1,1488	1mg	DMSO
AT1G0927(	4	3	25,6074	0,589399151	-0,22959	1,19945	DMSO	1mg
AT4G0031!	1	1	5,8412	0,58970158	-0,22937	15,41117	DMSO	1mg
AT5G6319(	4	3	21,9984	0,591204966	-0,22826	1,252438	1mg	Control
AT1G0655(	5	2	34,2859	0,592229872	-0,22751	1,648351	1mg	Control
AT1G4976(	6	3	41,2291	0,59224182	-0,2275	1,210312	1mg	DMSO
AT5G0226(	1	1	6,2317	0,59240493	-0,22738	1,294961	1mg	DMSO
AT5G5346(	6	3	37,5131	0,592630177	-0,22722	1,171039	DMSO	Control
AT5G2741(	2	1	10,6474	0,594383236	-0,22593	1,561707	Control	1mg
AT3G0287(	2	2	11,0006	0,594748375	-0,22567	1,255261	1mg	Control
AT4G2098(	12	3	73,1354	0,595228754	-0,22532	1,244459	1mg	Control
AT2G4615(	2	1	10,6376	0,595561531	-0,22507	2,459057	DMSO	1mg
AT2G1976(	7	4	85,1919	0,595890278	-0,22483	1,178269	Control	DMSO
AT3G0635(	8	6	45,2357	0,597410517	-0,22373	1,19254	Control	1mg
AT2G3315(	22	16	201,0661	0,597509675	-0,22366	1,087133	Control	DMSO
AT2G2623(	2	1	10,1945	0,597691477	-0,22352	1,525736	1mg	Control
AT2G4529(	22	11	173,1307	0,599030924	-0,22255	1,107886	Control	1mg
AT3G4912(	11	3	112,3596	0,600448329	-0,22152	1,314049	Control	1mg
AT3G5624(	1	1	6,7338	0,601263436	-0,22094	1,765805	DMSO	Control
AT3G2936(	20	6	171,2006	0,603495339	-0,21933	1,250734	DMSO	Control
AT4G2984(	20	11	152,6029	0,60415607	-0,21885	1,117365	1mg	Control
AT5G0501(	13	11	98,0602	0,604789569	-0,2184	1,119233	DMSO	Control
AT4G0319(	4	3	21,3565	0,605429691	-0,21794	1,27439	1mg	Control
AT1G0848(	5	3	41,7352	0,605844125	-0,21764	1,30546	DMSO	Control
AT5G2805(	5	2	38,2878	0,606211179	-0,21738	1,37329	DMSO	Control
AT2G2956(	7	4	52,4241	0,607042502	-0,21678	1,122295	Control	1mg
AT1G6081(	8	1	70,7812	0,609236493	-0,21521	1,175049	DMSO	1mg
AT1G7838(	2	1	12,6264	0,609281679	-0,21518	1,366	Control	DMSO
AT3G4817(	10	7	72,0252	0,609329735	-0,21515	1,098344	1mg	Control
AT4G1115(	6	3	43,0708	0,609775014	-0,21483	1,244533	1mg	DMSO
AT2G4405(	6	4	37,46	0,611161038	-0,21384	1,172148	1mg	Control
AT1G4966(	3	3	19,3636	0,613441708	-0,21223	1,066405	DMSO	1mg
AT4G2365(	14	11	112,9075	0,616436928	-0,21011	1,071806	DMSO	1mg
AT1G2062(	14	9	110,7765	0,617358297	-0,20946	1,149471	DMSO	1mg
AT5G1371(	2	2	12,1234	0,619082039	-0,20825	1,261225	DMSO	Control
AT5G6693(	1	1	5,6054	0,620735625	-0,20709	1,131729	Control	1mg
AT4G2389!	9	2	66,9154	0,621888345	-0,20629	1,290386	Control	1mg
AT2G2381(	1	1	5,7114	0,624417847	-0,20452	1,197925	DMSO	Control
AT5G4703(	5	5	35,7994	0,62469088	-0,20433	1,661294	1mg	Control
AT5G0330(	19	4	223,1368	0,625992622	-0,20343	1,172672	DMSO	1mg
AT1G0775(	12	7	93,5833	0,626687573	-0,20295	1,092497	1mg	DMSO
AT4G2385(	23	18	165,8678	0,627203891	-0,20259	1,136519	DMSO	Control
AT1G7890(	52	40	595,1578	0,627903627	-0,20211	1,123795	DMSO	1mg
AT3G5761(	18	13	110,5884	0,629826578	-0,20078	1,151124	DMSO	Control
AT5G2389(	1	1	6,2747	0,631504142	-0,19962	1,141026	DMSO	1mg
AT5G4378(	7	4	39,3703	0,63165313	-0,19952	1,342188	DMSO	1mg

AT3G2430(	2	1	11,2627	0,633748569	-0,19808	1,33883	DMSO	1mg
AT1G2331(	4	2	22,3314	0,63415757	-0,1978	1,317099	DMSO	Control
AT3G4432(	9	5	65,4357	0,634170877	-0,19779	1,090741	Control	DMSO
AT5G6412(	4	4	23,1478	0,63557214	-0,19684	1,304656	DMSO	1mg
AT5G4279(	15	3	116,4008	0,63671716	-0,19605	1,145254	Control	1mg
AT5G1817(	9	4	81,3805	0,637068237	-0,19581	1,343301	Control	1mg
AT2G3933(	5	1	33,692	0,637829933	-0,1953	1,283742	1mg	Control
AT3G0982(	20	7	231,429	0,638635762	-0,19475	1,082188	Control	1mg
AT1G7603(	28	5	298,0517	0,640195807	-0,19369	1,08587	1mg	Control
AT4G1101(	11	6	99,9403	0,64054654	-0,19345	1,179574	1mg	DMSO
AT5G3952(	2	1	18,3773	0,642498162	-0,19213	1,630332	1mg	DMSO
AT5G0363(	30	22	307,2443	0,646027318	-0,18975	1,09391	DMSO	1mg
AT3G2211(	8	6	62,1676	0,650096643	-0,18702	1,289341	1mg	DMSO
AT3G1702(	6	5	45,9949	0,651227839	-0,18627	1,527761	Control	1mg
AT3G1776(	7	2	65,7532	0,651428371	-0,18613	1,587476	1mg	DMSO
AT1G1729(	20	12	152,9122	0,652087851	-0,18569	1,115324	DMSO	1mg
AT5G5085(	11	10	83,7868	0,653165412	-0,18498	1,1392	DMSO	1mg
AT1G5147(	20	4	227,871	0,653713022	-0,18461	1,226256	DMSO	1mg
AT3G2792!	1	1	5,0703	0,654154525	-0,18432	1,152462	DMSO	Control
AT2G4221(	2	1	12,0579	0,65841725	-0,1815	1,218297	DMSO	1mg
AT3G5789(	4	4	23,6668	0,659784847	-0,1806	1,182712	1mg	Control
AT4G1614:	15	7	97,3224	0,660624342	-0,18005	1,100716	1mg	DMSO
AT1G6758(	3	1	16,7872	0,660716587	-0,17998	1,57652	Control	1mg
AT5G2099(	1	1	5,552	0,661154774	-0,1797	1,188786	DMSO	Control
AT3G5220(	4	3	22,1311	0,662253652	-0,17898	1,126068	1mg	Control
AT5G6351(	9	2	60,0789	0,662817933	-0,17861	1,075065	Control	1mg
AT5G1700(	1	1	5,1965	0,663494477	-0,17816	1,670322	DMSO	1mg
AT3G6161(	2	1	11,3369	0,664864684	-0,17727	1,267366	Control	DMSO
AT1G5276(	6	5	38,5355	0,666554443	-0,17616	1,184997	DMSO	1mg
AT1G5328(	3	2	17,1862	0,667987269	-0,17523	1,224598	DMSO	Control
AT2G3092(	2	2	10,6832	0,669523004	-0,17423	1,098328	Control	DMSO
AT3G5411(	9	4	54,1139	0,670341179	-0,1737	1,086103	DMSO	1mg
AT5G5095(	11	2	79,4853	0,671928993	-0,17268	1,143852	DMSO	1mg
AT5G6256(	1	1	5,0979	0,672424749	-0,17236	1,935069	Control	1mg
AT1G1593(	7	3	53,9051	0,677656375	-0,16899	1,177825	1mg	Control
AT4G2839(	19	2	181,2408	0,678348259	-0,16855	1,048564	Control	1mg
AT3G2578(	7	5	62,2097	0,680274478	-0,16732	1,065503	1mg	Control
AT5G1792(	58	22	825,3524	0,680623733	-0,16709	1,137591	DMSO	Control
AT2G4122(	34	20	229,2857	0,681229614	-0,16671	1,082035	1mg	DMSO
AT1G1603(	16	3	143,7471	0,681876685	-0,16629	1,226512	1mg	Control
AT2G4435(	9	7	56,854	0,682018757	-0,1662	1,168191	Control	DMSO
AT4G3016(	4	2	22,039	0,682063762	-0,16618	1,080513	1mg	DMSO
AT5G4848(	6	4	37,344	0,683194219	-0,16546	1,137113	DMSO	1mg
AT4G3579(	8	4	55,1353	0,683453768	-0,16529	1,120413	1mg	Control
AT5G1311(	10	4	57,3981	0,683965148	-0,16497	1,077833	Control	1mg
AT1G4712{	13	8	114,7412	0,685790407	-0,16381	1,144328	1mg	Control
AT4G3676(	9	5	63,0275	0,686270337	-0,1635	1,148343	Control	DMSO
AT3G0665(	23	4	193,426	0,687914957	-0,16247	1,072008	DMSO	Control
AT3G2289(	5	3	38,5044	0,68845912	-0,16212	1,167994	Control	DMSO
AT2G2278(	7	4	52,8295	0,689053859	-0,16175	1,095077	Control	DMSO

AT5G1166(	2	2	15,7368	0,689467471	-0,16149	2,15819	Control	1mg
AT5G6098(	3	3	16,2379	0,689568076	-0,16142	1,164782	1mg	DMSO
AT5G1509(	18	12	171,3678	0,689743578	-0,16131	1,091801	1mg	Control
AT2G4153(	4	3	32,6064	0,69035244	-0,16093	1,103657	Control	DMSO
AT1G4967(	5	2	32,7208	0,690567946	-0,16079	1,218102	1mg	Control
AT5G6502(	8	7	48,397	0,691959871	-0,15992	1,526607	1mg	Control
AT4G3420(	25	17	278,7157	0,692185866	-0,15978	1,196094	DMSO	Control
AT5G3900(	5	1	27,2752	0,695312927	-0,15782	1,207014	DMSO	Control
AT3G1598(	15	2	88,4706	0,695389402	-0,15777	1,464129	1mg	Control
AT3G4434(	13	10	73,353	0,695946959	-0,15742	1,120254	DMSO	Control
AT4G0877(	8	3	87,7628	0,695984071	-0,1574	1,144642	1mg	DMSO
AT1G4771(	7	4	39,8989	0,696174007	-0,15728	1,084196	Control	1mg
AT1G3036(	9	5	63,0264	0,697580867	-0,15641	1,063987	DMSO	Control
AT1G5641(	20	2	217,9509	0,698822673	-0,15563	1,151248	DMSO	1mg
AT1G6641(	6	1	57,6191	0,699099538	-0,15546	1,631914	DMSO	Control
AT5G2025(	8	3	49,9849	0,705258155	-0,15165	1,073524	DMSO	Control
AT1G5400(	18	11	220,8254	0,706440672	-0,15092	1,180139	Control	1mg
AT1G6071(	9	2	52,2022	0,707640289	-0,15019	1,099884	1mg	DMSO
AT2G3175(	5	3	41,9514	0,708383442	-0,14973	1,11398	1mg	Control
AT2G2620(	1	1	5,0437	0,709040153	-0,14933	1,556241	DMSO	Control
AT3G6107(	3	2	16,8508	0,709550386	-0,14902	1,153188	1mg	DMSO
AT1G7364(	3	1	18,4419	0,709801457	-0,14886	1,68157	DMSO	1mg
AT1G7080(	2	1	15,9808	0,710085453	-0,14869	1,494877	Control	DMSO
AT3G1959	2	1	14,3134	0,712882451	-0,14698	1,068949	Control	DMSO
AT3G0994(	12	10	89,1281	0,71338682	-0,14667	1,109611	DMSO	Control
AT2G3631(	2	1	11,5638	0,714435875	-0,14604	1,425102	Control	DMSO
AT3G1502(	13	3	136,2358	0,715556267	-0,14536	1,091551	1mg	DMSO
AT5G3764(	10	2	87,0193	0,716783038	-0,14461	1,357152	DMSO	1mg
AT5G0141(	1	1	6,1645	0,718349681	-0,14366	1,143827	DMSO	Control
AT3G0920(	15	7	201,1372	0,719775779	-0,1428	1,259403	1mg	Control
AT2G1417(	14	8	95,3973	0,723583003	-0,14051	1,087535	DMSO	1mg
AT2G1742(	13	13	146,3594	0,72398708	-0,14027	1,089025	1mg	Control
AT3G5526(	2	2	11,7301	0,724319313	-0,14007	1,024978	DMSO	Control
AT3G2652(	2	2	22,4677	0,725227698	-0,13953	1,165347	DMSO	Control
AT5G4434(	25	3	306,4206	0,725845404	-0,13916	1,165678	DMSO	Control
AT4G1488(	18	9	162,6233	0,726257915	-0,13891	1,078917	1mg	DMSO
AT1G3129(	4	2	22,2964	0,726501525	-0,13876	1,335586	DMSO	Control
AT5G1168(	1	1	13,1772	0,728014991	-0,13786	1,836762	1mg	DMSO
AT1G6628(	32	15	362,6234	0,728618363	-0,1375	1,123901	DMSO	1mg
AT2G3973(	7	6	43,8319	0,729789355	-0,1368	1,053781	Control	1mg
AT1G7578(	22	3	233,6524	0,730588089	-0,13633	1,267963	DMSO	1mg
AT2G0153(	6	2	62,9006	0,734347044	-0,1341	1,425454	Control	DMSO
AT1G7528(	2	1	12,1503	0,735480403	-0,13343	1,192354	Control	DMSO
AT2G2012(	1	1	5,5943	0,736878323	-0,1326	1,203527	Control	1mg
AT1G7679(	9	4	59,8733	0,738058862	-0,13191	1,074346	Control	DMSO
AT5G6274(	3	1	19,3558	0,741177429	-0,13008	1,178076	DMSO	Control
AT4G1273(	7	6	65,4709	0,741750599	-0,12974	1,118378	DMSO	1mg
AT2G2702(	14	10	106,6995	0,741936451	-0,12963	1,05856	DMSO	Control
AT5G4823(	21	19	205,7308	0,742526419	-0,12929	1,122823	DMSO	1mg
AT4G3148(	28	22	219,7519	0,746050628	-0,12723	1,072081	DMSO	Control

AT4G3083(	7	5	38,9192	0,747251382	-0,12653	1,113505	1mg	DMSO
AT1G5826!	1	1	5,6116	0,748220892	-0,12597	1,612345	Control	1mg
AT1G6728(	6	3	39,0015	0,749750872	-0,12508	1,097683	1mg	Control
AT4G2482(	5	2	27,5497	0,749890794	-0,125	1,151917	DMSO	Control
AT5G0853(	12	8	92,043	0,751595296	-0,12402	1,099299	Control	1mg
AT3G4606(	7	4	55,2021	0,752456934	-0,12352	1,169571	1mg	DMSO
AT1G6428(	1	1	5,999	0,753162666	-0,12311	1,160343	1mg	Control
AT4G2158(	6	5	34,2399	0,7554203	-0,12181	1,080068	1mg	DMSO
AT2G3838(	12	7	134,0046	0,756844439	-0,12099	1,163218	DMSO	1mg
AT5G5264(	20	6	177,7554	0,757778199	-0,12046	1,123229	DMSO	1mg
AT5G4407(	6	4	36,0261	0,757890183	-0,12039	1,20718	1mg	Control
AT5G2745(	1	1	5,161	0,758641933	-0,11996	1,381358	Control	1mg
AT2G3874(	3	2	22,9236	0,759981227	-0,1192	1,152564	1mg	DMSO
AT5G5676(	2	2	10,7897	0,760320882	-0,119	1,085495	1mg	Control
AT2G0256(	18	14	118,006	0,760912957	-0,11867	1,138827	1mg	Control
AT3G2148(	2	1	11,507	0,764080341	-0,11686	2,435652	1mg	Control
AT5G4551(	4	3	41,6039	0,764690486	-0,11651	1,118648	1mg	Control
AT5G0980(	4	2	21,9926	0,765256548	-0,11619	1,23066	DMSO	Control
AT3G2523(	6	6	48,9598	0,765471214	-0,11607	1,219681	1mg	Control
AT5G6172(	1	1	5,4127	0,766296324	-0,1156	1,435537	1mg	Control
AT5G0959(	29	16	199,8769	0,766642404	-0,11541	1,039922	1mg	DMSO
AT4G3565(	12	4	98,9439	0,76678052	-0,11533	1,083599	1mg	DMSO
AT1G7923(	13	11	85,5346	0,767037025	-0,11518	1,053293	Control	DMSO
AT3G1595(	49	41	492,8129	0,769812982	-0,11361	1,093616	Control	1mg
AT2G2458(	5	4	29,0271	0,771386119	-0,11273	1,215235	Control	DMSO
AT2G3093(	6	4	36,138	0,774584615	-0,11093	1,131467	1mg	DMSO
AT3G1005(	2	2	10,7699	0,776790343	-0,1097	1,246101	DMSO	Control
AT4G2201(	10	7	75,1258	0,777095097	-0,10953	1,07945	DMSO	Control
AT5G5348(	14	11	90,7927	0,777407173	-0,10935	1,07344	DMSO	Control
AT5G4274(	6	6	48,5797	0,778446864	-0,10877	1,149331	1mg	Control
AT2G3020(	17	13	118,7821	0,779062219	-0,10843	1,090688	DMSO	Control
AT1G2095(	28	18	226,6477	0,780944279	-0,10738	1,135882	DMSO	Control
AT1G1821(	7	6	45,7885	0,783018644	-0,10623	1,1143	DMSO	1mg
AT2G0769{	34	28	400,4613	0,784179226	-0,10558	1,080792	1mg	Control
AT5G2636(	23	19	166,7206	0,787058199	-0,10399	1,067546	DMSO	Control
AT5G3760(	16	5	144,1335	0,790525635	-0,10208	1,118352	1mg	Control
AT1G1247(	3	1	16,2516	0,791441383	-0,10158	1,243286	DMSO	Control
AT3G1133(	3	2	16,3942	0,797278098	-0,09839	1,109275	DMSO	Control
AT5G6357(	5	3	27,3798	0,79770834	-0,09816	1,089898	1mg	DMSO
AT2G0440(	7	3	43,4935	0,798207849	-0,09788	1,118092	DMSO	Control
AT2G2800(	23	17	151,5012	0,798399361	-0,09778	1,081177	DMSO	1mg
AT1G0641(	5	2	27,8806	0,798454365	-0,09775	1,239483	Control	DMSO
AT5G4219(	4	1	30,062	0,798888256	-0,09751	1,356123	DMSO	Control
AT4G3637(	3	1	16,1045	0,804291111	-0,09459	1,334342	Control	1mg
AT1G6366(	8	4	42,4426	0,804753504	-0,09434	1,119467	DMSO	1mg
AT2G4291(	16	11	127,6176	0,805346503	-0,09402	1,062625	Control	DMSO
AT3G0669(	2	1	11,4332	0,807470255	-0,09287	1,165028	1mg	Control
AT4G0553(	6	4	49,2977	0,808787528	-0,09217	1,074671	DMSO	1mg
AT2G4006(	3	2	18,0469	0,809390191	-0,09184	1,10771	DMSO	Control
AT2G3065(	2	1	12,0778	0,809866648	-0,09159	5,971769	Control	1mg

AT2G2188(	2	1	11,4319	0,811002608	-0,09098	1,079899	1mg	DMSO
AT5G4394(	15	13	126,5756	0,814214562	-0,08926	1,076613	DMSO	1mg
AT5G0287(	23	6	220,1974	0,815270978	-0,0887	1,095303	DMSO	1mg
AT5G1345(	2	1	11,7976	0,816948114	-0,08781	1,112304	1mg	DMSO
AT1G1957(	6	5	61,5957	0,817945194	-0,08728	1,484098	DMSO	1mg
AT4G0062(	3	2	16,091	0,81931217	-0,08655	2,416865	Control	1mg
AT5G2382(	4	3	34,8868	0,81952559	-0,08644	1,125367	DMSO	1mg
AT1G6182(	1	1	5,5113	0,819927534	-0,08622	1,15486	1mg	DMSO
AT1G6941(	5	1	51,4112	0,82286555	-0,08467	1,200137	Control	1mg
AT4G1198(	1	1	10,0744	0,827702088	-0,08213	1,077774	1mg	DMSO
AT5G2510(	5	3	35,807	0,827753879	-0,0821	1,104654	DMSO	1mg
AT5G5459(	2	1	11,1829	0,828070244	-0,08193	1,193896	1mg	Control
AT1G5365(	1	1	5,9535	0,830682502	-0,08056	1,047177	Control	1mg
AT5G5825(	2	1	10,569	0,830683958	-0,08056	1,211667	DMSO	1mg
AT2G3273(	21	10	146,5203	0,830996777	-0,0804	1,036387	DMSO	Control
AT3G6313(	3	3	15,9105	0,833085039	-0,07931	1,069366	1mg	Control
AT5G5942(	3	2	18,2645	0,833472503	-0,07911	1,113305	1mg	DMSO
AT1G3063(	2	1	10,8138	0,835713744	-0,07794	1,07758	1mg	DMSO
AT3G5299(	26	4	207,6628	0,83630528	-0,07764	1,052343	Control	1mg
AT4G2991(	2	1	10,9304	0,839799485	-0,07582	1,040175	DMSO	1mg
AT3G2220(	23	16	195,9674	0,840817075	-0,0753	1,050682	Control	1mg
AT1G2436(	14	8	106,1813	0,844338802	-0,07348	1,055468	1mg	Control
AT1G5375(	10	5	71,3443	0,847410606	-0,07191	1,169243	Control	1mg
AT2G4250(	8	7	56,646	0,847828151	-0,07169	1,225632	DMSO	Control
AT5G4822(	2	2	10,4961	0,84850388	-0,07135	1,103264	DMSO	Control
AT4G1833(	3	1	17,3458	0,850213584	-0,07047	1,103867	DMSO	Control
AT3G4853(	7	6	39,6345	0,852329348	-0,06939	1,066622	Control	DMSO
AT5G5331(	1	1	5,1414	0,852978823	-0,06906	1,035118	Control	1mg
AT3G5902(	4	3	21,8817	0,856843913	-0,0671	1,261492	Control	1mg
AT1G0811(	2	1	10,9364	0,857098624	-0,06697	2,796505	DMSO	Control
AT1G8056(	6	3	44,0836	0,85713419	-0,06695	1,179373	DMSO	1mg
AT1G3314(	14	7	139,89	0,85902327	-0,066	1,122163	DMSO	1mg
AT1G1726(	15	8	101,7635	0,861751281	-0,06462	1,171064	1mg	Control
AT4G2639(	15	1	101,5197	0,862406407	-0,06429	1,147715	DMSO	1mg
AT3G4968(	2	1	11,1913	0,864548144	-0,06321	1,222276	DMSO	Control
AT4G3880(	8	6	68,0244	0,865994869	-0,06248	1,130211	DMSO	1mg
AT5G4027(	1	1	5,4296	0,868248184	-0,06136	1,117945	DMSO	1mg
AT1G4296(	1	1	7,5526	0,869165258	-0,0609	1,054025	1mg	Control
AT1G5772(	16	6	138,5627	0,869284233	-0,06084	1,045764	Control	1mg
AT3G5288(	17	15	108,625	0,870609778	-0,06018	1,02635	1mg	Control
AT3G0396(	24	22	179,2693	0,8732909	-0,05884	1,065709	DMSO	Control
AT3G2730(	9	4	49,1311	0,875763917	-0,05761	1,190632	1mg	DMSO
AT4G2115(	21	15	170,9528	0,876865936	-0,05707	1,050272	DMSO	Control
AT2G3999(	14	12	120,7259	0,87828075	-0,05637	1,073274	DMSO	1mg
AT3G0112(	10	7	62,4139	0,878385084	-0,05632	1,076403	1mg	DMSO
AT2G4309(	13	9	84,2436	0,883318563	-0,05388	1,07268	1mg	DMSO
AT3G4644(	10	5	67,7487	0,88352094	-0,05378	1,142285	DMSO	1mg
AT1G0962(	35	26	236,4994	0,884172601	-0,05346	1,018713	Control	1mg
AT2G4336(	1	1	5,2539	0,885025741	-0,05304	1,049387	DMSO	1mg
AT1G4501!	2	2	12,9679	0,88574762	-0,05269	1,018919	DMSO	Control

AT2G2335(	13	6	87,1514	0,885823962	-0,05265	1,051657	DMSO	1mg
AT3G6143(	15	6	157,8093	0,887285249	-0,05194	1,072596	1mg	Control
AT3G0209(	18	14	158,3593	0,889170013	-0,05102	1,141779	1mg	DMSO
AT3G6212(	14	13	81,7413	0,890418963	-0,05041	1,147987	1mg	Control
AT5G5354(	3	1	17,7659	0,892974669	-0,04916	1,096593	1mg	DMSO
AT5G6750(	20	14	171,5473	0,895035406	-0,04816	1,05498	Control	DMSO
AT1G4742(	2	2	11,1477	0,895924855	-0,04773	1,053302	Control	1mg
AT1G0777(	6	3	66,8239	0,897541715	-0,04695	1,179569	DMSO	Control
AT5G5548(	12	7	70,2907	0,897833589	-0,0468	1,072212	1mg	Control
AT2G3161(	16	1	188,1962	0,897961914	-0,04674	1,092785	DMSO	Control
AT5G1167(	30	18	306,0472	0,901216914	-0,04517	1,036935	1mg	DMSO
AT5G0812(	2	1	10,2334	0,905060992	-0,04332	1,037207	1mg	Control
AT5G5729(	3	1	36,1323	0,909400048	-0,04125	1,055358	DMSO	1mg
AT3G6088(	2	1	11,1035	0,911705573	-0,04015	1,061779	Control	1mg
AT2G0599(	20	14	133,4331	0,9129459	-0,03955	1,046041	1mg	Control
AT5G4550(	2	1	11,307	0,913463601	-0,03931	1,030173	Control	DMSO
AT3G2577(	5	1	36,4614	0,915053539	-0,03855	1,041601	Control	1mg
AT3G0479(	4	2	23,5695	0,915572664	-0,03831	1,48262	Control	DMSO
AT5G2629(	6	4	37,7475	0,917536721	-0,03738	1,051068	DMSO	1mg
AT3G4430(	4	1	23,524	0,91961369	-0,03639	1,062885	1mg	DMSO
AT2G3086(	12	9	145,0803	0,920546115	-0,03595	1,065503	1mg	DMSO
AT3G3298(	17	8	232,3017	0,922491211	-0,03504	1,302421	DMSO	1mg
AT2G1474(	6	3	33,9167	0,922553537	-0,03501	1,132297	DMSO	1mg
AT2G1356(	10	8	67,08	0,92479381	-0,03396	1,070777	Control	1mg
AT5G1054(	14	7	84,6458	0,927008882	-0,03292	1,049248	1mg	DMSO
AT1G7993(	38	7	303,2756	0,927615747	-0,03263	1,036075	Control	DMSO
AT3G1180(	3	3	23,8128	0,928094389	-0,03241	1,055727	1mg	Control
AT1G4417(	7	5	42,7482	0,928494652	-0,03222	1,041446	Control	1mg
AT4G2935(	8	4	94,2831	0,928993978	-0,03199	1,040695	1mg	DMSO
AT4G3114(	1	1	5,9571	0,931353297	-0,03089	1,519541	DMSO	1mg
AT4G2744(	2	1	20,2813	0,934477186	-0,02943	1,071295	Control	1mg
AT5G1403(	7	6	55,7901	0,934874853	-0,02925	1,084604	1mg	DMSO
AT1G2230(	6	4	46,1682	0,9350323	-0,02917	1,155496	DMSO	1mg
AT3G6001(	2	1	11,7719	0,935248649	-0,02907	1,788047	1mg	Control
AT3G0253(	19	6	187,6372	0,937129507	-0,0282	1,038639	Control	DMSO
AT1G1481(	12	10	86,5409	0,939747852	-0,02699	1,030386	DMSO	Control
AT1G7955(	30	23	348,0833	0,94298493	-0,0255	1,037144	1mg	Control
AT1G2663(	14	7	137,9762	0,944146651	-0,02496	1,040562	Control	1mg
AT1G0174(	1	1	5,1363	0,944380137	-0,02485	1,300983	Control	DMSO
AT3G5342(	11	3	135,3074	0,946477204	-0,02389	1,045262	1mg	Control
AT4G3509(	12	5	100,6606	0,952840392	-0,02098	1,187745	1mg	Control
AT1G1332(	17	3	100,0402	0,953421473	-0,02072	1,059749	DMSO	Control
AT4G2462(	24	16	178,8089	0,953747071	-0,02057	1,026275	1mg	Control
AT2G1990(	9	4	73,7211	0,956884001	-0,01914	1,005247	Control	1mg
ATCG0042(	1	1	5,524	0,957240981	-0,01898	1,196618	1mg	Control
AT4G1480(	8	4	60,5973	0,957594893	-0,01882	1,020678	1mg	Control
AT1G5331(	35	6	309,7013	0,959245453	-0,01807	1,0138	1mg	DMSO
AT1G2148(	3	2	16,6102	0,961583731	-0,01701	1,022746	1mg	DMSO
AT3G6033(	10	2	76,9871	0,962385557	-0,01665	1,0672	Control	1mg
AT4G0002(	4	1	20,3826	0,963756433	-0,01603	1,027451	1mg	Control

AT4G3863(	6	2	43,1487	0,968227818	-0,01402	1,077346	1mg	Control
AT1G1227(	1	1	5,0359	0,970307452	-0,01309	1,160289	DMSO	Control
AT5G4087(	2	1	10,9609	0,970316981	-0,01309	1,112588	DMSO	1mg
AT3G5180(	3	3	24,6694	0,971473148	-0,01257	1,157072	1mg	Control
AT3G2231(	2	1	15,8404	0,971619415	-0,0125	1,06671	DMSO	Control
AT5G3848(	17	5	137,6336	0,973345501	-0,01173	1,031772	DMSO	1mg
AT2G1660(	7	5	74,4934	0,973520714	-0,01165	1,275969	DMSO	Control
AT4G1393(	26	22	270,9306	0,978042367	-0,00964	1,02199	DMSO	1mg
AT4G2340(	11	5	110,7118	0,978395651	-0,00949	1,019685	DMSO	Control
AT3G0277(	1	1	5,5728	0,980071385	-0,00874	1,098555	Control	DMSO
AT1G4725(	12	2	97,4506	0,980747747	-0,00844	1,039004	DMSO	Control
AT4G2483(	18	16	126,683	0,982336149	-0,00774	1,053059	DMSO	1mg
AT5G1810(	2	1	13,0552	0,98454827	-0,00676	1,056863	Control	1mg
AT5G4745(	4	1	37,4458	0,986980289	-0,00569	1,029242	1mg	DMSO
AT3G5615(	22	15	146,4611	0,987110015	-0,00563	1,009765	DMSO	1mg
AT5G5507(	13	2	101,3637	0,988723058	-0,00493	1,108211	DMSO	Control
AT1G6238(	4	3	23,7691	0,98893719	-0,00483	1,082427	Control	1mg
AT1G7281(	13	7	81,1744	0,989494854	-0,00459	1,005674	1mg	Control
AT5G4195(	4	3	27,3819	0,991038949	-0,00391	1,019998	DMSO	1mg
AT3G5361(	3	1	22,6068	0,991342241	-0,00378	1,617503	Control	DMSO
AT5G4058(	1	1	5,4711	0,991382459	-0,00376	1,044336	DMSO	Control
AT4G2669(	12	9	95,1063	0,995719131	-0,00186	1,022498	1mg	DMSO
AT5G4680(	3	2	18,0116	0,997215073	-0,00121	1,014668	DMSO	1mg
AT3G2263(	7	3	54,7692	0,998359239	-0,00071	1,004073	1mg	DMSO
AT5G5356(	3	2	20,9523	0,998443113	-0,00068	1,180792	DMSO	Control
AT1G0482(	29	1	379,3547	0,998945285	-0,00046	1,045184	DMSO	Control

Description	2016_03_26_EDUARDO_Control_001	2016_03_26_EDUARDO_Control_002	2016_03_26_EDUARDO_Control_003	**MEDIA_CONTROL**	2016_03_26_EDUARDO_DMSO_001	2016_03_26_EDUARDO_DMSO_002	2016_03_26_EDUARDO_DMSO_003	**MEDIA_DMSO**	2016_03_26_EDUARDO_1mg_001
Peroxidase	12964,85	11940,75	10509,55	**11805,05**	72209,38	135546,7	118277,4	**108677,8**	12680,1
TRAF-like fa	239,4628	335,5721	289,6709	**288,2353**	231,8347	240,1516	306,8539	**259,6134**	77,07828
Pentatricop	57,73843	26,57803	33,79055	**39,369**	28,19632	9,143088	15,68649	**17,6753**	771,3225
P-loop cont	334,6455	262,257	297,3893	**298,0973**	156,5218	220,8911	239,8302	**205,7477**	8,79466
Nse4, comp	2820,415	2403,554	2478,532	**2567,501**	5006,123	5288,289	6074,095	**5456,169**	3969,788
PGL34 plas	2899,983	3044,967	3392,273	**3112,408**	4662,14	2930,964	3774,458	**3789,187**	728,5912
Ribosomal	0	0	0	**0**	64,37971	60,59894	450,0151	**191,6646**	0
MEK1, NMA	67,57178	209,0535	53,23443	**109,9532**	79,89603	369,1415	80,62545	**176,5543**	0
Plant invert	4286,645	5144,351	3723,264	**4384,753**	1236,228	1353,216	815,4489	**1134,965**	192,7069
Pentatricop	950,717	917,3897	867,8544	**911,9871**	437,5242	775,3815	398,0561	**536,9873**	133,3561
emb2386 R	224,0015	544,4437	688,5363	**485,6605**	7892,052	14378,28	19874,26	**14048,2**	1427,699
Peroxidase	2662,633	3537,942	2974,273	**3058,283**	5663,575	8842,677	9103,927	**7870,06**	1813,268
Small nucle	313,0691	427,6352	364,0162	**368,2402**	609,9139	382,9057	594,7178	**529,1791**	12,49268
DELTA-TIP,	12874,39	12961,62	13818,17	**13218,06**	17489,31	15960,9	22117,35	**18522,52**	7093,357
OVA5, ATKI	1209,097	1129,403	1151,373	**1163,291**	601,3518	372,2374	408,9218	**460,837**	1598,138
ATFRO4, FF	3514,034	5598,946	3964,044	**4359,008**	12205,64	11399,07	10711,86	**11438,86**	5446,602
Protein of u	12821,5	11096,12	13120,48	**12346,04**	12161,48	13999,91	14246,3	**13469,23**	6937,799
GRF9, GF14	3645,949	3817,12	3301,209	**3588,093**	4162,419	4703,363	4635,755	**4500,513**	2825,112
RHS12 root	3898,577	4217,864	4211,383	**4109,275**	4717,641	4683,884	5006,24	**4802,589**	5147,849
RHS13 root	3130,966	2614,688	2539,574	**2761,742**	5911,982	11000,7	11527,93	**9480,204**	1390,909
PRP3, ATPF	7508,601	7154,068	6375,468	**7012,712**	10726,58	14432,46	16877,99	**14012,34**	4980,892
sks16 SKU5	3584,786	2626,429	2677,134	**2962,783**	2288,086	2627,319	2331,892	**2415,766**	1454,527
MMT meth	23382,45	18162,1	23122,9	**21555,82**	23297,24	20380,79	20487,66	**21388,56**	12199,91
Protein of u	2491,735	2550,876	2852,897	**2631,836**	1733,059	2174,138	1701,772	**1869,656**	1513,737
AXS2 UDP-I	6699,71	5905,854	5971,793	**6192,452**	5140,213	5339,427	4985,556	**5155,066**	3169,166
EIF4A-III eu	19205,38	22190,93	21894,3	**21096,87**	25684,43	27599	26714,94	**26666,13**	18525,84
Kunitz fami	1429,331	2843,793	2502,106	**2258,41**	5133,685	12632,8	10593,42	**9453,301**	811,3743
SOX, AT-SO	4143,192	4287,893	3885,958	**4105,681**	4852,962	5058,618	4984,897	**4965,492**	5426,459
PGM2 Phos	3764,278	3956,306	4739,664	**4153,416**	3456,177	3962,796	4108,239	**3842,404**	2474,487
AGP31 arak	54501,57	60430,16	62232,31	**59054,68**	92247,34	111035,3	119441,5	**107574,7**	82385,99
Putative m	1039,003	827,4689	802,2961	**889,5892**	1747,159	814,1867	987,0434	**1182,796**	4682,385
CSR1, ALS,	25751,91	25476,31	26440,15	**25889,46**	20802,71	22174,1	19553,43	**20843,41**	20928,51
RNI-like su	13934,49	9023,053	9371,089	**10776,21**	9705,915	20716,56	8654,397	**13025,62**	2516,352
Heat shock	927,9053	799,6161	822,796	**850,1058**	1379,142	1317,526	1739,541	**1478,737**	1400,492
O-methyltr	15659,88	18358,95	16129,41	**16716,08**	22767,88	17926,73	23312,33	**21335,65**	9372,826
RPN1B, ATI	4753,529	5557,024	5269,158	**5193,237**	4744,678	6202,315	4223,079	**5056,69**	2465,345
AtFAAH, FA	600,1735	345,737	377,9406	**441,2837**	1091,824	302,6685	647,4052	**680,6325**	56,97369
AtRABA1i, I	1113,529	1143,748	1160,985	**1139,421**	1191,642	1506,702	1012,846	**1237,063**	2021,715

Ribosomal	225,3093	202,6723	275,6622	**234,548**	833,8499	1151,983	2010,843	**1332,225**	376,373
Plant mitoc	7227,503	6441,855	6971,011	**6880,123**	8888,773	9196,484	8517,994	**8867,751**	6639,096
Ribosomal	1010,235	989,7094	785,1499	**928,3647**	2766,976	3933,691	4005,371	**3568,679**	1537,223
RHD4 Phos	2817,339	2398,505	2202,111	**2472,652**	3511,566	3224,842	4215,779	**3650,729**	1851,673
Ribosomal	873,3527	1051,631	1067,58	**997,5212**	3650,308	6779,967	8638,077	**6356,117**	1606,963
ATFIM1, FII	1914,727	2211,006	2140,984	**2088,906**	2379,402	2082,713	2362,754	**2274,957**	2807,369
Protein of	701,7975	521,3503	784,3045	**669,1508**	397,8147	534,8937	164,0694	**365,5926**	76,34488
RPL34 ribo:	3009,795	2965,827	3400,314	**3125,312**	4614,559	6773,374	7496,26	**6294,731**	2875,597
fumarylace	1626,982	1246,91	1348,919	**1407,603**	2265,101	1162,91	2071,48	**1833,164**	468,2748
Aminotrans	7135,506	6539,264	6378,128	**6684,299**	8889,566	7936,254	9215,036	**8680,285**	9798,956
Stress resp	3095,021	3537,553	2737,89	**3123,488**	1019,419	3270,649	921,0887	**1737,052**	200,2417
Mannose-b	18346,4	17103,38	18551,3	**18000,36**	17721,45	19928,23	18216,69	**18622,12**	15174,75
SPFH/Band	3055,035	2891,467	2914,824	**2953,775**	3325,82	3897,842	3882,204	**3701,955**	4107,239
DEA(D/H)-b	2079,711	3019,003	2698,187	**2598,967**	2524,346	3185,594	1960,403	**2556,781**	861,0245
IMPA-6 imp	54,69849	179,6315	298,7139	**177,6813**	830,0447	79,7164	73,95681	**327,906**	11583,35
PGDH D-3-p	15572,54	12831,34	13827,33	**14077,07**	12571,39	11730,31	12200,14	**12167,28**	9871,294
LAZ5 Disea	2963,781	3967,083	5061,577	**3997,48**	4165,768	3226,485	7596,631	**4996,295**	1294,383
TIF3B1, EIF	13818,45	13541,18	13131,61	**13497,08**	11852,64	14836,44	12851,22	**13180,1**	8505,532
RAB6, ATRA	10565,36	8664,481	11037,73	**10089,19**	10987,07	13050,74	13087,98	**12375,26**	19051,61
HAD superf	2210,327	3705,696	4148,665	**3354,896**	13948,23	18667,44	18747,38	**17121,01**	7996,576
Ribosomal	0	0	0	**0**	13,79199	962,4271	759,3125	**578,5105**	0
SDH1-2 suc	18223,82	20409,22	19707,47	**19446,84**	15494	14174,08	14852,73	**14840,27**	16590,63
ATLFNR2, F	2985,496	4466,55	3598,05	**3683,365**	6065,354	4829,56	6100,756	**5665,223**	7084,075
EDA39 caln	2488,507	2306,079	2508,766	**2434,451**	3682,876	3199,353	2737,761	**3206,663**	5543,724
Emsy N Ter	683,0874	1375,186	676,2804	**911,5179**	1054,967	840,2087	1660,363	**1185,18**	197,6625
RPL10B Rib	8290,407	10266,07	9750,277	**9435,585**	11797,33	10255,58	10960,25	**11004,39**	13391,57
LHB1B2, LH	696,6259	963,4202	696,0028	**785,3496**	874,3026	1025,187	1496,282	**1131,924**	3694,219
RHD3 Root	1700,871	2979,184	1119,875	**1933,31**	2158,125	1538,968	690,0872	**1462,394**	259,248
Nucleic aci	6355,469	5767,081	7659,515	**6594,022**	7809,351	10123,76	10393,62	**9442,242**	5227,556
Zincin-like	1550,995	1989,707	1703,43	**1748,044**	2384,56	2290,947	2639,888	**2438,465**	2494,02
Ribosomal	10502,33	10694,03	9488,803	**10228,39**	12347,93	11787,66	12946,37	**12360,66**	4296,896
HXK3 hexo	4218,978	4969,271	5227,486	**4805,245**	5246,683	5838,355	6971,686	**6018,908**	7782,14
TIF3H1 trar	4116,615	3333,507	3479,706	**3643,276**	3701,515	2549,149	3200,512	**3150,392**	1924,235
BEST Arabi	956,6566	746,7064	654,3658	**785,9096**	1767,701	3485,148	3285,591	**2846,147**	1173,397
Glycosyl hy	66147,37	46051,25	40401,6	**50866,74**	49572,1	42838,62	66938,64	**53116,46**	245593,8
ATNUDT6,	6601,15	5778,099	7195,963	**6525,07**	4114,308	5597,724	4433,009	**4715,014**	3474,672
ATSERAT2;	7073,966	7784,39	8799,109	**7885,822**	6349,295	10503	6796,268	**7882,855**	2875,261
TT5, A11, C	3812,991	3896,869	3912,475	**3874,111**	7166,01	4739,959	6985,788	**6297,252**	9403,475
CAM5 calm	2247,413	1333,755	1286,2	**1622,456**	1225,776	1807,916	916,1943	**1316,629**	9799,839
Peroxidase	4216,518	5073,321	3687,322	**4325,72**	4393,112	7370,969	4873,206	**5545,762**	10733,5
Cysteine pr	14281,72	16087,13	15838,94	**15402,6**	20645,44	17412,09	19309,76	**19122,43**	21006,12
AGD2 Pyric	9142,822	11331,63	11910,91	**10795,12**	15554,37	19737,79	20505,58	**18599,25**	12696,74
Class I gluta	6506,273	7193,71	6356,628	**6685,537**	7846,674	7994,905	7647,337	**7829,639**	7823,169
TUB9 tubul	4482,399	4897,363	4849,26	**4743,007**	4936,906	4170,258	5244,094	**4783,753**	3484,172
DHS1 3-dec	5531,342	4794,419	4399,42	**4908,394**	5278,181	4876,685	5613,436	**5256,101**	7145,499
Peroxidase	7478,216	8178,741	8431,656	**8029,538**	11356,14	17206,42	14039,31	**14200,62**	9717,235
FLA1 FASCI	1565,812	2236,312	2289,484	**2030,536**	2962,886	3044,421	3243,887	**3083,732**	4012,744
Succinyl-Co	8215,362	9575,529	9636,769	**9142,554**	5138,151	8053,724	4774,214	**5988,696**	15068,08
Peroxidase	1270,065	1611,744	1453,344	**1445,051**	1037,385	1012,513	194,3713	**748,0899**	142,9048
6-phosphog	19893,46	21296,85	18940,86	**20043,72**	16991,44	16534,08	16646,18	**16723,9**	18782,07

EMB1144 c	18445,83	20383,87	19408,08	**19412,59**	26825,91	22850,89	24062,31	**24579,7**	24703,23
ALDH7B4 a	17930,42	17912,49	18825,33	**18222,75**	20461,6	16722,89	18704,96	**18629,82**	14151,01
ADH1, ADH	5334,741	5258,638	5503,148	**5365,509**	7223,287	9264,627	8511,248	**8333,054**	12803,44
GAD, GAD1	49798,15	58992,36	55576,7	**54789,07**	69403,11	46886,8	59138,43	**58476,11**	34186,35
IVD, ATIVD	7693,114	13859,88	12970,09	**11507,69**	5706,999	5884,748	5505,333	**5699,027**	5405,842
receptor-lil	2853,65	2776,713	2504,519	**2711,627**	2604,973	2904,634	2316,649	**2608,752**	1359,787
ATP citrate	56517,75	53866,54	67034,17	**59139,49**	73710,4	71455,64	72843,19	**72669,74**	56173,98
ATRABG3F,	2218,788	1719,367	1659,93	**1866,028**	904,7284	1298,381	854,8266	**1019,312**	606,9748
SAG2, AALF	35278,7	29247,62	30554,27	**31693,53**	32292,6	31233,89	20552,02	**28026,17**	16679,35
ATTPS6, TP	25617,77	20885,59	21857,51	**22786,96**	17579,94	20795,57	18066,15	**18813,89**	15728,78
Ribosomal	9984,781	9754,093	10667,46	**10135,45**	12348,04	26274,56	23209,52	**20610,7**	6661,464
NFA02, NFA	9312,106	7902,708	6288,811	**7834,541**	6494,716	8212,485	7683,642	**7463,614**	4287,069
ketose-bisp	58827,15	52531,56	53855,47	**55071,39**	45118,41	50196,51	45197,91	**46837,61**	43283,02
Ribosomal	66,08306	232,5594	292,4367	**197,0264**	937,4875	1503,163	1595,418	**1345,356**	232,1277
GDSL-like L	823,9087	757,8693	614,0189	**731,9323**	1205,094	1854,69	1440,956	**1500,247**	883,9527
ARR5, ATRR	6770,66	7410,715	5794,771	**6658,715**	4555,182	5037,954	4227,26	**4606,798**	5200,106
ATFRUCT5,	3865,961	3278,068	4209,828	**3784,619**	2591,795	3349,717	2281,764	**2741,092**	2233,548
PAL2, ATPA	210,6489	172,1943	177,5997	**186,8143**	605,0655	265,5627	568,5952	**479,7411**	72,86003
ASP4 aspar	5415,465	7044,935	7658,326	**6706,242**	6852,714	6598,224	6673,192	**6708,044**	12364,72
VHA-A3 vac	11084,05	12603,84	11542,25	**11743,38**	15770,47	16771,12	13328,45	**15290,02**	9201,091
GLU1, GLS1	9768,484	7704,786	7660,22	**8377,83**	8438,287	9046,892	6721,459	**8068,879**	4239,963
TUB7 tubul	9810,331	10433,95	10324,43	**10189,57**	10460,66	11388,21	12763,65	**11537,51**	8768,868
sks17 SKU5	2619,806	2095,368	1639,948	**2118,374**	3014,383	1305,309	2781,468	**2367,053**	7379,41
RNA-bindin	869,8724	604,6324	750,6032	**741,7026**	1063,411	1167,215	1472,152	**1234,26**	1250,615
Protein of u	1146,865	854,4566	925,4489	**975,59**	528,6367	601,528	324,4262	**484,8636**	337,6694
DEAD box F	197,5674	190,667	243,8806	**210,705**	538,2181	118,6413	645,3992	**434,0862**	31,31049
GroES-like	9642,565	9465,148	6768,295	**8625,336**	3160,706	5563,679	2717,726	**3814,037**	3874,678
D-mannose	20380,15	21562,72	24535,67	**22159,52**	13641,13	15144,07	8714,993	**12500,07**	12251,43
PEP7 elicito	2133,536	1951,211	1967,831	**2017,526**	2029,625	1621,682	1604,423	**1751,91**	2613,706
ATCNGC6,	3354,038	3258,725	3440,534	**3351,099**	2880,09	4020,227	3588,717	**3496,345**	2328,018
RGP3, RGP	1468,423	914,0123	1019,678	**1134,038**	764,9038	617,1308	770,6955	**717,5767**	1268,927
unknown p	1433,481	1755,289	1799,479	**1662,75**	1996,087	1812,072	1649,534	**1819,231**	2521,184
SPFH/Band	1286,335	1021,631	1656,333	**1321,433**	1284,296	2091,614	1729,176	**1701,695**	805,692
AVA-P4 vac	50488,15	45896,12	56072,45	**50818,9**	47802,98	59353,83	72510,49	**59889,1**	36970,16
AIR12 auxir	5289,135	4860,927	4999,782	**5049,948**	6168,51	5876,172	6800,331	**6281,671**	8296,749
Actin family	4664,004	3829,252	4160,662	**4217,973**	2709,812	3107,967	1841,51	**2553,096**	2241,543
Ribosomal	2734,167	2504,605	3425,954	**2888,242**	1566,787	3120,351	347,9013	**1678,347**	265,5598
CYT1 Gluco	9793,719	11167,12	9413,182	**10124,67**	12178,74	9146,978	11919,22	**11081,64**	7189,172
BTB/POZ do	1849,162	1914,778	1762,121	**1842,021**	1314,951	3741,5	695,924	**1917,458**	272,9044
Thioredoxir	20493,29	19358,38	19054,15	**19635,28**	17537,31	15313,63	15739,51	**16196,82**	17671,77
NAXT1 nitra	2454,707	2286,791	1752,33	**2164,609**	4143,263	2507,888	3114,587	**3255,246**	4382,551
ATTPS5, TP	1784,722	2098,832	1835,802	**1906,452**	1824,257	2334,232	2163,69	**2107,393**	3653,785
RPN1A, ATI	27972,64	33116,09	29775,74	**30288,15**	37602,05	26226,72	29999,09	**31275,95**	19814,19
ATGSTT1, G	6610,399	4787,507	5386,485	**5594,797**	2556,182	4761,466	2312,694	**3210,114**	2204,659
Ribosomal	2829,104	3491,847	4736,055	**3685,669**	3153,816	5953,271	2557,248	**3888,112**	1294,641
ATAAT, AAT	14509,43	15717,28	18284,18	**16170,3**	12484,15	10126,8	10986,84	**11199,26**	10271,32
Protein pho	2967,205	2801,877	2423,504	**2730,862**	2951,686	3752,585	2068,975	**2924,415**	6733,402
Coatomer,	14056,39	12608,06	11585,46	**12749,97**	10050,39	14723,8	8217,498	**10997,23**	6605,936
TPX2 thiore	3923,9	4603,204	5273,024	**4600,043**	3067,776	5764,626	3219,193	**4017,198**	1662,549
AGT, AGT1,	15908,9	15780,99	18590,08	**16759,99**	12472,38	11522,54	11623,79	**11872,9**	15590,71

ARM repea	2730,789	3973,366	4012,257	**3572,137**	1169,496	4094,885	668,8406	**1977,741**	432,4035
ATKTI1, KTI	46311,38	37333,82	40445,37	**41363,53**	25968,51	38143,74	25687,86	**29933,37**	66654,5
Aldolase-ty	19873,63	17040,02	21179,64	**19364,43**	17132,97	15920,4	15129,7	**16061,02**	14654,6
unknown p	361,3496	369,969	380,1014	**370,4733**	412,2681	438,1205	363,5214	**404,6367**	484,6999
Saccharopi	15780,67	11621,37	11100,97	**12834,34**	5422,585	9875,732	4350,839	**6549,719**	5017,658
HSP70, ATH	13047,39	15475,68	23066,79	**17196,62**	13052,56	13703,31	9371,884	**12042,59**	7915,737
GRF12, GF1	10688,49	11335,05	13705,97	**11909,84**	10143,42	12592,7	15923,18	**12886,43**	5896,878
Isocitrate/i	61326,8	58053,37	58562,59	**59314,25**	38073,74	51288,19	36964,63	**42108,85**	44138,36
ATGLDH, G	384,3892	185,6638	269,5292	**279,8607**	680,7354	465,7226	655,8722	**600,7767**	540,4403
ATGDI1, AT	39464,38	31149,15	29266,96	**33293,5**	25125,39	45645,36	19297,98	**30022,91**	12637,79
CLPP5, NCL	4509,658	3937,482	3767,852	**4071,664**	3786,641	3759,579	3558,875	**3701,698**	4520,725
60S acidic r	9523,473	8921,427	10289,36	**9578,087**	16905,93	14762,98	16474,37	**16047,76**	13697,7
Lactate/ma	16412,73	26057,78	16494,58	**19655,03**	18152,75	14684,73	14059,3	**15632,26**	9805,584
Pyridoxal-d	521,9326	644,0563	819,5379	**661,8423**	1583,718	1850,925	3296,244	**2243,629**	945,0093
ATH9, TRX	433,8668	531,6399	426,417	**463,9746**	314,8257	303,6493	157,7599	**258,7449**	531,914
RHS19 root	5852,369	2884,987	3804,761	**4180,706**	7263,791	15197,61	14093,18	**12184,86**	5170,209
RBCL ribulo	54373,45	44256,14	40572,9	**46400,83**	82698,02	46383,26	75911,27	**68330,85**	136379,2
Peroxidase	1538,861	2558,831	2448,526	**2182,072**	4700,872	12924,9	10699,3	**9441,691**	2224,9
Transducin	11707,98	10036,22	10257,28	**10667,16**	11641,42	17646,91	13551,64	**14279,99**	22833,62
Chaperone	947,3236	541,2403	785,9867	**758,1835**	1459,646	998,686	1324,474	**1260,935**	1809,067
Class-II DAH	22615,38	22827,77	25750,89	**23731,34**	25207,28	28218,71	26907,86	**26777,95**	21155,24
PAL4 pheny	11974,68	16158,06	16844,98	**14992,57**	8038,454	13338,52	11502,38	**10959,78**	6449,118
Glycosyl hy	4297,728	3198,23	3439,486	**3645,148**	1756,516	2998,618	2373,394	**2376,176**	1253,454
6-phosphog	9295,367	9778,452	10569,26	**9881,026**	7045,583	6054,573	4313,118	**5804,425**	7703,143
Ribosomal	229,624	399,1733	318,1596	**315,6523**	610,0049	798,9478	744,8715	**717,9414**	488,8788
RD19, RD19	3275,435	3482,194	3637,797	**3465,142**	3157,017	3822,791	5004,623	**3994,81**	2413,88
UGD1, ATU	1995,147	1170,63	1777,333	**1647,703**	1287,403	1742,348	785,5415	**1271,764**	4649,734
Cytokine-in	21991,82	31812,29	24036,6	**25946,91**	33398,39	27610,06	30502,53	**30503,66**	20076
Glycoprote	4251,918	2583,731	3050,92	**3295,523**	2391,843	2898,73	2422,354	**2570,975**	1836,248
glycyl-tRNA	10700,41	14037,87	12547,16	**12428,48**	20002,32	18338,23	23630,45	**20657**	17790,38
TRAF-like fa	50478,11	49613,44	52167,53	**50753,03**	53050,54	50703,51	55280,77	**53011,6**	59108,23
Copper tra	1650,032	3848,763	3115,294	**2871,363**	2992,561	4530,677	2842,781	**3455,34**	595,8306
IIL1, ATLEU	57187,73	58407,84	54040,27	**56545,28**	73054,91	63016,88	75687,86	**70586,55**	62938,22
ADL1C, ADL	3506,796	4876,739	4235,171	**4206,235**	3663,668	5772,751	3138,828	**4191,749**	1924,594
SKS6 SKU5-	4512,333	4256,879	4585,331	**4451,514**	5446,557	8387,673	8745,657	**7526,629**	4748,206
ATCLPC, AT	14690,8	15897,88	16991,07	**15859,92**	10326,33	13959,31	12049,38	**12111,67**	12173,44
EMB2296 F	3501,565	5752,978	6384,346	**5212,963**	4642,706	10709,35	7954,891	**7768,982**	2696,612
PYD1 pyrim	39432,5	34006,38	39120,23	**37519,7**	46491,31	32387,17	43413,95	**40764,14**	27464,43
AAC1 ADP/	153800	146004,2	150586,4	**150130,2**	197286,7	140342,6	216567,9	**184732,4**	121601
Calcium-bin	382,0992	147,2573	224,4502	**251,2689**	23,80364	217,2312	23,87502	**88,30328**	21,02743
atnudt3, NU	3422,59	3261,971	1995,933	**2893,498**	1863,251	1808,056	1524,302	**1731,87**	748,539
SPY Tetratr	809,3736	691,1264	744,1385	**748,2128**	1035,416	913,6939	1004,783	**984,631**	1002,462
alpha/beta	318,0413	463,3455	560,0541	**447,147**	812,4451	436,8492	622,082	**623,7921**	951,6365
P-loop cont	691,1537	703,5997	1067,483	**820,7454**	1363,141	1336,911	1794,756	**1498,269**	868,3619
SYTE, ATSY	1556,995	954,0425	1096,081	**1202,373**	851,9054	686,3735	935,7879	**824,6889**	1486,87
Zn-depende	4389,406	4420,195	3481,127	**4096,909**	7175,977	4605,796	6768,153	**6183,309**	6563,581
Nascent po	6978,838	7229,733	7501,986	**7236,853**	6122,943	7736,037	6309,85	**6722,943**	5402,776
HSP60, HSP	72584,16	65485,16	65800,49	**67956,6**	78690,32	82014,99	86812,4	**82505,9**	72967,04
TPP2 tripep	47262,05	52325,18	49030,94	**49539,39**	41472,85	52678,68	43555,19	**45902,24**	36355,03
APK, AKN1,	3480,753	2521,132	3686,786	**3229,557**	2740,498	2546,003	2842,469	**2709,657**	2064,926

MLP28 MLI	428,2649	972,4104	421,1393	**607,2715**	772,0423	1731,51	769,6055	**1091,053**	267,5105
Phosphogly	22951,82	22351,33	25511,24	**23604,8**	33289,23	27576,37	32915,39	**31260,33**	32803,97
AMP-deper	69797,2	75532,41	75097,52	**73475,71**	58909,17	67017,59	60294,15	**62073,64**	72742,3
ATPPC3, PF	15759,15	10523,45	9930,419	**12071,01**	10174,96	13038,45	10248,68	**11154,03**	6768,938
AtHip1, HIF	13053,97	10010,22	11494,36	**11519,52**	9286,143	8471,476	8773,306	**8843,642**	6880,242
BTR1, BTR1	17059,59	18836,53	19883,72	**18593,28**	16316,74	16343,53	17038,41	**16566,23**	15109,08
Nucleotidy	727,5626	7,576787	9,022951	**248,0541**	21,33316	300,9773	713,7011	**345,3372**	0
SYP43, ATS	9096,159	5815,072	6757,893	**7223,041**	8305,283	9453,738	9267,059	**9008,693**	14141,53
Subtilase fa	12072,98	9983,604	10131,68	**10729,42**	12826,07	16247,64	16587,44	**15220,38**	12688,75
pfkB-like ca	25638,59	25978,16	24736,27	**25451,01**	15150,92	21550,59	13915,7	**16872,4**	13271,86
EIF3K, TIF3	3740,874	5041,415	5564,833	**4782,374**	5977,783	4736,527	6360,818	**5691,709**	3513,043
ATXYL1, XY	18458,49	13317,05	14387,78	**15387,77**	19795,8	24790,69	31872,92	**25486,47**	15525,85
ATGLX1, GL	82144,3	69496,48	65233,46	**72291,41**	69974,13	72693,22	79325,89	**73997,75**	56287,89
GRF8, 14-3	49171,45	44915,56	52216,27	**48767,76**	27979,24	48402,64	21655,93	**32679,27**	23317,5
AHA5, HA5	11272,04	9642,459	10015,8	**10310,1**	10114,01	10132,15	10027,44	**10091,2**	8843,988
Aluminium	2699,511	6405,745	5610,675	**4905,31**	2345,021	3519,793	2743,939	**2869,584**	1668,993
TCP-1/cpn6	12056,6	13269,16	15589,91	**13638,55**	17180,29	12727,89	16155,39	**15354,52**	18853,94
VHA-E3 vac	805,9178	1210,41	1265,491	**1093,94**	5066,64	1510,122	3429,947	**3335,57**	3243,469
DEAD/DEAI	2081,842	4420,8	1996,253	**2832,965**	5006,613	8031,074	9597,459	**7545,049**	3526,845
Ribosomal	3766,873	3357,552	4088,961	**3737,795**	8025,058	14527	18635,65	**13729,24**	2602,789
CAS1 cyclo	1792,051	2535,643	2676,702	**2334,798**	3523,697	2642,806	4317,662	**3494,722**	4347,216
ATGR1, GR	76202,28	81616,24	67331,46	**75049,99**	64413,11	65698,82	52548,3	**60886,74**	56852,57
THO5, AtTH	3747,141	3087,105	3431,149	**3421,798**	2234,789	2887,232	2007,484	**2376,502**	3216,098
ICDH isocit	9829,988	6445,278	6968,701	**7747,989**	3614,763	9936,097	4184,265	**5911,708**	2545,737
NAD(P)-link	1639,542	842,1008	1106,544	**1196,062**	1698,519	2022,196	1198,473	**1639,729**	609,7013
MD-2-relat	6300,228	5000,55	5795,641	**5698,806**	4015,341	4721,177	3800,336	**4178,952**	4769,541
NDB3 NAD	224,4254	198,8547	288,652	**237,3107**	194,5643	136,2333	247,8683	**192,8886**	586,7808
CUE1, PPT,	13569,81	10663,48	11500,25	**11911,18**	8207,382	10227,49	7222,163	**8552,345**	8627,49
emb1027 A	4767,385	4261,469	4156,569	**4395,141**	5249,847	3289,99	5317,231	**4619,023**	2747,346
PAE2 20S p	1011,733	461,112	459,007	**643,9507**	300,9725	267,8293	191,4271	**253,4096**	670,4187
Ribosomal	8473,198	7262,381	7904,817	**7880,132**	11031,24	18947,12	24370,91	**18116,42**	8131,597
PFK7 phosp	1365,299	1569,841	1358,443	**1431,194**	1485,651	1953,116	1228,847	**1555,871**	816,3173
MEE51 Pho	2976,657	726,0718	1048,464	**1583,731**	408,1096	224,4213	293,9021	**308,811**	709,2106
Eukaryotic	117,5982	327,0999	330,4808	**258,393**	777,1872	621,2606	1400,484	**932,9772**	476,6199
ALAAT2 ala	29,36347	286,3589	252,9931	**189,5718**	212,5938	328,5895	566,0758	**369,0864**	1762,92
ATMPK7, M	76,62741	59,74176	73,66206	**70,01041**	328,5224	58,6801	192,995	**193,3992**	277,126
Mitochond	91544,14	81088,22	87428,13	**86686,83**	86126,58	79443,74	95765,59	**87111,97**	73788,88
GMD1 GDP	47,49365	92,84246	131,7957	**90,71059**	49,34	15,8458	59,14944	**41,44508**	172,4539
ATMLP-300	22458,11	15469,94	16847,24	**18258,43**	12542,08	17489,39	12360,59	**14130,69**	10001,79
Galactose r	1543,697	1280,817	1165,985	**1330,166**	1219,873	817,9537	1396,22	**1144,682**	791,1741
P5CS2 delta	596,2679	575,3082	568,972	**580,1827**	1185,989	734,1635	900,3917	**940,1814**	693,162
PBE1 20S p	48288,72	50082,04	53769,2	**50713,32**	47676,81	43182,34	53061,54	**47973,56**	42196,82
Mannose-b	3470,344	3020,095	3804,498	**3431,646**	2288,848	3993,436	2347,547	**2876,61**	1828,389
ASAR1, ATS	815,4578	596,6588	432,7825	**614,9664**	1559,358	868,8016	1346,016	**1258,059**	1343,873
HCT hydrox	893,4288	580,0527	715,9586	**729,8133**	470,3402	426,6142	304,7664	**400,5736**	581,3173
CHY1 beta-	906,5092	381,5309	381,0475	**556,3625**	860,5774	438,2194	611,4574	**636,7514**	164,6318
EIF4A1, RH	11778,57	13733,81	15236,02	**13582,8**	11343,2	17230,78	13686,55	**14086,84**	9044,519
unknown p	137,9162	989,0243	1119,803	**748,9144**	1926,946	648,4067	1448,133	**1341,162**	6678,293
GAD2 gluta	4316,461	2844,569	3709,634	**3623,555**	5427,852	4785,258	6824,787	**5679,299**	5240,357
Tyrosine tra	798,6576	1002,068	1081,281	**960,6687**	608,0735	363,1639	686,1146	**552,4507**	406,1582

Glycosyl hy	25175,25	23237,29	23586,3	**23999,61**	19845,78	24582,09	24534,64	**22987,5**	19832,16
Pectin lyase	208,4883	292,6615	545,1307	**348,7602**	554,8135	331,0251	546,5768	**477,4718**	226,0655
FAD-bindin	4770,481	5760,654	4207,667	**4912,934**	6402,104	12568,91	9318,494	**9429,835**	5742,873
O-methyltr	1549,623	1543,257	1523,524	**1538,801**	1440,287	704,9074	1126,599	**1090,598**	1996,275
AT-E1 ALPH	3989,586	4462,22	3859,391	**4103,732**	3025,61	3966,324	3271,789	**3421,241**	3273,345
NDPK1 Nuc	74981,62	74890,17	73638,1	**74503,3**	71915,03	64339,38	85638,35	**73964,26**	95661,68
DPE2 dispro	10814,24	13299,56	15295,25	**13136,35**	13451,99	15375,85	13722,02	**14183,29**	10644,79
AVP1, ATAV	14464,6	10407,07	12585,61	**12485,76**	13014,14	17123,59	13974,64	**14704,12**	11060,78
BGLU21 Gly	169224,3	170112,8	172095,8	**170477,6**	100043,5	205978,9	63192,3	**123071,6**	62629,23
Protein of u	11672,58	7850,747	9786,381	**9769,901**	4046,426	5510,822	1781,358	**3779,535**	3367,674
ASHH3, SDG	2713,457	1575,854	1767,189	**2018,833**	667,483	1347,249	815,383	**943,3718**	1038,715
GAMMA-AT	29352,34	36568,27	39772,79	**35231,13**	29235,5	30276,79	37290,74	**32267,67**	21677,93
AtRLP55, R	662,7178	690,8583	645,6847	**666,4203**	800,7374	1391,339	853,956	**1015,344**	1095,174
SUR1, HLS3	1862,246	1907,728	2221,115	**1997,03**	1864,295	1662,013	2341,221	**1955,843**	2638,365
SMT3 stero	618,4831	644,3443	664,2846	**642,3707**	927,8867	2270,565	1896,415	**1698,289**	727,8645
Peroxidase	46559,41	53008,5	55370,29	**51646,06**	62760,52	92849,85	103888,9	**86499,76**	48110,88
Glycosyl hy	1012,48	1222,04	1387,378	**1207,299**	1188,766	1537,011	1785,84	**1503,872**	1838,938
Disease res	36080,01	37690,6	35201,58	**36324,07**	41285,95	46880,34	55697,21	**47954,5**	35212,01
LACS3 AMP	2014,657	1317,673	1217,415	**1516,582**	2983,937	1259,318	3297,83	**2513,695**	3630,033
ARP1, emb	9742,657	9802,924	9515,74	**9687,107**	25846,02	39304,99	50912,23	**38687,75**	23838,22
MUR1, MU	16475,57	15175,31	17111,73	**16254,2**	10868,85	13338,46	10224,12	**11477,14**	12989,66
O-methyltr	624,7147	3034,255	2057,001	**1905,323**	1561,899	82,77275	63,83535	**569,5025**	5268,573
BIP3 Heat s	19889,05	15799,59	17681,04	**17789,89**	12259,81	14840,83	9598,642	**12233,1**	12385,51
TSA1 TSK-a	729,5949	951,4505	368,9854	**683,3436**	71,16781	599,3006	65,61139	**245,3599**	43,36667
NSF AAA-ty	23424,84	23374,94	21191,23	**22663,67**	24069,87	19629,17	25189,43	**22962,82**	19224,05
Mitochond	2268,125	1963,771	2501,099	**2244,332**	2774,636	1714,783	1728,874	**2072,764**	1565,32
COX2 cytoc	1485,681	1260,303	1453,933	**1399,973**	966,3184	1237,321	809,7303	**1004,457**	962,8162
PFK1 phosp	16194,91	16573,19	11952,09	**14906,73**	10286,93	8003,659	9837,276	**9375,954**	8608,184
WIN1 HOPV	6044,692	4930,558	5135,734	**5370,328**	3974,325	4623,8	3786,618	**4128,248**	4791,278
PAA1 prote	22254,99	22084,76	22322,39	**22220,71**	21024,94	22600,27	20817,7	**21480,97**	23536,42
MAC3B MO	845,8234	778,6248	605,2182	**743,2221**	561,1228	644,8475	519,6791	**575,2165**	509,1952
Phosphogly	3327,224	1970,149	1939,624	**2412,333**	4690,377	1863,572	4158,548	**3570,832**	1030,764
HTB4 Histo	7662,159	8444,816	8877,538	**8328,171**	15318,19	30944,95	31456,08	**25906,41**	3676,728
FUM1 fuma	23098,43	21687,65	23485,39	**22757,16**	20995,63	17124,47	17490,25	**18536,79**	21002,28
IDH-VI isoc	5077,22	4982,291	5103,416	**5054,309**	4480,186	3602,067	3082,16	**3721,471**	4413,015
ATPB, PB A	19154,03	18337,2	17318,47	**18269,9**	13290,97	19005,83	8243,532	**13513,44**	8299,682
alpha/beta	1122,485	1600,588	1200,309	**1307,794**	1602,219	1424,479	2213,862	**1746,854**	986,5608
ATGCN1, G	3300,132	3506,03	3875,594	**3560,585**	5721,041	4074,557	5606,903	**5134,167**	3764,625
Thiamine p	36751	34929,9	38341,4	**36674,1**	24282,39	38314,41	24953,56	**29183,45**	18991,33
Serine carb	9557,856	14136,59	11858,28	**11850,91**	15109,37	17454,3	17531,16	**16698,28**	13282,32
SHM5, EDA	72,72538	133,7623	285,9921	**164,1599**	817,084	329,904	811,8416	**652,9432**	221,6046
PAE1, ATPA	1789,25	1049,741	1153,373	**1330,788**	729,3787	574,5726	588,7251	**630,8921**	1151,134
ATNADP-M	20099,62	31277,11	30554,34	**27310,36**	18939,92	27616,64	26080,78	**24212,45**	12237,85
AAPT1, ATA	450,4486	450,1178	585,0868	**495,2177**	753,9777	647,8535	992,202	**798,0111**	603,2281
AtRABA1g,	106,302	164,4796	148,7484	**139,8433**	121,7094	109,6199	71,19804	**100,8424**	60,61595
ATNADP-M	1121,867	2789,091	3614,461	**2508,473**	364,5705	786,3944	112,366	**421,1103**	544,1181
ASP5, AAT3	44527,75	36424,01	44767,64	**41906,47**	31691,77	34601,9	31797,18	**32696,95**	32536,25
ATPUB14, P	8365,592	15825,71	11415,89	**11869,07**	7959,564	16342,93	14236,16	**12846,22**	5514,34
GRF7, GF14	14,94894	86,58597	23,91209	**41,81566**	76,55368	78,41535	61,11413	**72,02772**	128,1103
4CL1, 4CL.1	4657,719	5637,586	4236,426	**4843,91**	5706,821	5066,71	5585,737	**5453,09**	6236,932

ACD32.1, A	6913,204	2194,485	3772,104	**4293,264**	1539,379	5803,161	1003,864	**2782,135**	687,3401
TUB6 beta-	6337,107	8162,108	6860,376	**7119,864**	8222,651	9972,694	11564,41	**9919,919**	7228,133
Lactate/ma	274115,9	231545,7	211763	**239141,5**	246224,7	234417	355832,1	**278824,6**	186528,5
RPS6, RPS6	776,1784	1003,112	1211,629	**996,9729**	1423,574	1710,169	2573,59	**1902,444**	1189,524
Pyridoxal p	10748,41	10970,78	10710,8	**10810**	13183,77	9823,834	14543,59	**12517,06**	14944,93
structural r	13935,78	14723,01	13521,88	**14060,23**	13778,79	13527,71	13524,51	**13610,34**	12331,03
Lipase/lipo	26013,59	23604,63	16197,43	**21938,55**	29000,96	37738,09	49204,93	**38647,99**	23087,65
ACLB-2 ATF	9265,476	12430,25	11971,31	**11222,34**	9589,268	8868,136	8936,424	**9131,276**	8626,533
ATPDIL1-2,	6301,741	6268,91	6022,262	**6197,638**	5679,331	5973,274	7670,948	**6441,184**	7814,526
ATCXE12, C	2716,368	2101,549	2191,246	**2336,388**	2614,662	1979,363	2338,366	**2310,797**	1617,828
GL22 germ	952,1069	1333,406	1781,474	**1355,662**	3746,256	1073,014	4446,085	**3088,452**	4728,911
CAD5, ATCA	25727,59	29050,4	26738,94	**27172,31**	24897,81	21754,1	19778,58	**22143,49**	24940,87
APT1 adeni	1060,337	2986,904	2534,861	**2194,034**	2374,524	1881,666	509,3782	**1588,523**	493,6002
RGP1, ATR(	79459,07	73456,25	72703,44	**75206,25**	86517,6	60998	72639,99	**73385,19**	88601,9
RNA-bindin	3241,704	2154,581	1802,065	**2399,45**	1598,841	2206,87	847,3517	**1551,021**	998,8794
NMT1, ATN	10750,97	9016,162	9171,88	**9646,338**	8659,273	7648,781	8518,423	**8275,492**	6800,299
Ribophorin	4111,123	4901,549	4854,655	**4622,442**	5700,481	6496,328	5893,467	**6030,092**	4744,415
Pentatricop	207,8799	394,0233	109,5988	**237,1673**	485,2844	276,9774	490,2534	**417,5051**	168,9512
Protein of u	5418,007	1627,986	1545,411	**2863,801**	3122,996	815,7753	4892,09	**2943,62**	23487,98
RPT5A, ATS	19102,02	21014,45	21143,87	**20420,12**	22743,6	16485,05	27088,08	**22105,57**	28418,89
MTHFR1 m	95961,28	104831,9	109366,8	**103386,7**	132033,3	93446,27	123643,5	**116374,3**	82297,89
GHMP kina	9614,076	7201,057	8298,732	**8371,288**	6685,236	9840,705	6323,25	**7616,397**	5173,622
Aldolase su	9694,771	6366,236	7675,399	**7912,135**	4792,088	6855,218	4915,578	**5520,961**	4997,936
ATHXK2, HX	10471,94	11880,08	10956,39	**11102,81**	6280,97	9139,72	3489,366	**6303,352**	13724,31
GLP5 germ	22417,84	15870,09	23227,86	**20505,26**	10380,27	14906,44	12254,47	**12513,73**	15159,48
Coatomer,	10171,24	12594,74	11652,74	**11472,91**	12763,09	14509,73	16923,42	**14732,08**	11516,58
UGT71C1 U	15342,03	13538,44	15847,05	**14909,17**	8748,94	20443,33	12582,75	**13925,01**	7246,593
26S protea	143408,5	150410,5	120228,9	**138016**	86264,19	107827,6	62869,18	**85653,65**	113748,5
Transducin	114,4752	156,321	123,3278	**131,3747**	41,57929	67,63611	13,63106	**40,94882**	63,2901
sks15 SKU5	2457,675	5259,489	3924,976	**3880,713**	7753,804	6143,221	10091,99	**7996,337**	5772,323
DEA(D/H)-b	4995,836	5658,185	5987,732	**5547,251**	4553,062	4764,669	4330,489	**4549,407**	4059,467
ATPK3, PK3	1254,191	2331,612	2125,471	**1903,758**	4383,576	2512,859	5181,165	**4025,867**	3563,815
BGLU36 be	24035,3	8040,318	7953,147	**13342,92**	2728,544	20510,44	6509,541	**9916,174**	1581,943
Glycine clea	7186,685	6560,594	7370,161	**7039,147**	4822,027	6665,57	4203,088	**5230,228**	5116,929
thioredoxin	922,2538	335,2802	613,8424	**623,7921**	545,3151	859,9222	903,6894	**769,6422**	257,8325
ATDGK3, D	3246,031	2971,77	3271,628	**3163,143**	3034,886	2428,538	2754,028	**2739,15**	3244,865
ALDH2B7, A	7905,13	11143,76	12288,56	**10445,82**	8662,71	6486,803	6226,64	**7125,384**	10492,39
Zinc-bindin	6701,849	9340,894	7934,649	**7992,464**	7945,726	5847,854	7402,268	**7065,283**	5612,908
Leucine-ric	609,7374	434,1862	569,6783	**537,8673**	309,1771	385,7852	350,2449	**348,4024**	349,6516
SNL5 SIN3-	9300,714	8979,013	10404,27	**9561,331**	8352,489	7130,019	9112,227	**8198,245**	7677,539
SIP1 Raffino	14764,4	16713,58	14682,83	**15386,94**	20118,85	15192,7	18231,67	**17847,74**	21048,57
Ribosomal	184,3855	62,79776	43,0482	**96,74381**	60,76643	585,6328	469,1826	**371,8606**	33,21587
RmlC-like c	645,0614	984,5676	1350,487	**993,3721**	1069,936	993,6713	1640,895	**1234,834**	2260,679
unknown p	24613,13	30292,38	16031,38	**23645,63**	22132,43	28763,35	32843,91	**27913,23**	16486,14
HXXXD-typ	2411,542	1419,275	1880,412	**1903,743**	2915,626	2831,018	2864,3	**2870,315**	2790,061
GDCH glyci	308,1041	364,385	947,9015	**540,1302**	1784,647	355,9306	1976,481	**1372,353**	2409,661
HSK, DMR1	3715,293	3319,068	3871,91	**3635,424**	4416,135	2478,575	4774,791	**3889,834**	5762,813
ATPDIL1-4,	16594,89	23240,55	21184,96	**20340,14**	29044,23	23798,41	30428,7	**27757,11**	30909,75
MDH malat	90273,86	100489,6	100167,7	**96977,04**	132345,2	91854,36	124299	**116166,2**	133693,4
PIP3 plasm	44209,74	41496,63	50532,89	**45413,09**	47462,45	35294,02	46274,52	**43010,33**	36486,93

ARF3, ARL1	1691,421	1466,541	1707,662	**1621,875**	1299,9	1530,166	1881,844	**1570,637**	1134,933
Nascent po	18974,75	19437,33	21466,63	**19959,57**	15139,52	18251,21	12795,63	**15395,45**	13860,14
PRXIIF, ATP	9772	11610,07	11889,76	**11090,61**	8730,274	13406,81	12715,12	**11617,4**	15776,29
GroES-like	784,0675	630,784	1059,691	**824,8473**	1634,148	640,218	1433,795	**1236,054**	537,5034
OASB O-ace	16474,67	17015	16540,36	**16676,68**	15301,06	11786,96	12603,33	**13230,45**	13893,03
APM1, ATA	26422,86	28544,7	27767,95	**27578,5**	32842,46	27495,17	31324,03	**30553,89**	26663,96
EIF4A-2 eif	10697,2	13463,56	14812,38	**12991,04**	17822,48	12289,59	15949,54	**15353,87**	19390,89
OLI7, RPL5I	32627,45	35097,39	40025,43	**35916,76**	26068,62	39343	33932,05	**33114,56**	18588,96
NTF2A nucl	13984,05	11454,88	13963,37	**13134,1**	11932,9	9093,882	9786,533	**10271,1**	16541,54
glutamate-	617,2948	995,4022	863,3509	**825,3493**	3501,209	821,954	1488,219	**1937,127**	2950,612
Haem oxyg	3363,178	3309,875	3428,42	**3367,158**	1770,864	2500,865	732,5944	**1668,108**	1482,169
DCT, DIT2.1	749,7848	941,3923	805,3774	**832,1848**	1288,055	1061,46	1316,302	**1221,939**	1070,99
unknown p	2243,928	1576,514	1430,156	**1750,199**	2962,358	1997,767	2782,656	**2580,927**	2582,175
YBR159, KC	12247,27	10425,21	10985,52	**11219,33**	8852,859	10706,43	8121,114	**9226,799**	9292,462
Plant prote	573,9433	1517,257	3127,061	**1739,421**	7930,203	3052,223	8936,708	**6639,711**	4832,886
NAD(P)-link	398,0709	339,3896	359,4448	**365,6351**	185,2425	153,4058	231,8318	**190,16**	222,1904
AtRABA4a,	3485,238	4878,661	5466,102	**4610**	6390,832	4171,707	5663,791	**5408,777**	9230,571
ARM repea	480,6476	946,3815	836,5194	**754,5162**	960,2354	1709,718	1523,666	**1397,873**	1357,353
Ribosomal	193,6733	381,9848	536,6323	**370,7635**	872,9968	634,0909	773,5597	**760,2158**	613,4738
alpha/beta	223,2401	186,5221	290,2534	**233,3386**	211,1447	116,8577	389,3689	**239,1238**	7,515776
60S acidic r	15142,13	19337,57	16315,96	**16931,89**	18770,11	26598,83	21451,74	**22273,56**	24163,58
AP2/B3-like	1470,946	1441,006	1533,376	**1481,776**	861,6219	1333,803	772,7106	**989,3786**	1112,509
heat shock	2623,7	3248,947	2724,323	**2865,657**	3357,713	2719,022	3613,78	**3230,172**	2627,647
RPT6A, ATS	7300,795	6629,563	8040,022	**7323,46**	6238,7	4784,367	6114,929	**5712,666**	6817,682
CRA1, ATCF	2520,849	2145,603	1744,399	**2136,95**	1800,112	10850,25	10729,33	**7793,229**	1006,302
CAC3 acety	4772,347	3577,406	3325,402	**3891,718**	5598,253	4630,261	7149,078	**5792,531**	5575,346
Protein of u	18346,86	15779,73	18343,9	**17490,16**	12619,48	14340,75	8096,224	**11685,48**	10663,39
NTF2B nucl	29263,53	17659,19	20300,08	**22407,6**	17265,09	22534,89	15480,65	**18426,87**	14155,85
LOS4 P-loo	3455,934	2986,768	2168,412	**2870,371**	1536,558	2850,711	2325,317	**2237,529**	1247,292
ATS9, RPN6	29395,55	31118,2	28400,68	**29638,14**	27668,14	35651,02	33860,35	**32393,17**	22923,65
PAI3 phosp	1169,477	1554,507	1782,78	**1502,255**	961,6529	1201,265	675,9534	**946,2904**	755,8279
Eukaryotic	1885,227	2032,071	3065,513	**2327,604**	1471,871	1389,544	818,1969	**1226,538**	1015,778
NUZ, TRZ1	4207,484	2486,874	4786,41	**3826,922**	1595,029	2591,491	1269,939	**1818,82**	2609,48
UXS4 UDP-	27,32481	252,8632	58,24984	**112,8126**	488,8181	116,507	321,7376	**309,0209**	35,96278
ELM1 Prote	307,4776	427,958	330,1766	**355,2041**	790,261	810,1208	208,3875	**602,9231**	64,23559
lysine-keto	2213,223	3084,032	3476,941	**2924,732**	1803,083	1654,611	1358,296	**1605,33**	1449,077
Protein of u	697,0643	688,509	479,5785	**621,7172**	141,3452	1445,417	239,3308	**608,6977**	5,969791
Calcium-de	238,7885	156,9731	478,3188	**291,3601**	180,0843	275,5048	239,2937	**231,6276**	655,1247
AT hook m	1163,31	1417,067	1494,57	**1358,315**	536,5206	996,7864	322,2613	**618,5228**	349,2167
Sec23/Sec2	11751,12	13869,46	12453,47	**12691,35**	15336,68	11299,57	14072,04	**13569,43**	10846,97
APT2, ATAF	1111,381	197,9944	198,9419	**502,7726**	181,6585	191,7273	110,0332	**161,1397**	60,84968
RPL23AB ri	47,10333	10,35113	22,04564	**26,50003**	217,5692	51,62294	1195,839	**488,3436**	45,79313
DWF1, DIM	8693,751	8254,852	8612,083	**8520,229**	12183,89	7996,673	11734,97	**10638,51**	14504,77
Phosphogly	9835,272	9856,005	10561,07	**10084,12**	10440,4	9304,716	10546,69	**10097,27**	11180,95
tRNA synth	27108,17	21915,29	30376,27	**26466,57**	23682,73	31312,34	23946,05	**26313,71**	19967,22
PAL1, ATPA	5564,383	6765,858	6156,838	**6162,359**	7253,289	7096,422	5398,432	**6582,714**	5048,51
Ribosomal	1528,604	1812,358	1905,467	**1748,81**	1094,693	1287,886	590,9459	**991,175**	1383,145
Translation	89365,64	68011,44	70568,5	**75981,86**	93257,12	75063,89	113176,9	**93832,65**	65202,5
PAB1 prote	14898,1	20582,47	19442,91	**18307,82**	11968,34	12419,63	14254,24	**12880,74**	11607,24
CYP85A2, E	1337,254	1199,304	1012,301	**1182,953**	3535,994	1228,716	2919,891	**2561,534**	2434,023

Eukaryotic	2290,392	2626,066	2087,484	**2334,647**	3442,712	2911,314	4360,401	**3571,475**	3058,522
ACX4, ATSC	11136,82	12191,87	10104,84	**11144,51**	15624,82	9799,538	15943,65	**13789,33**	17745,24
ATPAP26, F	6511,629	8299,065	8119,594	**7643,429**	7495,661	11371,05	9635,927	**9500,878**	5006,213
Rhodanese	2638,051	1529,824	1843,349	**2003,741**	330,7443	1645,206	178,7685	**718,2396**	515,2808
Signal reco	1869,664	1840,502	2700,251	**2136,806**	2910,611	2121,217	2406,133	**2479,321**	1566,087
TCP-1/cpn6	2210,498	1597,713	2641,245	**2149,818**	1481,452	1546,57	1192,096	**1406,706**	1898,363
Protein kin	873,8648	601,2157	598,2062	**691,0956**	933,4157	2519,371	1007,025	**1486,604**	602,7189
Lactate/ma	2142,745	2203,115	2332,128	**2225,996**	3020,169	3092,485	3090,536	**3067,73**	2472,097
NAD(P)-bin	1249,947	3711,235	3065,323	**2675,502**	3390,13	1478,837	1599,321	**2156,096**	4774,04
Glutathion	11200,71	5882,78	4342,622	**7142,038**	11115,64	14088,58	20293,35	**15165,86**	8675,015
AIR3 Subtil	352,4082	213,5046	288,3112	**284,7414**	219,2011	304,2052	129,5927	**217,6663**	108,2896
D-3-phosph	6705,718	8371,006	8190,298	**7755,674**	4132,409	7507,215	4950,711	**5530,112**	4123,101
UNE18, OR	482,0861	1927,047	2070,157	**1493,097**	2083,437	137,3908	310,3657	**843,731**	4613,182
ACL, ATME	82,65868	51,44337	420,4376	**184,8465**	631,1445	281,6753	541,1216	**484,6472**	565,7933
GDH3 gluta	342,3744	366,7982	217,1826	**308,7851**	331,3396	481,3546	926,9554	**579,8832**	246,7964
TCTP transl	136446,6	110208,6	126246,7	**124300,6**	138144,5	126545,2	155911,1	**140200,3**	117331,2
ATIMD3, IM	10604,85	12097,52	13829,73	**12177,37**	11378,76	11561,79	10672,01	**11204,19**	9583,293
PCK1, PEPC	17911,92	21185,89	17712	**18936,6**	22328,39	22555,34	20699,61	**21861,11**	18361,12
CRU3, CRC	200,7149	142,5916	365,3028	**236,2031**	303,0448	3421,698	3968,592	**2564,445**	152,8166
BETAG4 be	5072,509	6205,819	6303,101	**5860,476**	4751,507	8652,174	7920,442	**7108,041**	3714,375
diaminopin	4187,699	3940,631	3481,432	**3869,92**	6269,045	3318,79	6835,617	**5474,484**	6771,712
ATMLP-470	107604,9	150698	122704,8	**127002,6**	127765,4	138994	165384,8	**144048,1**	103173
BTI2, RTNLI	7683,657	6835,482	5404,663	**6641,267**	14070,19	8242,837	18209,37	**13507,47**	9770,651
ACHT2 atyp	1302,105	1867,774	2076,562	**1748,814**	1459,738	1396,559	2678,226	**1844,841**	892,007
Pyruvate ki	22043,37	28264,37	27908,86	**26072,2**	41680,78	29919,41	36438,31	**36012,83**	34839,5
PTB3 polyp	850,727	1461,454	1198,016	**1170,066**	1854,953	1115,604	2071,257	**1680,604**	1958,396
FBA1 fructo	177,5423	280,0807	174,1579	**210,5937**	395,633	173,6019	380,4751	**316,57**	399,0379
Protein kin	1153,82	1590,16	1591,12	**1445,034**	1898,628	1591,341	1974,907	**1821,626**	2179,868
ATGRP7, CC	5352,962	7072,667	7741,148	**6722,259**	14959,05	8303,878	21041,39	**14768,11**	17425,52
S-adenosyl-	135,9078	191,1518	183,1488	**170,0694**	295,4247	214,428	193,7554	**234,536**	220,4951
transducin	1991,867	2239,277	1691,98	**1974,374**	3256,398	1690,509	3152,37	**2699,759**	1601,925
CONTAINS	115,1882	72,03366	41,64135	**76,28775**	106,6069	51,23936	326,9052	**161,5838**	272,6256
Class I gluta	1846,814	1726,895	1350,522	**1641,411**	2095,837	1457,418	925,6469	**1492,967**	874,4788
DEAD/DEA	3279,895	2903,468	3171,027	**3118,13**	4658,767	2663,95	5544,628	**4289,115**	1876,586
VQ motif-c	21351,08	20236,38	21337,25	**20974,9**	12898,65	20152,7	10743	**14598,12**	12960,2
Pentatricop	313,1932	477,0078	446,0278	**412,0763**	623,0029	277,9442	624,0415	**508,3295**	912,987
PBC1 prote	12128,07	16165,09	12339,83	**13544,33**	18077,29	13272,99	16179,38	**15843,22**	18226,94
PBB1 N-ter	2713,62	1686,339	1630,75	**2010,236**	2264,887	2206,536	2436,431	**2302,618**	3077,443
FIB2, ATFIB	5315,293	4513,781	3028,171	**4285,748**	5630,25	6970,367	7053,34	**6551,319**	5322,893
FLDH NAD(	14396,06	12282,38	13045,86	**13241,43**	14572,66	15758,92	22242,81	**17524,8**	12738,49
NRPA2 nuc	554,348	591,4777	812,3088	**652,7115**	362,4856	433,0864	324,8228	**373,4649**	243,7758
TUB5 tubul	20916,19	16501,39	18102,51	**18506,7**	18280,35	22003,03	25409,09	**21897,49**	16491,9
GDSL-like L	20589,46	20104,15	18114,8	**19602,8**	20901,24	29095,45	22157,74	**24051,48**	17895,99
AtGLDP1, G	16413,7	9452,384	10539,22	**12135,1**	9194,83	12101,42	10837,19	**10711,15**	7172,662
ACLA-1 ATF	11126,29	11518,25	11738,64	**11461,06**	13410,16	8663,796	7499,315	**9857,758**	7329,643
Histidine ki	607,0833	333,1207	420,6376	**453,6139**	593,7272	306,6664	826,1697	**575,5211**	183,0906
Dihydrolipo	34628,05	27958,86	28455,72	**30347,54**	32211,23	41750,75	40968,17	**38310,05**	23956,4
Plant inver	15464,78	13705,32	14629	**14599,7**	14633,84	12562,27	15534,9	**14243,67**	13100,71
ATGSR2, GS	31046,23	43050,69	37058,36	**37051,76**	48460,93	29136,92	41681,43	**39759,76**	30542,37
mismatche	1439,476	1888,163	1917,114	**1748,251**	2036,014	2060,767	1696,558	**1931,113**	2089,016

NF-YA8 nuc	1438,082	138,105	69,16664	**548,4512**	51,48793	5,127613	12,82262	**23,14605**	62,76858
ATPMEPCR	112,2043	1429,167	1439,789	**993,72**	377,985	118,6003	70,58942	**189,0582**	1251,736
PBF1 N-ter	22753,74	20850,12	21780,96	**21794,94**	12156,48	16643,65	14489,59	**14429,91**	10979,64
myosin hea	97,20116	57,98883	166,7167	**107,3022**	295,1339	31,39833	234,1189	**186,8837**	507,3842
MAP2A me	2088,87	1475,8	1753,697	**1772,789**	1154,519	2331,222	6639,727	**3375,156**	289,0528
NAD(P)-link	17,31591	108,527	39,19209	**55,01168**	210,5347	46,86078	374,146	**210,5138**	16,05975
FAD-bindin	1074,939	1944,046	1014,16	**1344,382**	1921,894	2767,905	2092,665	**2260,821**	1676,055
TUA5 tubul	27916,75	25654,62	26752,57	**26774,64**	52252,78	22469,87	47199,67	**40640,78**	56536,76
Ribosomal	14622,6	12824,7	14118,02	**13855,11**	9919,955	16468,43	9305,758	**11898,05**	8998,829
CPHSC70-2	1823,889	1439,318	2276,528	**1846,578**	1305,519	1694,905	1180,158	**1393,527**	1360,051
FLA7 FASCI	73598,37	89437,24	89303,62	**84113,08**	109201,5	89927,46	97798,91	**98975,95**	80094,05
Alkaline-ph	21526,36	12632,56	13871,47	**16010,13**	12377,92	15276,92	15323,84	**14326,23**	9743,261
TRIP-1, TIF3	23209,44	34338,15	26878,16	**28141,92**	30187,82	34710,51	22114,54	**29004,29**	19165,34
BGLU16 be	1776,482	1241,74	1440,372	**1486,198**	1629,189	1203,132	1587,42	**1473,247**	879,9444
ARA12 Sub	2128,934	3278,729	2407,257	**2604,973**	3247,301	3575,666	3919,39	**3580,786**	3204,544
cpHsc70-1	5799,142	9276,531	8172,621	**7749,431**	10772,67	8998,637	11534,15	**10435,15**	11941,74
RCI3, RCI3A	95731,74	106180,2	101607,1	**101173**	93890,09	132035,7	114470	**113465,3**	92074,04
UBP13 ubiq	362,1688	78,39001	33,8319	**158,1302**	80,10435	76,07573	376,4038	**177,528**	666,0027
unknown p	3850,434	2809,914	2898,866	**3186,405**	3466,567	3384,99	2724,16	**3191,906**	4197,426
ATPMEPCR	105055,3	82713,3	93061,02	**93609,89**	116710,6	110082	135137,2	**120643,3**	160746,2
ACX6 acyl-C	1638,934	1332,611	1430,403	**1467,316**	426,5502	1276,995	203,0855	**635,5435**	302,6787
AtMC4, MC	9103,582	7100,809	7358,976	**7854,456**	6769,534	8816,285	10416,25	**8667,355**	6134,314
Ribosomal	8975,704	9676,309	9478,491	**9376,835**	8392,603	20221,78	17750,7	**15455,03**	5652,439
GDP dissoc	2518,9	1585,242	2245,821	**2116,654**	812,7634	2874,31	1639,978	**1775,684**	474,6822
RACK1C_AT	9455,833	6927,101	6013,864	**7465,599**	11359,66	8849,74	11826,53	**10678,64**	10331,5
ATFP8, ATF	1326,267	1293,394	1262,917	**1294,193**	1016,169	1426,251	871,6265	**1104,682**	1468,845
GLN1;5 glu	7844,471	9879,249	9754,172	**9159,297**	8002,586	6673,662	5090,124	**6588,791**	6280,317
PBP1 PYK10	403363,1	361198,7	374609,6	**379723,8**	363623,9	367759,8	470335,3	**400573**	309740,4
Adaptin far	15113,18	18254,71	17901,68	**17089,86**	10043,93	20148,02	10512,13	**13568,03**	10252,54
PGK1 phos	18700,91	21017,87	19042,98	**19587,26**	22389,36	17063,24	22155,45	**20536,01**	26071,48
TRAF-like fa	110887,5	94298,84	92136,43	**99107,59**	126828,6	115691,4	175574,4	**139364,8**	116457,1
SBP3 selen	378,4804	506,9769	572,052	**485,8364**	602,1877	170,0768	339,5577	**370,6074**	745,0244
CA2, CA18,	7602,398	4171,729	5253,821	**5675,983**	3020,344	5438,743	3556,173	**4005,087**	3280,461
Heat shock	99639,21	95898,56	96267,66	**97268,48**	105470,8	76688,44	91904,51	**91354,6**	77838,35
MD-2-relat	4907,363	4625,825	5456,634	**4996,607**	3990,29	4031,043	4282,44	**4101,258**	4065,218
F-box assoc	11416,82	18959,12	22403,77	**17593,24**	23009,13	25907,54	47367,06	**32094,58**	9348,558
PLP5 PATAT	64,51922	225,9568	271,9221	**187,466**	86,98941	33,19875	33,78217	**51,32344**	107,0233
Acyl transfe	1319,149	1239,206	1094,292	**1217,549**	2023,381	1331,787	2313,106	**1889,425**	1744,263
SEC8, ATSE	27179,04	20223,66	17455,92	**21619,54**	11336,48	31295,38	3857,353	**15496,4**	7118,62
IDH1, IDH-I	9645,033	13549,64	13066,84	**12087,17**	14386,13	10268,71	12470,48	**12375,11**	15406,97
SPFH/Band	26095,57	27897,23	27408,23	**27133,68**	38323,95	25912,89	39235,9	**34490,91**	25522,04
haloacid de	38120,34	57229,08	53743,41	**49697,61**	33832,72	65578,83	50270,02	**49893,86**	26964,09
NRS/ER, UE	65965,65	77240,87	80188,98	**74465,17**	97044,71	78671,37	99223,76	**91646,61**	86914,58
Pentatricop	2939,921	2189,939	2249,207	**2459,689**	1995,132	1863,25	1409,795	**1756,059**	2093,98
ADNT1 ade	303,0272	630,0934	1172,613	**701,9112**	1187,363	446,094	1042,443	**891,9667**	306,9092
CYP98A3 cy	7816,664	7070,977	5731,621	**6873,088**	9123,837	7124,684	11647,24	**9298,586**	10064,25
AT-IMP, AT	2230,116	3026,161	2885,216	**2713,831**	3545,897	1890,888	3630,519	**3022,435**	4633,715
ADL2, DRP3	7103,871	5825,542	6184,181	**6371,198**	5653,392	8382,022	5195,051	**6410,155**	4363,455
SLAH2 SLAC	55,51507	1879,435	5602,892	**2512,614**	21379,81	16713,43	8922,035	**15671,76**	12125,86
Phosphorib	4783,924	2978,65	3624,292	**3795,622**	2602,928	879,6	2692,418	**2058,315**	4293,15

VDAC4, AT\	202,3851	420,8074	202,4953	**275,2293**	663,7838	113,1078	756,2751	**511,0556**	993,4786
P-loop cont	1898,815	3680,327	4456,309	**3345,15**	3360,305	3233,066	3577,052	**3390,141**	5353,168
Leucine-ric	578,4799	229,8283	293,78	**367,3628**	499,1866	315,612	446,9843	**420,5943**	221,839
Peroxidase	45648,68	51938,57	54158,81	**50582,02**	57328,48	126893,2	92544,65	**92255,45**	40966,36
OASC, ATC	22951,54	30948,41	27696,89	**27198,95**	22491,75	21836,96	16194,37	**20174,36**	23026,45
ATDFB, DFE	344,2726	465,7135	349,8544	**386,6135**	955,8663	231,5303	1017,642	**735,0128**	1138,862
Pyruvate ki	929,1523	760,9955	524,9764	**738,3748**	668,4141	498,8201	421,9984	**529,7442**	363,381
AGT2 alanii	24368,66	28786,96	25449,75	**26201,79**	35055,1	28476,26	37524,57	**33685,31**	33208,08
NHO1, GLI1	46604,62	50977,24	47697,49	**48426,45**	51703,2	36068,39	40668,49	**42813,36**	39051,51
RSW3, PSL5	6736,391	8323,696	7192,697	**7417,594**	8613,682	8607,552	8263,194	**8494,809**	8412,462
Ribosomal	4506,181	2903,639	3558,032	**3655,95**	1476,206	3538,353	2701,1	**2571,886**	1344,758
cellular apo	672,8154	1256,408	1562,643	**1163,955**	2115,878	1109,119	1322,747	**1515,915**	2106,64
Tudor1, AtT	20548,68	24582,85	23827,47	**22986,33**	27877,32	27748,81	41350,03	**32325,39**	25693,48
MLP31 MLI	3202,129	3864,154	5055,543	**4040,609**	4737,529	4339,494	5624,673	**4900,565**	5292,62
PDF1, PR 6.	1788,035	1519,888	1545,921	**1617,948**	2236,298	1803,733	3055,917	**2365,316**	1962,508
coatomer g	163152,1	177618,1	178768,2	**173179,5**	182970,1	183547,1	170662,5	**179059,9**	168172,6
Protein of u	28526,8	34408,61	30794,74	**31243,38**	41952	32459,93	44174,98	**39528,97**	38678,77
ATHSRP54A	148,6449	135,8596	115,3518	**133,2854**	92,35266	114,041	46,4816	**84,29175**	37,86642
Transmeml	77,87609	380,1751	74,28583	**177,4457**	509,5143	132,7382	602,9353	**415,0626**	776,6093
Class I gluta	144620,5	166613,6	175943,3	**162392,5**	162390,2	109636,9	136643,7	**136223,6**	122569,5
MAG1 Calc	1178,778	398,5931	263,6399	**613,6702**	177,4525	368,3291	338,7304	**294,8373**	141,8865
GroES-like :	9139,38	10338,42	8985,041	**9487,613**	18052,3	9759,789	22925,79	**16912,63**	8371,367
TCP-1/cpn6	15275,55	17773,01	15645,09	**16231,22**	19849,96	17238,43	22809,69	**19966,03**	17587,12
ATPDIL2-3,	13475,1	13069,22	13183,47	**13242,6**	13869,03	14970,7	16346,4	**15062,04**	12926,4
BGLU18 be	3186,052	2208,168	2596,455	**2663,558**	3152,821	3215,561	4016,319	**3461,567**	4026,691
IAR3, JR3 p	1251,915	828,3504	832,9144	**971,0601**	536,1092	1402,854	1235,374	**1058,112**	144,5067
Metallopep	22,068	274,2639	343,5666	**213,2995**	725,7316	499,7423	808,4575	**677,9771**	775,7178
RAB7, ATRA	10656,88	3864,423	4962,149	**6494,483**	1811,836	6617,672	1587,333	**3338,947**	1624,851
HXXXD-typ	1101,645	715,3356	889,1655	**902,0486**	724,7469	594,3926	882,072	**733,7372**	607,8409
ATECA4, EC	3555,598	3716,438	3022,324	**3431,454**	4930,617	4001,313	6281,744	**5071,225**	3625,978
dehydratas	9867,237	10290,76	11978,03	**10712,01**	13370,62	7480,787	11118,72	**10656,71**	7562,627
ATCYSC1, A	4697,894	4076,833	4608,171	**4460,966**	2829,164	3550,748	5745,716	**4041,876**	5638,139
F-box/RNI-l	1280,54	2141,255	2423,801	**1948,532**	2447,127	3842,496	2005,709	**2765,111**	3513,811
CSD1 coppe	1207,7	4407,677	2510,927	**2708,768**	2571,901	2560,22	2236,923	**2456,348**	4516,577
pfkB-like ca	92033,04	98267,25	85536,67	**91945,66**	107913,6	68015,95	107984,3	**94637,96**	123781,7
ROC5, ATCY	189225,4	257110,1	205428	**217254,5**	236875,1	247785,6	216517,5	**233726,1**	265158,8
xylose isom	6670,721	6386,444	6425,604	**6494,256**	4497,157	5511,716	3426,49	**4478,454**	3871,014
EMB3003 2	21317,59	17161,59	19876,8	**19451,99**	17938,9	14531,58	17198,18	**16556,22**	17041,25
alpha/beta	677,5289	480,1077	596,8186	**584,8184**	735,8471	350,282	840,2413	**642,1235**	1025,03
SMC5 struc	6473,59	4620,954	4757,695	**5284,08**	4159,003	4599,103	3281,715	**4013,274**	3816,142
ATHDH, HIS	7158,492	7854,079	8808,77	**7940,447**	9383,428	10587,28	8721,747	**9564,152**	9077,969
Galactose r	26628,51	42048,57	35080,1	**34585,73**	39882,89	49320,1	48993,81	**46065,6**	33589,71
BED zinc fir	5045,235	4315,41	4511,844	**4624,163**	5484,952	3561,012	6606,614	**5217,526**	3497,415
PKp3 plasti	299,7876	1521,473	1676,211	**1165,824**	1051,173	713,6839	854,2571	**873,038**	411,7082
DEA(D/H)-b	36220,79	33861,48	41052,7	**37044,99**	33638,93	27777,36	33377,18	**31597,82**	39918,06
EID1 F-box	1246,562	344,3141	328,6508	**639,8423**	136,5263	417,0158	68,65616	**207,3994**	120,2024
PDH-E1 BET	15554,85	10631,61	11488,66	**12558,37**	11715,14	12867,16	11276,78	**11953,03**	9316,524
HA9 H(+)-A	584,7086	653,4676	585,0555	**607,7439**	662,6474	412,1362	415,176	**496,6532**	668,0033
ALDH10A8	4957,83	3240,654	4041,672	**4080,052**	3474,22	2364,368	2728,221	**2855,603**	3353,638
dihydrolipo	11200,42	13353,82	14845,76	**13133,34**	8549,877	12770,26	6807,264	**9375,801**	8476,402

Transducin	118,0323	2336,873	2922,911	**1792,605**	2369,169	1626,839	1482,631	**1826,213**	142,3241
PIP3B, PIP2	9101,773	8202,27	8410,066	**8571,37**	5238,343	8224,453	5096,951	**6186,582**	7278,045
SCPL49, CP	1081,347	1252,605	1235,655	**1189,869**	1368,144	1623,586	2011,809	**1667,846**	722,2952
ADP-glucos	692,5987	1465,501	1437,125	**1198,408**	964,9282	588,0516	468,4444	**673,8081**	720,4161
NADH-ubiq	2176,237	2626,75	2809,771	**2537,586**	3790,827	4040,401	3825,757	**3885,662**	2675,017
ATPA ATP s	14103,91	18293,41	19669	**17355,44**	14363,01	17939,34	16971,27	**16424,54**	12506,71
BCE2, LTA1	617,2117	1326,659	1217,066	**1053,645**	1950,467	750,7097	1631,912	**1444,363**	2306,051
CONTAINS	486,301	46,86171	387,2119	**306,7916**	35,31162	231,3373	31,25954	**99,30281**	15,7448
AIM1 Enoy	24777,87	26794,67	30076,01	**27216,19**	27044,09	22706,27	26759,82	**25503,4**	22992,02
Pectin lyase	4,657284	51,21264	67,11745	**40,99579**	53,08126	13,05541	10,64492	**25,59386**	118,4974
GRF11, GF1	1825,309	2189,665	2199,749	**2071,574**	3363,714	1712,212	3247,986	**2774,637**	3287,033
ATIMD1, IM	7247,284	5152,704	3806,479	**5402,156**	6251,694	9145,527	9067,634	**8154,952**	7098,716
FBD, F-box	456,2396	652,9549	548,3762	**552,5236**	345,7074	515,8596	316,0829	**392,5499**	555,5581
ERF1-2 euk	2621,366	4312,948	5199,36	**4044,558**	4805,037	4882,769	5150,679	**4946,162**	5872,478
UGE2, ATU	320,3157	449,7111	915,4442	**561,8237**	215,5998	230,0044	382,9531	**276,1858**	625,8056
ATPHB4, PH	5391,203	4394,19	4254,012	**4679,802**	904,2679	4936,999	217,8994	**2019,722**	609,443
VLN2, ATVL	6309,114	4961,725	5084,635	**5451,825**	3710,638	5720,273	5549,901	**4993,604**	4169,158
CINV1 cyto	32593,57	23052,78	25173,87	**26940,07**	22851,51	23780,74	19709,17	**22113,81**	17104,91
Translation	3674,729	6939,787	8144,147	**6252,888**	7586,307	3540,195	3586,165	**4904,223**	9294,329
IDH-V isoci	4886,868	6008,469	5986,391	**5627,243**	5511,397	4096,392	3607,094	**4404,961**	5621,057
RPT3 regula	24011,07	23695,32	19292,38	**22332,92**	16875,45	20066,5	18339,55	**18427,17**	15304,27
MFP2, ATM	30982,16	32482,08	31861,31	**31775,18**	33929,82	28451,95	38046,24	**33476**	28819,68
Zinc-bindin	703,9065	739,0737	740,6384	**727,8729**	1203,305	1861,314	4597,482	**2554,034**	556,3484
RTNLB3 Re	2224,343	3050,272	2705,169	**2659,928**	5280,626	2855,967	5679,752	**4605,449**	4433,238
CR88, EMB	14206,41	12915,65	12459,25	**13193,77**	14291,63	13367,33	12728,37	**13462,44**	12189
ATPDIL1-1,	148587,3	170116,2	168724,3	**162475,9**	185708,9	172317,2	229580,5	**195868,9**	165643,5
Phosphoen	2383,805	2150,022	2256,152	**2263,326**	4390,949	2143,04	4525,549	**3686,513**	4863,872
ATFER3, FE	1163,003	675,5217	839,9144	**892,8132**	613,3712	769,766	892,2785	**758,4719**	1303,518
HAP13 Clat	1161,261	1646,653	1449,025	**1418,98**	1515,426	1992,199	1526,51	**1678,045**	1165,717
Ankyrin rep	4614,976	4023,418	4360,649	**4333,014**	5014,571	3188,326	4072,478	**4091,792**	2637,181
RGP2, ATRO	74557,07	59602,51	71449,05	**68536,21**	64880,2	50530,51	61604,54	**59005,09**	68319,81
MTHFR2 m	32449,09	32056,33	35066,98	**33190,8**	36959,54	28599,76	40263,31	**35274,21**	27609,4
GGH2 gamı	1302,139	1251,193	1513,124	**1355,486**	2462,671	1352,239	2961,899	**2258,936**	2195,776
MLP34 MLI	83100,24	86270,79	84558,75	**84643,26**	66781,46	119173,2	88881,9	**91612,17**	33771,68
PEN3, PDR8	29391,82	42920,57	47566,74	**39959,71**	51214,98	40662,56	72410,3	**54762,61**	67243,53
OTC ornith	1573,35	770,2457	999,5792	**1114,392**	716,1918	771,8409	779,0259	**755,6862**	520,7135
EMB1006 F	5066,662	1763,563	2438,78	**3089,668**	1226,626	5241,405	1455,2	**2641,077**	958,9143
ATPase, AA	2140,742	2905,707	2836,512	**2627,654**	2264,027	1390,83	1882,788	**1845,882**	2880,462
Hsp89.1, A	28387,75	39599,89	34760,76	**34249,47**	36978,41	36904,66	34520,64	**36134,57**	31086,87
F3H, TT6, F	70,75784	153,7614	95,91158	**106,8103**	67,49549	101,0347	186,1444	**118,2249**	16,66045
ATPME3, P	44246,32	56682,09	56192,1	**52373,5**	63694,06	47963,84	84694,1	**65450,66**	77404,27
ATCWINV1	23360,4	18923,69	21922,77	**21402,29**	20765,25	28480,02	33003,33	**27416,2**	20864,03
MEE34 Mo	11304,32	10322,02	23365,1	**14997,15**	9611,381	10129,1	5931,024	**8557,17**	7163,786
LOS1 Ribos	458573,7	452299,6	417583,5	**442819**	636799,4	385434,7	698523,8	**573586**	413688,3
ATP-depen	361,4346	1121,03	887,6503	**790,0384**	454,5344	301,8922	278,5256	**344,9841**	542,4284
2-oxoglutaı	3110,179	1332,193	3741,606	**2727,993**	1676,391	2315,303	1398,299	**1796,664**	1359,207
SHD, HSP90	128491	128558,1	136819,8	**131289,6**	159951,9	131527,9	173767,3	**155082,4**	157996,9
ketol-acid r	203892	235635	219147,2	**219558,1**	269934	171129,3	191548,6	**210870,6**	171833
Protein of ı	40,91787	55,88715	110,8488	**69,21794**	96,11999	44,37932	178,1913	**106,2302**	196,302
Protein of ı	1154,54	858,7988	1025,829	**1013,056**	2376,346	1027,487	2983,814	**2129,216**	2055,689

ATP3 gamn	62865,48	63414,62	67871,91	**64717,33**	79428,66	52563,16	103620,5	**78537,43**	98639,41
PLDALPHA1	199988,9	213471,5	218197,2	**210552,5**	275225,9	203395,5	299390,6	**259337,3**	207529,6
GAPC-2, GA	76635,2	58740,59	58801,9	**64725,89**	114368,9	65587,96	199048,3	**126335,1**	62421,87
unknown p	575,6222	1162,871	1075,911	**938,1347**	604,8279	339,8804	621,591	**522,0997**	445,5559
ATPLC2, PL	2763,677	5304,052	4647,588	**4238,439**	6256,879	5903,658	6624,835	**6261,791**	5802,685
Translation	18736,31	19070,5	19025,16	**18943,99**	18062,35	15048,29	19722,14	**17610,93**	21022,36
Carbohydra	6042,495	8645,12	7078,002	**7255,206**	8426,288	8694,672	10984,34	**9368,432**	8335,04
Ribosomal	2387,084	3591,324	3007,494	**2995,301**	6580,106	11660,79	9748,121	**9329,674**	10203,24
TMN1, AtTI	1041,172	640,0543	805,3019	**828,8428**	997,4619	2359,862	3910,758	**2422,694**	1349,637
ALDH5F1, S	9109,982	10417,64	10701,87	**10076,5**	11432,42	8397,088	9385,232	**9738,247**	11673,9
methionine	2362,248	1063,404	1291,96	**1572,537**	1871,944	1228,518	2647,994	**1916,152**	4289,443
Ribosomal	1126,726	796,1771	975,5592	**966,154**	1580,008	4977,958	3964,875	**3507,614**	320,589
UBQ12 ubi	431,3914	584,0504	754,6804	**590,0407**	742,8852	610,0473	551,9089	**634,9471**	845,9915
Protein kin	11227,03	17958,97	17235,32	**15473,77**	11089,47	13434,07	8464,98	**10996,17**	9853,074
Glycine cle	19567,2	26803,65	22033,7	**22801,52**	16620,37	21111,67	16100,58	**17944,21**	16027,05
FIB4 Plastic	2036,743	3426,707	2612,238	**2691,896**	3803,813	2550,287	3555,084	**3303,061**	3801,951
60S acidic r	83,15763	168,924	245,1082	**165,7299**	262,7238	5,493412	13,43774	**93,885**	403,5903
GroES-like	2218,826	1566,408	1737,994	**1841,076**	1283,81	1677,484	1153,835	**1371,71**	1555,748
INVOLVED	10076,32	9916,37	12070,46	**10687,72**	11682,88	10405,66	11576,31	**11221,61**	14146,81
O-Glycosyl	2265,725	3012,583	2930,582	**2736,297**	3171,466	2322,883	2417,665	**2637,338**	2140,81
BGLU24 be	2641,746	3227,267	3233,07	**3034,028**	2929,254	1827,013	2346,08	**2367,449**	2137,259
Eukaryotic	29682,97	15998,82	15834,04	**20505,28**	13529,08	6178,249	13146,1	**10951,14**	19609,32
GRF5, GF14	47,30991	60,29338	28,49236	**45,36521**	60,44981	437,1748	116,7292	**204,7846**	78,30749
Phosphofru	34140,87	37261,24	40651,92	**37351,34**	61037,86	36035,39	64374,46	**53815,9**	49378,4
Phosphofru	34409,84	36944,74	42223,41	**37859,33**	30861,18	49033,19	26135,82	**35343,39**	28419,91
RACK1B_AT	8160,293	14287,54	16584,4	**13010,75**	15240,67	13665,23	18138,98	**15681,63**	9869,673
RmlC-like c	399,7221	631,7508	892,5805	**641,3511**	906,7366	1600,449	1216,979	**1241,388**	248,5551
ACC1 acety	3536,723	3449,75	3854,105	**3613,526**	2987,745	3372,05	3274,301	**3211,365**	3302,788
ILA ILITYHIA	12802,34	27808,85	23557,69	**21389,63**	30130,05	12672,92	16958,57	**19920,51**	33271,75
VHA-A2 vac	5494,19	1718,518	984,5064	**2732,405**	3663,117	2648,898	5758,417	**4023,477**	6088,2
TUA1 alpha	16774,94	15969,59	17417,24	**16720,59**	12357,57	15594,3	8423,691	**12125,19**	12729,29
sks5 SKU5 s	12226,28	15069,43	15747,19	**14347,63**	15225,36	15135,38	19124,54	**16495,1**	17633,47
RD28, PIP2	19707,62	21609	22514,2	**21276,94**	14547,38	23221,35	14358,91	**17375,88**	15946,8
ERD7 Senes	14765,64	8611,706	13054,16	**12143,83**	14021,47	13326,21	9133,088	**12160,26**	7790,852
Cyclase fan	7105,842	6127,947	5291,722	**6175,171**	6266,786	6457,482	8833,07	**7185,779**	5304,582
MSBP1 me	145,5802	265,5186	185,5527	**198,8838**	1011,775	219,6289	773,1944	**668,1993**	430,7893
CDA1, AT-C	1071,344	1288,222	1624,567	**1328,044**	1391,145	1092,019	1080,306	**1187,823**	1720,294
CDC48, ATC	24118,9	33296,54	29829,18	**29081,54**	38722,67	28383,01	40212,11	**35772,59**	38145,83
ATHM2 Thi	654,8146	529,1591	582,0022	**588,6586**	328,2981	610,7867	338,5829	**425,8893**	607,4196
MAML-4, IF	682,6705	953,5922	1171,168	**935,8102**	1036,44	835,7893	1398,9	**1090,376**	1507,831
CSI1 bindin	192,1472	458,6987	772,3704	**474,4055**	1746,203	389,8972	1238,893	**1124,997**	1268,802
PDR7, ATPD	9362,534	11010,96	12018,53	**10797,34**	10470	12157,1	8846,054	**10491,05**	8834,442
FLA10 FASC	630,8779	734,5251	1026,508	**797,3038**	1944,179	470,4457	1041,176	**1151,934**	1506,545
TPR1 TOPL	13603,56	13124,5	13247,71	**13325,25**	12936,82	11288,23	11802,48	**12009,18**	13043,16
Flavin-bind	5830,394	3582,676	4470,827	**4627,966**	3796,613	5300,715	1631,932	**3576,42**	1887,242
Clathrin, he	20426,17	30598,64	29329,84	**26784,88**	48774,6	27545,79	42819,91	**39713,43**	39112,85
Zinc-bindin	290,1554	280,7512	642,212	**404,3728**	874,7884	422,0067	982,7704	**759,8552**	699,7518
ATPHOS32,	7964,895	5719,387	5751,946	**6478,742**	5864,838	5381,878	5462,323	**5569,679**	5353,725
ENO1 enola	50893,02	64852,35	54428,03	**56724,47**	63098,41	52340,88	63499,67	**59646,32**	69957,03
THY-2 thym	167,8024	107,474	314,0728	**196,4497**	6,304749	283,1518	26,67147	**105,376**	8,992554

PDC2 pyru	5065,395	8857,349	14212,69	**9378,48**	9524,734	6488,457	10545,15	**8852,779**	18493,32
ASP3, YLS4	10516,32	9967,384	12872,25	**11118,65**	12123,75	12736,91	14330,05	**13063,57**	11644,95
NADH-ubiq	17028,53	29666,47	27259,22	**24651,4**	30588,6	26320,36	34303,99	**30404,32**	34711,34
ATC4H, C4I	96355,54	88801,68	88048,22	**91068,48**	103551	91359,43	105366,1	**100092,2**	95989,85
KAS III 3-ke	1889,795	2963,136	3674,388	**2842,44**	3792,84	4413,298	3796,972	**4001,037**	3445,882
DET3, ATVI	52953,22	55785,68	57827,47	**55522,12**	71195,29	50895,86	71048,64	**64379,93**	68612,91
MDAR6 mo	108241	129575,1	120144,3	**119320,1**	146956,9	116254,7	140383,8	**134531,8**	145533,2
Zn-depend	11531,67	14618,51	14135,15	**13428,44**	14020,65	10052,12	14001,47	**12691,41**	17190,93
EDA38, SBI	147,7341	190,6943	133,2653	**157,2312**	173,7295	577,2874	451,1451	**400,7206**	131,1359
Coatomer,	31721,12	42834,31	46724,12	**40426,52**	53024,08	40722,97	50864,67	**48203,91**	36681,92
MVD1, ATM	12769,39	15132,92	16216,78	**14706,36**	13259,21	15340,53	18549,93	**15716,56**	11128
Leucine car	69,62899	399,2175	408,1592	**292,3352**	558,0527	387,7507	1104,623	**683,4754**	218,3915
ATGSKB6, (	20305,39	13693,81	16976,33	**16991,84**	18754,01	14628,63	23497,98	**18960,21**	14114,48
GLX2-3, ETI	14973,31	8529,929	10994,19	**11499,14**	9565,569	7572,214	9446,505	**8861,43**	8274,095
ATCAP1, CA	7484,105	23728,44	26346,93	**19186,49**	7187,794	10252,88	8989,327	**8809,999**	8500,252
ATPURM, P	1585,5	1551,51	1364,943	**1500,651**	3418,112	1948,813	3035,219	**2800,714**	1857,973
Sec23/Sec2	3025,547	2008,203	2350,109	**2461,286**	2179,748	1086,014	2616,318	**1960,693**	3509,784
SRG3 sene	6083,349	5164,143	4307,725	**5185,072**	3306,676	5237,811	2842,433	**3795,64**	3755,234
DHS2 3-de	483,4119	384,3075	496,8987	**454,8727**	363,7699	460,6627	274,0802	**366,1709**	440,789
Mannose-b	20679,72	22601,35	27800,21	**23693,76**	15826,11	27422,61	21646,93	**21631,88**	13937,34
PFK5 phosp	943,5783	1718,29	1194,926	**1285,598**	2025,018	1477,266	2684,63	**2062,305**	1482,955
GTP bindin	56332,92	54031,56	52248,54	**54204,34**	54288,95	56601,52	76237,97	**62376,15**	67543,39
PBA1 N-ter	42681,86	49200,29	50553,35	**47478,5**	62201,24	46849,04	58730,28	**55926,85**	59288,09
D-mannose	64240,8	61734,56	71816,12	**65930,49**	58273,38	66810,11	82953,38	**69345,62**	92992,07
Mannose-b	4664,825	3948,109	5235,17	**4616,035**	3771,895	6412,501	4333,012	**4839,136**	3434,424
TRAF-like f	4204,831	4290,849	4553,329	**4349,669**	4806,166	3901,076	4864,42	**4523,887**	5748,872
RNA-bindin	968,9959	983,3665	1967,722	**1306,695**	193,8559	1234,957	109,2725	**512,6953**	585,3854
Thioestera	13967,93	16565,22	15653,21	**15395,45**	14578,1	13519,82	12718,52	**13605,48**	14579,73
ARA, ARA-1	121,4093	120,9229	168,2018	**136,8447**	100,3237	60,46329	111,7137	**90,83356**	103,2316
Threonyl-tI	14840,81	19183,33	20054,25	**18026,13**	19711,34	12673,83	17532,03	**16639,07**	23058,53
Protein of u	4327,563	3498,694	3842,843	**3889,7**	3702,842	4887,468	3275,072	**3955,127**	4433,088
hemf2 Cop	1946,261	2346,358	2386,671	**2226,43**	1552,865	2183,687	2165,181	**1967,244**	1739,053
ACX1, ATA(	10585,8	7858,822	8509,798	**8984,807**	9298,692	6974,663	8279,27	**8184,209**	6327,031
ATPPC2, PF	1085,715	1155,89	1057,44	**1099,681**	935,8933	1425,69	1191,686	**1184,423**	907,7682
ACT2, DER1	12304,08	18776,72	19296,38	**16792,39**	20214,23	13717,88	22415,42	**18782,51**	23718,05
NRP1 NAP1	489,3789	65,90911	426,3323	**327,2068**	1967,832	419,3287	1355,714	**1247,625**	594,3879
MSH1, CHN	25071,94	33650,73	33939,17	**30887,28**	31414,59	36997,81	42443,66	**36952,02**	17189,9
ORF110A R	526,7552	817,2763	671,0562	**671,6959**	696,4719	421,1194	176,0444	**431,2119**	858,1914
TSB1, TRPB	5217,747	7157,431	6887,933	**6421,037**	7876,173	6275,379	8460,589	**7537,38**	7447,929
XH domain	35,09923	131,2644	57,11504	**74,49287**	296,6584	48,95948	26,0481	**123,8887**	255,8455
AtRABA1c,	3299,573	676,1729	1309,789	**1761,845**	785,4185	508,612	745,2529	**679,7612**	1355,826
Peroxidase	26364,9	29097,78	28756,57	**28073,08**	31570,57	27801,66	32901,51	**30757,91**	32027,91
ATBETAFRU	20830,67	20540,66	24490,55	**21953,96**	20375,75	18913,94	18386,12	**19225,27**	20292,76
RNA 3'-tern	3424,971	3423,404	3930,239	**3592,871**	4340,984	2649,319	4234,652	**3741,652**	4540,106
XK-2, XK2 x	207,5297	50,27132	95,39648	**117,7325**	264,6581	205,2136	295,8467	**255,2395**	139,7485
Protein of u	8799,435	12992,69	11656,95	**11149,69**	13243,81	14012,96	13231,95	**13496,24**	10724,86
Ankyrin rep	3751,684	832,3283	1765,227	**2116,413**	593,1039	1616,075	640,2685	**949,8159**	795,4426
Adenine nu	10744,71	5836,74	7916,131	**8165,862**	4268,491	7372,608	3286,388	**4975,829**	5734,273
UXS2, ATU)	1337,018	727,8771	835,9154	**966,9367**	547,5712	1252,361	252,159	**684,0305**	357,6761
ATMPK4, M	8359,749	5840,668	6867,431	**7022,616**	5024,276	6681,649	4593,573	**5433,166**	5639,617

TUBG1 gam	9,947365	36,4343	55,98404	**34,1219**	80,00959	41,06239	152,6032	**91,22504**	109,7648
ATCBR, CBI	21447,66	21947,21	16688,22	**20027,7**	21406,71	16666,22	25550,76	**21207,9**	16539,78
AK-HSDH I,	1711,677	1555,231	1694,832	**1653,913**	2502,625	1380,443	1995,387	**1959,485**	2020,231
ADR1-L3 AI	296,5643	72,83764	111,0384	**160,1468**	95,33695	1441,906	2865,17	**1467,471**	43,33084
ATARFB1B,	207,0669	85,98618	111,5086	**134,8539**	71,54959	233,4512	55,8849	**120,2952**	80,95746
SKU5 Cupre	13630,27	17879,02	16873,93	**16127,74**	13551,99	17834,09	17395,65	**16260,57**	11070,03
GAPCP-2 gl	1368,171	1020,045	1051,146	**1146,454**	1283,313	2065,729	1890,105	**1746,382**	1459,087
NTMC2TYP	20907,68	17559,23	18436,9	**18967,94**	12852,31	24758,3	12764,02	**16791,54**	12704,7
P-loop cont	13816,97	17243,59	19895,71	**16985,42**	12685,25	18188,99	23292,76	**18055,66**	10617,77
Bifunctiona	300,2184	2862,096	2457,996	**1873,437**	7741,009	3846,271	13543,36	**8376,879**	11376,15
AT-HF, HISI	3815,04	4575,833	4506,558	**4299,144**	4143,156	4372,443	5206,234	**4573,944**	4304,534
ARL1 ARG1	41000,99	36843,36	43071,4	**40305,25**	23830,38	40957,08	12598,25	**25795,23**	27067,51
IDH2, IDH-I	8751,3	9932,667	11806,91	**10163,62**	17255,57	7688,216	13331,13	**12758,31**	15731,86
ATMP2, AT	491,187	602,3326	667,3516	**586,9571**	1138,583	236,7341	1129,963	**835,0933**	1493,281
transducin	44931,52	50144,88	55721,72	**50266,04**	51256,5	44534,99	59098,24	**51629,91**	45345,16
SAR1, ATSA	4255,272	8315,556	8166,098	**6912,309**	11138,55	6799,132	11562,78	**9833,486**	10371,86
NAD(P)-bin	4292,13	3818,209	4874,006	**4328,115**	3255,702	4143,078	2343,942	**3247,574**	3483,526
ATHSGBP, /	1494,195	482,1486	759,1266	**911,8236**	2811,42	253,3525	2012,55	**1692,441**	3338,745
AXS1 UDP-I	5431,317	7322,064	6583,171	**6445,517**	13849,21	4578,645	6160,034	**8195,961**	7079,873
HEMC hydr	1895,376	1674,526	1329,239	**1633,047**	1238,88	1442,87	777,3122	**1153,021**	1459,137
AT-RAB2, A	12008,67	13692,48	15145,94	**13615,69**	14180,61	15628,83	16263,76	**15357,73**	15623,07
Pectinacety	796,0571	1174,577	1447,402	**1139,345**	1352,407	1293,646	1715,958	**1454,004**	1574,334
P40, AP40,	97942,89	94892,38	106358,8	**99731,35**	89563,07	91308,31	100832,8	**93901,39**	75247,27
SUS1, ASUS	2358,43	1130,933	1391,082	**1626,815**	1872,225	2039,951	1755,105	**1889,094**	2222,345
Actin-bindi	4857,065	6700,378	5587,559	**5715,001**	7370,898	5496,178	7842,1	**6903,059**	7340,511
Glycosyl hy	46533,65	64119,57	56752,28	**55801,83**	70319,74	57625,13	84007,76	**70650,88**	58112,72
ACCD acety	7726,024	8814,551	9219,596	**8586,724**	15126,81	8700,221	12694,61	**12173,88**	9554,873
Translocon	10898,97	17454,39	12244,16	**13532,5**	21337,67	15147,46	29425,24	**21970,12**	24809,12
AAA-type A	4232,644	3121,155	3843,459	**3732,419**	4605,462	3737,762	5090,565	**4477,93**	2980,862
6-phospho	27383,9	26509,98	26502,06	**26798,64**	21531,12	23937,01	23416,57	**22961,57**	22835,7
aTNUDT9, I	960,3198	1021,032	1144,524	**1041,959**	657,2263	1472,121	440,0567	**856,4679**	597,032
Peroxidase	118877,9	98683,86	103061,1	**106874,3**	104749,5	123848,5	136232,2	**121610,1**	130633,1
Leucine-ric	22461,4	39570,85	38588,38	**33540,21**	36156,06	25336,77	27562,17	**29685**	39854,77
RHM1, ROL	5047,526	7170,092	5927,883	**6048,5**	5339,507	7917,697	4836,643	**6031,283**	4263,426
ASP1 aspar	29224,7	28641,65	24659,44	**27508,6**	19397,7	28300,61	22077,34	**23258,55**	18725,61
RAB1C, ATI	7702,447	7158,697	7848,292	**7569,812**	10419,88	5276,951	10069,53	**8588,788**	10820,51
SecY protei	10841,18	12287,49	13581,99	**12236,89**	10879,67	11965,81	10883,84	**11243,11**	11303,02
PIP2B, PIP2	39074,34	67839,2	60930,66	**55948,07**	76180,42	51830,02	87720,48	**71910,3**	92154,31
ATUBA2, U	1719,651	1308,476	1711,904	**1580,01**	1425,853	1871,343	1338,019	**1545,072**	2069,497
PMEAMT, /	20702,67	14668,4	14493,78	**16621,61**	17153,08	18701,4	19264,26	**18372,91**	14677,2
RBCS1A rib	705,7168	671,5406	715,9641	**697,7405**	1080,148	691,1184	719,9915	**830,4192**	825,8492
AtPP2-A13,	6448,722	6899,635	7941,974	**7096,777**	6438,14	9441,92	5881,372	**7253,811**	9019,566
EIF3E, TIF3	289,6497	210,4395	185,3469	**228,4787**	122,2934	306,7363	250,8723	**226,634**	42,45411
SAM-2, MA	33252,21	40162,35	28296,27	**33903,61**	59589,12	32056,51	84881,63	**58842,42**	29408,52
ADA2A hor	3092,592	3589,091	3854,522	**3512,068**	3169,929	3309,85	3699,658	**3393,145**	2917,368
FLA13 FAS(	81,07322	344,4523	232,2348	**219,2534**	189,5402	444,9753	330,9839	**321,8331**	338,8442
TOM40 tra	5230,504	6043,526	6535,994	**5936,675**	4834,376	3938,191	5578,378	**4783,648**	4241,066
UGP2 UDP-	62,84292	225,2903	44,15005	**110,7611**	290,266	305,3468	134,8602	**243,491**	130,6181
PIP2F, PIP2	5117,032	10049,43	9064,527	**8076,997**	12787,36	7962,691	14654,03	**11801,36**	13724,85
UKL2 uridir	6608,9	8447,909	7303,652	**7453,487**	7028,935	8119,53	5658,636	**6935,7**	5904,334

alpha/beta	654,2373	1019,457	1055,911	**909,8683**	731,4067	1281,046	896,3913	**969,6148**	1337,315
Clathrin, he	33984,76	37697,66	41099,4	**37593,94**	58032,47	37983,18	54605,56	**50207,07**	44302,63
NPSN11, A	6472,053	10066,55	11693,55	**9410,719**	14428,83	10735,01	17299,35	**14154,4**	15883,56
Cysteine pr	10106,41	5107,354	6274,088	**7162,618**	8140,554	14634,06	7856,9	**10210,5**	10865,23
NSP2, ATN	6186,432	7397,146	8387,642	**7323,74**	7303,133	3705,508	3330,385	**4779,675**	5290,771
UBQ8 ubiq	28933,15	32664,27	30512,59	**30703,34**	33328,74	29277,95	33193,47	**31933,39**	36102,9
STV1, RPL2	100,905	192,2853	256,6772	**183,2892**	169,6237	326,7953	343,8062	**280,0751**	276,8971
Insulinase (	49707,12	47073,18	46593,05	**47791,11**	75081,53	44769,59	91827,73	**70559,62**	52455,42
PKP1, PKP-	18502,95	23573,24	20259,43	**20778,54**	25056,9	21351,46	23897,41	**23435,26**	23268,23
CCoAOMT1	67605,58	54240,78	51797,23	**57881,2**	41873,7	48161,8	39389,26	**43141,58**	28033,7
Cytosol am	6439,915	2922,659	4095,942	**4486,172**	5102,653	4327,546	6321,19	**5250,463**	6775,612
ACHT5 atyp	3316,652	4614,525	2392,804	**3441,327**	6161,027	2151,579	8779,713	**5697,44**	7944,606
Protein of u	1666,183	505,5926	1081,374	**1084,383**	412,4838	6926,227	9341,932	**5560,214**	531,3789
Pyridoxal-d	1978,403	3547,308	2520,44	**2682,05**	2643,87	5652,615	5484,989	**4593,824**	3416,671
OASA2 O-a	2954,667	2911,033	2630,926	**2832,209**	2362,051	3050,284	3589,302	**3000,546**	2159,423
ACO1 acon	87269,04	98667,02	94560,08	**93498,71**	110011,3	96154,02	116128,3	**107431,2**	108630,7
phenylalan	1482,827	1150,172	1715,13	**1449,377**	1572,374	1692,891	1885,671	**1716,979**	1797,242
FUNCTION	1601,857	2220,812	2401,633	**2074,767**	3216,843	2141,944	3579,521	**2979,436**	3351,553
Domain of	6764,394	5657	6409,779	**6277,057**	5203,192	6747,491	4404,067	**5451,583**	5015,541
unknown p	250,0634	208,7288	29,33835	**162,7102**	578,3615	69,11031	620,5553	**422,6757**	454,0817
SOUL-1 SO	3812,585	2348,526	2794,078	**2985,063**	1289,282	3402,682	3185,909	**2625,957**	1560,934
Aldolase su	6519,384	2233,707	911,8752	**3221,655**	2836,314	6827,765	9143,099	**6269,06**	1692,374
PGL4 6-pho	963,6724	1414,796	1384,455	**1254,308**	1432,191	771,5468	1403,255	**1202,331**	1882,076
alpha/beta	468,9932	10,61779	43,26938	**174,2935**	219,0352	97,97223	356,1011	**224,3695**	487,1057
L-Aspartase	696,588	436,7823	540,7403	**558,0369**	505,0136	183,758	350,5463	**346,4393**	412,1863
GME GDP-I	5017,584	6739,972	5897,69	**5885,082**	5169,357	6009,655	5610,144	**5596,385**	4258,889
CRT1b, AtC	21571,82	23500,6	18324,47	**21132,3**	15782,3	21429,53	26927,46	**21379,76**	13561,87
Cytidine/de	9579,076	17145,55	16071,35	**14265,32**	25821,75	19135,67	45782,66	**30246,69**	13637,59
AtRLP13, R	590,4268	287,4143	257,2757	**378,3723**	179,5377	485,5283	57,13077	**240,7323**	510,4032
jacalin-rela	1674,885	1680,028	1941,192	**1765,368**	2504,144	1754,441	3454,256	**2570,947**	2001,382
Aldolase-ty	2412,972	3657,43	2807,009	**2959,137**	3003,243	4438,345	5024,196	**4155,262**	3840,171
Mannose-b	499456,5	600253,3	620941,2	**573550,3**	587527,1	555983,8	741124,3	**628211,7**	513076,7
Mitochond	6626,289	8424,439	7237,622	**7429,45**	6299,283	6847,651	5008,574	**6051,836**	6282,188
L-Aspartase	4747,838	6404,073	5326,33	**5492,747**	5697,939	3481,481	4175,401	**4451,607**	5854,157
ATSAR1, AT	735,8944	171,3954	115,753	**341,0143**	127,0358	847,1068	692,8777	**555,6734**	133,6501
ACHT4 atyp	10689,08	13049,69	11838,24	**11859**	5672,264	15595,24	4324,33	**8530,61**	5708,651
AAO1, AO1	5352,173	5848,747	6553,864	**5918,261**	7618,365	5797,311	6215,546	**6543,741**	7339,827
Lactate/ma	4792,378	4353,042	4826,218	**4657,213**	2876,847	6097,741	2770,549	**3915,046**	2310,168
AtNUDT7, (	1887,016	2252,704	1979,994	**2039,905**	3512,97	1948,52	3122,937	**2861,476**	2793,119
GSH2, GSH	4292,836	5925,09	6516,067	**5577,998**	5018,819	3948,031	4564,015	**4510,288**	4163,752
DUF8, ATD	1962,025	797,0924	245,1229	**1001,413**	799,2421	2159,494	2578,827	**1845,854**	676,1578
unknown p	23,05247	28,87168	38,09445	**30,0062**	531,7512	205,0463	293,7652	**343,5209**	659,079
ATMIN7 HC	7942,72	8911,447	9590,739	**8814,969**	12138,14	7810,777	10651,41	**10200,11**	7172,039
ACLA-3 AT	4817,14	7773,003	6360,536	**6316,893**	6795,578	4597,179	6064,577	**5819,112**	7666,776
GHMP kina	7174,704	3462,145	4330,423	**4989,09**	1179,901	2832,984	2638,986	**2217,29**	2486,057
GLP3, GLP3	24799,01	15538,74	12588,7	**17642,15**	35867,84	18937,23	33766,26	**29523,78**	21502,15
ATERDJ2A	993,417	1180,464	1460,84	**1211,574**	1475,677	3069,808	1454,036	**1999,841**	1256,771
ATPMM, PI	17148,23	14278,93	16927,32	**16118,16**	16319,27	14109,72	12983,31	**14470,77**	12928,71
Single hybr	10385,37	10001,39	8961,535	**9782,767**	10358,45	13013,09	12604,76	**11992,1**	10172,84
RPT5B 26S	9343,438	7972,521	8340,157	**8552,039**	6502,244	8062,489	4588,521	**6384,418**	6307,404

tRNA synth	977,7537	2009,904	1905,096	**1630,918**	2922,282	1694,073	2136,606	**2250,987**	2718,862
Tudor2, At	35166,59	35371,21	34455,91	**34997,91**	37137,77	38477,2	43687,67	**39767,55**	37899,68
HXXXD-typ	23559,2	28410,84	29180,64	**27050,23**	30300,34	23650,81	33088,03	**29013,06**	31279,22
UCC2 uclac	117306,5	177990	206137,9	**167144,8**	162820,4	142208	176793	**160607,1**	235148,3
NmrA-like	65,63467	28,26504	50,74158	**48,21376**	122,6179	13,3566	63,57874	**66,51774**	9,763048
ATTRX3, AT	68072,28	69155,95	60872,89	**66033,7**	44841,9	73488,16	41703,23	**53344,43**	68525,76
F-box and a	3621,292	2862,916	2895,318	**3126,509**	2233,877	3364,079	1926,352	**2508,103**	2509,519
UDP-Glyco	22216,93	27438,41	19808,26	**23154,53**	9714,805	54392,81	7489,162	**23865,59**	6431,146
BIP, BIP2 H	13730,91	11357,38	12913,65	**12667,31**	20642,34	11956,38	28058,27	**20219**	16361,16
Thiolase fa	4703,259	5197,328	5312,227	**5070,938**	4811,327	5225,622	3987,444	**4674,798**	4501,917
PMH2, ATR	553,7349	269,4814	361,7442	**394,9868**	398,1033	309,5776	175,8267	**294,5025**	222,99
UDP-Glyco	1152,578	1580,931	1872,849	**1535,452**	1590,869	846,9629	1385,833	**1274,555**	1630,87
Cytidine/de	3310,125	3214,53	2748,815	**3091,157**	8038,93	1934,044	9517,901	**6496,958**	6773,332
SYNC1, EM	1529,349	1244,077	738,545	**1170,657**	1056,761	2457,856	1174,857	**1563,158**	792,1187
PBG1 20S p	14341,32	10811,3	9307,335	**11486,65**	7240,599	14522,31	6505,406	**9422,771**	5168,459
Eukaryotic	55839,58	64224,2	59904,2	**59989,33**	57312,8	72763,29	80733,03	**70269,71**	58919,46
RSW10 Rib	1797,229	4926,976	5634,152	**4119,452**	4596,038	4350,315	1619,134	**3521,829**	2445,287
Plant inver	6652,332	3812,032	4248,788	**4904,384**	5161,772	5671,265	5984,265	**5605,767**	4116,427
GS2, GLN2,	10602,99	13492,73	10950,72	**11682,15**	14925,08	11314,9	19830,83	**15356,93**	15238,06
EXPRESSED	551,9069	534,9712	542,8109	**543,2297**	758,5606	209,8146	321,8868	**430,0873**	704,8912
RPT2a regu	6071,454	6930,105	6886,358	**6629,306**	8198,142	5597,215	9301,476	**7698,944**	8220,566
HSC70-1, H	108350,9	117993,3	112090,3	**112811,5**	160693,1	97898,65	165283,2	**141291,6**	107455,3
ATPOP1, A	1605,701	2535,697	2557,899	**2233,099**	3744,055	2262,05	3953,327	**3319,811**	2983,919
Putative ad	3709,575	2815,867	2329,059	**2951,501**	2156,629	2694,632	1692,831	**2181,364**	2346,239
UDP-glucos	10309,94	7281,171	8814,701	**8801,939**	12202,52	6594,136	7311	**8702,553**	7123,193
TUB3 tubul	43544,78	59034,69	62017,23	**54865,57**	67983,33	58398,43	87648,3	**71343,35**	59717,95
ATTRX5, AT	12423,25	21794,46	15453,95	**16557,22**	20116,33	18915,25	19824,58	**19618,72**	23812,5
PSAT phosp	29305,33	45725,68	45675,04	**40235,35**	46354,49	28737,54	28238,09	**34443,38**	50811,97
EXL6 EXOR	38,76346	166,495	235,1256	**146,7947**	514,411	153,0816	427,149	**364,8805**	261,3477
MIRO1 MIF	1998,107	2130,453	2610,083	**2246,214**	2519,119	1617,965	3767,995	**2635,027**	3734,398
Aldolase-ty	175918,6	182129,1	156123,3	**171390,3**	168000,2	185784,1	166438,2	**173407,5**	144731,2
AMP-deper	5631,894	5600,467	4996,762	**5409,707**	5415,656	3580,112	4780,281	**4592,016**	5885,497
NAD(P)-bin	490,9037	2313,584	1383,493	**1395,994**	2195,863	1365,196	980,0116	**1513,69**	2556,41
SS2 strictos	18763,96	25832,27	23234,51	**22610,25**	26042,39	20037,21	27658,12	**24579,24**	28079,48
PATL2 PATI	63751,99	76540,56	80688,88	**73660,48**	84706,58	76834,76	103804,9	**88448,75**	89828,93
Dihydrolipo	25071,85	34002,72	36091,94	**31722,17**	36078,47	35700,43	51934,49	**41237,8**	32171,02
ATCYSD1, C	3826,67	3723,263	3298,855	**3616,263**	2763,824	3901,483	3649,024	**3438,11**	3133,305
Ribosomal	29054,13	23931,32	28185,99	**27057,15**	19967,4	35436,93	23349,02	**26251,12**	22333,16
BSK1 BR-sig	121,1543	401,3643	439,0571	**320,5252**	454,9063	304,2124	510,7586	**423,2924**	672,269
SNF4, ATSN	4256,456	4479,578	4836,062	**4524,032**	4822,221	4294,002	5856,646	**4990,957**	5629,01
PGM3 Phos	20038,4	21992,01	22071,2	**21367,2**	23082,64	15828,28	23498,82	**20803,25**	26541,35
HXXXD-typ	516,1757	417,9916	418,0626	**450,7433**	202,8695	359,5228	14,62882	**192,3404**	133,4889
GRF1, GF14	10881,35	9080,54	10657,22	**10206,37**	10620,45	11339,91	11751,93	**11237,43**	10776,22
ATOMT1, C	65335,2	78474,93	80349,81	**74719,98**	85510,93	70518,71	95046,83	**83692,16**	69788,13
TIM, PDTPI	74734,32	75074,06	73218,9	**74342,42**	72469,67	81892,67	76827,61	**77063,32**	73571,34
TCP-1/cpn6	36262,68	40854,89	39592,54	**38903,37**	41823,78	36816,93	46817,75	**41819,49**	42603,06
DEA(D/H)-b	7419,222	6093,191	7785,163	**7099,192**	4506,076	7689,595	5275,307	**5823,659**	5187,587
ATARLA1D,	3624,188	4732,263	6631,856	**4996,102**	10994,7	4501,012	10840,67	**8778,794**	6849,066
NACA3 nas	662,848	633,9172	704,6626	**667,1426**	587,2761	510,7269	579,2135	**559,0722**	508,4174
PMS1 DNA	7200,719	15645,42	16297,98	**13048,04**	13891,96	12700,37	26874,38	**17822,24**	8261,177

ATCAD1, CA	1502,232	1732,854	2416,096	**1883,727**	1098,492	1986,971	795,8462	**1293,77**	1821,856
DOT3 Phot	497,3853	280,131	340,2927	**372,603**	116,098	344,6849	44,17583	**168,3196**	107,4693
Ribosomal	11208,86	16702,55	16916,56	**14942,66**	9162,304	19208,38	8417,513	**12262,73**	8077,35
ATB' GAMM	82,71459	46,58588	122,6864	**83,99561**	43,42399	19,73352	88,15014	**50,43588**	139,9286
PUR4 purin	17920,6	19567,84	21486,12	**19658,19**	23187,27	20408,52	19498,24	**21031,34**	21132,66
GlcNAc1pU	6073,045	3202,336	3619,032	**4298,138**	3586,306	3163,06	3425,59	**3391,652**	4600,622
Protein kin	67,03848	2911,956	2811,266	**1930,087**	1528,433	16,81394	84,71807	**543,3217**	1629,427
VAMP7B, V	3990,007	4297,564	4709,116	**4332,229**	4078,17	6244,233	3900,922	**4741,108**	3485,486
UKL3 uridir	4231,601	5329,486	6156,636	**5239,241**	4739,148	4276,681	2865,157	**3960,329**	4536,732
Protein of u	41860,84	45929,42	49737,84	**45842,7**	51645,98	51135,12	49783,92	**50855,01**	51019,85
ATARCA, RA	213008,1	285080,4	275992,9	**258027,2**	289649,4	242599,5	325792,5	**286013,8**	243583,1
SR1, ATSRP	40,29378	136,0034	188,6841	**121,6604**	119,0089	355,5345	442,6702	**305,7379**	150,5212
Late embry	5099,998	5411,349	5073,446	**5194,931**	4744,034	4194,25	4801,312	**4579,865**	5149,87
RPN12a reg	590,138	735,9225	698,17	**674,7435**	410,4111	789,1199	649,2896	**616,2735**	409,9327
SPDS2 sper	6506,462	6649,564	6643,766	**6599,93**	12424,4	6073,897	10911,68	**9803,327**	10566,3
ATPase, V1	11213,74	11752,62	12956,07	**11974,14**	12140,54	15264,74	12946,72	**13450,67**	15431,58
emb2734 A	31855,51	29470,17	33694,07	**31673,25**	37799,06	25957,36	37361,12	**33705,85**	40320,65
Mov34/MP	22323,16	24747,02	27033,92	**24701,37**	19428,31	23588	24048,3	**22354,87**	24986,83
TOPP4 type	188,5554	63,95118	292,7187	**181,7418**	339,1404	174,0846	480,4705	**331,2318**	335,4795
6-phospho	47449,76	45235,76	47592,01	**46759,18**	42273,46	43292,44	38901,38	**41489,09**	48376,65
MVP1 GDS	6680,199	5012,762	5578,859	**5757,273**	4715,547	4734,953	4026,005	**4492,169**	5258,875
MPPalpha	6331,146	5904,03	7064,476	**6433,217**	6787,63	6668,967	7468,312	**6974,97**	6959,379
FIP1 FH inte	1908,171	2177,387	2220,115	**2101,891**	1815,883	2079,839	3133,957	**2343,226**	1490,039
GDH2 gluta	164817,1	146313,2	151361,1	**154163,8**	133301,7	127506,3	160394,4	**140400,8**	138909,8
Pentatricop	1339,936	1296,129	1471,65	**1369,238**	1851,673	790,0196	1637,392	**1426,361**	1626,079
Saposin-like	661,7126	477,1239	787,7047	**642,1804**	301,3036	544,7147	534,0631	**460,0271**	663,4746
Pyruvate ki	2574,445	2539,976	1085,033	**2066,485**	3651,032	2187,336	5492,394	**3776,921**	3000,03
AHA2, PMA	37872,35	39930,01	42654,73	**40152,36**	39130,99	33919,34	41580,46	**38210,27**	37402,77
NIR1, NIR,	11779,88	15392,42	15199,15	**14123,82**	19147,21	11871,64	20119,36	**17046,07**	17620,1
Peptidase M	2911,631	2599,576	2711,759	**2740,989**	1876,87	3415,402	1407,454	**2233,242**	2977,293
PATL1 PATI	35291,26	42430,63	45990,96	**41237,62**	33204,87	38899,58	37421,86	**36508,77**	35323,24
CINV2 cyto	29,38331	24,36517	59,93133	**37,89327**	154,7329	27,68547	408,6219	**197,0134**	32,13272
RPL16A rib	30974,85	89798,95	87411,57	**69395,12**	115818,4	42197,6	33537,19	**63851,05**	119145,6
Dihydrodip	97,77497	184,247	123,6522	**135,2247**	218,1111	66,77098	207,4515	**164,1112**	261,9091
UDP-Glycos	927,1363	584,9648	749,619	**753,9067**	396,3225	72,13238	617,9449	**362,1333**	367,458
vacuolar AT	175,9446	474,8657	873,6909	**508,1671**	515,3323	2138,16	510,8415	**1054,778**	223,7677
Eukaryotic	174310,2	278931,7	236106,4	**229782,8**	262213,9	269789,3	353482,3	**295161,8**	254938,7
GroES-like	3832,512	5367,007	5500,24	**4899,919**	5620,925	3112,456	5723,762	**4819,048**	6272,553
ubiquitin fa	312,3218	172,3913	89,7087	**191,4739**	419,0814	81,05266	445,4219	**315,1853**	448,594
GRF2, 14-3	695,2423	1879,684	1533,505	**1369,477**	2178,178	1588,528	2962,492	**2243,066**	1551,473
Ubiquinol-c	10021,47	9213,422	9539,516	**9591,468**	3637,289	16107,01	5150,063	**8298,121**	4204,996
unknown p	140,0987	170,794	30,46796	**113,7869**	112,792	158,4032	106,8234	**126,0062**	187,2164
ORP2B OSE	1734,108	835,7062	1511,186	**1360,334**	870,4092	1154,428	949,0197	**991,2856**	935,2619
GAMMA CA	1265,508	1375,929	711,0721	**1117,503**	1877,445	749,6668	2531,099	**1719,404**	2264,287
alpha/beta	406,3795	413,2979	389,8667	**403,1814**	513,2807	518,2238	397,2132	**476,2392**	440,8809
unknown p	6680,689	6211,784	5006,181	**5966,218**	2103,251	9130,113	2449,735	**4561,033**	2649,714
SDG37 SET	1468,054	745,0524	1069,906	**1094,338**	1615,965	695,3125	1771,23	**1360,836**	1984,01
AHA4, HA4	212,1627	162,5583	148,5509	**174,424**	59,45639	203,4791	13,80698	**92,24751**	41,23344
PIP1C, TMP	49433,59	51883,44	67163,38	**56160,14**	44862,51	43321,21	43504,46	**43896,06**	59016,13
RPN8A, AE3	4046,096	4365,098	3781,977	**4064,39**	5117,753	3516,784	5522,78	**4719,106**	3702,767

IAR4 Thiam	33652,2	29401,65	30821,08	**31291,64**	26359,2	33507,93	27352,56	**29073,23**	25524,88
CRT1, CRT1	11894,99	12916,49	12445,76	**12419,08**	8035,766	21321,4	17692,87	**15683,34**	7886,37
Plant neutr	248,6518	229,8076	210,3189	**229,5927**	60,64479	182,9167	2,707491	**82,08967**	46,02075
Plant neutr	2623,345	2504,273	2223,577	**2450,399**	2774,303	2193,969	1936,414	**2301,562**	2769,774
Oxidoreduc	3,159739	159,7376	176,6004	**113,1659**	121,356	562,2476	204,1064	**295,9033**	205,9005
mtHsc70-1	31873,94	35542,15	35278,32	**34231,47**	42028,46	33029,3	43035,77	**39364,51**	37490,23
NAD(P)-bin	30236,43	33594,69	35428,42	**33086,51**	17318,87	33816,07	22535,81	**24556,91**	23935,67
RD2 Adenir	11932,84	9897,672	12185,24	**11338,58**	4283,234	10744,11	5833,311	**6953,55**	4011,222
Pyridoxal p	4854,806	5357,4	5731,06	**5314,422**	5890,988	3410,78	4236,108	**4512,625**	4059,228
G6PD3 gluc	13461,53	1974,502	2300,215	**5912,084**	1995,651	1754,805	2140,61	**1963,689**	1548,577
Class II ami	63315,38	63782,49	69433,92	**65510,6**	53827	62883,09	61198,2	**59302,76**	64064,1
NAD7 NAD	14369,49	17366,61	17868,39	**16534,83**	19005,21	16901,67	23194,78	**19700,55**	19104,57
PHT3;1 pho	117323,8	153741,5	136902,9	**135989,4**	167920,1	134071,3	167920,1	**156637,2**	137147,2
EMB2733,	3592,803	4269,78	5776,338	**4546,307**	1175,785	6451,13	628,1379	**2751,684**	1829,925
PYK10, PSR	1025969	1081945	995256,8	**1034390**	893617,5	1558585	1506023	**1319408**	689747,2
AtRABA1e,	745,6707	10473,62	12187,54	**7802,278**	4855,53	1268,673	949,6003	**2357,934**	7862,547
PYR4, DHO	1537,212	1121,39	1274,09	**1310,897**	470,0028	923,4707	503,4992	**632,3242**	683,2878
CARA carba	8269,912	10342,31	11848,19	**10153,47**	10579,69	9079,83	9154,085	**9604,537**	8882,599
CID10 CTC-	177,4612	375,596	1087,124	**546,7269**	139,2543	256,7998	149,11	**181,7214**	277,2078
UXS1, ATU)	14767,59	13007,58	16242,84	**14672,67**	9676,387	18115,59	9361,347	**12384,44**	10066,52
RING/U-bo	8231,074	4706,655	5544,963	**6160,897**	5127,255	2246,197	2521,477	**3298,31**	5223,625
NAD(P)-bin	5330,894	6163,279	5901,156	**5798,443**	6654,203	5899,244	7417,043	**6656,83**	5649,987
Adenine nu	143,4202	295,2607	337,2015	**258,6275**	311,4435	748,0217	344,428	**467,9644**	249,5739
HOG1, EMI	238630,9	234554,7	239229,9	**237471,8**	339092,8	181012,1	286374,6	**268826,5**	293337,3
Transducin	2481,238	2674,307	2842,14	**2665,895**	3787,803	2010,813	3960,498	**3253,038**	4222,283
Cleavage ar	501,6296	1028,177	844,8732	**791,5598**	664,4387	1390,852	1966,031	**1340,44**	719,2677
ATNAP8, N	939,8769	762,7546	941,2118	**881,2811**	1089,751	1324,167	850,8571	**1088,258**	964,485
Homeodon	4077,41	5286,546	4159,243	**4507,733**	2501,006	5295,907	1389,093	**3062,002**	3159,851
D-alanine--	535,5499	314,6339	664,1543	**504,7794**	757,3842	1020,527	510,5339	**762,8149**	664,2795
CBL cystath	1749,835	1321,445	1246,9	**1439,393**	1558,256	1363,091	1414,977	**1445,441**	1016,782
ATRAB7, A1	360,2407	555,4632	568,2322	**494,6454**	548,5574	593,5264	842,2419	**661,4419**	584,7101
BETA-UP, P	1375,265	2020,188	2306,486	**1900,646**	1940,836	1445,39	2248,454	**1878,227**	1474,158
AK-HSDH II	515,693	527,1109	504,8389	**515,8809**	764,9536	460,9672	657,1464	**627,6891**	551,8163
Bifunctiona	485,0268	672,7485	977,7826	**711,8526**	460,482	1895,919	359,5305	**905,3106**	288,7292
NAD(P)-bin	5419,248	2404,938	4595,13	**4139,772**	2480,041	3021,734	2836,817	**2779,53**	4138,719
TRAF-like fa	280993,4	325291	333560,5	**313281,6**	278424,6	288254,7	324174,2	**296951,2**	293115,7
BGLU34, TC	30235,03	28509,06	32407,25	**30383,78**	28764,94	37954,76	41128,1	**35949,27**	33476,04
ATPHB3, PI	852,8408	539,072	578,4777	**656,7968**	251,3435	680,6253	365,645	**432,5379**	549,0556
Family of u	91066	228720,4	173373,9	**164386,8**	158115,6	264072,9	234260,7	**218816,4**	223879,2
Plant neutr	163,0655	516,152	408,3794	**362,5323**	533,0636	335,8738	212,1015	**360,3463**	550,7913
PHT3;2 pho	2314,24	1219,366	2054,174	**1862,593**	2326,438	2046,36	3007,377	**2460,058**	1814,253
NAD-ME2 I	8666,054	9500,699	7799,392	**8655,382**	13751,51	7878,087	14897,32	**12175,64**	10774,15
G6PD6 gluc	15243,07	15274,91	15988,65	**15502,21**	18521,31	10250,12	20260,84	**16344,09**	19752,05
tetratricop	218,0401	567,1717	151,7534	**312,3217**	437,533	229,3184	1203,696	**623,5159**	808,5895
SYTD, ATSY	1528,205	530,7135	667,3218	**908,7466**	562,5191	1000,515	599,1707	**720,735**	422,9059
LRR1 Leucir	4026,11	4118,185	4013,551	**4052,615**	3978,296	7171,452	3951,597	**5033,782**	5555,762
mMDH1 La	114830,1	115344,9	126242,3	**118805,8**	159867,1	88352,53	141564,4	**129928**	162039,9
Quinone re	8957,826	11743,1	11927,9	**10876,28**	11014,48	8591,779	12536,8	**10714,35**	8126,938
HD2B, HDT	5292,801	3484,564	4936,312	**4571,226**	6582,368	3818,889	9085,291	**6495,516**	4339,835
AICARFT/IN	2008,492	1431,653	2209,652	**1883,266**	844,3524	2442,816	843,0148	**1376,728**	844,6137

ACT domai	31926,87	26270,91	28786,29	**28994,69**	16329,53	39006,08	16886,88	**24074,17**	16812,84
UBP14, TTN	2173,269	2644,597	2403,199	**2407,022**	3229,748	1571,696	2084,156	**2295,2**	2619,868
UDP-glucos	20734,31	24448,02	24337,97	**23173,43**	36866,89	18578,49	30805,51	**28750,29**	32191,88
DGL1 dolicl	24699,89	25327,82	22383,88	**24137,19**	28602,81	24831,73	32979,86	**28804,8**	30177,09
GAD3 gluta	1084,065	1069,911	1349,392	**1167,789**	1417,241	1220,383	1432,659	**1356,761**	1431,853
Aluminium	25857,21	24038,65	23950,69	**24615,51**	37454,06	21808,28	35583,91	**31615,42**	26322,07
transducin	145,0171	994,5001	1340,279	**826,5987**	1251,591	837,5945	1697,117	**1262,101**	1602,354
NAD(P)-bin	16354,22	16165,51	12710,16	**15076,63**	9386,504	17340,39	6691,494	**11139,46**	10931,54
RNA polym	2329,313	2347,434	2920,759	**2532,502**	2170,276	2256,111	3176,801	**2534,396**	2149,4
MTO3, SAM	84658,52	128671,1	96351,43	**103227**	126651,1	88344,7	151316,8	**122104,2**	90479,81
Sec14p-like	634,3235	700,5608	939,4455	**758,1099**	827,4511	551,3209	1356,02	**911,5974**	1092,262
LIN2, HEMI	603,4702	315,9045	463,6186	**460,9978**	464,4044	329,7033	414,567	**402,8916**	506,0909
Clathrin ad	773,2797	1077,574	1122,543	**991,1324**	1309,729	745,0432	1617,774	**1224,182**	1427,991
JR1 Manno	43002,49	52713,03	48951,08	**48222,2**	47351,65	46876,6	61304,18	**51844,14**	41780,37
AAC2 ADP/	2896,921	1776,604	2550,553	**2408,026**	1569,115	2685,315	1146,384	**1800,271**	2415,621
ACT3 actin	2859,138	3468,326	5256,438	**3861,301**	3170,716	2740,933	2939,754	**2950,468**	3392,064
ROC4 rotar	7375,46	6492,04	7753,069	**7206,856**	7004,707	8150,936	7758,797	**7638,147**	7094,852
HEME2 Urc	9973,86	10989,79	13329,47	**11431,04**	24990,14	9693,788	24590,85	**19758,26**	15202,14
Chalcone-fl	4806,711	2741,382	3707,363	**3751,819**	3575,835	4043,45	4007,091	**3875,459**	3474,134
SOT16, ATS	2064,511	1203,007	1285,531	**1517,683**	1651,553	3077,381	1915,166	**2214,7**	1639,089
NSP4 nitrile	11940,61	11387,95	10500,1	**11276,22**	15653,73	9122,253	15615,77	**13463,92**	9976,841
TRN1, ATTF	15727,16	16294,21	16986,3	**16335,89**	12078,8	18367,65	7838,266	**12761,57**	12044,18
Succinyl-Co	1112,927	6735,099	5563,773	**4470,6**	8860,825	5510,329	9072,533	**7814,562**	6088,19
AtGLDP2, G	8087,235	8083,338	9711,841	**8627,471**	14784,05	6847,219	13848,44	**11826,57**	12668,62
FSD1, ATFS	9256,037	4955,714	3961,029	**6057,593**	3011,08	12909,87	4161,653	**6694,2**	2448,407
DRP3B dyn	4354,368	533,854	534,7691	**1807,664**	830,3213	3270,284	1622,832	**1907,813**	564,3267
GSA2 gluta	546,7488	847,8195	277,8455	**557,4713**	174,7998	267,7807	333,7667	**258,7824**	161,9012
Protein of u	9,006506	796,9881	795,8549	**533,9498**	46,75966	16,13076	67,67182	**43,52075**	265,7199
CHLI2, CHL	97,07241	49,46413	74,35725	**73,63127**	86,13868	27,01915	114,7279	**75,96192**	101,1145
TSA1, TRP3	8072,292	61879,35	62750,96	**44234,2**	80739,65	38565,09	88365,41	**69223,38**	23940,8
peptidase M	5418,914	6006,185	6486,196	**5970,432**	8460,871	4724,012	7699,664	**6961,516**	4921,529
Oligosaccha	13383,56	16614,15	14293,04	**14763,58**	8698,995	24580,06	8105,97	**13795,01**	8956,139
MLP328 M	100426,6	82422,21	87357,54	**90068,79**	38289,47	143006,3	55126,03	**78807,26**	31798,47
SYTA, NTM	1227,718	1313,179	527,139	**1022,679**	1380,71	411,7695	1995,392	**1262,624**	573,6232
SAHH2, ATS	17760,56	5622,913	6484,486	**9955,987**	10177,84	11629,75	14233,29	**12013,63**	6923,708
unknown p	187,8675	928,0172	265,6533	**460,5127**	491,0098	1045,839	974,471	**837,1067**	496,4423
Pyruvate ki	1857,595	460,2594	416,3599	**911,4048**	502,9143	1797,091	2317,649	**1539,218**	502,262
RNA-bindin	732,6919	674,5656	333,676	**580,3112**	188,4038	632,5016	23,7874	**281,5643**	202,0261
pfkB-like ca	12604,61	10590,08	11701,47	**11632,05**	9150,684	11444,22	7357,374	**9317,425**	10752,67
BEST Arabi	675,6644	2053,009	2844,716	**1857,796**	3343,692	1967,884	4363,302	**3224,959**	4682,246
Protein of u	1947,741	1429,197	1838,157	**1738,365**	2170,61	1487,863	2606,401	**2088,292**	1348,74
ATPase, V0	12666,62	17292,42	18092,84	**16017,29**	37179,56	12607,74	30757,95	**26848,42**	22951,46
Protein of u	3463,924	6284,946	5390,071	**5046,314**	5226,858	6339,091	4401,72	**5322,556**	4195,237
Protein of u	5367,718	3039,451	4215,189	**4207,452**	3967,224	7048,523	5967,597	**5661,115**	4377,27
60S acidic r	4220,484	3647,044	3768,318	**3878,615**	4711,514	3397,601	4570,667	**4226,594**	4421,992
HSP20-like	42783,73	67122,62	53861,71	**54589,36**	80760,75	54935,96	76248,32	**70648,34**	68984,23
Ribosomal	23105,15	23619,45	25305,4	**24010**	33496,84	22070,08	33043,31	**29536,74**	27940,71
Translation	6939,151	7095,51	7080,268	**7038,309**	7347,573	5223,559	6123,706	**6231,613**	6611,726
ATRAB11C,	360,5924	164,1787	266,6144	**263,7952**	242,7936	6,933926	343,2941	**197,6739**	497,1643
unknown p	6151,664	3647,402	4352,137	**4717,068**	2668,534	6265,928	2491,135	**3808,532**	2871,026

EMB2761 t	2948,911	4375,867	4838,276	**4054,351**	8329,26	1647,242	5780,41	**5252,304**	7174,205
ERMO2, SE	9729,616	11200,69	9780,095	**10236,8**	11969,6	8995,35	11992,61	**10985,85**	9268,371
ATGSTF10,	87864,58	78338,22	77930,07	**81377,62**	70627,4	79321,03	57836,29	**69261,58**	83547,16
GAMMA C/	11511,15	13063,37	15351,01	**13308,51**	14891,63	10965,04	15102,38	**13653,02**	15996,68
ATRAB8D, /	27483,87	36221,49	35861,85	**33189,07**	30709,47	30469,83	27241,32	**29473,54**	30431,79
FAD-linked	392,324	322,5647	361,349	**358,7459**	120,3139	406,3805	174,1157	**233,6034**	287,1758
SBP (S-ribo	145,3868	312,2852	424,033	**293,9017**	43,66289	485,7235	24,63343	**184,6733**	25,91559
Peptidase (	957,8545	918,0736	721,8556	**865,9279**	765,5261	983,046	497,5254	**748,6992**	676,3871
XH/XS dom	2981,819	23136,72	18035,16	**14717,9**	30314,2	2513,572	1859,772	**11562,51**	22111,31
Cysteine pr	5166,265	4280,456	5005,695	**4817,472**	5319,669	4340,409	5823,011	**5161,03**	4635,87
PDF1A, ATI	7162,869	8939,238	9959,002	**8687,036**	10263,69	7503,296	10709,19	**9492,06**	9957,479
CARB carba	32762,04	42029,51	40970,74	**38587,43**	34726,92	43128,95	35516,15	**37790,67**	27482,11
Pentatricop	162,5456	11,07207	26,35937	**66,65903**	36,90639	32,32923	27,09267	**32,10943**	86,06852
CRU2, CRB	58,3939	23,47829	63,99434	**48,62218**	11,48466	1659,028	1813,189	**1161,234**	5,902381
SDH1-1 suc	17221,94	19178,8	21398,48	**19266,41**	19802,1	16385,04	22017,79	**19401,64**	23386,38
emb1624 T	1943,844	3756,664	3307,332	**3002,613**	3770,394	3199,696	3552,053	**3507,381**	3572,712
2-oxoglutai	19356,95	25390,49	26359,68	**23702,37**	22518,49	21776,94	24061,12	**22785,52**	25585,08
CCR1, ATGI	7511,466	9331,984	7337,721	**8060,39**	7993,466	7580,676	10435,73	**8669,959**	11757,43
CLPC, ATHS	5746,183	7130,959	6586,366	**6487,836**	6819,655	6185,948	6653,039	**6552,881**	6827,103
ATXTH17, X	322,3858	6819,211	10140,22	**5760,605**	8120,221	58,59012	6,062313	**2728,291**	3090,421
Basic-leucir	13174,9	5999,973	7920,257	**9031,711**	6819,961	6595,783	6963,983	**6793,242**	9484,756
GSH1 gluta	4929,483	3494,248	3376,812	**3933,514**	4386,034	2705,871	4091,899	**3727,935**	5293,162
Adaptin far	8055,337	10220,1	11468,8	**9914,747**	11490,22	10754,39	11401,28	**11215,3**	10420,15
PLATZ tran:	146,8553	2158,119	1601,386	**1302,12**	2410,587	1548,464	2443,467	**2134,173**	2429,913
NAD(P)-bin	16827,96	19725,66	23859,47	**20137,69**	21731,82	18286,64	25165,1	**21727,85**	23376,58
ACT domai	1622,704	1191,51	1825,777	**1546,664**	956,0742	1272,252	731,1645	**986,4969**	884,5003
MA3 doma	1440,875	1663,336	1449,574	**1517,928**	2419,456	1170,194	1829,441	**1806,364**	1895,847
CPN20, CPI	28700,46	27842,59	30926,82	**29156,62**	21055,66	32159,2	21021,83	**24745,56**	27515,21
CONTAINS	964,4809	471,8734	521,0082	**652,4542**	400,7667	627,0018	245,4234	**424,3973**	570,5135
Granulin re	24349,69	24099,15	23701,41	**24050,08**	23161,8	26251,69	30341,12	**26584,87**	26018,72
UGP, UGP1	41310,06	27749,02	35122,64	**34727,24**	22757,8	32543,65	25709,87	**27003,78**	23175,46
GAPC, GAP	103587,8	93875,15	92253,26	**96572,08**	166939,8	89194,12	225873,6	**160669,2**	120085
Phosphoin	26,30789	420,2543	1321,518	**589,3602**	499,5118	67,22201	7,340418	**191,3581**	446,8661
FUNCTION:	2270,806	2700,393	3061,516	**2677,572**	3006,8	2844,764	7884,275	**4578,613**	3543,137
unknown p	1385,89	2783,626	2868,276	**2345,931**	3922,215	2219,048	4023,48	**3388,248**	2754,242
CPN60B, LE	343,5546	48,93453	164,4676	**185,6522**	268,7338	172,4034	653,9987	**365,0453**	710,6565
ATUBA1, M	53219,4	51764,39	49210,67	**51398,15**	71820,11	47641,88	53529,11	**57663,7**	48330,32
EMB2024,	3208,2	3302,247	3782,937	**3431,128**	2853,866	2950,264	2499,193	**2767,774**	2510,765
ALDH12A1,	13476,55	11422,38	11120,84	**12006,59**	12351,2	9791,643	11570,25	**11237,7**	12623,23
NAD(P)-bin	794,5812	1331,12	1079,296	**1068,332**	277,5693	1403,886	472,4425	**717,966**	1129,745
SUS4, ATSU	47,97481	13,13739	22,39303	**27,83508**	53,68339	36,96926	71,30043	**53,98436**	17,96014
LPD1, ptlpc	26891,79	26856,53	22861,51	**25536,61**	16137,94	46990,88	20154,67	**27761,16**	11872,98
CAC2 acety	14111,9	14158,29	15537,4	**14602,53**	16909,3	13328,18	17349,21	**15862,23**	14177,61
AFC2, AME	898,6469	1240,345	1707,093	**1282,028**	3276,782	847,5621	4450,795	**2858,38**	2764,978
JAL31 jacal	7799,744	5891,705	6944,6	**6878,683**	5306,682	4538,366	8748,694	**6197,914**	7342,922
Cysteine pr	3024,678	2055,886	2338,984	**2473,183**	1624,646	2858,768	3066,951	**2516,788**	1737,434
ATPME2, P	2437,106	2106,618	2459,032	**2334,252**	1972,908	2325,134	2448,84	**2248,961**	2122,899
CSY2 citrat	7194,742	7080,296	6797,407	**7024,148**	5591,166	6898,025	6656,505	**6381,899**	6556,268
ALDH2C4, /	49785,15	47246,52	46114,95	**47715,54**	51048,94	44968,08	44256,69	**46757,9**	38972,56
ALPHA-DO)	40498,33	38062,15	36654,44	**38404,97**	37658,76	34281,38	30226,52	**34055,55**	40597,24

AtUGT85A2	3405,585	6364,855	4969,639	**4913,36**	6964,639	4321,345	6065,959	**5783,981**	4253,33
Cytosol am	104962	115434	113677,7	**111357,9**	135464,9	106713,5	134289,7	**125489,4**	122278,1
ATRAB11A,	137,3479	179,4327	260,8323	**192,5376**	495,6289	25,88273	514,1548	**345,2221**	541,2787
RNA bindin	66,03844	97,52055	65,44452	**76,3345**	115,1454	29,48705	164,8369	**103,1564**	179,8668
EIF2 GAMM	34270,36	45616	40245,74	**40044,03**	44021,71	38351,69	51648,38	**44673,93**	39737,86
Copper am	1615,926	1704,662	2052,522	**1791,037**	2218,694	1706,394	2324,198	**2083,095**	2767,805
UBQ6 ubiq	2256,913	5565,582	5683,134	**4501,876**	6003,739	6269,853	6387,158	**6220,25**	7718,104
S-locus pro	1348,254	2564,132	2787,401	**2233,262**	3753,063	2780,556	2544,218	**3025,946**	2412,841
PDH-E1 ALI	17204,53	23437,7	20775,52	**20472,58**	21596,83	21515,34	27500,24	**23537,47**	21706,41
Translation	65403,18	61644,26	63165,08	**63404,17**	50863,59	66140,08	76134,71	**64379,46**	60085,59
Protein of |	1929,466	750,7085	836,1232	**1172,099**	1345,658	309,7554	1015,615	**890,3428**	1753,572
UBP6, ATU	4473,033	4872,131	1574,198	**3639,787**	2424,597	936,4542	3934,503	**2431,851**	1414,744
HXXXD-typ	45856,14	38595,47	46127,89	**43526,5**	42400	36247,07	41920,05	**40189,04**	46568,97
Proteasom	2616,157	3154,42	2475,27	**2748,616**	3518,35	2326,638	4504,557	**3449,848**	3716,286
ATNUC-L1,	7636,531	4789,631	5115,569	**5847,244**	9014,137	5780,189	9372,518	**8055,615**	6978,573
unknown p	360,1904	219,0102	243,8748	**274,3585**	315,9048	341,0284	476,4216	**377,7849**	215,0628
ATBCA4, B(	17566,49	19543,97	18511,28	**18540,58**	16229,67	17492,66	16138,57	**16620,3**	16991,74
emp24/gp2	2988,773	1587,525	2173,978	**2250,092**	3175,928	2223,398	4594,546	**3331,291**	2741,752
AtRABA6b,	824,4733	880,8073	779,0539	**828,1115**	604,9775	986,8056	312,5116	**634,7649**	596,2022
Mannose-b	13005,42	18649,68	15160,87	**15605,33**	16416,84	15608,36	14291,64	**15438,95**	12836,65
SDH2-1 suc	859,0365	142,0683	1094,961	**698,6886**	549,0838	1634,156	1108,538	**1097,259**	970,2234
Glycosyl hy	195009,9	198513,5	189675,9	**194399,8**	193398,9	180466,4	205419,9	**193095,1**	193965,6
HSP60-3A I	9827,026	11879,31	10459,6	**10721,98**	13774,71	10376,17	12800,02	**12316,97**	11549,95
BGLU19 be	7970,813	7092,494	4112,417	**6391,908**	5617,172	7538,718	4496,711	**5884,2**	5284,674
Endomemb	4378,451	6107,405	4880,599	**5122,152**	5252,56	6966,452	4449,354	**5556,122**	4869,354
CAD4, ATC/	104335,9	134856,1	145528	**128240**	176966,5	123307,2	163162	**154478,5**	138310
Alkaline-ph	62,3443	74,85959	93,68105	**76,96165**	181,9963	6,06853	255,3821	**147,8157**	236,3074
UVR8 Regu	70,68093	428,0362	365,138	**287,9517**	361,2303	317,9364	505,1897	**394,7855**	431,551
Granulin re	7405,621	6809,041	9169,423	**7794,695**	8268,213	6954,524	9808,071	**8343,602**	10128,15
RAN2 RAS-I	48339,61	44155,08	43901,27	**45465,32**	35758,8	41759,34	39664,4	**39060,85**	41982,97
ATSBT4.12,	62932,21	63228,96	59854,62	**62005,26**	54953,65	76623,59	78598,05	**70058,43**	61834,38
MVA1, HM	15607,89	19290,21	20101,28	**18333,12**	21084,18	18220,34	20696,33	**20000,28**	17794,84
Lipase/lipo	16874,81	15542	16758,21	**16391,68**	17315,52	15251,03	19727,7	**17431,42**	17687,69
Disease res	5892,702	4513,995	4067,662	**4824,786**	3885,823	5543,984	4279,944	**4569,917**	3826,428
SIR sulfite r	47216,18	48352,71	54119,96	**49896,28**	54998,51	45987,22	50619,59	**50535,11**	57192,35
UGT74B1 U	0,422894	644,2436	672,8072	**439,1579**	1031,298	872,8667	813,9448	**906,0364**	579,8992
Anion-trans	3526,045	1931,977	2479,116	**2645,713**	1057,867	4000,342	990,1606	**2016,123**	1395,105
unknown p	8787,851	6676,236	7643,774	**7702,62**	7111,423	6146	7299,393	**6852,272**	6697,397
protein kin	11164,23	16129,84	17776,19	**15023,42**	9906,527	18571,3	9217,329	**12565,05**	11828,34
ATRABB1B,	1749,705	844,5486	1441,978	**1345,41**	1143,24	521,3432	1093,601	**919,3948**	1189,99
PRX33, PRX	481,7398	187,9177	167,4872	**279,0482**	649,6487	255,4191	387,3579	**430,8086**	695,8593
P5CR, AT-P	8906,58	10800,72	11667,54	**10458,28**	14083,28	10112,66	11161,09	**11785,68**	8214,627
MEF22 mit	122,9129	129,692	83,53177	**112,0456**	223,7076	85,81921	531,1162	**280,2143**	153,9181
SBP1 selen	30642,79	38502,41	41359	**36834,73**	32680,57	33421,58	29688,53	**31930,22**	33660,31
NAD9 NAD	9771,128	5082,633	4587,706	**6480,489**	4348,492	5596,091	2950,427	**4298,337**	5322,884
ACS acetyl-	14165,88	8062,112	7465,015	**9897,669**	8302,74	9212,417	6224,844	**7913,334**	7845,883
TUB8 tubul	1491,319	2943,22	2992,33	**2475,623**	3482,436	2205,807	3694,113	**3127,452**	2254,56
Ubiquitin c	13885,73	10357,85	8242,179	**10828,59**	11674,66	7954,772	9686,115	**9771,851**	12476,33
GT, UGT74I	17,35125	242,588	171,2991	**143,7461**	579,6346	165,1837	231,6072	**325,4752**	107,068
ARM repea	988,3718	1259,698	1248,904	**1165,658**	1664,581	1004,964	1821,289	**1496,945**	1839,477

Transketola	62875	84478,73	81827,15	**76393,63**	115284,6	68281,02	112208	**98591,21**	92129
NAD(P)-bin	824,3266	1891,581	1847,082	**1520,997**	2124,183	1318,871	1698,591	**1713,882**	904,7045
KASI, KAS1	63260,82	87276,77	93813,89	**81450,49**	112198,7	70559,91	123900,9	**102219,8**	78805,92
Pyruvate ki	13766,63	19511,3	12608,87	**15295,6**	19870,52	10717,49	23439,47	**18009,16**	11295,13
Polynucleo	3133,543	2664,741	3126,832	**2975,039**	1813,247	4073,149	1493,073	**2459,823**	2181,874
ATMS3, MS	10633,26	9350,003	8961,643	**9648,302**	9589,71	8950,316	6810,974	**8450,333**	9175,722
Glutamyl/g	6388,644	7212,388	7760,911	**7120,648**	8221,952	5784,35	6073,541	**6693,281**	8485,066
Late embry	65402,99	70248,83	59612,04	**65087,95**	76554,84	61875,98	84777,73	**74402,85**	68417,57
Glycosyl hy	20305,14	18741,01	20153,22	**19733,13**	15219,14	20539,45	17116,65	**17625,08**	17154,12
ZCW7 ZCW	770,5332	575,4052	557,2793	**634,4059**	726,9798	0,61192	559,0691	**428,887**	826,194
Protein of u	3270,285	7148,044	6668,405	**5695,578**	8081,255	5856,293	10748,67	**8228,738**	5398,657
PAA2 20S p	484,9435	602,2801	1075,199	**720,8076**	954,7093	753,5449	778,4968	**828,917**	949,8454
Transketola	874,4111	2798,612	1482,583	**1718,535**	2019,953	2459,757	2307,553	**2262,421**	2055,301
Melibiase f	40139,69	37225,47	44796,61	**40720,59**	37263,37	32903,15	38311,57	**36159,36**	42122,41
ATDABB1, I	4074,289	22238,89	21186,36	**15833,18**	27669,08	3926,036	4280,302	**11958,47**	20703,96
phytochror	4278,448	4716,633	4039,989	**4345,023**	1502,157	7099,412	1687,662	**3429,744**	2489,962
TSBtype2 t	485,1194	1256,251	1438,8	**1060,057**	397,1101	1485,601	113,9464	**665,5526**	322,5736
APA1, ATAI	99822,79	152196,5	140981	**131000,1**	192816,3	112281	208823,2	**171306,8**	171029,9
AtGRF7, GF	29,96154	100,2391	342,8254	**157,6753**	159,4843	8,267226	85,55686	**84,43614**	217,055
ATFD3, FD3	17606,98	21488,52	28106,42	**22400,64**	23459,13	18159,45	20852,36	**20823,65**	17170,63
RmlC-like c	765,9296	1221,17	1549,411	**1178,837**	1701,119	290,4256	2545,114	**1512,22**	2370,487
ATKDSA2 A	339,2265	1005,66	657,196	**667,3609**	1201,479	638,1485	1098,268	**979,2984**	570,3516
NSP3 nitrile	14458,26	9517,961	10875,56	**11617,26**	6808,514	18988,53	7900,498	**11232,51**	7204,035
Amino acid	24960,21	27874,64	25967,86	**26267,57**	32030,9	23732,66	43347,06	**33036,87**	28674,59
LOS2, ENO2	699185,3	749448,9	741900,3	**730178,2**	938549,3	666247,9	988490,2	**864429,1**	851037,5
ADL3, CF1,	7293,941	8716,186	8403,298	**8137,808**	6541,617	6157,921	9511,919	**7403,819**	6001,757
jacalin lecti	137,2641	62,16828	179,3291	**126,2538**	78,70227	139,7451	64,51925	**94,32222**	103,2516
FCLY farnes	4,085532	476,1098	346,8772	**275,6908**	712,7041	292,1215	568,4739	**524,4332**	674,316
ATSLY1 Sec	121,8465	344,0913	321,9877	**262,6419**	275,1772	336,5235	292,1257	**301,2754**	424,8322
SMT2, CVP	505,5802	374,3698	822,3304	**567,4268**	922,4226	471,7002	636,1016	**676,7415**	506,1421
CNX1, ATCI	2219,437	2850,475	2739,666	**2603,193**	4010,533	2221,052	4813,686	**3681,757**	3240,983
FAB1, KAS2	2147,301	2874,538	3302,885	**2774,908**	4524,551	1713,732	4118,272	**3452,185**	1766,198
ACT11 actii	1537,579	1665,639	2167,998	**1790,406**	1837,309	1191,406	2201,499	**1743,404**	2319,653
APT1, ATAF	289,1078	247,5593	565,632	**367,4331**	656,1673	376,4099	721,9825	**584,8532**	708,5827
RING SBP (S	3402,096	4475,831	4139,442	**4005,79**	2906,982	3550,03	3820,17	**3425,727**	2845,239
Protein kin	2711,96	10501,03	10163,4	**7792,127**	12620,3	8830,498	13962,27	**11804,36**	12802,52
Ribosomal	3681,148	6263,262	6812,646	**5585,686**	11101,72	5026,435	7726,251	**7951,467**	12097,92
NAGK N-ac	4201,962	4034,702	4363,603	**4200,089**	6266,892	3050,504	4928,637	**4748,678**	5427,33
Aldolase su	4596,722	5901,024	5259,213	**5252,32**	4671,012	5668,977	3504,308	**4614,766**	4906,161
SNC4 suppr	4744,01	6505,731	6384,333	**5878,025**	4731,568	6484,414	4708,124	**5308,035**	4883,853
SPFH/Band	1263,778	814,9047	1707,981	**1262,221**	1530,011	1723,171	1508,405	**1587,196**	1265,162
TCP-1/cpn6	19027,31	15832,01	5355,318	**13404,88**	14149,24	152,0072	7346,364	**7215,871**	12510,45
Alpha-1,4-g	6799,87	19342,86	18001,28	**14714,67**	12661,8	6102,493	7097,435	**8620,575**	9209,134
APFI, GAMI	25718,04	26976,43	28701,97	**27132,15**	33939,56	24610,87	35137,13	**31229,19**	32984,49
SAM1, SAM	11366,27	18882,39	11941,86	**14063,51**	21357,7	11701,65	30341,78	**21133,71**	12861,84
Sec23/Sec2	34471,99	50760,4	28243,96	**37825,45**	29971,52	47251,16	15904,8	**31042,5**	26234,34
Dihydrolipo	2127,673	1770,619	2292,965	**2063,753**	3222,485	434,1852	4548,146	**2734,939**	4498,279
ATARP4, AF	3562,26	4264,904	4722,32	**4183,161**	5261,244	3340,957	4904,69	**4502,297**	5058,124
ATTIM23-2	1050,184	2149,651	1786,719	**1662,185**	1921,753	2254,11	2001,472	**2059,112**	1745,219
FAD/NAD(F	563,3719	1989,532	1443,139	**1332,014**	1334,307	5140,663	2661,016	**3045,329**	766,9946

ACX3, ATA(	2114,715	1621,674	2096,611	**1944,333**	2292,914	1306,312	2240,819	**1946,682**	2705,396
SKP1, ASK1	1654,165	2592,889	3155,419	**2467,491**	3757,749	2693,725	3456,957	**3302,81**	2122,455
3-dehydro(	4835,492	3796,039	3659,32	**4096,95**	7574,425	3495,721	3812,016	**4960,721**	7466,449
Ribosomal	25557,87	25242,13	28322,22	**26374,08**	24781,84	31617,8	40693,4	**32364,35**	25482,35
Leucine-ric	1203,719	2255,347	2270,012	**1909,693**	1424,807	1557,888	1308,199	**1430,298**	1701,091
PCNA1, ATI	866,4994	1514,778	1447,372	**1276,216**	3439,297	980,6577	2600,255	**2340,07**	2132,172
ATARFA1D,	135812,9	109202,8	105231,2	**116749**	150745,3	105986	190286,5	**149006**	131097,1
AFP1 ABI fi	8012,607	2641,581	4983,106	**5212,431**	5378,975	2296,284	6254,785	**4643,348**	8888,427
Class II aaR	20240,34	20491,05	16402,04	**19044,48**	15884,05	20413,33	12714,14	**16337,17**	16776,05
ATCRSH, CF	2403,395	1544,259	923,3935	**1623,683**	1147,38	2292,794	914,5524	**1451,575**	2161
Ribosomal	8396,129	9260,091	10750,55	**9468,925**	8473,244	24547,46	19652,91	**17557,87**	8321,135
LOCATED II	60682,54	67459,91	70503,54	**66215,33**	68987,71	60654,23	71879,5	**67173,81**	71907,27
transducin	8985,729	8194,322	8870,485	**8683,512**	8133,794	9437,803	12451,9	**10007,83**	9093,822
Coatomer (	662,7726	790,73	916,996	**790,1662**	991,0212	992,6544	595,0172	**859,5643**	602,4308
HSL2 HAES/	1258,798	1328,601	1560,875	**1382,758**	969,6674	1662,405	1343,137	**1325,07**	1249,612
Phosphogly	120731,3	129713,6	137925,3	**129456,8**	168707,8	118948,8	175155	**154270,6**	160209
UBC11, ATU	2585,463	8745,058	9064,078	**6798,2**	7878,151	12781,72	11370,86	**10676,91**	5868,454
Peroxidase	31084,95	30855,24	32342,27	**31427,48**	29449,77	37693,56	42137,43	**36426,92**	41486,02
ALATS, ACD	45119,03	43164,62	44184,61	**44156,09**	44173,48	35162,26	41707,76	**40347,83**	45796,02
GTP bindin	27741,7	30565,68	29625,01	**29310,8**	29057,53	30593,88	32911,24	**30854,22**	26553,7
ORP3B OSE	18359,79	28953,14	23782,43	**23698,45**	16507,86	25380,98	14643,64	**18844,16**	20950,01
endoribonu	18044,42	16621,8	18727,82	**17798,01**	19124,85	14358,89	18683,01	**17388,92**	22400,08
Ribosomal	9169,207	10667,07	10480,19	**10105,49**	8398,301	14878,3	12521,01	**11932,54**	8962,488
SPFH/Band	155,6174	261,6147	304,1455	**240,4592**	613,5616	150,2911	555,3576	**439,7368**	311,7192
TPX1 thiore	34360,97	41745,15	38959,66	**38355,26**	30571,49	39124,95	29101,94	**32932,79**	25696,14
PHS1 poor	444,6547	1537,192	711,6142	**897,8205**	851,2908	982,3295	1193,238	**1008,953**	544,9122
SMAD/FHA	1113,807	1167,418	1172,671	**1151,299**	984,5212	1650,392	1104,552	**1246,489**	1090,622
SS3 strictos	35,95536	95,59077	150,5113	**94,01914**	54,41023	66,51409	37,27431	**52,73288**	83,84665
PIA1 PP2C	10987,84	7740,539	9141,071	**9289,816**	10807,08	6764,714	11443,11	**9671,633**	10965,46
neurofilam	315,0119	128,296	184,2083	**209,1721**	67,65942	245,1337	94,88681	**135,8933**	142,6229
Disease res	2467,483	1029,273	539,0942	**1345,283**	2058,609	857,8479	1627,697	**1514,718**	622,2317
TraB family	2575,435	1820,727	2033,322	**2143,161**	1132,205	1437,172	2509,697	**1693,025**	1960,53
F-box and a	348,258	10,23083	468,0353	**275,508**	237,8072	206,2664	335,6941	**259,9226**	356,5355
TCP-1/cpn6	31480,02	33866,88	42643,39	**35996,76**	46355,03	29717,64	40963,78	**39012,15**	43250,67
RPSAb 40s	1911,302	2366,96	2176,65	**2151,638**	2148,995	3547,654	1888,601	**2528,417**	1849,145
Nuclear tra	4695,467	5474,999	5965,81	**5378,759**	4278,807	5770,706	4163,524	**4737,679**	5452,22
ATPHB1, PI	262,0304	471,1314	390,1565	**374,4394**	675,8978	368,8856	418,5469	**487,7768**	229,5474
ZW9 TRAF-	41606,06	38715,53	41395,63	**40572,41**	29981,99	43672,55	36020,22	**36558,26**	37490,3
RNA 3'-terr	1336,962	1838,74	1954,321	**1710,008**	2135,365	1136,793	2503,058	**1925,072**	2274,605
Transducin	6215,634	12063,68	11694,67	**9991,327**	17223,51	6993,628	17855,04	**14024,06**	14946,63
BGAL4 beta	10486,34	13405,6	7926,813	**10606,25**	10916,9	12563,44	12391,73	**11957,36**	10706,13
GAPCP-1 gl	43613,08	53875,01	62851,25	**53446,45**	78790,72	47498,21	76424,58	**67571,17**	61044,57
Peroxidase	4146,3	7334,789	6583,682	**6021,59**	5084,372	13135,04	8546,609	**8922,006**	4074,17
RCN1, REG/	4635,818	8073,203	6944,411	**6551,144**	4400,973	7896,308	3422,482	**5239,921**	4325,81
GTP bindin	500317,6	530055,7	570225,1	**533532,8**	428253,3	839177,1	727443,5	**664957,9**	449190,8
mtLPD1 mi	9992,005	11158,21	13506,18	**11552,13**	12638,05	11120,13	17684,98	**13814,39**	13577,4
HSP60-2 he	60095,3	62050,11	59629,53	**60591,65**	62903,81	55622,88	54158,51	**57561,73**	60060,4
ATPDIL1-3,	31397,39	36961,39	40295,06	**36217,95**	41297,69	34113,52	52961,14	**42790,79**	37025,33
FER Malect	4438,267	2115,715	6247,921	**4267,301**	1675,789	5620,073	1919,342	**3071,735**	2332,556
NMT1, XPL	7502,805	2733,753	2785,306	**4340,621**	2347,681	6277,265	3303,363	**3976,103**	2041,718

CRK11, AT-	171,7221	1285,508	398,0079	**618,4128**	2037,66	400,1616	1521,817	**1319,88**	473,257
ROC7 rotar	9313,85	8518,98	8917,799	**8916,876**	5315,605	8726,964	7887,267	**7309,945**	6264,502
ATGSTU13,	23555,12	29534,98	32257,24	**28449,11**	28366,32	21859,17	23970,93	**24732,14**	26979,74
ATMS2, MS	101578,3	65447,42	70992,14	**79339,27**	91363,93	87483,96	94764,24	**91204,04**	81073,59
GAL1, GALK	12863,82	12018,14	9852,26	**11578,07**	12908,69	9331,885	10526,86	**10922,48**	9181,149
ubiquitin-a	1252,474	2718,563	3324,089	**2431,709**	3263,296	1567,72	4048,788	**2959,935**	3597,585
Zinc-bindin	18859,03	20755,36	19548,92	**19721,1**	18606,03	40690,45	20773,86	**26690,11**	24703,62
GH3.17 Aux	974,3599	2475,989	2175,685	**1875,344**	2508,296	2364,549	1965,521	**2279,456**	1778,289
Subtilase fa	34309,18	37037,61	37132,96	**36159,92**	47587,58	28616,03	58196,46	**44800,02**	46836,34
PAB2, PABI	8390,945	6765,067	6870,497	**7342,17**	7610,585	7806,815	9200,263	**8205,888**	8027,923
FIB1, FBR1,	1674,028	3758,271	4016,788	**3149,695**	4052,644	3048,982	5391,13	**4164,252**	3097,578
ATTCP-1, T	25089,37	25102,13	28506,73	**26232,74**	31024,71	23947,88	218591,1	**91187,9**	23603,77
NOP56 hor	4947,601	6486,662	7363,667	**6265,976**	9587,892	5953,812	8787,287	**8109,664**	8641,799
Auxin-resp	2341,227	1919,541	2429,609	**2230,126**	1754,542	2299,566	3676,16	**2576,756**	1820,91
EMB1467,	46915,51	50955,47	53833,31	**50568,1**	62256,89	45110,09	71516,24	**59627,74**	58934,96
2-oxoglutar	20877,69	28651,23	23005,74	**24178,22**	30496,71	18686,09	23785,5	**24322,77**	21175,24
tolB protei	2119,442	2660,955	1160,009	**1980,136**	1190,801	2662,985	1400,464	**1751,417**	1202,689
Alkaline-ph	4822,362	16194,94	17454,34	**12823,88**	19931,53	5505,131	6071,186	**10502,62**	16117,15
glycosyl hy	338,8895	362,0606	386,4473	**362,4658**	809,7822	24,34206	320,8931	**385,0058**	473,2621
ATRFNR2, I	3003,519	3068,328	3955,508	**3342,451**	1848,365	4119,254	2208,978	**2725,533**	2059,801
Bifunctiona	6346,931	8109,979	8120,326	**7525,745**	7571,47	6877,856	7275,921	**7241,749**	6854,451
CIPK9, PKS	469,3535	69,65016	28,34383	**189,1158**	385,6563	18,61121	358,7057	**254,3244**	707,6169
XYL4, ATBX	614,0058	549,4215	282,5596	**481,9956**	104,0133	769,2251	122,2645	**331,8343**	222,4474
VEP1, AWI3	10760,3	11159,68	11302,3	**11074,09**	12448,09	5961,604	10557,56	**9655,75**	12558,03
uridine 5'-r	26406,55	41595,56	36025,04	**34675,71**	47591,7	31811,45	47059,48	**42154,21**	39593,37
Phospholip	35507,4	34070,24	27128,51	**32235,38**	19001,51	52433,09	18659,98	**30031,53**	21318,77
Pectinacety	11247,05	26813,48	33585,01	**23881,85**	31604,13	10946,79	13871,51	**18807,48**	31605,83
HIT3, HINT	1531,634	862,3581	887,8791	**1093,957**	456,0315	1813,236	407,5712	**892,2795**	1082,344
ADL1, ADL1	3945,112	4784,332	4616,743	**4448,729**	5286,924	3048,343	5648,746	**4661,337**	3498,172
DRT102 DN	1276,281	750,3942	1334,53	**1120,402**	714,9487	974,6707	789,5785	**826,3993**	1091,399
CRK21 cyst	5411,337	8193,537	6474,802	**6693,225**	7648,7	7077,096	8781,742	**7835,846**	6669,557
Pentatricop	4467,284	5336,962	5095,427	**4966,558**	6109,329	3076,459	5741,349	**4975,712**	5494,21
Gag-Pol-rel	5860,43	7885,798	9467,127	**7737,785**	10901,27	5993,357	9224,141	**8706,255**	8877,793
ATRABA4B,	104,3962	69,65901	93,74596	**89,26704**	45,46515	97,4153	118,4108	**87,09707**	100,1908
PCK2, PEPC	3510,842	3382,688	2733,88	**3209,136**	2689,935	3798,632	2102,313	**2863,626**	3254,642
RmlC-like c	3477,4	4014,289	1375,647	**2955,779**	4310,257	2635,695	5909,003	**4284,985**	4039,039
Aldolase su	68984,34	63940,91	61482,71	**64802,65**	43336,93	90683,37	47487,61	**60502,64**	47766,96
ATJ2, J2 DN	22,97012	358,0974	200,3563	**193,8079**	111,4093	106,7972	18,15312	**78,78655**	193,8526
MEE5 Ribo	1344,076	2689,993	3084,916	**2372,995**	5624,658	1765,314	4088,572	**3826,181**	4397,657
CRB, CSP41	640,8035	640,1896	700,7245	**660,5725**	336,4154	876,2877	469,6695	**560,7909**	674,8782
GAMMA CA	1553,693	221,2497	307,9621	**694,3015**	1415,607	310,0163	1036,868	**920,8305**	1840,018
phosphorib	809,2259	1083,777	1311,47	**1068,158**	1472,7	692,5525	1263,088	**1142,78**	1314,953
HMGB2, HI	162,5481	859,1858	1169,703	**730,479**	413,9441	336,9012	175,391	**308,7454**	209,251
Mannose-b	117583,4	112704,2	128301,9	**119529,8**	94674,67	105169,9	122908,9	**107584,5**	115818,2
PLDALPHA2	14559,04	19457,57	17816,48	**17277,69**	17928,86	20184,36	15872	**17995,07**	16989,39
NIT1, ATNI	3664,094	4400,781	5196,415	**4420,43**	5625,845	3001,402	4483,309	**4370,185**	5072,125
MAT3 meth	117559,5	127408,8	131193,7	**125387,3**	105574,9	117580,5	137711,8	**120289,1**	98685,81
catalytics c	14951,31	18104,41	17036,53	**16697,42**	19594,06	15958,74	17637,96	**17730,25**	18540,83
PYRD pyrim	621,5894	1595,241	449,5909	**888,807**	3231,604	641,6868	4174,487	**2682,593**	2823,973
2-oxoglutar	821,9717	433,4373	578,7879	**611,3989**	609,1739	305,9011	819,0244	**578,0331**	822,7543

ACT4 actin	112,6739	153,8761	141,7242	**136,0914**	94,04455	219,0717	48,16905	**120,4284**	34,11749
PAL3 pheny	6167,181	5807,645	7105,829	**6360,218**	6398,709	6561,212	5433,104	**6131,009**	5878,826
ALDH2B4, /	43534,65	52298,84	53496,74	**49776,74**	52764,72	41870,63	38157,87	**44264,41**	48268,37
EMB3010,	40,18531	34,85406	28,44917	**34,49618**	135,0062	14,19185	478,7478	**209,3153**	70,59989
ATRPS5A, /	79126,12	65834,76	74559,96	**73173,62**	68158,13	89034,46	93791,48	**83661,36**	75504,95
ATRABG3C	4478,199	5179,662	5185,462	**4947,774**	3490,583	5867,543	3625,34	**4327,822**	4442,789
Ribosomal	2923,848	3355,124	3611,705	**3296,892**	2597,088	3539,923	2739,003	**2958,671**	2947,285
Pyruvate ki	1650,75	2537,209	2034,951	**2074,303**	3279,438	1444,94	3025,074	**2583,151**	1699,345
PDR12, ATI	804,99	856,3711	754,5	**805,287**	1071,633	700,7211	923,8936	**898,7491**	953,3102
Tetratricop	251,0477	336,0126	188,2341	**258,4315**	424,8076	201,2188	582,7596	**402,9287**	342,8711
GroES-like	42724,18	61440,75	61563,87	**55242,93**	55260,27	42039,58	44701,62	**47333,83**	52348,68
Eukaryotic	3746,1	4563,889	4205,784	**4171,924**	3037,764	8063,047	5572,524	**5557,779**	3998,967
ACO2 acon	83668	94004,7	104013,7	**93895,48**	116611,6	76842,56	105215	**99556,39**	83524,96
Ubiquitin C	979,7017	950,8982	1141,769	**1024,123**	1321,499	968,005	945,7525	**1078,419**	1056,809
C2H2-like z	2072,55	885,9838	933,6128	**1297,382**	1123,279	3508,495	1590,92	**2074,231**	1515,76
INVOLVED	1035,456	967,7098	1015,69	**1006,285**	1288,324	882,2532	1435,112	**1201,896**	1242,573
ATRABA2D	1428,125	400,9507	337,2222	**722,0993**	226,5233	340,8419	385,1067	**317,4906**	15,21212
ASP2, AAT2	51328,56	59078,02	53957,61	**54788,06**	66324,71	52556,07	76853,49	**65244,76**	70385,02
Protein kin	65182,21	57684,45	72584,28	**65150,31**	76309,21	49312,69	63529,34	**63050,42**	67834,51
Aldolase su	302810,6	347865,8	376868,4	**342514,9**	435162,5	274201	496382,6	**401915,4**	391232,4
TUA6 Tubu	2026,784	4135,591	3509,514	**3223,963**	3718,12	3666,365	3401,385	**3595,29**	3979,406
ATNTT2 TL	2636,439	2623,553	2034,059	**2431,35**	3904,921	2611,306	2103,663	**2873,297**	1751,586
RPS15 cyto	1813,989	1603,685	1790,774	**1736,149**	1580,817	5115,21	3707,781	**3467,936**	1019,259
PKP1, PKP-	12461,68	19411,04	18904,12	**16925,62**	22968,84	15177,75	22361,16	**20169,25**	18774,95
Ribosomal	1164,299	735,6473	814,9251	**904,9571**	618,563	1365,773	1053,378	**1012,571**	479,4439
ATGDI2, GI	44828,22	60697,29	52279,39	**52601,64**	74851,17	46006,32	71481,97	**64113,15**	58605,8
unknown p	245,8433	1105,131	922,8254	**757,9332**	1492,152	169,0269	337,1036	**666,0942**	921,4785
Nitrilase/cy	2928,268	6778,137	4489,085	**4731,83**	3427,507	4127,78	3573,889	**3709,726**	3222,185
Ribosomal	2802,339	2405,368	2654,069	**2620,592**	2051,528	6418,047	5188,439	**4552,671**	2274,801
vacuolar A	24204,18	25401,8	25509,41	**25038,46**	27482,2	25178,1	25607,86	**26089,39**	31271,85
HSP81-2, E	17335,47	13646,49	16922	**15967,99**	16435,81	9732,059	14822,52	**13663,46**	14071,21
ROC1 rotar	12318,94	14531,47	12019,66	**12956,69**	8223,773	27227,67	10950,74	**15467,39**	15829,95
Adenine nu	11261,05	12539,04	14381,2	**12727,1**	14658,13	11386,79	15712,97	**13919,3**	13113,76
alpha-ADR	614,7546	547,8989	456,7749	**539,8095**	1143,983	458,8124	1142,076	**914,9573**	735,9564
Ankyrin-rep	14309,36	13661,59	10887,02	**12952,66**	7201,923	14747,3	9190,186	**10379,8**	7448,361
GHBDH, AT	16598,51	19733,86	20741,85	**19024,74**	30073,19	15435,94	22249,89	**22586,34**	23140,9
RUB1, NED	280,1068	96,91419	242,0796	**206,3669**	61,85945	181,9357	131,1422	**124,9791**	105,5842
Glycosyl hy	7206,622	5272,365	5782,209	**6087,065**	5597,701	5502,8	5376,082	**5492,194**	6418,499
ADT4 aroge	301,9488	186,0529	215,8362	**234,6126**	176,0511	139,6711	232,6832	**182,8018**	184,6368
Ribosomal	113,6279	13,44504	39,20935	**55,42744**	80,60409	194,1737	211,5107	**162,0962**	7,469737
mtLPD2 lip	22314,3	27454,28	27388,48	**25719,02**	33312,24	22725,61	33350,31	**29796,05**	26136,13
pfkB-like ca	1624,527	1545,952	1408,899	**1526,46**	1153,227	2569,272	1368,641	**1697,047**	1286,426
OXP1 oxop	3641,7	4718	3146,999	**3835,566**	4540,473	4117,487	4200,188	**4286,049**	4413,558
ARM repea	1861,585	7561,215	9029,414	**6150,738**	9439,727	986,9309	3639,066	**4688,575**	7183,614
APX1, MEE	281513,2	270857,1	278839,7	**277070**	206827,6	275402,9	271495,7	**251242,1**	176211,5
KAB1, KV-B	4187,256	5482,815	5861,502	**5177,191**	5343,318	3910,164	5467,54	**4907,007**	865,9296
ATP syntha	689565,2	798152,5	784171	**757296,3**	1016424	626914,2	1021626	**888321,6**	958516,5
ATHXK1, GI	15418,52	12995,34	15674,9	**14696,25**	18230,72	13249,33	21514,06	**17664,7**	17798,06
F-box famil	63,48866	528,8415	319,7456	**304,0252**	1961,226	12,84866	21,63023	**665,235**	214,8483
Ribosomal	2116,96	2185,87	1710,968	**2004,599**	2076,444	3249,908	2024,92	**2450,424**	2394,652

ROC2 rotar	3483,02	3878,39	4418,393	**3926,601**	2979,489	3896,631	3458,793	**3444,971**	3385,462
LACS9 long	1407,442	1213,46	988,9635	**1203,288**	1221,499	941,2356	1997,259	**1386,665**	1075,709
ACO3 acon	116882,9	147283	144307,9	**136157,9**	160283,1	130479,5	161070,2	**150610,9**	151876,5
Actin-like A	24805,35	40584,02	40164,45	**35184,61**	47527,8	29346,38	52347,68	**43073,95**	36648,55
ACT7 actin	68712,96	76933,74	73357,91	**73001,54**	109515,4	55929,8	111194,2	**92213,13**	70726,31
Peroxidase	8280,626	13971,47	15562,01	**12604,7**	13785,08	9587,476	7256,579	**10209,71**	10458,62
Peptidase I	19827,5	24116,76	22286,34	**22076,87**	27144,59	24020,2	20835,58	**24000,12**	23892,75
Cyclophilin	4526,479	4955,128	5456,352	**4979,32**	6360,648	4388,276	5814,691	**5521,205**	4818,788
PLP1, PLA I	13400,7	17045,96	14127,99	**14858,21**	22040,91	11931,49	24241,53	**19404,64**	17382,1
ASD1, ARAI	23555,51	28145,49	24702,83	**25467,95**	20628,39	23496,57	30608,07	**24911,01**	23272,55
SHM2 serir	1210,592	1452,797	1226,309	**1296,566**	800,3757	1983,342	1409,999	**1397,905**	1478,567
AHA1, PMA	2923,635	1780,833	2193,6	**2299,356**	1514,604	2191,214	2009,969	**1905,262**	2300,081
ATHOL1, H	42763,06	62737,03	56097,58	**53865,89**	55033,41	40146,84	43876,46	**46352,24**	39363,81
TCP-1/cpn6	45052,99	111495	120595,9	**92381,3**	107768,8	42919,68	56405,31	**69031,27**	92646,56
ATGSTF8, A	1972,236	1354,471	1646,183	**1657,63**	1230,97	7232,742	2358,39	**3607,367**	1400,187
Hsp81.4, A	7082,079	8563,395	10539,2	**8728,225**	10746,13	5225,627	5525,915	**7165,892**	6769,977
EMB2719,	669,3628	939,5278	1246,257	**951,7157**	574,4861	733,1227	724,1508	**677,2532**	594,8782
ATRAB18, A	3643,139	4711,875	3558,936	**3971,317**	2026,578	8353,336	1766,746	**4048,887**	2127,219
POLGAMM	1917,701	1115,921	2579,312	**1870,978**	1487,117	956,327	1372,213	**1271,886**	1530,647
VDAC1, AT	144476,3	169758	164713,3	**159649,2**	179962,7	140791,6	176406,7	**165720,3**	181912
cICDH cyto	274185,4	349516,8	308764,8	**310822,3**	370453,8	262416,3	416439,8	**349770**	367942
ANNAT1, O	49759,09	37949,19	31707,22	**39805,16**	26300,65	85064,46	44268,19	**51877,77**	22025,87
FIP2 potass	273,0015	7773,735	6254,893	**4767,21**	11624,57	612,7035	257,5723	**4164,947**	5331,841
ATB5-B, B5	5200,712	2962,015	3237,986	**3800,238**	2661,276	4891,948	4298,779	**3950,667**	2784,686
Aluminium	917,9525	2181,258	2575,966	**1891,726**	2430,576	1491,674	2724,608	**2215,619**	2455,002
oxidoreduc	6486,93	15851,4	8592,396	**10310,24**	11040,05	15348,9	11441,93	**12610,29**	10346,24
UBQ13 ubi	8971,682	5157,658	4726,113	**6285,151**	6197,938	4693,619	3750,354	**4880,637**	5318,172
AMC6, ATM	175,9934	2032,074	593,5673	**933,8783**	1903,958	248,18	144,7153	**765,6179**	667,6447
HA3 H(+)-A	7284,684	9231,42	10759,37	**9091,825**	8022,379	7481,97	8567,537	**8023,962**	8502,154
Ribosomal	26761,62	32063,49	32010,16	**30278,42**	28627,1	31597,43	37240,48	**32488,34**	27604,43
TRAF-like fa	3298,166	3820,451	4059,333	**3725,983**	3470,048	3399,389	3575,713	**3481,717**	4131,624
SRZ-22, SRZ	1728,088	2141,506	1747,707	**1872,434**	1369,208	2382,683	1277,581	**1676,491**	1619,871
BRIZ1 zinc f	658,1497	100,453	93,145	**283,9159**	63,89867	723,6898	789,0786	**525,5557**	148,6526
F-box and a	16863,28	13627,09	16189,36	**15559,91**	14133,26	17321,99	18081,75	**16512,34**	15146,96
Thioredoxi	23353,82	27207,37	35346,29	**28635,83**	27187,41	14652,75	28577,04	**23472,4**	24875,04
Cystathioni	6377,502	4086,63	3599,856	**4687,996**	4250,509	8011,098	5393,466	**5885,024**	5031,722
CYSD2, ATC	3223,47	4464,162	3659,814	**3782,482**	3691,466	3591,626	3233,406	**3505,499**	3889,069
LTA2, PLE2	24824,22	28044,72	29578,5	**27482,48**	30674,96	24335,83	32183,16	**29064,65**	33069,64
Sec14p-like	9271,94	10209,61	9329,942	**9603,829**	9686,26	5510,779	9666,749	**8287,929**	8456,906
5'-3' exonu	283,9589	322,7019	419,8387	**342,1665**	286,3523	354,9815	316,6879	**319,3406**	317,4893
Eukaryotic	70118,72	65143,97	63802,53	**66355,08**	53953,72	103385,8	65031,53	**74123,69**	58027,87
THFS 10-fo	38691,22	36615,82	34260,33	**36522,46**	32638,12	36704,02	34043,31	**34461,82**	37157,31
ATNAP9, N	26720,77	28893,68	25519,63	**27044,69**	21512,76	34146,13	29641,24	**28433,38**	21842,93
MD-2-relat	37047,7	39984,47	42262,25	**39764,81**	40159,28	29901,01	40763,92	**36941,4**	37963,2
Methionine	2091,189	1820,627	1145,356	**1685,724**	1511,997	995,1976	1541,689	**1349,628**	1402,637
AOC4 allen	3977,332	6282,163	4368,893	**4876,129**	4545,769	7135,967	4036,548	**5239,428**	5609,786
TIP2;2, DEL	19674,56	29745,21	28785,32	**26068,36**	29368,1	27575,52	33566,08	**30169,9**	30589,72
Glyceraldel	2578,863	1945,634	2506,333	**2343,61**	2091,215	2289,954	3526,793	**2635,987**	2928,787
D-isomer sp	178,3013	381,3665	343,2093	**300,9591**	443,4104	234,7172	548,8954	**409,0077**	294,5602
GGT3, GGT	1240,798	994,8508	733,1165	**989,5885**	589,5542	976,3169	1010,4	**858,7571**	1347,637

ATCTIMC, T	345719,2	353093,1	376164,8	**358325,7**	528285	295533,7	489660,6	**437826,5**	426668,5
pfkB-like ca	4271,641	2606,991	2948,351	**3275,661**	2298,425	3611,85	3362,918	**3091,064**	2646,969
GAMMA-TI	25301,11	35320,53	43650,6	**34757,41**	43676,65	31561,69	48625,35	**41287,9**	47287,83
FLA8, AGP8	32495,26	45650,94	43280,75	**40475,65**	53763,24	28308,4	59873,72	**47315,12**	53070,58
FQR1 flavo	64761,6	52384,43	45704,06	**54283,36**	44102	74228,11	43133,21	**53821,11**	61254,9
IMPA-4 imp	5850,789	5878,364	4833,799	**5520,984**	4704,832	8162,73	5634,893	**6167,485**	4786,843
F-box/RNI-l	1619,469	48,9131	39,32888	**569,2371**	25,13577	2136,187	140,2332	**767,1853**	84,75532
MA3 doma	765,582	1046,53	1062,063	**958,0582**	1286,865	596,8738	1246,799	**1043,513**	1281,745
ATP-depen	8291,211	1441,701	394,0882	**3375,667**	6719,087	1093,048	4244,951	**4019,029**	7775,604
PAB8, PABI	3701,795	1964,508	2044,792	**2570,365**	2750,732	1609,159	3225,078	**2528,323**	2835,166
ATEXPA9, E	98,55013	178,2786	167,1076	**147,9788**	202,9638	110,059	51,47919	**121,5007**	112,9304
GLT1 NADH	1949,892	3012,326	2884,955	**2615,724**	3703,865	2482,764	3002,719	**3063,116**	2650,248
D-aminoaci	46,08104	66,31213	70,27323	**60,8888**	11,8963	34,41667	122,4231	**56,24536**	18,08848
VTC4 Inosit	5412,957	4408,457	4073,766	**4631,727**	3430,107	6509,978	4725,566	**4888,551**	4577,685
Eukaryotic	753,2083	1186,176	1509,305	**1149,563**	1534,567	755,234	1403,131	**1230,977**	1571,708
Late embry	578,0108	5988,257	2874,933	**3147,067**	1454,956	6918,897	1519,148	**3297,667**	719,3944
PFN1, PRF1	42086,38	57134,05	58174,8	**52465,08**	58929,19	30361,37	44291,21	**44527,26**	46973,68
EMB3004,	14301,77	13879,66	12597,21	**13592,88**	10682,64	17841,47	9168,148	**12564,09**	11256,4
PKT3, PED1	82929,73	81761,48	90944,04	**85211,75**	86148,53	62225,57	86772,15	**78382,09**	85631,62
uricase / ur	330,7567	1288,754	1277,091	**965,5337**	1771,576	328,2221	1525,92	**1208,573**	1408,211
Transketola	9952,924	9129,528	11099,3	**10060,58**	10857,37	8345,718	9435,811	**9546,299**	9858,559
ATPERX34,	14732,15	11431,55	10962,47	**12375,39**	7055,717	18405,58	7469,671	**10976,99**	10579,19
CCH coppe	382,2414	1026,248	1037,739	**815,4094**	751,7459	2694,436	873,3802	**1439,854**	821,6128
UDP-glucos	10623,39	11897,25	11518,54	**11346,39**	18324,3	8447,123	15802,54	**14191,32**	14395,87
MTO2, TS F	19172,7	19478,79	20954,75	**19868,75**	22456,01	15526,5	23915,62	**20632,71**	22829,49
clathrin ada	21643,73	31669,01	31030,89	**28114,54**	29619,53	29388,6	35392,07	**31466,73**	28595,06
GRH1, ATG	1448,693	1215,528	1174,927	**1279,716**	2096,803	892,4414	1531,046	**1506,763**	1531,349
unknown p	16081,95	12585,47	12743,96	**13803,79**	15571,43	12963,53	25525,93	**18020,3**	17297,96
Cytidine/de	1581,108	1394,677	1501,876	**1492,554**	2934,334	1141,962	2072,83	**2049,708**	1447,968
ENOC cytos	3048,858	3272,196	3748,444	**3356,499**	3723,857	2519,781	3273,824	**3172,487**	3145,362
ACLA-2 ATF	1452,119	1216,283	1473,446	**1380,616**	1094,638	1676,932	1417,396	**1396,322**	768,093
ATGSTU19,	312,5925	190,5902	206,9851	**236,7226**	20,50534	379,8629	119,5204	**173,2962**	33,05392
ALDH10A9	13993,02	15714,62	11888,31	**13865,32**	15125,98	12464,71	16007	**14532,56**	14129,56
TUF, emb24	2717,571	2012,567	2298,773	**2342,97**	1343,682	3683,859	1209,83	**2079,124**	2194,734
COS1 6,7-d	3084,786	3883,379	2674,607	**3214,257**	4058,14	2411,231	3378,062	**3282,478**	4324,893
AtCXE5, CX	5254,77	6051,603	5154,137	**5486,837**	6088,301	5629,202	5037,541	**5585,015**	5326,415
CDPK6, CPK	13489,78	11503,13	12912,07	**12635**	13029,13	12363,73	14758,97	**13383,95**	12854,15
CAT3, SEN2	23399,26	20737,67	22931,3	**22356,08**	18241,3	27291,16	28179,8	**24570,75**	20520,91
SMT1, CPH	2390,746	2215,205	2469,576	**2358,509**	2505,529	2692,619	3725,684	**2974,61**	1541,588
unknown p	3754,766	4685,725	5672,929	**4704,474**	4190,892	4427,273	4323,775	**4313,98**	3783,977
Pleckstrin h	2474,788	4250,298	2728,861	**3151,316**	2438,711	4047,779	1929,713	**2805,401**	2415,717
TET8 tetras	1061,317	588,34	671,9191	**773,8588**	1006,027	690,7972	1084,25	**927,0247**	803,9513
ATPase, F1	7289,046	6252,199	5295,409	**6278,884**	14905,73	3581,613	12773,31	**10420,22**	12087,96
ADK2 aden	35833,21	33473,82	37660	**35655,68**	43954,68	26122,88	41499,8	**37192,46**	34955,8
RmlC-like c	18099,28	18433,64	19462,58	**18665,16**	17043,49	15034,12	22602,74	**18226,78**	20878,3
LACS4 AMF	18340,59	28643,9	27334,57	**24773,02**	29063,75	23541,21	31860,09	**28155,02**	25417,71
VHA-A vacu	505388,9	570977,9	597227,3	**557864,7**	651356	486233,6	713413,5	**617001**	582447,8
ADSS aden	22890,54	29656,26	25547,57	**26031,46**	35566,28	22588,09	31741,94	**29965,44**	27962,31
LOCATED II	680,2972	859,0186	959,4068	**832,9075**	1018,782	782,2343	1022,833	**941,2832**	879,4327
APS4 Pseud	3934,072	2037,468	1276,142	**2415,894**	2007,997	2975,944	4366,929	**3116,957**	2225,197

AMT1;3, AT	991,7337	2343,571	1213,806	**1516,37**	1961,817	1209,741	2837,543	**2003,034**	1193,885
GGT1, AOA	1255,407	4761,493	3589,571	**3202,157**	5417,982	2635,9	4598,787	**4217,556**	3014,988
NIT3, AtNIT	4096,658	3808,208	4227,322	**4044,063**	3113,134	3375,999	4633,749	**3707,627**	3897,602
Peroxidase	4215,997	3381,531	4072,435	**3889,988**	2715,662	6453,452	3813,751	**4327,622**	3143,86
PAF1, ATPS	10757,33	11219,5	12697,22	**11558,01**	9972,648	13954,64	7785,983	**10571,09**	9320,697
GDH1 gluta	17910,43	29591,76	25908,48	**24470,23**	15225,03	39143,84	16177,79	**23515,55**	16755,25
JAL23 jacal	6264,707	4241,589	5197,178	**5234,491**	4172,283	5946,915	5747,925	**5289,041**	4908,423
ADK1, ATAI	92363,17	97879,68	98805,23	**96349,36**	97851,9	70842,41	98407,54	**89033,95**	94769,39
ATPase, V1	58968,13	65885,14	66710,54	**63854,61**	68716,47	56579	70230,27	**65175,25**	74620,49
NDPK3 nuc	26166,09	18338,73	19721,12	**21408,65**	10434,65	25955,03	19188,24	**18525,98**	19829,59
Protein of u	1467,738	68,27351	44,81756	**526,9429**	690,1388	27,61077	845,6531	**521,1342**	1243,435
ATMDAR2	132306,3	154429,8	145597,9	**144111,3**	162609,1	122500,2	161460,4	**148856,6**	141116,2
PAC1 20S p	9063,06	6044,448	5291,1	**6799,536**	4701,315	6677,406	6471,278	**5950**	6020,17
Adenine nu	21586,8	10252,68	10144,98	**13994,82**	5747,388	21897,53	11431,83	**13025,58**	8104,07
GAD5 gluta	4596,025	674,0884	593,8723	**1954,662**	2401,41	635,1392	2699,389	**1911,98**	3884,611
AlaAT1 alar	27759,16	23776,12	30942,03	**27492,44**	29962,23	23226,29	32419,62	**28536,05**	25448,32
MAB1 Tran	50315,49	71341,33	66899,59	**62852,14**	79414,69	55484,29	70075,41	**68324,8**	65865,84
BGLU35, TC	36456,2	26161,84	31788,86	**31468,96**	24080,03	39874,42	45022,92	**36325,79**	27864,11
DEGP1, Deg	3043,711	4946,046	5756,193	**4581,983**	4855,256	4667,597	6318,834	**5280,562**	4716,77
ATOEP16-3	2338,053	2019,162	2296,438	**2217,884**	1915,983	3745,056	2185,776	**2615,605**	2130,696
Tubulin bin	4653,852	4611,314	3173,811	**4146,326**	4941,213	3244,588	6228,34	**4804,713**	4494,838
IMPA-2 imp	13618,3	14600,09	14245,03	**14154,47**	15757,48	10170,65	15560,74	**13829,62**	16056,89
Protein kin	1924,605	764,0798	645,0768	**1111,254**	502,8906	1528,866	960,1236	**997,2934**	528,3877
B73, SIR4, (	706,3455	786,2414	874,8189	**789,1353**	863,0891	649,6109	1301,639	**938,1131**	784,8738
LTA3 Dihyd	4454,513	4997,638	5647,637	**5033,263**	5099,65	4561,727	6414,12	**5358,499**	5439,081
GAMMA CA	30133,39	33679,13	34435,02	**32749,18**	31029,72	30077,69	35711,54	**32272,98**	27602,18
Zinc-bindin	844,3269	585,8636	669,947	**700,0458**	213,5493	1776,043	191,8868	**727,1596**	365,4494
Galactose r	506,0524	507,5575	367,429	**460,3463**	610,6716	355,9247	123,0961	**363,2308**	562,8444
LysoPL2 lys	5058,985	9887,611	9602,141	**8182,912**	9223,012	9161,475	8567,099	**8983,862**	8572,799
Class I gluta	1237,757	2178,562	2739,58	**2051,966**	2834,833	1547,886	3155,784	**2512,834**	2211,663
ATCOQ3, E	1960,514	2659,228	2705,036	**2441,592**	2186,568	2330,663	2151,794	**2223,008**	2567,395
ATPUMP1,	6105,616	5723,923	5960,425	**5929,988**	5127,436	7101,77	6125,231	**6118,146**	5654,317
FUM2 FUM	2318,151	3342,287	3910,229	**3190,222**	3423,092	3139,754	4138,201	**3567,016**	2681,28
RING/U-bo	188,2847	275,3452	137,0213	**200,2171**	230,3864	292,7231	7,713572	**176,941**	79,22501
Ribosomal	4828,034	6751,213	8045,43	**6541,559**	9136,362	5427,372	8260,434	**7608,056**	8919,964
AAC3, ATAA	9989,351	10585,86	11288,88	**10621,37**	9671,098	11308,37	10625,81	**10535,1**	10728,31
AOC3 allen	9188,503	9257,921	9617,294	**9354,572**	9242,675	10817,78	9594,232	**9884,896**	9380,247
ATCIMS, AT	925919,2	794144,2	795473,6	**838512,4**	1011996	704696,1	1144960	**953883,9**	874189,5
GLU2 gluta	27402,81	26415,81	32055,69	**28624,77**	31772,04	22768,84	28799,73	**27780,2**	31848,8
Hsp70b hea	2199,254	677,4573	747,7294	**1208,147**	934,2413	1618,353	1746,056	**1432,883**	1661,343
ATCS, CSY4	9044,02	10279,65	11279,38	**10201,02**	7374,243	10999,71	7822,999	**8732,317**	7456,952
VLN4, ATVL	3314,732	4340,946	4618,519	**4091,399**	3711,276	3935,189	4302,376	**3982,947**	4289,4
Lactoylglut	6764,063	5936,309	6877,043	**6525,805**	7941,862	5306,148	7168,373	**6805,461**	6323,345
ATPLDDELT	5148,022	4325,027	2946,359	**4139,803**	4418,513	3728,097	4921,085	**4355,898**	4285,05
G6PD2 gluc	4264,463	3496,945	3774,725	**3845,378**	3914,795	3157,416	3652,02	**3574,744**	3659,529
RD21, RD21	66388,76	62091,53	73311	**67263,76**	73644,59	53859,22	89933,59	**72479,13**	84603,54
ATAPP1, AP	4323,507	5990,516	6367,287	**5560,437**	6200,236	3922,453	4403,726	**4842,139**	5601,087
ACLB-1 ATP	3782,756	5303,999	4839,085	**4641,947**	4999,835	4765,603	5163,172	**4976,203**	4527,016
APS1 ATP s	7149,053	4965,074	4583,297	**5565,808**	3892,721	6705,678	3697,418	**4765,272**	4503,464
PMDH1 per	3148,894	3778,526	3184,139	**3370,52**	2560,253	2889,393	3784,003	**3077,883**	2875,383

Protein of \	3812,095	890,2014	560,7493	**1754,349**	714,0995	2506,353	1099,511	**1439,988**	713,9443
Nuclear tra	2330,754	1752,678	1830,234	**1971,222**	1374,68	2038,07	1833,252	**1748,667**	1561,53
VDAC3, AT\	135510,1	149506,1	151425,1	**145480,4**	177616,9	122573,3	171561	**157250,4**	165739,8
ATSFGH, SF	4705,338	5674,816	6926,562	**5768,905**	5231,773	4791,656	5657,813	**5227,081**	5455,781
NQR ARP p	1939,422	2136,494	2687,73	**2254,549**	3323,854	1642,091	2599,159	**2521,701**	2104,36
ANNAT2 ar	4103,448	3850,589	3212,982	**3722,34**	2436,832	6916,361	4741,64	**4698,278**	3906,806
EDA9 D-3-p	115949,1	127182,4	127517,4	**123549,6**	190422,9	96137,42	156770,7	**147777**	150488,6
Malectin/re	2680,391	4147,442	4351,081	**3726,305**	4234,311	3218,969	6039,821	**4497,7**	3743,028
Coatomer,	1756,399	987,3117	1075,676	**1273,129**	2035,126	556,0319	1904,417	**1498,525**	2742,242
CEF clone e	3683,534	5811,339	6059,113	**5184,662**	7061,979	4833,349	5529,089	**5808,139**	5757,325
Prx37 Pero	12987,5	9055,906	8455,767	**10166,39**	7261,048	15111,44	8126,248	**10166,24**	10112,06
Serine prot	7154,672	8179,824	7541,509	**7625,335**	9089,173	6346,208	7329,791	**7588,391**	7625,287
ERD4 Early-	10121,21	9190,751	8240,576	**9184,178**	8999,152	10399,8	9916,589	**9771,848**	9508,819
ERD2, HSP7	6642,609	7404,126	5013,329	**6353,355**	5329,685	8407,241	5402,952	**6379,96**	6133,201
CAM4, ACA	2763,317	4526,579	4273,235	**3854,377**	6742,492	170,2567	11957,29	**6290,012**	5753,86
DIN10 Raffi	2796,98	3099,056	3297,055	**3064,364**	3118,13	3092,038	3658,838	**3289,668**	2965,006
GDSL-like L	187253,5	193644,8	180714,3	**187204,2**	128740,7	277726,6	151277,7	**185915**	148571,9
ATB2 NAD(	1994,051	2708,779	2272,048	**2324,959**	2153,633	2416,844	2167,947	**2246,141**	2172,515
UGT74D1 L	2941,464	5758,785	4674,737	**4458,328**	5125,219	4079,993	4247,275	**4484,162**	4608,661
S-adenosyl-	151,483	31,26481	194,9875	**125,9118**	366,2926	114,1715	107,3833	**195,9491**	64,19946
PEX11E per	2123,027	3289,518	2405,474	**2606,007**	2881,438	1764,759	2498,386	**2381,528**	3052,13
AtRABA6a,	63,34149	51,78059	118,7798	**77,9673**	49,33597	242,0209	89,04126	**126,7994**	58,95169
Calcium-de	274,6196	962,7984	1443,413	**893,6105**	1008,512	534,499	250,3352	**597,7821**	870,3248
Haloacid de	4269,189	3488,366	3419,282	**3725,612**	3273,066	3308,674	3874,169	**3485,303**	3580,778
MDHAR, AT	12563,35	12033,7	12257,38	**12284,81**	14183,17	11042,91	15667,99	**13631,36**	13546,36
URH1 uridi	61,31706	214,2448	128,1303	**134,564**	91,08206	144,7416	47,44888	**94,42417**	115,424
mMDH2 La	9778,591	10201,82	11589,28	**10523,23**	12182,54	8237,916	10414,67	**10278,37**	11983,22
UBQ9 ubiq	207,3035	739,2953	629,4721	**525,357**	430,7397	883,5838	576,4668	**630,2634**	500,209
PDX1, ATPI	4544,446	4734,606	5188,831	**4822,628**	3986,603	6633,893	5928,252	**5516,249**	4796,126
Ribosomal	70138,1	93592,7	94623,02	**86117,94**	139017	67047,4	119194,3	**108419,6**	140360,4
ALDH6B2 a	18969,04	24113,32	22607,77	**21896,71**	22004,99	19469,83	26230,84	**22568,55**	21933,34
NTRA, ATN	79978,37	102395,6	104181,1	**95518,36**	108157,5	81757,49	121945,3	**103953,4**	103821,7
HEXO1, ATI	4190,141	4552,648	4753,405	**4498,731**	4593,152	4728,128	4512,026	**4611,102**	4458,443
TIP2, SITIP,	66635,7	90079,68	106658,5	**87791,29**	107890,3	93526,76	105505	**102307,4**	114270,7
TUB4 tubul	8235,463	12528,43	12347,58	**11037,16**	14710,4	8350,407	15536,5	**12865,77**	13658,68
OASA1 O-a	127279,9	130851,7	140065	**132732,2**	156347,9	99604,54	131879,4	**129277,3**	143351,7
AGO3 ARG	416,092	4081,141	3131,821	**2543,018**	9441,273	382,6784	365,3045	**3396,419**	2157,907
FUNCTIONS	522,9499	1054,754	948,2893	**841,9978**	531,8941	689,647	513,8765	**578,4725**	579,3733
BGLU22 Gl	101227,2	115172,5	101949	**106116,2**	80333,82	123880,7	119873,9	**108029,5**	63569,79
RCA rubisc	11011,76	10443,36	11593,76	**11016,29**	10258,89	11414,29	10443,67	**10705,62**	12023,08
TUB1 tubul	2400,516	2968,451	2619,879	**2662,949**	3421,095	1792,436	4188,905	**3134,146**	2501,726
MLP329 M	1549,367	523,825	744,4982	**939,2301**	694,6854	754,6673	527,3439	**658,8989**	808,5487
NmrA-like	1371,048	620,402	873,7183	**955,0562**	1204,816	465,4171	732,7181	**800,9836**	929,545
COV1 Prote	315,6032	494,0909	704,5342	**504,7428**	549,6661	383,1004	576,4956	**503,0873**	491,0523
O-methyltr	8725,217	8570,381	9645,446	**8980,348**	9548,658	8239,302	7288,724	**8358,895**	8144,835
HIR1, ATHI	62,51035	191,1445	173,1879	**142,2809**	215,5801	101,3367	185,9364	**167,6177**	173,4241
FLA2 FASCI	78212,41	92148,12	88146,85	**86169,12**	68623,63	104386,7	94546,3	**89185,54**	72019,81
PAG1 20S p	25305,89	26815,85	28752,85	**26958,19**	30055,77	24852,87	30701,98	**28536,87**	29062,78
EMB1276,	111292,9	111567,6	119466,4	**114109**	139317,6	83120,02	128581,5	**117006,3**	104963,1
Coatomer,	40891,61	50674	48639,76	**46735,12**	54623,95	41365,1	54322,47	**50103,84**	45193,42

Protein of u	3374,547	2165,423	1986,205	**2508,725**	3024,741	2004,117	2467,786	**2498,881**	3093,586
Cytochrom	748,3309	193,2409	938,667	**626,7463**	502,99	482,6318	379,2692	**454,9637**	614,9456
Glyoxalase,	14120,66	14390,92	14158,29	**14223,29**	11065,26	17960,68	15478,4	**14834,78**	14721,28
26S protea	2387,568	4429,18	3859,312	**3558,687**	3777,786	4305,668	4214,484	**4099,313**	2753,702
CI51 51 kDa	18129,19	17866,4	14811,94	**16935,84**	13048,08	20278,89	16914,49	**16747,15**	15417,63
ARA3, ARA-	3764,245	2768,076	3357,412	**3296,578**	4406,063	1749,575	3036,652	**3064,097**	4386,839
NPR1, SAI1	4904,682	5619,255	4436,127	**4986,688**	4443,417	3809,2	7574,795	**5275,804**	5247,073
oxidoreduc	4203,529	4347,242	4392,019	**4314,263**	4985,136	2985,777	4652,108	**4207,674**	4546,244
Peroxidase	71263,6	53776,9	62757,14	**62599,21**	47581,97	86019,77	73581,72	**69061,15**	61097,77
HSP81-1, A	12114,92	11893,02	10670,97	**11559,64**	10148,95	9852,369	15841,38	**11947,57**	10154,85
CAD1, ARA	3107,799	4458,328	4938,319	**4168,148**	6400,078	2970,907	5453,983	**4941,656**	6577,454
MVK, MK n	170,021	457,3955	401,8745	**343,097**	330,1545	350,0818	128,6615	**269,6326**	205,7641
Haloacid de	33274,22	30141,84	33542,48	**32319,51**	21757,25	41134,23	29404,08	**30765,19**	28595,22
ATSERAT1;	1633,566	1076,533	1237,27	**1315,79**	1347,705	1563,871	1189,948	**1367,175**	1568,325
CAND1, AT	9236,138	7603,879	7850,786	**8230,268**	11919,77	5222,499	8746,538	**8629,601**	9080,174
BRCT doma	124,4101	14,52434	67,40038	**68,77828**	136,5182	10,87345	80,02122	**75,8043**	238,2
Leucine-ric	10050,17	2600,358	4847,11	**5832,545**	6644,706	4027,394	6904,696	**5858,932**	6557,037
ARM repea	7649,093	6541,459	5738,028	**6642,86**	4953,766	10162,98	9408,55	**8175,099**	5218,614
ROF1, ATFK	4501,772	5028,39	6238,664	**5256,275**	6036,249	3515,778	9458,86	**6336,962**	5700,199
Protein of u	47,74847	121,9164	139,1135	**102,9261**	114,6374	142,4445	165,992	**141,0247**	68,95477
MTHSC70-2	26019,29	26895,14	30776,81	**27897,08**	26092,49	26294,35	29827,57	**27404,8**	28830,47
IDH-III isoci	7987,544	10542,77	11212,54	**9914,287**	10258,78	10243,73	7465,223	**9322,578**	10221,11
ST1, ATMST	18175,39	20856,73	17127,91	**18720,01**	19371,04	17426,21	16521,29	**17772,85**	17332,72
NAI2 DNA t	223559,5	204051,5	218745,1	**215452**	210089,4	153888,4	244454,1	**202810,6**	176748,7
FAD-depen	1459,653	2935,121	1805,144	**2066,639**	2102,887	913,0763	2085,865	**1700,609**	1891,247
unknown p	4920,631	6772,221	7977,8	**6556,884**	8463,58	4720,578	6341,774	**6508,644**	8168,172
OMR1 L-O-	1383,721	1432,801	1485,229	**1433,917**	2004,888	991,5725	2363,956	**1786,806**	1640,81
sks4 SKU5 s	10225,72	10807,7	12118,18	**11050,53**	12771,15	9456,477	13557,88	**11928,5**	12217,46
ARM repea	10563,81	11104,08	12477,26	**11381,72**	13541,84	9884,063	13226,86	**12217,59**	12085,76
Sugar isom	7660,032	8451,001	7586,508	**7899,18**	13031,03	5882,032	7812,143	**8908,401**	9094,476
catalytics;t	27271,74	28716,72	32151,85	**29380,1**	35816,28	24950,28	35367,06	**32044,54**	30414,13
Phosphofru	35179,38	53124,03	51785,67	**46696,36**	67653,14	36758,87	54712,61	**53041,54**	54508,39
Calcium-bir	13511,87	18670,47	20845,03	**17675,79**	19255,8	14440,3	20605,23	**18100,44**	15361,87
ATPase, F1	537284,1	599000,3	657582,1	**597955,5**	689484,7	470258,5	689316,6	**616353,3**	674262,4
TCP-1/cpn6	75386,18	89015,41	86584	**83661,86**	86960,56	78124,64	102853,4	**89312,86**	83478,2
ATGSR1, GI	21180,08	21374,03	22715,6	**21756,57**	27270,33	17007,93	25649,08	**23309,11**	26178,51
zinc ion bin	3262,887	3622,912	3530,406	**3472,068**	2893,932	7651,84	2404,545	**4316,772**	4583,111
PIRL9 plant	1485,755	1773,211	1527,581	**1595,516**	1799,906	1316,032	2193,658	**1769,865**	1752,714
GSA1 gluta	6384,931	7417,517	5822,885	**6541,778**	7365,85	5317,791	5924,327	**6202,656**	6006,217
Aldolase-ty	1998,407	2284,866	2644,871	**2309,381**	2898,802	1870,983	2976,518	**2582,101**	2430,601
CPN60A, CI	29101,95	32321,1	28475,52	**29966,19**	37424,14	25140,17	33913,28	**32159,2**	29027,41
ATTPS7, TP	520,0714	618,0011	1173,671	**770,5813**	499,1541	645,4832	720,4501	**621,6958**	820,3543
ASK2, SKP1	18,19692	29,50282	80,80534	**42,83503**	30,50045	125,9035	17,8647	**58,08954**	12,22749
unknown p	9060,743	21306	21930,66	**17432,47**	13173,56	23586,18	9599,247	**15453**	12874,18
GMP synth	6319,184	5904,878	5391,879	**5871,98**	5960,652	4711,658	7792,463	**6154,924**	6154,484
Phosphorib	28314,92	34483,95	31109,61	**31302,83**	34755,87	23436,66	30181,53	**29458,02**	30143,67
acyl-CoA ox	125,188	251,9553	294,9276	**224,0236**	377,2266	80,84524	234,7424	**230,9381**	325,9507
IBR1, SDRA	6185,593	8985,583	9840,242	**8337,139**	9321,615	8290,009	8666,472	**8759,365**	7996,473
Clathrin ligl	3280,469	5230,299	3947,073	**4152,614**	4970,99	3396,02	5432,669	**4599,893**	4239,073
ATP-depen	17902,2	480,3516	337,7867	**6240,114**	473,762	3532,642	2982,235	**2329,546**	208,9806

ATRAB7A, /	3413,074	4663,449	4366,61	**4147,711**	3472,189	5420,361	2937,7	**3943,417**	4294,413
HOT5, ADH	46552,3	51940,91	51536,63	**50009,95**	51752,15	40785,33	60372,9	**50970,13**	50610,79
Ribosomal	13968,44	15111,38	17179,55	**15419,79**	12518,68	19456,07	15095,83	**15690,19**	16884,43
ATP5 delta	4478,136	3308,938	4208,849	**3998,641**	2560,52	6532,155	2019,776	**3704,15**	3352,296
DHAR1, AT	20246,05	9790,343	11324,46	**13786,95**	4492,39	35978,34	5506,639	**15325,79**	6472,582
Amino acid	2119,134	184,1106	276,8296	**860,0246**	99,2155	838,8757	548,2518	**495,4477**	81,55381
MD-2-relat	5784,905	10817,6	8362,283	**8321,598**	8967,062	9757,019	7002,934	**8575,672**	7683,001
BGLU46 be	175,1008	443,9147	348,324	**322,4465**	344,0734	178,1568	379,7191	**300,6498**	300,4524
ATELF5A-3,	916,3697	1809,197	1841,685	**1522,417**	1087,545	1419,942	1642,601	**1383,363**	1339,554
ATNUDT14	1227,673	1547,924	1993,527	**1589,708**	1540,316	1726,753	1332,837	**1533,302**	1580,062
Endomemb	4506,592	2465,33	3843,567	**3605,163**	3459,336	4559,659	3652,858	**3890,618**	3662,228
CRLK1 Prot	1103,244	2305,291	3233,645	**2214,06**	3168,641	1244,669	2972,298	**2461,87**	2792,239
CID8 CTC-ir	25040	31962,53	27921,27	**28307,93**	27867,23	30489,25	25867,2	**28074,56**	25416,51
unknown p	114,3401	164,9336	117,8312	**132,3683**	203,2423	87,06061	141,7725	**144,0252**	122,8112
26S protea	35498	38833,44	37704,87	**37345,44**	37320,97	40767,1	38024,88	**38704,32**	40524,26
RANGAP1,	2366,712	3022,968	3336,52	**2908,733**	3020,302	2230,498	3516,618	**2922,473**	3246,535
ORP3C OSE	1364,819	2637,224	2975,172	**2325,738**	2425,591	1449,17	2909,88	**2261,547**	2407,409
Coatomer e	492,4761	242,3736	272,5682	**335,806**	167,2062	579,9291	247,7709	**331,6354**	380,581
Pyruvate ki	7296,356	7498,907	8078,875	**7624,713**	7396,021	8668,2	6458,403	**7507,541**	7523,635
ORC5, ATO	1835,763	1735,707	2001,413	**1857,628**	1851,669	1703,637	2080,04	**1878,449**	1921,736
POP2, GAB	73902,71	68720,68	73405,53	**72009,64**	79589,9	55075,98	73421,7	**69362,53**	71030,03
NAD(P)-bin	33260,35	32395,55	41424,65	**35693,52**	40066,18	31178,52	39326,68	**36857,13**	35042,54
RPT1A regu	12522,91	6551,476	7444,302	**8839,561**	4830,389	11082,55	7426,429	**7779,788**	6566,475
PP2A-3 pro	5829,171	10348,87	6574,866	**7584,302**	11571,02	4991,735	11323,95	**9295,567**	6808,334
Aldolase-ty	1134,125	1448,394	1579,028	**1387,182**	2021,241	1235,814	1334,229	**1530,428**	1401,437
Translation	2296,445	1681,749	2459,254	**2145,816**	2277,039	2900,369	1928,679	**2368,696**	2574,726
KING1 SNF	3568,69	4179,89	4097,489	**3948,69**	4980,093	2275,279	3850,782	**3702,052**	4040,991
myosin hea	3594,359	3051,596	3660,304	**3435,42**	3078,582	3602,114	3322,691	**3334,462**	3316,798
ARM repea	2242,366	5559,084	6403,861	**4735,104**	4707,788	4228,565	3747,89	**4228,081**	4595,097
lactoylgluta	19,37944	31,75793	38,92751	**30,02162**	26,4173	210,3272	15,12242	**83,95563**	62,80079
ATIMD2, IM	1334,918	1740,623	2751,156	**1942,232**	2801,23	1691,806	1443,944	**1978,993**	1709,985
PGY2 Ribos	97196,19	104143,8	98719,49	**100019,8**	74617,52	140786,9	111092,9	**108832,4**	77063,12
AHA10 auto	8132,197	12234,12	12414,31	**10926,88**	9453,54	13989,52	10153,23	**11198,76**	9376,934
Pyruvate ki	1335,354	1603,718	2008,488	**1649,186**	1627,9	1270,018	2443,717	**1780,545**	1740,585
ATBCAT-3,	900,1262	825,2249	1020,611	**915,3206**	1694,991	545,4108	1115,922	**1118,775**	901,4008
ATMTN1, A	16255,32	21105,97	12720,07	**16693,79**	20330,12	12357,46	20036,92	**17574,83**	15769,62
HD domain	2698,143	1945,586	2528,046	**2390,592**	1063,035	4776,949	1367,436	**2402,474**	2452,293
expressed	5081,707	5728,359	5880,825	**5563,63**	6575,833	5277,708	5714,372	**5855,971**	6856,519
Translation	26346,99	28116,23	26471,88	**26978,37**	23612,67	25253,34	32015,03	**26960,35**	23945,35
ATMDAR1,	39487,54	37771,26	40308,41	**39189,07**	35118,14	44041,18	38856,54	**39338,62**	41215,68
TCP-1/cpn6	59090,79	66854,44	67042,32	**64329,18**	76682,24	52848,21	76138,21	**68556,22**	67084
G6PD5 gluc	1271,191	761,263	815,8408	**949,4315**	535,1396	1241,656	763,5821	**846,7924**	642,3255
HAP6 ribop	31649,58	41319,37	37339,46	**36769,47**	42485,39	33791,92	39576,58	**38617,96**	39914,48
EIF2, AteIF3	25746,92	33036,03	33220,13	**30667,69**	37259,13	25589	34325,48	**32391,2**	31780,75
MTO1, CGS	9327,922	5705,586	6826,504	**7286,67**	8811,906	5155,587	7120,398	**7029,297**	6982,941
Aconitase/	11192,74	14573,93	15986,1	**13917,59**	14626,16	14346,4	10661,65	**13211,4**	12774,1
UXS5 UDP-	11148,38	9365,731	8777,857	**9763,988**	8064,309	15323,41	7019,738	**10135,82**	8296,392
ATP binding	47292,52	52496,26	47284,69	**49024,49**	50555,2	46561,88	49734,27	**48950,45**	47888,88
BIO2, BIOB	8010,086	9030,902	10158,62	**9066,537**	5414,035	13841,52	8280,011	**9178,522**	5207,093
MD-2-relat	1286,765	5620,468	5553,339	**4153,524**	5326,648	3483,876	3885,79	**4232,105**	3509,37

PAB4, PABI	6786,12	7558,819	7698,447	**7347,795**	8440,211	6199,544	7863,681	**7501,146**	6589,728
PIP1A, ATP	54575,5	76406,74	79058,93	**70013,73**	69662,19	63971,19	81540,38	**71724,59**	81662,04
MPPBETA I	45190,94	45073,38	43076,62	**44446,98**	28389,77	51743,54	40860,81	**40331,37**	32590,79
Class II aaR	8574,85	8325,125	8169,922	**8356,632**	6978,189	11539,3	9617,345	**9378,278**	7257,236
P-loop cont	75,92793	41,46717	37,35573	**51,58361**	25,17159	73,1332	52,36418	**50,22299**	44,5889
VDAC2, AT'	47234,78	50043	51015,53	**49431,1**	37566,33	59501,82	43496,95	**46855,03**	45240,69
SDH5 succi	9190,293	11757,3	10043,82	**10330,47**	10586,82	7739,058	11471,3	**9932,393**	10082,98
RPS15A rib	25621,57	21446,97	22956,85	**23341,8**	16449,5	41331,73	24818,52	**27533,25**	21911,6
SVL1 SHV3-	8152,185	7893,828	10127,03	**8724,347**	9265,343	6349,495	11056,53	**8890,456**	10138,61
Ribosomal	4800,481	3817,925	3295,164	**3971,19**	1145,572	9414,257	2459,14	**4339,656**	1817,727
ATNADP-M	164892,6	186031,9	196885,4	**182603,3**	223864,4	135961,1	176548,4	**178791,3**	183004,8
MPB2C mo	501,6285	461,3647	496,1834	**486,3922**	365,9257	576,1759	518,735	**486,9455**	466,9265
60S acidic r	11191,76	16835,88	19153,91	**15727,18**	15331,59	16009,16	17050,05	**16130,27**	15263,67
DHDPS, DH	12273,9	9169,224	9672,231	**10371,79**	7700,78	10020,38	12246,66	**9989,274**	10086,08
MOD1, ENI	50230,32	51918,29	54702,46	**52283,69**	65762,87	42779,92	55378,51	**54640,43**	55459,39
RNI-like su	1887,449	1644,827	1952,18	**1828,152**	1326,052	2523,539	1474,231	**1774,607**	1735,79
AOC2 allen	508,1316	336,6182	535,1273	**459,959**	131,9128	474,3388	759,1636	**455,1384**	297,0504
Ribose 5-pl	725,0823	182,5492	121,6738	**343,1018**	199,412	259,1317	235,7036	**231,4158**	316,9848
TRAF-like fa	2123,538	2808,555	2553,163	**2495,085**	2668,569	2906,398	2153,124	**2576,03**	2148,488
NIT2, AtNIT	1768,699	1759,146	1856,543	**1794,796**	1665,879	1502,203	2160,904	**1776,329**	2144,893
ATGSTF9, G	130217,2	99535,05	109366,4	**113039,6**	54937,87	191489,1	81785,71	**109404,2**	80514,85
Peroxidase	400231,4	277652,5	241326,9	**306403,6**	177480,2	619043,7	273001,6	**356508,5**	265489,7
ATVSR3, VS	2700,143	4416,354	2798,577	**3305,025**	2312,179	4768,921	3717,893	**3599,665**	3258,312
NAD-ME1 N	38080,2	28461,4	30964,25	**32501,95**	25170,59	43482,18	27295,73	**31982,83**	27729
Zincin-like r	3510,466	5172,06	3694,905	**4125,81**	4882,518	3102,758	4292,907	**4092,727**	4505,068
HSP91 heat	8654,87	11247,63	9887,836	**9930,113**	11139,1	6902,802	10711,18	**9584,36**	9958,556
unknown p	18030,93	27123,67	25624,81	**23593,14**	25982,74	19547,35	27833,97	**24454,69**	28100,7
ALDH3H1, /	7930,561	9392,745	10178,39	**9167,233**	9329,531	9918,294	7618,564	**8955,463**	7821,317
PFN2, PRO:	44389,55	76144,33	67465,44	**62666,44**	71921,73	51415,71	63088,83	**62142,09**	68518,72
O-Glycosyl	191,4875	175,5601	30,25107	**132,4329**	195,0585	41,76687	259,5397	**165,455**	93,50374
PORB proto	4086,453	4787,144	6322,88	**5065,492**	6023,905	2900,754	5589,846	**4838,168**	4664,652
translocon-	33622,38	34861,73	31617,63	**33367,25**	29096,88	35582,25	33911,97	**32863,7**	29279,63
GRF10, GF1	658,789	600,7156	1171,953	**810,486**	697,4664	1431,685	580,6993	**903,2836**	558,5551
ASK13, SK1	1228,786	740,1561	1161,962	**1043,635**	1674,477	595,5485	2070,111	**1446,712**	2803,556
TCP-1/cpn6	31672,54	30738,07	45009,05	**35806,55**	39908,91	27288,57	36225,99	**34474,49**	35786,58
semialdehy	20846,18	18620,75	19462,91	**19643,28**	20148,84	16889,67	23681,95	**20240,15**	20919,51
PGK phosp	334352	369526,2	391443,9	**365107,4**	424248,7	285225,3	414434,1	**374636**	386550
FBR12, ATE	27843,56	23689,18	29700,83	**27077,86**	21013,96	30075,89	29948,51	**27012,79**	23561,33
Protein kin	1576,777	6215,674	2223,421	**3338,624**	2782,62	2450,837	2465,241	**2566,233**	3183,398
PIP2A, PIP2	36769	59368,69	66702,46	**54280,05**	61502,38	33903,05	69506,18	**54970,54**	63770,11
CAT2 catala	8813,784	11385,64	12434,53	**10877,99**	7632,735	17998,72	12762,3	**12797,92**	7239,605
PP2AA3 pro	19979,43	19452,05	18386,37	**19272,62**	16214,78	25587,88	19469,77	**20424,14**	17080,51
PGI1, PGI p	38128,34	44065,52	43023,54	**41739,14**	53511,88	32581,84	40384,3	**42159,34**	43629,75
ATNADP-M	6405,173	5159,48	5312,776	**5625,81**	3175,536	8166,338	5472,106	**5604,66**	4763,088
NDHJ NADI	96,8962	55,21211	74,14582	**75,41804**	45,13613	165,4587	55,94654	**88,84711**	68,15806
PBD2 20S p	11013,93	8418,097	7880,075	**9104,034**	9510,707	8761,773	9422,683	**9231,721**	9230,67
ATPPC1, PE	21918,55	20149,54	18950,07	**20339,39**	26064,31	15578,07	18889,85	**20177,41**	20224,95
Exostosin fa	7854,346	5805,127	5956,173	**6538,549**	6722,889	6226,676	6394,293	**6447,953**	6715,485
HA7 H(+)-A	1156,697	1523,86	2065,362	**1581,973**	1456,185	1688,673	1342,391	**1495,75**	1662,486
MEE43, ED	100,143	154,1873	138,9435	**131,0913**	68,20941	122,8789	208,0409	**133,0431**	97,3991

RPN10, MC	2477,652	2578,862	2303,43	**2453,315**	3037,326	1417,271	3215,395	**2556,664**	2332,452
Hop1 stres	1253,609	817,9166	850,6672	**974,0643**	796,7233	1860,85	733,0152	**1130,196**	792,3727
ATUK/UPRT	2436,227	2992,164	1695,983	**2374,791**	2206,428	1692,752	3628,785	**2509,322**	2323,555
ATG2 meta	2027,478	2499,537	2255,868	**2260,961**	1409,262	3930,234	2081,027	**2473,508**	1304,279
PMH1, ATR	1595,196	2407,186	1708,559	**1903,647**	2480,518	1118,761	2492,638	**2030,639**	2051,269
GRF3, RCI1	4267,869	6421,211	7965,559	**6218,213**	6175,247	5162,093	7836,781	**6391,373**	5168,053
ROC3 rotar	26434,87	31472,53	35311,53	**31072,97**	20408,51	73476,18	25059,74	**39648,14**	27999,02
SHM4 serir	349438,1	373496,8	367921,1	**363618,7**	407916,9	308427,2	386206	**367516,7**	359569,4
PIP1D, PIP1	19477,17	21688,28	27762,18	**22975,88**	22981,99	20450,93	26851,56	**23428,16**	24796,63
Ribonuclea	1082,332	2027,92	2641,66	**1917,304**	1925,489	1420,136	1890,265	**1745,297**	1845,481
PAF2 20S p	2086,732	3704,45	3189,938	**2993,707**	3988,753	2227,145	3115,521	**3110,473**	3183,492
arginosucci	21708,07	26436,3	27814,82	**25319,73**	32236,72	18760,5	27823,87	**26273,7**	26315,19
CSD3 coppe	1030,971	1377,928	1892,107	**1433,669**	1333,053	1309,654	1458,872	**1367,193**	1403,889
ATTIP2;3, T	10873,83	15460,39	17891,52	**14741,91**	13911,42	13677,39	16574,86	**14721,22**	17692,46
EIF3C, ATEI	14349,46	18731,53	19378,04	**17486,34**	16418	16902,33	19649,97	**17656,76**	17554,17
Dihydrolipo	1565,29	675,9474	1203,864	**1148,367**	1524,623	349,43	1943,845	**1272,633**	1388,343
ACO2, ATA	1605,909	436,6248	616,6883	**886,4074**	535,8303	814,0481	1189,642	**846,5068**	581,5994
Pyridoxal-5	6441,749	7729,35	7994,099	**7388,399**	7162,71	6977,37	8070,067	**7403,382**	7671,537
Tetratricop	2881,127	1715,87	2275,004	**2290,667**	3391,895	1650,215	1895,878	**2312,663**	2407,458
ATRAB8, At	42,29878	336,0388	467,58	**281,9725**	178,5541	149,7857	194,6377	**174,3258**	179,0268
PBB2 20S p	408,2564	596,1111	768,7295	**591,0323**	614,0941	289,5704	948,045	**617,2365**	791,0156
SHV3, MRH	34130,01	37059,41	37282,48	**36157,3**	34713,47	34917,26	38108,9	**35913,21**	39624,8
BOU Mitoc	1941,737	2153,705	1858,896	**1984,779**	1796,572	1709,621	2491,79	**1999,328**	2171,565
PBD1, PRC	23365,97	29223,69	29945,42	**27511,69**	29559,99	24703,84	28054,66	**27439,5**	29344,94
ATB5-A, B5	6132,964	2815,231	3131,391	**4026,529**	2195,687	9290,766	2777,023	**4754,492**	2844,992
TUA4, TOR	13535,55	13558,71	15812,34	**14302,2**	18501,86	9609,055	16734,39	**14948,43**	15497,29

2016_03_26_EDUARDO_1mg_002	2016_03_26_EDUARDO_1mg_003	**MEDIA_1mg**	FC_CONTROL/DMSO	**TAG_CONTROL/DMSO**	FC_CONTROL/1mg	**TAG_CONTROL/1mg**	FC_DMSO/1mg
10616,83	12383,36	**11893,43**	0,108624	DOWN	0,992569	UNCHANGED	9,137634
74,80303	78,83958	**76,90696**	1,110248	UNCHANGED	3,747844	UP	3,375681
702,3568	677,7549	**717,1448**	2,227346	UP	0,054897	DOWN	0,024647
21,50927	7,502815	**12,60225**	1,448848	UNCHANGED	23,65429	UP	16,32627
4139,024	3888,777	**3999,196**	0,470568	DOWN	0,642004	DOWN	1,364316
691,5078	966,3523	**795,4838**	0,821392	UNCHANGED	3,912598	UP	4,763374
0	0	**0**	Unique	UP	Unique	UP	-
0	0	**0**	0,622773	DOWN	Unique	UP	Unique
83,97632	46,96699	**107,8834**	3,86334	UP	40,64345	UP	10,52029
106,2788	152,6797	**130,7715**	1,69834	UP	6,973895	UP	4,106301
1032,777	1404,146	**1288,207**	0,034571	DOWN	0,377005	DOWN	10,90523
1801,318	2169,896	**1928,16**	0,388597	DOWN	1,586114	UP	4,081642
52,35861	20,07981	**28,31037**	0,695871	UNCHANGED	13,00726	UP	18,69206
5941,683	8114,081	**7049,707**	0,713621	UNCHANGED	1,87498	UP	2,627417
1478,846	1201,37	**1426,118**	2,524301	UP	0,815705	UNCHANGED	0,323141
4672,716	5010,164	**5043,161**	0,38107	DOWN	0,864341	UNCHANGED	2,268192
8355,754	7386,747	**7560,1**	0,91661	UNCHANGED	1,633052	UP	1,78162
2946,269	3077,767	**2949,716**	0,797263	UNCHANGED	1,21642	UNCHANGED	1,525744
5129,431	5360,336	**5212,539**	0,855637	UNCHANGED	0,788344	UNCHANGED	0,921353
2122,173	1339,516	**1617,533**	0,291317	DOWN	1,70738	UP	5,860905
5735,65	5334,794	**5350,445**	0,500467	DOWN	1,310678	UNCHANGED	2,618912
1345,769	1481,294	**1427,197**	1,226436	UNCHANGED	2,075946	UP	1,692665
10272,99	12197,81	**11556,9**	1,00782	UNCHANGED	1,86519	UP	1,850717
1373,877	1350,217	**1412,61**	1,407658	UNCHANGED	1,863101	UP	1,323547
3562,136	2496,735	**3076,012**	1,201236	UNCHANGED	2,013143	UP	1,675892
17652,09	19595,17	**18591,03**	0,791149	UNCHANGED	1,134787	UNCHANGED	1,434354
337,8992	700,2024	**616,492**	0,238902	DOWN	3,663325	UP	15,33402
5062,15	5681,312	**5389,974**	0,826843	UNCHANGED	0,761726	UNCHANGED	0,921246
2455,25	2601,954	**2510,563**	1,080942	UNCHANGED	1,654376	UP	1,530495
69868,48	78859,03	**77037,83**	0,548964	DOWN	0,766567	UNCHANGED	1,396388
3129,348	4916,735	**4242,823**	0,752107	UNCHANGED	0,209669	DOWN	0,278776
19587,57	21189,14	**20568,41**	1,242093	UNCHANGED	1,2587	UNCHANGED	1,01337
2500,07	2330,771	**2449,064**	0,827309	UNCHANGED	4,400134	UP	5,318612
1271,988	1303,926	**1325,469**	0,574887	DOWN	0,641362	DOWN	1,115633
12211,7	10644,1	**10742,87**	0,783481	UNCHANGED	1,556016	UP	1,986028
2952,587	2433,768	**2617,233**	1,027003	UNCHANGED	1,984247	UP	1,932075
24,64278	11,15007	**30,92218**	0,648344	DOWN	14,27078	UP	22,01114
2099,9	2055,395	**2059,003**	0,921069	UNCHANGED	0,553385	DOWN	0,600807

555,4494	346,619	**426,1471**	0,176057	DOWN	0,550392	DOWN	3,12621
5379,205	5897,66	**5971,987**	0,775859	UNCHANGED	1,152066	UNCHANGED	1,484891
2771,191	1546,99	**1951,801**	0,260142	DOWN	0,475645	DOWN	1,828403
1694,726	2069,589	**1871,996**	0,677303	UNCHANGED	1,320864	UNCHANGED	1,95018
827,8124	1751,243	**1395,34**	0,156939	DOWN	0,714895	UNCHANGED	4,555248
2988,576	2900,245	**2898,73**	0,918218	UNCHANGED	0,720628	UNCHANGED	0,784811
16,30242	18,24787	**36,96506**	1,830318	UP	18,10225	UP	9,890222
2680,125	3045,918	**2867,213**	0,496497	DOWN	1,090017	UNCHANGED	2,195418
282,0547	529,5302	**426,6199**	0,767855	UNCHANGED	3,299432	UP	4,296948
8726,938	9910,376	**9478,757**	0,770055	UNCHANGED	0,705187	UNCHANGED	0,915762
230,144	51,15141	**160,5124**	1,798155	UP	19,45949	UP	10,82192
13989,62	14806,49	**14656,95**	0,966612	UNCHANGED	1,228111	UNCHANGED	1,270532
4545,611	3956,248	**4203,033**	0,797896	UNCHANGED	0,702772	UNCHANGED	0,880782
1055,707	1194,757	**1037,163**	1,016499	UNCHANGED	2,505843	UP	2,465169
8130,25	12731,87	**10815,15**	0,541867	DOWN	0,016429	DOWN	0,030319
10154,46	10522,39	**10182,71**	1,156961	UNCHANGED	1,382448	UNCHANGED	1,194896
1011,003	1030,533	**1111,973**	0,800089	UNCHANGED	3,594944	UP	4,49318
7631,855	9800,455	**8645,947**	1,02405	UNCHANGED	1,561088	UP	1,524425
15783,31	19866,91	**18233,94**	0,815271	UNCHANGED	0,553319	DOWN	0,678694
3295,597	5189,444	**5493,872**	0,195952	DOWN	0,610661	DOWN	3,116384
0	0	**0**	Unique	UP	Unique	UP	-
14852,22	16462,99	**15968,61**	1,31041	UNCHANGED	1,217817	UNCHANGED	0,92934
7513,283	7347,465	**7314,941**	0,650171	DOWN	0,50354	DOWN	0,774473
3952,991	5486,624	**4994,447**	0,759185	UNCHANGED	0,487432	DOWN	0,642046
234,4851	302,5044	**244,884**	0,769097	UNCHANGED	3,722244	UP	4,83976
15431,92	14296,2	**14373,23**	0,857439	UNCHANGED	0,656469	DOWN	0,765617
1970,616	3600,086	**3088,307**	0,693818	UNCHANGED	0,254298	DOWN	0,366519
124,9871	213,0732	**199,1027**	1,322018	UNCHANGED	9,710114	UP	7,344919
4987,195	5115,283	**5110,012**	0,698353	UNCHANGED	1,290412	UNCHANGED	1,847793
2669,546	2547,884	**2570,484**	0,716862	UNCHANGED	0,680045	UNCHANGED	0,948641
7485,559	5740,004	**5840,82**	0,827496	UNCHANGED	1,751191	UP	2,116254
8448,212	7932,822	**8054,391**	0,798358	UNCHANGED	0,596599	DOWN	0,747283
1855,595	2124,984	**1968,271**	1,156452	UNCHANGED	1,851003	UP	1,600588
1638,883	1359,169	**1390,483**	0,276131	DOWN	0,565206	DOWN	2,046877
115059,8	237888,1	**199513,9**	0,957646	UNCHANGED	0,254953	DOWN	0,266229
3975,828	3612,137	**3687,545**	1,383892	UNCHANGED	1,769489	UP	1,278632
4172,583	2814,214	**3287,352**	1,000376	UNCHANGED	2,398837	UP	2,397934
7003,382	9625,438	**8677,432**	0,615207	DOWN	0,446458	DOWN	0,725705
3946,524	9036,994	**7594,453**	1,232281	UNCHANGED	0,213637	DOWN	0,173367
10288,28	9379,989	**10133,92**	0,780005	UNCHANGED	0,426856	DOWN	0,547247
20274,46	22556,86	**21279,15**	0,805473	UNCHANGED	0,723835	UNCHANGED	0,898647
11666,93	12105,22	**12156,3**	0,580406	DOWN	0,888027	UNCHANGED	1,530009
8254,8	8458,011	**8178,66**	0,853876	UNCHANGED	0,817437	UNCHANGED	0,957325
3230,433	3621,647	**3445,417**	0,991483	UNCHANGED	1,376613	UNCHANGED	1,388439
6641,344	7026,26	**6937,701**	0,933847	UNCHANGED	0,707496	UNCHANGED	0,757614
9271,069	9349,831	**9446,045**	0,565436	DOWN	0,850042	UNCHANGED	1,503341
3300,317	4090,006	**3801,022**	0,658467	DOWN	0,534208	DOWN	0,81129
12273,25	12778,81	**13373,38**	1,526635	UP	0,683638	UNCHANGED	0,447807
69,24084	36,55109	**82,8989**	1,931654	UP	17,43149	UP	9,024123
20009,93	18611,4	**19134,47**	1,198508	UNCHANGED	1,047519	UNCHANGED	0,87402

23269,2	24765,05	**24245,83**	0,789781	UNCHANGED	0,800657	UNCHANGED	1,013771
13841,78	14957,72	**14316,84**	0,97815	UNCHANGED	1,27282	UNCHANGED	1,301252
8417,967	13644,64	**11622,02**	0,643883	DOWN	0,461668	DOWN	0,717006
35764,43	28949,85	**32966,88**	0,936948	UNCHANGED	1,661943	UP	1,773784
5027,703	5076,452	**5169,999**	2,019239	UP	2,22586	UP	1,102326
1963,233	1504,401	**1609,14**	1,039435	UNCHANGED	1,68514	UP	1,621208
50599,84	52337,11	**53036,98**	0,813812	UNCHANGED	1,115061	UNCHANGED	1,370171
516,7153	225,5568	**449,7489**	1,830674	UP	4,149044	UP	2,266402
12851,81	9425,435	**12985,53**	1,130855	UNCHANGED	2,44068	UP	2,158261
16268,14	14362,75	**15453,23**	1,211178	UNCHANGED	1,474576	UNCHANGED	1,217473
7287,335	7422,151	**7123,65**	0,491756	DOWN	1,422788	UNCHANGED	2,893279
4645,583	4751,92	**4561,524**	1,049698	UNCHANGED	1,717527	UP	1,636211
46118,65	42994,02	**44131,89**	1,175794	UNCHANGED	1,247882	UNCHANGED	1,06131
379,2894	284,5112	**298,6427**	0,146449	DOWN	0,659739	DOWN	4,504902
1143,156	957,9674	**995,0253**	0,487875	DOWN	0,735592	UNCHANGED	1,507747
5174,202	4798,976	**5057,761**	1,445411	UNCHANGED	1,316534	UNCHANGED	0,910837
2170,724	1970,739	**2125,004**	1,380698	UNCHANGED	1,780994	UP	1,289923
141,0396	133,2386	**115,7127**	0,389406	DOWN	1,614466	UP	4,145967
9138,726	11921,16	**11141,53**	0,999731	UNCHANGED	0,601914	DOWN	0,602075
7712,542	10775,61	**9229,748**	0,768043	UNCHANGED	1,272341	UNCHANGED	1,656602
4790,321	5660,805	**4897,029**	1,038289	UNCHANGED	1,710798	UP	1,647709
8590,573	9204,907	**8854,783**	0,883169	UNCHANGED	1,150742	UNCHANGED	1,302969
5274,928	7978,569	**6877,636**	0,894941	UNCHANGED	0,308009	DOWN	0,344167
1240,853	1177,887	**1223,118**	0,600929	DOWN	0,606403	DOWN	1,009109
343,2899	487,8046	**389,588**	2,012092	UP	2,504159	UP	1,244555
39,90062	49,98587	**40,39899**	0,485399	DOWN	5,215601	UP	10,74497
2853,482	2852,698	**3193,619**	2,261471	UP	2,700803	UP	1,194268
10941,41	10669,75	**11287,53**	1,772752	UP	1,963186	UP	1,107423
2598,771	2312,902	**2508,46**	1,151615	UNCHANGED	0,804289	UNCHANGED	0,698401
2196,658	2538,156	**2354,277**	0,958458	UNCHANGED	1,423409	UNCHANGED	1,485103
1475,566	1368,658	**1371,05**	1,580371	UP	0,827131	UNCHANGED	0,523377
2223,737	2598,84	**2447,92**	0,913985	UNCHANGED	0,67925	UNCHANGED	0,743174
676,0453	774,3443	**752,0272**	0,776539	UNCHANGED	1,757161	UP	2,262811
31991,9	34613,29	**34525,12**	0,84855	UNCHANGED	1,47194	UNCHANGED	1,734653
6989,679	6420,109	**7235,512**	0,803918	UNCHANGED	0,697939	UNCHANGED	0,868172
2364,869	1976,45	**2194,287**	1,652101	UP	1,922252	UP	1,16352
214,5952	199,4831	**226,546**	1,720885	UP	12,74903	UP	7,408413
7736,685	7095,711	**7340,523**	0,913644	UNCHANGED	1,379285	UNCHANGED	1,509653
215,3508	401,5202	**296,5918**	0,960657	UNCHANGED	6,210625	UP	6,464974
18111,84	17060,28	**17614,63**	1,212292	UNCHANGED	1,114714	UNCHANGED	0,919509
4034,836	4543,006	**4320,131**	0,66496	DOWN	0,501052	DOWN	0,753506
2627,539	4166,233	**3482,519**	0,904649	UNCHANGED	0,547435	DOWN	0,605135
20584,59	21473,28	**20624,02**	0,968417	UNCHANGED	1,468586	UNCHANGED	1,516482
1975,812	2295,498	**2158,656**	1,742865	UP	2,591796	UP	1,487089
1513,766	1164,749	**1324,385**	0,947933	UNCHANGED	2,782928	UP	2,935786
12597,95	10594,9	**11154,72**	1,443872	UNCHANGED	1,449637	UNCHANGED	1,003993
4498,937	7068,094	**6100,144**	0,933815	UNCHANGED	0,447672	DOWN	0,479401
6492,064	6583,368	**6560,456**	1,15938	UNCHANGED	1,943458	UP	1,67629
2364,935	1506,165	**1844,55**	1,145087	UNCHANGED	2,493857	UP	2,177875
19406,74	14779,32	**16592,26**	1,411617	UNCHANGED	1,010109	UNCHANGED	0,715569

435,1606	449,8055	**439,1232**	1,806171	UP	8,134704	UP	4,50384
47144,33	67701,94	**60500,25**	1,381853	UNCHANGED	0,683692	UNCHANGED	0,494764
14754,89	14578,65	**14662,71**	1,205679	UNCHANGED	1,320658	UNCHANGED	1,095365
468,0677	445,8011	**466,1896**	0,91557	UNCHANGED	0,794684	UNCHANGED	0,867966
4368,278	4902,971	**4762,969**	1,959525	UP	2,694608	UP	1,375134
7665,805	7598,927	**7726,823**	1,427984	UNCHANGED	2,225575	UP	1,558543
8300,026	6628,834	**6941,913**	0,924215	UNCHANGED	1,715642	UP	1,856323
39924,92	41647,92	**41903,73**	1,408593	UNCHANGED	1,415489	UNCHANGED	1,004895
678,0589	590,9879	**603,1624**	0,465832	DOWN	0,463989	DOWN	0,996045
10975,74	14361,46	**12658,33**	1,108936	UNCHANGED	2,630166	UP	2,371791
5305,792	4738,457	**4854,991**	1,099945	UNCHANGED	0,838655	UNCHANGED	0,762452
23071,98	15035,51	**17268,39**	0,596849	DOWN	0,55466	DOWN	0,929314
10715,51	10429,25	**10316,78**	1,257338	UNCHANGED	1,905151	UP	1,515227
1555,351	772,0794	**1090,813**	0,294987	DOWN	0,606742	DOWN	2,05684
576,3867	564,2091	**557,5033**	1,793173	UP	0,832236	UNCHANGED	0,464114
4946,946	4851,079	**4989,412**	0,343107	DOWN	0,837916	UNCHANGED	2,442144
83263,07	143372,3	**121004,8**	0,679061	UNCHANGED	0,383463	DOWN	0,564695
4307,502	2040,6	**2857,667**	0,23111	DOWN	0,763585	UNCHANGED	3,303985
15726,53	22816,18	**20458,78**	0,747	UNCHANGED	0,521397	DOWN	0,697988
1357,48	1557,35	**1574,633**	0,601287	DOWN	0,481499	DOWN	0,800781
22867,94	21149,39	**21724,19**	0,886227	UNCHANGED	1,092393	UNCHANGED	1,232633
8611,684	5866,122	**6975,642**	1,367962	UNCHANGED	2,149275	UP	1,571151
2044,683	1650,754	**1649,63**	1,53404	UP	2,209676	UP	1,440429
6648,606	7436,708	**7262,819**	1,702326	UP	1,360495	UNCHANGED	0,799197
797,0299	472,0084	**585,9724**	0,439663	DOWN	0,538681	DOWN	1,225214
2518,079	2440,182	**2457,381**	0,867411	UNCHANGED	1,410096	UNCHANGED	1,625638
2673,557	3895,452	**3739,581**	1,295604	UNCHANGED	0,440612	DOWN	0,340082
17480,52	20168,17	**19241,56**	0,850616	UNCHANGED	1,348482	UNCHANGED	1,5853
1220,753	1783,601	**1613,534**	1,281818	UNCHANGED	2,042425	UP	1,593381
13536,05	18472,8	**16599,74**	0,601659	DOWN	0,748715	UNCHANGED	1,244417
56420,86	64847,84	**60125,64**	0,957395	UNCHANGED	0,844116	UNCHANGED	0,88168
1555,651	518,6419	**890,041**	0,830993	UNCHANGED	3,226102	UP	3,882225
58791,37	61830,43	**61186,67**	0,801077	UNCHANGED	0,924144	UNCHANGED	1,153626
2191,869	2373,898	**2163,454**	1,003456	UNCHANGED	1,944222	UP	1,937526
5132,538	4861,049	**4913,931**	0,591435	DOWN	0,905897	UNCHANGED	1,531692
11173,36	10992,09	**11446,3**	1,309474	UNCHANGED	1,385594	UNCHANGED	1,05813
2606,21	2480,791	**2594,538**	0,670997	UNCHANGED	2,009207	UP	2,994361
28181,55	25466,65	**27037,54**	0,920409	UNCHANGED	1,387689	UNCHANGED	1,507687
95169,16	112483,9	**109751,4**	0,81269	UNCHANGED	1,367912	UNCHANGED	1,68319
4,801973	13,08105	**12,97015**	2,845522	UP	19,37286	UP	6,808193
768,76	1616,987	**1044,762**	1,670737	UP	2,769528	UP	1,657669
1431,355	1040,238	**1158,018**	0,759892	UNCHANGED	0,646115	DOWN	0,850273
954,2043	1256,281	**1054,041**	0,716821	UNCHANGED	0,424222	DOWN	0,59181
927,8935	1120,142	**972,1326**	0,547796	DOWN	0,844273	UNCHANGED	1,541219
1410,465	1464,489	**1453,941**	1,457972	UNCHANGED	0,826975	UNCHANGED	0,567209
6776,833	7218,276	**6852,897**	0,662576	DOWN	0,597836	DOWN	0,902291
5827,147	5507,393	**5579,105**	1,076441	UNCHANGED	1,297135	UNCHANGED	1,205022
62863,43	71808,92	**69213,13**	0,823657	UNCHANGED	0,981845	UNCHANGED	1,192056
39972,49	36774,77	**37700,76**	1,079237	UNCHANGED	1,314015	UNCHANGED	1,217541
1965,506	2237,655	**2089,362**	1,191869	UNCHANGED	1,545714	UP	1,296882

323,2732	203,8351	**264,8729**	0,556592	DOWN	2,29269	UP	4,119155
27293,53	32715,83	**30937,77**	0,755104	UNCHANGED	0,762977	UNCHANGED	1,010426
71910,9	67094,33	**70582,51**	1,183686	UNCHANGED	1,04099	UNCHANGED	0,879448
7441,194	7025,881	**7078,671**	1,08221	UNCHANGED	1,705265	UP	1,575724
7923,578	9115,029	**7972,95**	1,302576	UNCHANGED	1,444825	UNCHANGED	1,109206
16290,73	14271,16	**15223,66**	1,12236	UNCHANGED	1,221341	UNCHANGED	1,08819
0	0	**0**	0,718295	UNCHANGED	Unique	UP	Unique
10185,2	15233,48	**13186,73**	0,801786	UNCHANGED	0,547751	DOWN	0,683163
11415,01	12048,63	**12050,8**	0,704938	UNCHANGED	0,89035	UNCHANGED	1,263019
18294,77	12486,72	**14684,45**	1,50844	UP	1,733194	UP	1,148998
3316,001	3558,005	**3462,35**	0,840235	UNCHANGED	1,381251	UNCHANGED	1,643886
11963,51	15269,87	**14253,08**	0,603762	DOWN	1,079611	UNCHANGED	1,788138
41298,17	55936,2	**51174,09**	0,976941	UNCHANGED	1,412657	UNCHANGED	1,446
18608,14	22667,18	**21530,94**	1,492315	UNCHANGED	2,265009	UP	1,517782
7588,635	8808,226	**8413,616**	1,021692	UNCHANGED	1,225407	UNCHANGED	1,199389
1806,809	1827,453	**1767,752**	1,709415	UP	2,774887	UP	1,623296
21140,4	20024,47	**20006,27**	0,888243	UNCHANGED	0,681714	UNCHANGED	0,767486
3134,416	4151,047	**3509,644**	0,327962	DOWN	0,311695	DOWN	0,950401
2904,467	3519,086	**3316,799**	0,375473	DOWN	0,854126	UNCHANGED	2,274798
7633,993	2480,84	**4239,207**	0,272251	DOWN	0,88172	UNCHANGED	3,238633
3926,845	4678,125	**4317,395**	0,668093	UNCHANGED	0,540789	DOWN	0,809451
55734,2	56564,05	**56383,61**	1,232616	UNCHANGED	1,33106	UNCHANGED	1,079866
3091,94	3130,933	**3146,324**	1,439847	UNCHANGED	1,087554	UNCHANGED	0,755327
2969,476	2089,637	**2534,95**	1,310618	UNCHANGED	3,056466	UP	2,332081
838,7005	652,1601	**700,1873**	0,729427	UNCHANGED	1,708203	UP	2,341844
5051,453	4601,611	**4807,535**	1,363693	UNCHANGED	1,185391	UNCHANGED	0,86925
339,3826	378,8011	**434,9882**	1,230299	UNCHANGED	0,545557	DOWN	0,443434
7895,257	8345,446	**8289,398**	1,392738	UNCHANGED	1,436917	UNCHANGED	1,031721
2835,421	2933,856	**2838,874**	0,951531	UNCHANGED	1,548198	UP	1,627061
1194,951	558,87	**808,08**	2,541146	UP	0,79689	UNCHANGED	0,313595
11179,93	7373,382	**8894,968**	0,434972	DOWN	0,885909	UNCHANGED	2,036705
1093,667	938,7074	**949,5638**	0,919867	UNCHANGED	1,507213	UP	1,638511
493,9016	506,5567	**569,8896**	5,12848	UP	2,779014	UP	0,541879
456,9833	528,3094	**487,3042**	0,276955	DOWN	0,53025	DOWN	1,914568
1157,422	1950,663	**1623,668**	0,513625	DOWN	0,116755	DOWN	0,227316
409,4715	471,2356	**385,9444**	0,362	DOWN	0,1814	DOWN	0,501106
72176,73	72093,99	**72686,53**	0,99512	UNCHANGED	1,192612	UNCHANGED	1,198461
251,1364	154,893	**192,8278**	2,188694	UP	0,470423	DOWN	0,214933
11743,84	10439,07	**10728,23**	1,292112	UNCHANGED	1,701905	UP	1,317149
641,8244	787,2485	**740,0823**	1,16204	UNCHANGED	1,797322	UP	1,546696
806,2456	830,612	**776,6732**	0,617097	DOWN	0,74701	UNCHANGED	1,210524
39595,52	41380,85	**41057,73**	1,05711	UNCHANGED	1,235171	UNCHANGED	1,168442
1884,949	1947,401	**1886,913**	1,192948	UNCHANGED	1,818656	UP	1,524506
1386,971	1027,212	**1252,685**	0,488822	DOWN	0,490918	DOWN	1,004289
580,3494	471,6475	**544,4381**	1,821921	UP	1,340489	UNCHANGED	0,735756
244,5264	230,8527	**213,337**	0,873752	UNCHANGED	2,607905	UP	2,984721
9237,816	9713,575	**9331,97**	0,964219	UNCHANGED	1,455512	UNCHANGED	1,509525
3641,943	8687,319	**6335,852**	0,558407	DOWN	0,118203	DOWN	0,211678
6574,714	5489,442	**5768,171**	0,638029	DOWN	0,628198	DOWN	0,984593
547,7596	534,1183	**496,012**	1,738922	UP	1,936785	UP	1,113785

18795,04	18908,2	**19178,47**	1,044029	UNCHANGED	1,251383	UNCHANGED	1,19861
66,08877	66,46202	**119,5388**	0,730431	UNCHANGED	2,917548	UP	3,994284
4509,313	5272,913	**5175,033**	0,520999	DOWN	0,949353	UNCHANGED	1,822179
1844,377	2194,838	**2011,83**	1,41097	UNCHANGED	0,764876	UNCHANGED	0,542093
2903,072	2979,228	**3051,882**	1,199486	UNCHANGED	1,344656	UNCHANGED	1,121027
87625,39	98045,8	**93777,62**	1,007288	UNCHANGED	0,794468	UNCHANGED	0,78872
7707,925	9857,395	**9403,37**	0,926185	UNCHANGED	1,396983	UNCHANGED	1,50832
8196,944	8125,303	**9127,674**	0,849134	UNCHANGED	1,367902	UNCHANGED	1,610938
61974,64	30462,9	**51688,93**	1,385191	UNCHANGED	3,298146	UP	2,381004
4947,382	3277,722	**3864,259**	2,584948	UP	2,528273	UP	0,978075
1188,513	949,8676	**1059,032**	2,140019	UP	1,906301	UP	0,890787
27182,2	22228,26	**23696,13**	1,09184	UNCHANGED	1,486789	UNCHANGED	1,361728
1649,675	1145,981	**1296,943**	0,656349	DOWN	0,513839	DOWN	0,782875
2821,837	2548,721	**2669,641**	1,021058	UNCHANGED	0,748052	UNCHANGED	0,732624
1139,025	989,2249	**952,0382**	0,378246	DOWN	0,674732	UNCHANGED	1,783846
64051,59	51311,14	**54491,2**	0,597066	DOWN	0,947787	UNCHANGED	1,587408
1831,636	1974,939	**1881,838**	0,802794	UNCHANGED	0,641553	DOWN	0,799151
32639,23	40138,58	**35996,61**	0,757469	UNCHANGED	1,009097	UNCHANGED	1,332195
5473,501	4092,774	**4398,77**	0,603328	DOWN	0,344774	DOWN	0,571454
6754,267	23302,61	**17965,03**	0,250392	DOWN	0,53922	DOWN	2,153503
16483,29	12827,44	**14100,13**	1,416224	UNCHANGED	1,15277	UNCHANGED	0,813974
5996,86	5408,047	**5557,827**	3,345593	UP	0,342818	DOWN	0,102469
10583,91	12737,58	**11902,33**	1,454243	UNCHANGED	1,494656	UNCHANGED	1,02779
102,3275	25,8002	**57,16481**	2,785066	UP	11,95392	UP	4,29215
16319,31	17933,17	**17825,51**	0,986972	UNCHANGED	1,271418	UNCHANGED	1,2882
1191,23	1083,639	**1280,063**	1,082772	UNCHANGED	1,753298	UP	1,619267
740,4783	976,4059	**893,2334**	1,393761	UNCHANGED	1,567309	UP	1,124518
12038,34	8318,826	**9655,115**	1,58989	UP	1,543921	UP	0,971087
4900,341	4537,376	**4742,998**	1,300874	UNCHANGED	1,132264	UNCHANGED	0,870388
26392,14	23563,11	**24497,22**	1,034437	UNCHANGED	0,907071	UNCHANGED	0,876874
507,0655	549,007	**521,7559**	1,292074	UNCHANGED	1,424463	UNCHANGED	1,102463
1482,844	1077,089	**1196,899**	0,675566	UNCHANGED	2,015485	UP	2,983403
14777,7	4972,308	**7808,913**	0,321471	DOWN	1,066496	UNCHANGED	3,317543
20984,73	19843,02	**20610,01**	1,227675	UNCHANGED	1,10418	UNCHANGED	0,899407
4798,348	4516,668	**4576,01**	1,358148	UNCHANGED	1,104523	UNCHANGED	0,813257
6468,208	10159,96	**8309,283**	1,351979	UNCHANGED	2,198733	UP	1,626307
945,7271	1131,917	**1021,402**	0,748657	UNCHANGED	1,280391	UNCHANGED	1,710251
4269,85	3746,65	**3927,041**	0,693508	UNCHANGED	0,906684	UNCHANGED	1,307388
26728,93	18892,83	**21537,69**	1,256675	UNCHANGED	1,702787	UP	1,354994
13055,02	13595,58	**13310,98**	0,709708	UNCHANGED	0,890311	UNCHANGED	1,254474
169,5391	275,9412	**222,3616**	0,251415	DOWN	0,738257	UNCHANGED	2,936402
2520,632	1121,699	**1597,822**	2,109375	UP	0,832876	UNCHANGED	0,394845
18805,73	13147,26	**14730,28**	1,127947	UNCHANGED	1,854028	UP	1,64372
617,5039	496,5904	**572,4408**	0,620565	DOWN	0,865099	UNCHANGED	1,39405
30,21815	75,52201	**55,45204**	1,386751	UNCHANGED	2,521879	UP	1,818552
861,2769	404,0493	**603,1481**	5,956808	UP	4,158967	UP	0,698187
38266,15	33050,14	**34617,52**	1,281663	UNCHANGED	1,210557	UNCHANGED	0,94452
6501,25	5393,31	**5802,966**	0,923935	UNCHANGED	2,045345	UP	2,213733
255,6276	129,9212	**171,2197**	0,58055	DOWN	0,244222	DOWN	0,420674
6207,184	6274,222	**6239,446**	0,888287	UNCHANGED	0,776337	UNCHANGED	0,87397

339,4871	837,3533	**621,3935**	1,543155	UP	6,909091	UP	4,477251
5441,335	7031,4	**6566,956**	0,717734	UNCHANGED	1,084195	UNCHANGED	1,510581
143922	182773,5	**171074,7**	0,857677	UNCHANGED	1,397878	UNCHANGED	1,629841
1393,293	1115,967	**1232,928**	0,524048	DOWN	0,808622	UNCHANGED	1,543029
14757,45	15554,62	**15085,67**	0,863621	UNCHANGED	0,716574	UNCHANGED	0,829732
11453,79	13231,59	**12338,8**	1,033055	UNCHANGED	1,139513	UNCHANGED	1,103052
17575,26	24205,05	**21622,65**	0,56765	DOWN	1,014609	UNCHANGED	1,787384
8810,862	8330,658	**8589,351**	1,229001	UNCHANGED	1,306542	UNCHANGED	1,063093
8277,682	7717,874	**7936,694**	0,962189	UNCHANGED	0,780884	UNCHANGED	0,81157
1210,161	1847,791	**1558,593**	1,011074	UNCHANGED	1,499036	UNCHANGED	1,482617
5777,617	4899,763	**5135,431**	0,438946	DOWN	0,263982	DOWN	0,601401
27017,49	26920,99	**26293,12**	1,227101	UNCHANGED	1,033438	UNCHANGED	0,842178
432,9498	316,0081	**414,186**	1,381179	UNCHANGED	5,297218	UP	3,835288
103132,5	100931,5	**97555,3**	1,024815	UNCHANGED	0,770909	UNCHANGED	0,752242
833,9596	924,2595	**919,0328**	1,547013	UP	2,610842	UP	1,687667
8298,104	7531,892	**7543,431**	1,165651	UNCHANGED	1,278773	UNCHANGED	1,097046
3905,787	5259,129	**4636,443**	0,766562	UNCHANGED	0,99698	UNCHANGED	1,300586
95,41487	70,12095	**111,4957**	0,568059	DOWN	2,127144	UP	3,744585
7316,773	18789,96	**16531,57**	0,972884	UNCHANGED	0,173232	DOWN	0,178061
35016,52	29693,6	**31043**	0,923754	UNCHANGED	0,657801	DOWN	0,712095
76912,14	89638,67	**82949,56**	0,888397	UNCHANGED	1,24638	UNCHANGED	1,402953
5706,572	5206,181	**5362,125**	1,099114	UNCHANGED	1,561189	UP	1,420406
5005,67	5134,241	**5045,949**	1,433108	UNCHANGED	1,568017	UP	1,094137
10435,55	14346,51	**12835,45**	1,761413	UP	0,865011	UNCHANGED	0,491089
12908,33	9830,221	**12632,68**	1,638622	UP	1,623192	UP	0,990584
10792,81	11672,33	**11327,24**	0,77877	UNCHANGED	1,01286	UNCHANGED	1,300589
8536,389	7214,124	**7665,702**	1,070676	UNCHANGED	1,944919	UP	1,816534
93584,65	108801,6	**105378,2**	1,611326	UP	1,30972	UNCHANGED	0,812821
92,20752	71,24659	**75,58141**	3,208265	UP	1,738188	UP	0,541784
4399,371	4983,099	**5051,598**	0,485311	DOWN	0,768215	UNCHANGED	1,582932
4696,139	3243,294	**3999,634**	1,219335	UNCHANGED	1,38694	UNCHANGED	1,137456
3469,741	3352,758	**3462,104**	0,472882	DOWN	0,549885	DOWN	1,162838
2237,633	1761,067	**1860,214**	1,345572	UNCHANGED	7,172788	UP	5,330662
4785,317	4883,531	**4928,592**	1,345858	UNCHANGED	1,428227	UNCHANGED	1,061201
370,5958	264,1439	**297,5241**	0,810496	UNCHANGED	2,09661	UP	2,586823
3247,105	3430,813	**3307,594**	1,15479	UNCHANGED	0,956327	UNCHANGED	0,82814
12962,88	10560,01	**11338,43**	1,466	UNCHANGED	0,921276	UNCHANGED	0,628428
5478,274	5534,344	**5541,842**	1,131231	UNCHANGED	1,442203	UNCHANGED	1,274898
213,2682	375,8663	**312,9287**	1,54381	UP	1,718818	UP	1,11336
7296,804	7785,068	**7586,47**	1,166266	UNCHANGED	1,260314	UNCHANGED	1,08064
22610,16	18515,32	**20724,68**	0,862122	UNCHANGED	0,742445	UNCHANGED	0,861183
23,17849	21,09806	**25,83081**	0,260161	DOWN	3,745288	UP	14,39601
1617,017	2329,328	**2069,008**	0,804458	UNCHANGED	0,48012	DOWN	0,596824
12278,02	15748,27	**14837,47**	0,847112	UNCHANGED	1,593642	UP	1,881265
2328,764	3150,922	**2756,582**	0,663252	DOWN	0,690617	UNCHANGED	1,041259
3135,354	2868,512	**2804,509**	0,39358	DOWN	0,192593	DOWN	0,489338
5918,188	6541,588	**6074,197**	0,934596	UNCHANGED	0,598503	DOWN	0,640386
24827,61	31774,31	**29170,56**	0,73279	UNCHANGED	0,697283	UNCHANGED	0,951546
127744,1	140929,7	**134122,4**	0,834813	UNCHANGED	0,723049	UNCHANGED	0,866121
28621,57	32996,93	**32701,81**	1,055865	UNCHANGED	1,388703	UNCHANGED	1,315228

1167,043	1286,809	**1196,261**	1,032622	UNCHANGED	1,355786	UNCHANGED	1,312955
16545,84	13011,34	**14472,44**	1,296459	UNCHANGED	1,379144	UNCHANGED	1,063777
19438,32	14811,45	**16675,35**	0,954655	UNCHANGED	0,66509	DOWN	0,696681
468,091	415,5022	**473,6988**	0,667323	UNCHANGED	1,741291	UP	2,609366
16038,21	14466,9	**14799,38**	1,260477	UNCHANGED	1,12685	UNCHANGED	0,893987
26208,34	25425	**26099,1**	0,902618	UNCHANGED	1,056684	UNCHANGED	1,170687
18136,4	19572,89	**19033,4**	0,846109	UNCHANGED	0,682539	UNCHANGED	0,80668
29354,25	17576,36	**21839,86**	1,084621	UNCHANGED	1,644551	UP	1,516244
12212,13	15434,48	**14729,38**	1,278743	UNCHANGED	0,891694	UNCHANGED	0,697321
2384,5	2548,635	**2627,916**	0,426069	DOWN	0,31407	DOWN	0,737135
1210,821	1539,317	**1410,769**	2,018549	UP	2,386753	UP	1,18241
730,8674	995,2691	**932,3756**	0,681036	UNCHANGED	0,892543	UNCHANGED	1,310565
2838,874	2629,754	**2683,601**	0,678128	UNCHANGED	0,652183	DOWN	0,96174
7726,892	8566,918	**8528,757**	1,215951	UNCHANGED	1,315471	UNCHANGED	1,081846
4226,508	6688,898	**5249,431**	0,261972	UNCHANGED	0,331354	UNCHANGED	1,264844
406,6228	200,9799	**276,5977**	1,922776	UNCHANGED	1,321902	UNCHANGED	0,687497
6187,407	9190,934	**8202,971**	0,852318	UNCHANGED	0,561992	UNCHANGED	0,659368
1259,397	1405,769	**1340,84**	0,53976	UNCHANGED	0,562719	UNCHANGED	1,042536
628,7482	585,2137	**609,1452**	0,487708	UNCHANGED	0,608662	UNCHANGED	1,248004
135,5904	19,84467	**54,31695**	0,975807	UNCHANGED	4,29587	UNCHANGED	4,402378
33915,26	22827,47	**26968,77**	0,760179	UNCHANGED	0,627833	UNCHANGED	0,825902
1127,116	1153,71	**1131,112**	1,497683	UNCHANGED	1,310017	UNCHANGED	0,874696
1970,024	2246,337	**2281,336**	0,887153	UNCHANGED	1,256131	UNCHANGED	1,415912
6868,77	7139,419	**6941,957**	1,281969	UNCHANGED	1,054956	UNCHANGED	0,822919
1600,247	1108,002	**1238,184**	0,274206	UNCHANGED	1,725875	UNCHANGED	6,294082
5236,166	5973,768	**5595,093**	0,671851	UNCHANGED	0,695559	UNCHANGED	1,035288
12940,99	10055,64	**11220,01**	1,496743	UNCHANGED	1,558837	UNCHANGED	1,041486
13185,63	13165,86	**13502,45**	1,216028	UNCHANGED	1,659521	UNCHANGED	1,364706
1886,489	1180,484	**1438,088**	1,282831	UNCHANGED	1,995963	UNCHANGED	1,555905
28057,59	23899,49	**24960,24**	0,91495	UNCHANGED	1,187414	UNCHANGED	1,297791
681,316	1050,309	**829,151**	1,58752	UNCHANGED	1,811799	UNCHANGED	1,141276
1647,607	1232,198	**1298,528**	1,897703	UNCHANGED	1,792494	UNCHANGED	0,94456
2296,077	2131,255	**2345,604**	2,104069	UNCHANGED	1,63153	UNCHANGED	0,775416
17,71536	49,73761	**34,47192**	0,365065	UNCHANGED	3,272595	UNCHANGED	8,964425
120,9912	227,7125	**137,6464**	0,589137	UNCHANGED	2,580554	UNCHANGED	4,380231
2368,395	2450,857	**2089,443**	1,821888	UNCHANGED	1,399766	UNCHANGED	0,768305
106,3686	83,88303	**65,40714**	1,021389	UNCHANGED	9,505343	UNCHANGED	9,306289
442,694	551,17	**549,6629**	1,257882	UNCHANGED	0,530071	UNCHANGED	0,421399
840,193	320,0963	**503,1687**	2,196063	UNCHANGED	2,699523	UNCHANGED	1,229255
9965,896	10419,14	**10410,67**	0,93529	UNCHANGED	1,219071	UNCHANGED	1,303415
53,90201	105,7974	**73,51637**	3,120104	UNCHANGED	6,83892	UNCHANGED	2,191888
10,75781	19,25394	**25,26829**	0,054265	UNCHANGED	1,048746	UNCHANGED	19,32634
10631,81	15087,85	**13408,14**	0,800886	UNCHANGED	0,635452	UNCHANGED	0,793437
10939,66	12242,22	**11454,28**	0,998697	UNCHANGED	0,88038	UNCHANGED	0,881528
18140,57	20200,33	**19436,04**	1,005809	UNCHANGED	1,361726	UNCHANGED	1,353861
5056,733	4886,537	**4997,26**	0,936143	UNCHANGED	1,233148	UNCHANGED	1,317265
1363,294	1379,409	**1375,283**	1,76438	UNCHANGED	1,2716	UNCHANGED	0,720706
48791,85	65856,68	**59950,34**	0,809759	UNCHANGED	1,267413	UNCHANGED	1,565173
15938,11	11004,08	**12849,81**	1,421334	UNCHANGED	1,424754	UNCHANGED	1,002407
2547,965	2628,774	**2536,921**	0,461814	UNCHANGED	0,466295	UNCHANGED	1,009702

2138,778	2712,863	**2636,721**	0,653693	UNCHANGED	0,885436	UNCHANGED	1,354514
15531,83	18867,04	**17381,37**	0,808198	UNCHANGED	0,641175	UNCHANGED	0,79334
7235,911	5912,083	**6051,402**	0,804497	UNCHANGED	1,263084	UNCHANGED	1,570029
382,5762	396,9766	**431,6112**	2,789795	UNCHANGED	4,642468	UNCHANGED	1,664089
1660,256	1757,604	**1661,316**	0,861851	UNCHANGED	1,286213	UNCHANGED	1,492384
1842,286	1817,508	**1852,719**	1,528264	UNCHANGED	1,160359	UNCHANGED	0,759266
627,7555	578,3678	**602,9474**	0,464882	UNCHANGED	1,146195	UNCHANGED	2,465562
4433,06	4836,082	**3913,746**	0,725617	UNCHANGED	0,568764	UNCHANGED	0,783835
8068,441	5113,181	**5985,221**	1,240901	UNCHANGED	0,447018	UNCHANGED	0,360237
6304,609	8140,837	**7706,821**	0,470929	UNCHANGED	0,926717	UNCHANGED	1,967849
172,961	89,85205	**123,7009**	1,308155	UNCHANGED	2,301854	UNCHANGED	1,759619
5539,982	4426,657	**4696,58**	1,402444	UNCHANGED	1,651345	UNCHANGED	1,177476
4237,073	4664,787	**4505,014**	1,769636	UNCHANGED	0,33143	UNCHANGED	0,187287
531,8009	687,9729	**595,1891**	0,381404	UNCHANGED	0,310568	UNCHANGED	0,814274
64,054	183,6611	**164,8372**	0,532495	UNCHANGED	1,873273	UNCHANGED	3,517915
102690,3	111954,9	**110658,8**	0,886594	UNCHANGED	1,123279	UNCHANGED	1,26696
10486,3	9256,637	**9775,411**	1,086858	UNCHANGED	1,245714	UNCHANGED	1,14616
14658,54	18582,54	**17200,73**	0,866223	UNCHANGED	1,100919	UNCHANGED	1,270941
382,1629	230,7915	**255,257**	0,092107	UNCHANGED	0,925354	UNCHANGED	10,04652
4870,411	3727,867	**4104,218**	0,824485	UNCHANGED	1,427915	UNCHANGED	1,731887
6753,066	7043,034	**6855,937**	0,706901	UNCHANGED	0,564463	UNCHANGED	0,798503
111076,2	100895,5	**105048,2**	0,881668	UNCHANGED	1,208993	UNCHANGED	1,371256
6941,64	9037,647	**8583,313**	0,491674	UNCHANGED	0,773742	UNCHANGED	1,573689
1183,17	899,2379	**991,4717**	0,947948	UNCHANGED	1,763856	UNCHANGED	1,86071
29782,65	32010,5	**32210,88**	0,72397	UNCHANGED	0,809422	UNCHANGED	1,118033
2221,711	2127,473	**2102,526**	0,696217	UNCHANGED	0,556505	UNCHANGED	0,799326
437,8152	506,1713	**447,6748**	0,665236	UNCHANGED	0,470417	UNCHANGED	0,707143
1802,055	2877,451	**2286,458**	0,793266	UNCHANGED	0,631997	UNCHANGED	0,796702
9577,582	18649,29	**15217,46**	0,455188	UNCHANGED	0,441746	UNCHANGED	0,970471
329,3345	280,783	**276,8709**	0,725131	UNCHANGED	0,614255	UNCHANGED	0,847095
1367,493	1459,427	**1476,282**	0,731315	UNCHANGED	1,337397	UNCHANGED	1,828756
285,5558	392,5649	**316,9154**	0,472125	UNCHANGED	0,24072	UNCHANGED	0,509864
1036,654	625,825	**845,6526**	1,099428	UNCHANGED	1,940999	UNCHANGED	1,765462
2293,52	2679,623	**2283,243**	0,726987	UNCHANGED	1,365658	UNCHANGED	1,878519
15080,28	13935,93	**13992,14**	1,436822	UNCHANGED	1,499049	UNCHANGED	1,043309
711,1612	862,753	**828,9671**	0,810648	UNCHANGED	0,497096	UNCHANGED	0,613208
18557,18	18281,13	**18355,08**	0,854897	UNCHANGED	0,737906	UNCHANGED	0,863152
3144,86	2687,867	**2970,057**	0,873022	UNCHANGED	0,676834	UNCHANGED	0,775277
4578,981	4891,963	**4931,279**	0,654181	UNCHANGED	0,869095	UNCHANGED	1,328523
10665,06	13138,23	**12180,59**	0,755583	UNCHANGED	1,087093	UNCHANGED	1,438748
554,2715	296,9646	**365,004**	1,747718	UNCHANGED	1,788231	UNCHANGED	1,02318
15294,96	16988,79	**16258,55**	0,845152	UNCHANGED	1,138275	UNCHANGED	1,346829
19674,66	16354,2	**17974,95**	0,815035	UNCHANGED	1,090562	UNCHANGED	1,338055
8317,046	7388,995	**7626,234**	1,132941	UNCHANGED	1,591231	UNCHANGED	1,404513
7081,998	8092,648	**7501,43**	1,162644	UNCHANGED	1,52785	UNCHANGED	1,314117
130,6596	334,9395	**216,2299**	0,788179	UNCHANGED	2,097832	UNCHANGED	2,661617
32963,24	25019,22	**27312,96**	0,792156	UNCHANGED	1,111104	UNCHANGED	1,402633
11507,25	12018,56	**12208,84**	1,024996	UNCHANGED	1,19583	UNCHANGED	1,166668
21866,17	23057,88	**25155,47**	0,931891	UNCHANGED	1,472911	UNCHANGED	1,580561
2981,151	2396,302	**2488,823**	0,905307	UNCHANGED	0,702441	UNCHANGED	0,775914

69,97931	205,3525	**112,7001**	23,69523	UNCHANGED	4,866465	UNCHANGED	0,205377
3352,88	1147,473	**1917,363**	5,256158	UNCHANGED	0,518274	UNCHANGED	0,098603
20148,84	10948,4	**14025,63**	1,5104	UNCHANGED	1,553937	UNCHANGED	1,028825
584,0478	515,6084	**535,6801**	0,574166	UNCHANGED	0,20031	UNCHANGED	0,348872
1443,223	272,5809	**668,2856**	0,525247	UNCHANGED	2,652742	UNCHANGED	5,05047
0,340764	22,45446	**12,95166**	0,261321	UNCHANGED	4,247463	UNCHANGED	16,25382
1686,9	1820,496	**1727,817**	0,594643	UNCHANGED	0,778081	UNCHANGED	1,308484
43072,92	56829,04	**52146,24**	0,658812	UNCHANGED	0,513453	UNCHANGED	0,779362
9326,708	8599,04	**8974,859**	1,164486	UNCHANGED	1,543769	UNCHANGED	1,325709
972,7679	1154,948	**1162,589**	1,325111	UNCHANGED	1,588333	UNCHANGED	1,198641
53033,43	76211,12	**69779,54**	0,849833	UNCHANGED	1,205412	UNCHANGED	1,418409
11371,28	9952,512	**10355,68**	1,11754	UNCHANGED	1,546024	UNCHANGED	1,383417
21239,65	18384,19	**19596,39**	0,970267	UNCHANGED	1,436076	UNCHANGED	1,480083
1033,402	1226,868	**1046,738**	1,008791	UNCHANGED	1,419838	UNCHANGED	1,407465
4995,328	3714,459	**3971,444**	0,727486	UNCHANGED	0,655926	UNCHANGED	0,901633
9672,475	11609,89	**11074,7**	0,742627	UNCHANGED	0,699742	UNCHANGED	0,942252
76258,93	89167,99	**85833,65**	0,891665	UNCHANGED	1,17871	UNCHANGED	1,321921
634,2746	629,1026	**643,1266**	0,890734	UNCHANGED	0,245877	UNCHANGED	0,276039
3841,767	4320,258	**4119,817**	0,998277	UNCHANGED	0,773434	UNCHANGED	0,774769
103745,7	173566,8	**146019,6**	0,775923	UNCHANGED	0,641078	UNCHANGED	0,826213
707,6449	257,9996	**422,7744**	2,308758	UNCHANGED	3,470683	UNCHANGED	1,503268
6143,51	6136,274	**6138,033**	0,906211	UNCHANGED	1,279637	UNCHANGED	1,412074
9795,142	5516,452	**6988,011**	0,606718	UNCHANGED	1,341846	UNCHANGED	2,211649
1006,365	896,1988	**792,4152**	1,192022	UNCHANGED	2,671143	UNCHANGED	2,24085
9378,319	9108,782	**9606,2**	0,699115	UNCHANGED	0,777165	UNCHANGED	1,111641
1457,244	1743,704	**1556,597**	1,171552	UNCHANGED	0,831424	UNCHANGED	0,709678
7595,386	6402,114	**6759,272**	1,390133	UNCHANGED	1,355072	UNCHANGED	0,974778
340537,3	321549	**323942,2**	0,947952	UNCHANGED	1,172196	UNCHANGED	1,236557
10473,42	9004,175	**9910,045**	1,259568	UNCHANGED	1,724498	UNCHANGED	1,369118
22300,6	25905,59	**24759,22**	0,9538	UNCHANGED	0,791109	UNCHANGED	0,829429
100778,9	113196,3	**110144,1**	0,711138	UNCHANGED	0,899799	UNCHANGED	1,265295
759,6649	874,6121	**793,1004**	1,310919	UNCHANGED	0,612579	UNCHANGED	0,467289
3277,086	2804,616	**3120,721**	1,417194	UNCHANGED	1,818805	UNCHANGED	1,283385
78840,48	80143,05	**78940,63**	1,064735	UNCHANGED	1,232173	UNCHANGED	1,157257
4832,643	3680,013	**4192,625**	1,218311	UNCHANGED	1,191761	UNCHANGED	0,978208
21204,5	9135,839	**13229,63**	0,548168	UNCHANGED	1,329836	UNCHANGED	2,425961
92,32345	115,0294	**104,792**	3,65264	UNCHANGED	1,788934	UNCHANGED	0,489765
1522,217	1415,136	**1560,539**	0,644402	UNCHANGED	0,780211	UNCHANGED	1,210752
5036,9	2845,105	**5000,208**	1,395133	UNCHANGED	4,323727	UNCHANGED	3,099151
18303,39	15610,92	**16440,43**	0,976733	UNCHANGED	0,73521	UNCHANGED	0,752724
23320,89	26150,49	**24997,81**	0,786691	UNCHANGED	1,085442	UNCHANGED	1,379757
36937,79	25462,78	**29788,22**	0,996067	UNCHANGED	1,668365	UNCHANGED	1,674953
85731,67	87431,86	**86692,7**	0,812525	UNCHANGED	0,858955	UNCHANGED	1,057143
2521,982	2074,275	**2230,079**	1,400687	UNCHANGED	1,10296	UNCHANGED	0,787442
298,5716	218,46	**274,6469**	0,786925	UNCHANGED	2,555686	UNCHANGED	3,247685
9273,791	10048,01	**9795,347**	0,739154	UNCHANGED	0,701669	UNCHANGED	0,949286
4329,839	4210,533	**4391,362**	0,897896	UNCHANGED	0,617993	UNCHANGED	0,688268
4934,889	4525,643	**4607,996**	0,993923	UNCHANGED	1,38264	UNCHANGED	1,391094
11871,95	10069,28	**11355,7**	0,160328	UNCHANGED	0,221265	UNCHANGED	1,380079
3808,31	4227,855	**4109,772**	1,844043	UNCHANGED	0,92356	UNCHANGED	0,500834

1085,654	1159,391	**1079,508**	0,538551	UNCHANGED	0,254958	UNCHANGED	0,473415
5838,035	5048,733	**5413,312**	0,986729	UNCHANGED	0,617949	UNCHANGED	0,62626
156,2401	234,9272	**204,3354**	0,873437	UNCHANGED	1,797842	UNCHANGED	2,058353
69512,1	39386,7	**49955,05**	0,548282	UNCHANGED	1,012551	UNCHANGED	1,846769
22871,7	21581,5	**22493,22**	1,348194	UNCHANGED	1,209207	UNCHANGED	0,896909
1146,661	1103,401	**1129,641**	0,525996	UNCHANGED	0,342244	UNCHANGED	0,65066
190,2732	508,0541	**353,9027**	1,393833	UNCHANGED	2,086378	UNCHANGED	1,496864
27799,52	34822,32	**31943,3**	0,77784	UNCHANGED	0,820259	UNCHANGED	1,054534
36468,19	38320,42	**37946,71**	1,131106	UNCHANGED	1,27617	UNCHANGED	1,12825
9487,931	8146,935	**8682,443**	0,873191	UNCHANGED	0,854321	UNCHANGED	0,978389
2439,554	1505,139	**1763,15**	1,421505	UNCHANGED	2,073533	UNCHANGED	1,458688
2382,843	2135,433	**2208,305**	0,767824	UNCHANGED	0,527081	UNCHANGED	0,686461
29116,97	25923,67	**26911,37**	0,711092	UNCHANGED	0,854149	UNCHANGED	1,201179
5468,025	6081,924	**5614,19**	0,824519	UNCHANGED	0,719714	UNCHANGED	0,872889
2024,984	2327,695	**2105,062**	0,68403	UNCHANGED	0,768599	UNCHANGED	1,123632
159487,8	163902,1	**163854,2**	0,96716	UNCHANGED	1,056912	UNCHANGED	1,0928
36462,57	37202,77	**37448,04**	0,790392	UNCHANGED	0,834313	UNCHANGED	1,055569
97,93399	44,25847	**60,01963**	1,581239	UNCHANGED	2,220697	UNCHANGED	1,404403
465,5491	732,2756	**658,1447**	0,427515	UNCHANGED	0,269615	UNCHANGED	0,630656
124583	122370	**123174,2**	1,192102	UNCHANGED	1,318397	UNCHANGED	1,105943
155,649	198,4442	**165,3266**	2,081386	UNCHANGED	3,711867	UNCHANGED	1,783363
11663,86	7547,777	**9194,334**	0,560978	UNCHANGED	1,031898	UNCHANGED	1,839462
17910,48	17398,04	**17631,88**	0,812942	UNCHANGED	0,920561	UNCHANGED	1,132382
14555,21	13987,63	**13823,08**	0,879203	UNCHANGED	0,958006	UNCHANGED	1,08963
3025,283	4214,039	**3755,337**	0,769466	UNCHANGED	0,709273	UNCHANGED	0,921773
694,6228	18,15556	**285,7617**	0,917729	UNCHANGED	3,398147	UNCHANGED	3,702779
596,7294	693,4371	**688,6281**	0,314612	UNCHANGED	0,309746	UNCHANGED	0,984533
2179,311	1748,494	**1850,885**	1,945069	UNCHANGED	3,508852	UNCHANGED	1,803973
653,0702	588,5326	**616,4812**	1,229389	UNCHANGED	1,463221	UNCHANGED	1,190202
3408,215	4810,967	**3948,387**	0,676652	UNCHANGED	0,869077	UNCHANGED	1,284379
7513,145	7544,465	**7540,079**	1,00519	UNCHANGED	1,420676	UNCHANGED	1,413341
8389,105	5715,755	**6581**	1,103687	UNCHANGED	0,677855	UNCHANGED	0,614174
3611,508	3064,892	**3396,737**	0,704685	UNCHANGED	0,573648	UNCHANGED	0,814049
5564,344	4591,491	**4890,804**	1,102762	UNCHANGED	0,553849	UNCHANGED	0,502238
152223,3	112948,1	**129651,1**	0,971552	UNCHANGED	0,709178	UNCHANGED	0,729943
274925,9	258970,3	**266351,7**	0,929526	UNCHANGED	0,815668	UNCHANGED	0,877509
6131,94	4848,705	**4950,553**	1,450111	UNCHANGED	1,311824	UNCHANGED	0,904637
14286,77	15922,36	**15750,13**	1,174906	UNCHANGED	1,235037	UNCHANGED	1,05118
1040,953	946,0508	**1004,012**	0,910757	UNCHANGED	0,582482	UNCHANGED	0,639558
4165,761	3962,348	**3981,417**	1,316651	UNCHANGED	1,327186	UNCHANGED	1,008001
12002,01	9399,2	**10159,73**	0,83023	UNCHANGED	0,781561	UNCHANGED	0,941379
38446,18	32206,93	**34747,61**	0,750793	UNCHANGED	0,995341	UNCHANGED	1,32572
2778,705	3770,946	**3349,022**	0,886275	UNCHANGED	1,38075	UNCHANGED	1,557925
283,5305	40,32862	**245,1891**	1,335364	UNCHANGED	4,754794	UNCHANGED	3,560673
34479,27	40697,75	**38365,03**	1,172391	UNCHANGED	0,965593	UNCHANGED	0,82361
193,0337	102,3557	**138,5306**	3,085072	UNCHANGED	4,61878	UNCHANGED	1,497138
10704,8	8452,276	**9491,2**	1,050643	UNCHANGED	1,323159	UNCHANGED	1,25938
669,9472	682,4213	**673,4573**	1,223679	UNCHANGED	0,902424	UNCHANGED	0,737468
4363,07	3780,914	**3832,541**	1,428788	UNCHANGED	1,064582	UNCHANGED	0,745094
9381,453	9136,328	**8998,061**	1,40077	UNCHANGED	1,459574	UNCHANGED	1,04198

307,177	192,1068	**213,8693**	0,981597	UNCHANGED	8,381779	UNCHANGED	8,538921
6313,369	7631,708	**7074,374**	1,385477	UNCHANGED	1,211608	UNCHANGED	0,874506
1420,18	1070,59	**1071,022**	0,713417	UNCHANGED	1,110966	UNCHANGED	1,557247
524,7557	550,1255	**598,4324**	1,77856	UNCHANGED	2,002579	UNCHANGED	1,125955
4799,403	2693,328	**3389,249**	0,653064	UNCHANGED	0,748716	UNCHANGED	1,146467
14558,5	13054,84	**13373,35**	1,056677	UNCHANGED	1,297763	UNCHANGED	1,228155
2014,273	2254,655	**2191,66**	0,729488	UNCHANGED	0,480752	UNCHANGED	0,659027
58,03362	3,328368	**25,70226**	3,089455	UNCHANGED	11,93636	UNCHANGED	3,863582
20250,56	24038,05	**22426,88**	1,067159	UNCHANGED	1,213552	UNCHANGED	1,13718
154,0784	115,726	**129,4339**	1,601782	UNCHANGED	0,316731	UNCHANGED	0,197737
3220,375	3424,685	**3310,698**	0,746611	UNCHANGED	0,625721	UNCHANGED	0,838082
8354,165	7614,571	**7689,151**	0,662439	UNCHANGED	0,702569	UNCHANGED	1,060579
607,7522	474,5455	**545,9519**	1,407524	UNCHANGED	1,012037	UNCHANGED	0,719019
6042,267	5898,298	**5937,681**	0,817717	UNCHANGED	0,681168	UNCHANGED	0,833012
442,3018	531,2436	**533,117**	2,034224	UNCHANGED	1,053847	UNCHANGED	0,518058
1139,691	808,3261	**852,4866**	2,317052	UNCHANGED	5,48959	UNCHANGED	2,369213
3731,673	3843,462	**3914,764**	1,091761	UNCHANGED	1,392632	UNCHANGED	1,275582
23771,49	17529,8	**19468,73**	1,218247	UNCHANGED	1,383761	UNCHANGED	1,135863
9797,462	8946,876	**9346,222**	1,275001	UNCHANGED	0,669028	UNCHANGED	0,524728
5393,512	5826,046	**5613,538**	1,277478	UNCHANGED	1,002441	UNCHANGED	0,784703
20819,43	15957,56	**17360,42**	1,211956	UNCHANGED	1,286428	UNCHANGED	1,061447
25337,53	28655,14	**27604,12**	0,949193	UNCHANGED	1,151103	UNCHANGED	1,212718
1913,863	467,8615	**979,3575**	0,28499	UNCHANGED	0,743215	UNCHANGED	2,607866
3520,528	3236,173	**3729,98**	0,577561	UNCHANGED	0,713121	UNCHANGED	1,234711
9346,88	12098,53	**11211,47**	0,980043	UNCHANGED	1,17681	UNCHANGED	1,200774
169232,9	160046,2	**164974,2**	0,829514	UNCHANGED	0,984856	UNCHANGED	1,187269
2886,173	4904,433	**4218,159**	0,613948	UNCHANGED	0,536567	UNCHANGED	0,873962
936,9523	1296,355	**1178,942**	1,177121	UNCHANGED	0,7573	UNCHANGED	0,64335
1409,413	1053,42	**1209,517**	0,845615	UNCHANGED	1,173179	UNCHANGED	1,387368
3652,93	3195,94	**3162,017**	1,058953	UNCHANGED	1,370333	UNCHANGED	1,294045
77523,95	72818,59	**72887,45**	1,161531	UNCHANGED	0,940302	UNCHANGED	0,809537
27779,23	29464,55	**28284,39**	0,940937	UNCHANGED	1,173467	UNCHANGED	1,247126
1782,315	2356,26	**2111,45**	0,600055	UNCHANGED	0,641969	UNCHANGED	1,069851
88133,85	30980,96	**50962,16**	0,92393	UNCHANGED	1,660904	UNCHANGED	1,797651
53566,58	67488,9	**62766,34**	0,72969	UNCHANGED	0,636642	UNCHANGED	0,872484
880,0712	500,4654	**633,75**	1,474675	UNCHANGED	1,758409	UNCHANGED	1,192404
925,4406	970,2129	**951,5226**	1,169852	UNCHANGED	3,247078	UNCHANGED	2,775632
2081,496	2577,525	**2513,161**	1,423522	UNCHANGED	1,045557	UNCHANGED	0,734486
28587,88	28934,34	**29536,36**	0,947831	UNCHANGED	1,15957	UNCHANGED	1,223393
80,23372	40,33991	**45,74469**	0,90345	UNCHANGED	2,334921	UNCHANGED	2,58445
69872,62	80888,13	**76055,01**	0,800198	UNCHANGED	0,688627	UNCHANGED	0,86057
19693,49	20213,1	**20256,87**	0,780644	UNCHANGED	1,056545	UNCHANGED	1,353427
8767,412	7771,777	**7900,991**	1,752583	UNCHANGED	1,898135	UNCHANGED	1,08305
366697,4	358578,6	**379654,8**	0,772018	UNCHANGED	1,166373	UNCHANGED	1,510809
715,6797	532,8513	**596,9865**	2,290072	UNCHANGED	1,323377	UNCHANGED	0,577876
1208,777	1119,735	**1229,24**	1,518365	UNCHANGED	2,219252	UNCHANGED	1,461606
143256,1	162669,2	**154640,7**	0,84658	UNCHANGED	0,848998	UNCHANGED	1,002856
171076,9	155617,4	**166175,8**	1,041198	UNCHANGED	1,32124	UNCHANGED	1,268961
137,784	189,1614	**174,4158**	0,651584	UNCHANGED	0,396856	UNCHANGED	0,609063
1433,096	1693,362	**1727,382**	0,475788	UNCHANGED	0,586469	UNCHANGED	1,232626

91805,48	97442,57	**95962,49**	0,824032	UNCHANGED	0,674402	UNCHANGED	0,818418
180401	213974,7	**200635,1**	0,811887	UNCHANGED	1,04943	UNCHANGED	1,292582
32893,72	70488,55	**55268,05**	0,512335	UNCHANGED	1,171127	UNCHANGED	2,285861
556,0264	648,9477	**550,1767**	1,79685	UNCHANGED	1,705152	UNCHANGED	0,948967
4473,575	5484,311	**5253,523**	0,676873	UNCHANGED	0,80678	UNCHANGED	1,191922
20481,19	20093,6	**20532,38**	1,075695	UNCHANGED	0,92264	UNCHANGED	0,857715
9629,414	8675,699	**8880,051**	0,774431	UNCHANGED	0,817023	UNCHANGED	1,054998
1998,09	9748,147	**7316,493**	0,321051	UNCHANGED	0,40939	UNCHANGED	1,275157
665,2055	1471,211	**1162,018**	0,342116	UNCHANGED	0,713279	UNCHANGED	2,084903
11276,04	12421,03	**11790,32**	1,034734	UNCHANGED	0,854641	UNCHANGED	0,825952
2242,511	2935,717	**3155,89**	0,820675	UNCHANGED	0,498286	UNCHANGED	0,607167
2932,161	301,6869	**1184,812**	0,275445	UNCHANGED	0,815449	UNCHANGED	2,96048
980,1467	723,4415	**849,8599**	0,929275	UNCHANGED	0,69428	UNCHANGED	0,74712
10154,7	11600,32	**10536,03**	1,407196	UNCHANGED	1,468653	UNCHANGED	1,043673
20254,69	16654,92	**17645,55**	1,27069	UNCHANGED	1,292196	UNCHANGED	1,016925
3789,214	3979,952	**3857,039**	0,81497	UNCHANGED	0,697918	UNCHANGED	0,856372
134,818	560,6267	**366,345**	1,765244	UNCHANGED	0,452388	UNCHANGED	0,256275
1817,758	1745,917	**1706,474**	1,342176	UNCHANGED	1,078877	UNCHANGED	0,803827
11300,49	13224,45	**12890,59**	0,952422	UNCHANGED	0,82911	UNCHANGED	0,870528
1823,645	2285,631	**2083,362**	1,037522	UNCHANGED	1,313404	UNCHANGED	1,265905
2569,718	2058,229	**2255,069**	1,28156	UNCHANGED	1,345426	UNCHANGED	1,049835
13767,55	19747,57	**17708,15**	1,872432	UNCHANGED	1,157957	UNCHANGED	0,618424
96,21762	112,0968	**95,54065**	0,221526	UNCHANGED	0,474826	UNCHANGED	2,143429
52575,91	50968,32	**50974,21**	0,694058	UNCHANGED	0,73275	UNCHANGED	1,055748
22044,56	26142,83	**25535,77**	1,071185	UNCHANGED	1,4826	UNCHANGED	1,384074
10759,17	8908,15	**9845,664**	0,829681	UNCHANGED	1,32147	UNCHANGED	1,592745
679,1468	809,7553	**579,1524**	0,51664	UNCHANGED	1,107396	UNCHANGED	2,143457
3274,664	3655,06	**3410,837**	1,12523	UNCHANGED	1,059425	UNCHANGED	0,941518
47382,42	30198,2	**36950,79**	1,073749	UNCHANGED	0,578868	UNCHANGED	0,539109
6502,548	5857,038	**6149,262**	0,679115	UNCHANGED	0,444347	UNCHANGED	0,654303
14204,18	13133,21	**13355,56**	1,378996	UNCHANGED	1,251957	UNCHANGED	0,907876
20746,12	16637,34	**18338,98**	0,869812	UNCHANGED	0,782357	UNCHANGED	0,899456
14930,17	16072,04	**15649,67**	1,22451	UNCHANGED	1,359577	UNCHANGED	1,110303
8925,407	8211,688	**8309,316**	0,99865	UNCHANGED	1,461472	UNCHANGED	1,463449
5565,915	5399,558	**5423,352**	0,85936	UNCHANGED	1,138626	UNCHANGED	1,32497
212,0616	387,9821	**343,611**	0,297642	UNCHANGED	0,578805	UNCHANGED	1,944639
1428,416	1626,335	**1591,682**	1,118049	UNCHANGED	0,834365	UNCHANGED	0,746269
37888,74	37160,35	**37731,64**	0,812956	UNCHANGED	0,770747	UNCHANGED	0,948079
1360,493	598,6425	**855,5185**	1,382187	UNCHANGED	0,688072	UNCHANGED	0,497814
2274,656	1152,047	**1644,845**	0,858245	UNCHANGED	0,568935	UNCHANGED	0,662905
1165,625	1359,66	**1264,695**	0,421695	UNCHANGED	0,375114	UNCHANGED	0,88954
8360,218	8882,937	**8692,532**	1,029195	UNCHANGED	1,24214	UNCHANGED	1,206904
3488,36	1547,754	**2180,886**	0,692144	UNCHANGED	0,365587	UNCHANGED	0,528195
11340,06	12426,72	**12269,98**	1,109589	UNCHANGED	1,086004	UNCHANGED	0,978744
2533,384	2125,217	**2181,948**	1,294022	UNCHANGED	2,121025	UNCHANGED	1,639095
34985,02	36455,71	**36851,19**	0,674454	UNCHANGED	0,726839	UNCHANGED	1,07767
674,0561	675,7139	**683,1739**	0,532171	UNCHANGED	0,591903	UNCHANGED	1,112243
4495,244	5308,343	**5052,437**	1,163216	UNCHANGED	1,2823	UNCHANGED	1,102375
63454,53	71460,21	**68290,59**	0,951014	UNCHANGED	0,830634	UNCHANGED	0,873419
45,24649	12,71984	**22,31963**	1,864274	UNCHANGED	8,801656	UNCHANGED	4,721226

12368,85	16160,34	**15674,17**	1,059383	UNCHANGED	0,59834	UNCHANGED	0,5648
10339,69	11334,49	**11106,38**	0,851119	UNCHANGED	1,001105	UNCHANGED	1,176222
36677,93	32031,72	**34473,66**	0,810786	UNCHANGED	0,715079	UNCHANGED	0,881958
75105,23	83543,87	**84879,65**	0,909846	UNCHANGED	1,072913	UNCHANGED	1,179225
3502,662	3619,734	**3522,759**	0,710426	UNCHANGED	0,806879	UNCHANGED	1,135768
65448,64	75429,48	**69830,34**	0,862414	UNCHANGED	0,7951	UNCHANGED	0,921948
132591,1	147844,3	**141989,5**	0,886929	UNCHANGED	0,840345	UNCHANGED	0,947477
14589,17	17139,35	**16306,48**	1,058073	UNCHANGED	0,823503	UNCHANGED	0,778305
312,2083	159,9563	**201,1002**	0,392371	UNCHANGED	0,781855	UNCHANGED	1,992642
34852,96	38383,32	**36639,4**	0,838656	UNCHANGED	1,103362	UNCHANGED	1,31563
14162,37	10785,5	**12025,29**	0,935724	UNCHANGED	1,222953	UNCHANGED	1,306959
137,7653	230,5457	**195,5675**	0,427719	UNCHANGED	1,494805	UNCHANGED	3,494831
10167,19	14312,26	**12864,64**	0,896184	UNCHANGED	1,320817	UNCHANGED	1,473823
6704,765	8601,857	**7860,239**	1,297662	UNCHANGED	1,462951	UNCHANGED	1,127374
8114,73	8377,69	**8330,89**	2,177809	UNCHANGED	2,303054	UNCHANGED	1,05751
843,4815	2374,397	**1691,951**	0,53581	UNCHANGED	0,886935	UNCHANGED	1,655317
2644,606	3931,555	**3361,982**	1,255314	UNCHANGED	0,732094	UNCHANGED	0,583196
3513,984	3662,294	**3643,837**	1,36606	UNCHANGED	1,42297	UNCHANGED	1,04166
507,4948	568,2887	**505,5241**	1,242241	UNCHANGED	0,899804	UNCHANGED	0,724339
20364,34	13716,35	**16006,01**	1,095316	UNCHANGED	1,480304	UNCHANGED	1,351485
1751,485	2016,129	**1750,19**	0,623379	UNCHANGED	0,734548	UNCHANGED	1,178332
65189,73	66136,53	**66289,88**	0,868991	UNCHANGED	0,817686	UNCHANGED	0,94096
52858,18	59159,26	**57101,85**	0,848939	UNCHANGED	0,83147	UNCHANGED	0,979423
70027,39	94853,96	**85957,81**	0,950752	UNCHANGED	0,76701	UNCHANGED	0,80674
3668,959	3341,548	**3481,644**	0,953896	UNCHANGED	1,32582	UNCHANGED	1,3899
4467,736	5801,747	**5339,452**	0,961489	UNCHANGED	0,814628	UNCHANGED	0,847257
679,1659	648,5896	**637,7136**	2,548677	UNCHANGED	2,049031	UNCHANGED	0,803958
13494,55	14276,21	**14116,83**	1,131562	UNCHANGED	1,090574	UNCHANGED	0,963777
120,4349	89,23279	**104,2997**	1,506543	UNCHANGED	1,312033	UNCHANGED	0,87089
20435,46	22823,32	**22105,77**	1,083362	UNCHANGED	0,815449	UNCHANGED	0,752702
5277,805	4974,6	**4895,164**	0,983458	UNCHANGED	0,794601	UNCHANGED	0,807966
1558,835	1861,507	**1719,798**	1,131751	UNCHANGED	1,294588	UNCHANGED	1,143881
6894,892	7752,945	**6991,623**	1,097822	UNCHANGED	1,285082	UNCHANGED	1,170574
680,4612	1033,723	**873,9842**	0,928453	UNCHANGED	1,258239	UNCHANGED	1,3552
23208,56	24872,66	**23933,09**	0,894044	UNCHANGED	0,701639	UNCHANGED	0,784792
470,1321	776,3622	**613,6274**	0,262264	UNCHANGED	0,533234	UNCHANGED	2,033196
36190,4	17581,49	**23653,93**	0,835875	UNCHANGED	1,305799	UNCHANGED	1,562194
913,9753	648,9311	**807,0326**	1,557693	UNCHANGED	0,832303	UNCHANGED	0,534318
10101,6	8273,831	**8607,786**	0,851892	UNCHANGED	0,745957	UNCHANGED	0,875647
241,3457	252,7008	**249,964**	0,601289	UNCHANGED	0,298014	UNCHANGED	0,495626
1434,035	1492,166	**1427,342**	2,591859	UNCHANGED	1,234354	UNCHANGED	0,476243
29942,46	31704,07	**31224,81**	0,912711	UNCHANGED	0,899063	UNCHANGED	0,985047
17724,59	20430,92	**19482,75**	1,141933	UNCHANGED	1,126841	UNCHANGED	0,986784
4748,333	4863,837	**4717,426**	0,960237	UNCHANGED	0,761617	UNCHANGED	0,793155
37,93601	148,359	**108,6812**	0,461263	UNCHANGED	1,083284	UNCHANGED	2,348516
12134,9	10742,43	**11200,73**	0,826134	UNCHANGED	0,995443	UNCHANGED	1,204943
538,7134	712,7872	**682,3144**	2,228235	UNCHANGED	3,101815	UNCHANGED	1,39205
4993,092	5396,625	**5374,664**	1,641106	UNCHANGED	1,519325	UNCHANGED	0,925794
371,0413	406,0637	**378,2604**	1,413587	UNCHANGED	2,556273	UNCHANGED	1,808359
5430,06	5649,798	**5573,158**	1,292546	UNCHANGED	1,260078	UNCHANGED	0,974881

46,83259	166,243	**107,6135**	0,374041	UNCHANGED	0,317078	UNCHANGED	0,84771
12973,65	17339	**15617,48**	0,944351	UNCHANGED	1,28239	UNCHANGED	1,357959
2455,757	2541,025	**2339,004**	0,844055	UNCHANGED	0,707101	UNCHANGED	0,837743
255,5105	58,73708	**119,1928**	0,109131	UNCHANGED	1,343594	UNCHANGED	12,31174
34,40113	3,894318	**39,75097**	1,121024	UNCHANGED	3,392468	UNCHANGED	3,026221
14929,06	12536,06	**12845,05**	0,991831	UNCHANGED	1,255561	UNCHANGED	1,265902
2011,471	1180,611	**1550,39**	0,656474	UNCHANGED	0,739462	UNCHANGED	1,126415
13341,12	11648,16	**12564,66**	1,129613	UNCHANGED	1,509626	UNCHANGED	1,33641
14563,44	11343,17	**12174,8**	0,940725	UNCHANGED	1,39513	UNCHANGED	1,483036
3485,043	1655,081	**5505,424**	0,223644	UNCHANGED	0,340289	UNCHANGED	1,521568
6559,732	6526,562	**5796,943**	0,93992	UNCHANGED	0,741623	UNCHANGED	0,789027
20465,98	23048,66	**23527,38**	1,562507	UNCHANGED	1,713121	UNCHANGED	1,096392
16068,58	15743,58	**15848,01**	0,796628	UNCHANGED	0,641319	UNCHANGED	0,805042
1225,734	1572,349	**1430,455**	0,702864	UNCHANGED	0,410329	UNCHANGED	0,583796
38353,3	44019,61	**42572,69**	0,973584	UNCHANGED	1,180711	UNCHANGED	1,212747
11587,32	10205,69	**10721,62**	0,702936	UNCHANGED	0,644707	UNCHANGED	0,917164
3336,666	3430,271	**3416,821**	1,332722	UNCHANGED	1,266708	UNCHANGED	0,950467
3131,235	3502,177	**3324,052**	0,538762	UNCHANGED	0,274311	UNCHANGED	0,50915
1806,298	1878,611	**3588,261**	0,786426	UNCHANGED	1,796279	UNCHANGED	2,284104
1416,84	1459,258	**1445,078**	1,416321	UNCHANGED	1,130075	UNCHANGED	0,797895
15421,4	14917,62	**15320,7**	0,886569	UNCHANGED	0,888712	UNCHANGED	1,002417
1573,271	1472,107	**1539,904**	0,783592	UNCHANGED	0,739881	UNCHANGED	0,944217
100393,8	68935,06	**81525,37**	1,062086	UNCHANGED	1,223317	UNCHANGED	1,151806
2213,81	2528,087	**2321,414**	0,861162	UNCHANGED	0,700786	UNCHANGED	0,813769
7250,173	7122,144	**7237,609**	0,827894	UNCHANGED	0,789626	UNCHANGED	0,953776
47537,92	57978,49	**54543,05**	0,789825	UNCHANGED	1,023079	UNCHANGED	1,295323
10653,54	7987,715	**9398,709**	0,70534	UNCHANGED	0,913607	UNCHANGED	1,295272
14450,66	23740,32	**21000,03**	0,61595	UNCHANGED	0,644404	UNCHANGED	1,046195
2794,291	4141,008	**3305,387**	0,833515	UNCHANGED	1,129193	UNCHANGED	1,354737
30783,41	25259,1	**26292,74**	1,167109	UNCHANGED	1,019241	UNCHANGED	0,873305
381,9233	620,2833	**533,0795**	1,216577	UNCHANGED	1,954603	UNCHANGED	1,606642
118553,7	130372,1	**126519,6**	0,878828	UNCHANGED	0,844725	UNCHANGED	0,961195
44199,32	43386,62	**42480,23**	1,129871	UNCHANGED	0,789549	UNCHANGED	0,698796
4751,03	4451,726	**4488,727**	1,002855	UNCHANGED	1,347487	UNCHANGED	1,343651
25579,4	18392,8	**20899,27**	1,18273	UNCHANGED	1,316247	UNCHANGED	1,112888
11278,79	10876,46	**10991,92**	0,88136	UNCHANGED	0,68867	UNCHANGED	0,781373
9996,531	10474,01	**10591,19**	1,08839	UNCHANGED	1,155384	UNCHANGED	1,061553
69627,81	93246,18	**85009,43**	0,778026	UNCHANGED	0,65814	UNCHANGED	0,84591
1765,533	1956,349	**1930,46**	1,022613	UNCHANGED	0,818463	UNCHANGED	0,800365
14412,6	15301,27	**14797,03**	0,90468	UNCHANGED	1,123308	UNCHANGED	1,241663
1131,213	894,8948	**950,6525**	0,840227	UNCHANGED	0,73396	UNCHANGED	0,873525
8998,811	8947,116	**8988,498**	0,978352	UNCHANGED	0,78954	UNCHANGED	0,80701
270,1401	31,76117	**114,7851**	1,00814	UNCHANGED	1,99049	UNCHANGED	1,97442
42415,01	28716,02	**33513,18**	0,576176	UNCHANGED	1,01165	UNCHANGED	1,755799
3164,939	3060,062	**3047,456**	1,035048	UNCHANGED	1,152459	UNCHANGED	1,113435
848,3711	439,659	**542,2914**	0,681264	UNCHANGED	0,404309	UNCHANGED	0,593469
5635,093	4039,766	**4638,642**	1,241035	UNCHANGED	1,27983	UNCHANGED	1,031261
94,90274	142,2052	**122,5753**	0,454888	UNCHANGED	0,903616	UNCHANGED	1,98646
10124,07	15367,62	**13072,18**	0,684412	UNCHANGED	0,617877	UNCHANGED	0,902784
6322,994	5560,388	**5929,239**	1,074655	UNCHANGED	1,257073	UNCHANGED	1,169746

1219,931	1285,794	**1281,014**	0,938381	UNCHANGED	0,710272	UNCHANGED	0,756912
34127,8	43244,68	**40558,37**	0,748778	UNCHANGED	0,926909	UNCHANGED	1,237897
9923,574	16715,46	**14174,2**	0,664862	UNCHANGED	0,663933	UNCHANGED	0,998603
10370,21	12058,9	**11098,12**	0,701495	UNCHANGED	0,64539	UNCHANGED	0,920021
7606,367	5580,876	**6159,338**	1,532267	UNCHANGED	1,189047	UNCHANGED	0,776005
31975,48	34858,09	**34312,16**	0,961481	UNCHANGED	0,894824	UNCHANGED	0,930673
369,9233	300,3645	**315,7283**	0,654429	UNCHANGED	0,580528	UNCHANGED	0,887076
43446,42	52803,76	**49568,54**	0,677315	UNCHANGED	0,964142	UNCHANGED	1,423476
23368,19	24001,78	**23546,07**	0,886636	UNCHANGED	0,882463	UNCHANGED	0,995294
59533,98	32266,26	**39944,65**	1,341657	UNCHANGED	1,449035	UNCHANGED	1,080034
6218,489	6729,121	**6574,408**	0,854434	UNCHANGED	0,682369	UNCHANGED	0,798621
6647,45	7478,553	**7356,87**	0,604013	UNCHANGED	0,467771	UNCHANGED	0,774438
572,8363	427,247	**510,4874**	0,195025	UNCHANGED	2,124212	UNCHANGED	10,89197
3329,96	3591,861	**3446,164**	0,583838	UNCHANGED	0,778271	UNCHANGED	1,333026
2652,788	2293,271	**2368,494**	0,943898	UNCHANGED	1,195785	UNCHANGED	1,266858
92684,25	103052,5	**101455,8**	0,870312	UNCHANGED	0,921571	UNCHANGED	1,058896
1648,036	1822,28	**1755,852**	0,844144	UNCHANGED	0,825455	UNCHANGED	0,97786
2332,173	2698,201	**2793,976**	0,696362	UNCHANGED	0,742586	UNCHANGED	1,066379
4930,148	5076,344	**5007,344**	1,151419	UNCHANGED	1,25357	UNCHANGED	1,088718
1178,499	546,4178	**726,3328**	0,384953	UNCHANGED	0,224016	UNCHANGED	0,581931
1988,34	1330,777	**1626,684**	1,136752	UNCHANGED	1,83506	UNCHANGED	1,614301
2117,424	2003,472	**1937,756**	0,513898	UNCHANGED	1,66257	UNCHANGED	3,235215
1443,944	1892,703	**1739,574**	1,04323	UNCHANGED	0,721043	UNCHANGED	0,691164
410,7052	380,3117	**426,0408**	0,776814	UNCHANGED	0,4091	UNCHANGED	0,526638
370,3992	294,3345	**358,9733**	1,610778	UNCHANGED	1,554536	UNCHANGED	0,965084
5660,333	4427,806	**4782,342**	1,051586	UNCHANGED	1,230586	UNCHANGED	1,170218
19786,54	13611,71	**15653,37**	0,988425	UNCHANGED	1,350015	UNCHANGED	1,365825
32373,02	12802,06	**19604,22**	0,471633	UNCHANGED	0,727666	UNCHANGED	1,542866
627,9527	443,2074	**527,1878**	1,571756	UNCHANGED	0,717718	UNCHANGED	0,456635
2277,291	2330,381	**2203,018**	0,686661	UNCHANGED	0,801341	UNCHANGED	1,167011
3340,487	3632,999	**3604,552**	0,712142	UNCHANGED	0,820945	UNCHANGED	1,152782
516042,1	524524,9	**517881,2**	0,912989	UNCHANGED	1,107494	UNCHANGED	1,213042
7442,907	6848,814	**6857,969**	1,227636	UNCHANGED	1,083331	UNCHANGED	0,882453
5884,958	5325,362	**5688,159**	1,23388	UNCHANGED	0,965646	UNCHANGED	0,782609
89,90652	100,3902	**107,9823**	0,613695	UNCHANGED	3,158058	UNCHANGED	5,145969
7556,056	5410,409	**6225,039**	1,39017	UNCHANGED	1,905049	UNCHANGED	1,370371
6325,307	9100,684	**7588,606**	0,904416	UNCHANGED	0,779888	UNCHANGED	0,862311
3991,942	2220,058	**2840,723**	1,189568	UNCHANGED	1,639446	UNCHANGED	1,378186
2433,049	2419,673	**2548,613**	0,712886	UNCHANGED	0,800398	UNCHANGED	1,122758
4345,149	4701,469	**4403,456**	1,236728	UNCHANGED	1,266732	UNCHANGED	1,024261
463,1221	449,8432	**529,7077**	0,54252	UNCHANGED	1,890502	UNCHANGED	3,484665
974,8704	9,692597	**547,8807**	0,087349	UNCHANGED	0,054768	UNCHANGED	0,626999
8438,784	7957,659	**7856,161**	0,864203	UNCHANGED	1,122045	UNCHANGED	1,298358
7623,457	7697,811	**7662,682**	1,085542	UNCHANGED	0,824371	UNCHANGED	0,759409
7522,646	2448,889	**4152,531**	2,250084	UNCHANGED	1,201458	UNCHANGED	0,533961
12895,46	22539,5	**18979,04**	0,597557	UNCHANGED	0,92956	UNCHANGED	1,555599
1353,069	1561,814	**1390,551**	0,605835	UNCHANGED	0,87129	UNCHANGED	1,438164
14585,13	13886,2	**13800,01**	1,113843	UNCHANGED	1,167982	UNCHANGED	1,048605
13371,42	10167,24	**11237,17**	0,815767	UNCHANGED	0,870572	UNCHANGED	1,067182
8078,549	5301,606	**6562,519**	1,339517	UNCHANGED	1,303164	UNCHANGED	0,972861

2059,415	2714,939	**2497,739**	0,724535	UNCHANGED	0,652958	UNCHANGED	0,90121
49812,34	37425,15	**41712,39**	0,880062	UNCHANGED	0,839029	UNCHANGED	0,953375
34779,43	32718,38	**32925,68**	0,932347	UNCHANGED	0,821554	UNCHANGED	0,881168
182958,5	224017,2	**214041,4**	1,040706	UNCHANGED	0,7809	UNCHANGED	0,750356
27,78373	14,16328	**17,23669**	0,724826	UNCHANGED	2,79716	UNCHANGED	3,859079
68735,81	65362,75	**67541,44**	1,237874	UNCHANGED	0,977677	UNCHANGED	0,789803
2280,901	2285,105	**2358,508**	1,246563	UNCHANGED	1,32563	UNCHANGED	1,063427
12477,26	6087,146	**8331,852**	0,970206	UNCHANGED	2,779038	UNCHANGED	2,86438
15961,26	16081,51	**16134,64**	0,626505	UNCHANGED	0,7851	UNCHANGED	1,253142
4190,707	4435,237	**4375,954**	1,08474	UNCHANGED	1,158819	UNCHANGED	1,068292
221,3651	233,8055	**226,0535**	1,3412	UNCHANGED	1,747316	UNCHANGED	1,3028
2026,697	1867,823	**1841,797**	1,204697	UNCHANGED	0,833671	UNCHANGED	0,692017
7549,312	6447,953	**6923,532**	0,475785	UNCHANGED	0,446471	UNCHANGED	0,938388
870,3871	842,4308	**834,9789**	0,748905	UNCHANGED	1,40202	UNCHANGED	1,872093
9930,664	4989,59	**6696,238**	1,219031	UNCHANGED	1,715389	UNCHANGED	1,407174
54881,93	62562,14	**58787,85**	0,853701	UNCHANGED	1,020438	UNCHANGED	1,19531
1826,145	625,1022	**1632,178**	1,169691	UNCHANGED	2,523899	UNCHANGED	2,157748
4431,139	4103,421	**4216,996**	0,874882	UNCHANGED	1,163004	UNCHANGED	1,329327
14488,32	14737,96	**14821,45**	0,760708	UNCHANGED	0,788192	UNCHANGED	1,036129
745,683	602,1497	**684,2413**	1,263068	UNCHANGED	0,793915	UNCHANGED	0,628561
8686,855	8719,639	**8542,353**	0,861067	UNCHANGED	0,776051	UNCHANGED	0,901267
91163,66	112664,1	**103761**	0,79843	UNCHANGED	1,087224	UNCHANGED	1,361702
1822,161	1875,894	**2227,325**	0,672659	UNCHANGED	1,002592	UNCHANGED	1,490492
2504,062	2241,59	**2363,963**	1,353053	UNCHANGED	1,248539	UNCHANGED	0,922757
6004,225	5794,134	**6307,184**	1,01142	UNCHANGED	1,395542	UNCHANGED	1,379784
47426,98	60584,1	**55909,68**	0,769035	UNCHANGED	0,981325	UNCHANGED	1,276047
17785,52	24721,94	**22106,65**	0,84395	UNCHANGED	0,74897	UNCHANGED	0,887458
43431,28	49764,58	**48002,61**	1,168159	UNCHANGED	0,838191	UNCHANGED	0,717531
241,297	213,2649	**238,6365**	0,402309	UNCHANGED	0,615139	UNCHANGED	1,529022
2827,171	3643,433	**3401,667**	0,852445	UNCHANGED	0,660327	UNCHANGED	0,774628
173999,7	141423,2	**153384,7**	0,988367	UNCHANGED	1,117388	UNCHANGED	1,13054
4876,331	6373,641	**5711,823**	1,178068	UNCHANGED	0,947107	UNCHANGED	0,803949
3041,818	2179,405	**2592,544**	0,922245	UNCHANGED	0,538465	UNCHANGED	0,583863
27235,51	27955,47	**27756,82**	0,919892	UNCHANGED	0,814583	UNCHANGED	0,885521
77644,28	89062,77	**85511,99**	0,832804	UNCHANGED	0,861405	UNCHANGED	1,034343
36278,14	31457,27	**33302,14**	0,76925	UNCHANGED	0,952556	UNCHANGED	1,238293
2403,951	3196,245	**2911,167**	1,051817	UNCHANGED	1,242204	UNCHANGED	1,181008
12215,5	22158,07	**18902,25**	1,030705	UNCHANGED	1,431425	UNCHANGED	1,388783
440,3565	635,9555	**582,8603**	0,757219	UNCHANGED	0,549918	UNCHANGED	0,726233
4830,695	5748,985	**5402,897**	0,906446	UNCHANGED	0,837334	UNCHANGED	0,923756
22554,16	27313,91	**25469,81**	1,027109	UNCHANGED	0,838923	UNCHANGED	0,816781
147,2362	151,6544	**144,1265**	2,343467	UNCHANGED	3,127415	UNCHANGED	1,334525
9907,644	10201,47	**10295,11**	0,908248	UNCHANGED	0,991381	UNCHANGED	1,091531
71180,77	69614,24	**70194,38**	0,892795	UNCHANGED	1,064472	UNCHANGED	1,192291
65289,79	73748,96	**70870,03**	0,964693	UNCHANGED	1,048997	UNCHANGED	1,087389
45189,54	44302,22	**44031,61**	0,930269	UNCHANGED	0,883533	UNCHANGED	0,949761
6025,561	4957,749	**5390,299**	1,219026	UNCHANGED	1,317031	UNCHANGED	1,080396
6794,496	9430,043	**7691,201**	0,56911	UNCHANGED	0,649587	UNCHANGED	1,141407
719,823	575,2667	**601,169**	1,193303	UNCHANGED	1,109742	UNCHANGED	0,929975
11600,12	8817,316	**9559,537**	0,732121	UNCHANGED	1,364924	UNCHANGED	1,864341

1751,786	1718,153	**1763,932**	1,455999	UNCHANGED	1,067914	UNCHANGED	0,733458
356,8238	118,8113	**194,3681**	2,213664	UNCHANGED	1,916996	UNCHANGED	0,865983
11425,25	7793,83	**9098,809**	1,218542	UNCHANGED	1,642265	UNCHANGED	1,347729
54,43129	178,8537	**124,4045**	1,665394	UNCHANGED	0,675181	UNCHANGED	0,405418
22990,83	22218,85	**22114,11**	0,934709	UNCHANGED	0,888943	UNCHANGED	0,951037
4312,117	4989,59	**4634,11**	1,26727	UNCHANGED	0,9275	UNCHANGED	0,731889
3134,166	1785,685	**2183,093**	3,552383	UNCHANGED	0,884107	UNCHANGED	0,248877
4017,166	3361,94	**3621,531**	0,913759	UNCHANGED	1,196242	UNCHANGED	1,309145
4549,692	4474,297	**4520,24**	1,322931	UNCHANGED	1,159063	UNCHANGED	0,876132
45267,2	41260,51	**45849,19**	0,901439	UNCHANGED	0,999859	UNCHANGED	1,10918
216761,1	236835,9	**232393,4**	0,902149	UNCHANGED	1,110304	UNCHANGED	1,230731
184,0994	135,118	**156,5795**	0,397924	UNCHANGED	0,776988	UNCHANGED	1,952605
4022,831	4887,18	**4686,627**	1,134298	UNCHANGED	1,108458	UNCHANGED	0,97722
629,4625	346,497	**461,964**	1,094877	UNCHANGED	1,460597	UNCHANGED	1,334029
7110,318	10083,65	**9253,423**	0,673234	UNCHANGED	0,713242	UNCHANGED	1,059427
12020,38	15966,95	**14472,97**	0,890227	UNCHANGED	0,827345	UNCHANGED	0,929365
39649,25	35889,32	**38619,74**	0,939696	UNCHANGED	0,820131	UNCHANGED	0,872762
28034,77	24421,61	**25814,4**	1,104966	UNCHANGED	0,956883	UNCHANGED	0,865984
294,9804	336,4428	**322,3009**	0,548685	UNCHANGED	0,563888	UNCHANGED	1,02771
39109,89	48732,64	**45406,39**	1,127023	UNCHANGED	1,029793	UNCHANGED	0,913728
3369,639	5268,891	**4632,468**	1,281624	UNCHANGED	1,242809	UNCHANGED	0,969714
6982,846	7011,969	**6984,731**	0,922329	UNCHANGED	0,92104	UNCHANGED	0,998602
2009,412	1678,418	**1725,957**	0,897007	UNCHANGED	1,217812	UNCHANGED	1,357639
139040,1	135306,7	**137752,2**	1,098027	UNCHANGED	1,119139	UNCHANGED	1,019227
2401,943	2015,279	**2014,434**	0,959952	UNCHANGED	0,679714	UNCHANGED	0,708071
591,9694	561,2305	**605,5582**	1,395962	UNCHANGED	1,060477	UNCHANGED	0,759675
2528,444	2329,743	**2619,406**	0,547135	UNCHANGED	0,788914	UNCHANGED	1,4419
29599,28	36750,01	**34584,02**	1,050827	UNCHANGED	1,161009	UNCHANGED	1,104853
22389,77	17762,82	**19257,56**	0,828567	UNCHANGED	0,733417	UNCHANGED	0,885163
3719,754	2809,545	**3168,864**	1,227359	UNCHANGED	0,864975	UNCHANGED	0,704745
38339,56	32830,48	**35497,76**	1,129526	UNCHANGED	1,161696	UNCHANGED	1,028481
74,02295	31,03769	**45,73112**	0,192339	UNCHANGED	0,82861	UNCHANGED	4,308082
111254,5	112373,9	**114258**	1,086828	UNCHANGED	0,607355	UNCHANGED	0,558832
239,4523	221,0035	**240,7883**	0,823982	UNCHANGED	0,561592	UNCHANGED	0,681558
567,6852	291,8753	**409,0062**	2,081849	UNCHANGED	1,843265	UNCHANGED	0,885398
445,671	246,2438	**305,2275**	0,481776	UNCHANGED	1,66488	UNCHANGED	3,455712
235371,1	269756,3	**253355,4**	0,778498	UNCHANGED	0,906958	UNCHANGED	1,165011
6706,854	6270,566	**6416,658**	1,016782	UNCHANGED	0,763625	UNCHANGED	0,751021
455,3331	467,8877	**457,2716**	0,607496	UNCHANGED	0,418731	UNCHANGED	0,689274
2160,593	1700,555	**1804,207**	0,610538	UNCHANGED	0,759046	UNCHANGED	1,243242
6193,49	4064,247	**4820,911**	1,15586	UNCHANGED	1,989555	UNCHANGED	1,721277
208,708	228,6459	**208,1901**	0,903026	UNCHANGED	0,546553	UNCHANGED	0,605246
899,9583	948,5515	**927,9239**	1,372292	UNCHANGED	1,465997	UNCHANGED	1,068283
1693,098	2240,258	**2065,881**	0,649936	UNCHANGED	0,540933	UNCHANGED	0,832286
261,9038	402,563	**368,4492**	0,846594	UNCHANGED	1,094266	UNCHANGED	1,29255
3561,878	2642,29	**2951,294**	1,308085	UNCHANGED	2,02156	UNCHANGED	1,545435
1434,779	2163,216	**1860,668**	0,804166	UNCHANGED	0,588142	UNCHANGED	0,73137
104,5149	60,24736	**68,66524**	1,890826	UNCHANGED	2,540208	UNCHANGED	1,343438
39834,94	59374,63	**52741,9**	1,279389	UNCHANGED	1,064811	UNCHANGED	0,832281
3928,407	3525,263	**3718,812**	0,861263	UNCHANGED	1,092927	UNCHANGED	1,268982

30149,71	24944,14	**26872,91**	1,076304	UNCHANGED	1,164431	UNCHANGED	1,081879
11774,12	7681,865	**9114,119**	0,791864	UNCHANGED	1,362619	UNCHANGED	1,720774
132,4815	9,949984	**62,81741**	2,796853	UNCHANGED	3,654922	UNCHANGED	1,306798
2951,732	2511,961	**2744,489**	1,064668	UNCHANGED	0,892843	UNCHANGED	0,838612
631,0891	261,3823	**366,124**	0,382442	UNCHANGED	0,309092	UNCHANGED	0,808205
36678,91	38451,57	**37540,24**	0,869602	UNCHANGED	0,911861	UNCHANGED	1,048595
32963,76	22909,66	**26603,03**	1,34734	UNCHANGED	1,243712	UNCHANGED	0,923087
12658,12	4417,2	**7028,849**	1,630618	UNCHANGED	1,613149	UNCHANGED	0,989287
4405,979	4108,664	**4191,29**	1,177679	UNCHANGED	1,267968	UNCHANGED	1,076667
1800,467	1642,763	**1663,936**	3,010704	UNCHANGED	3,553072	UNCHANGED	1,180147
55578,96	62482,14	**60708,4**	1,10468	UNCHANGED	1,079103	UNCHANGED	0,976846
17680,58	18213,63	**18332,93**	0,839308	UNCHANGED	0,90192	UNCHANGED	1,0746
128612,5	139765,7	**135175,1**	0,868181	UNCHANGED	1,006024	UNCHANGED	1,158772
2149,764	1906,896	**1962,195**	1,652191	UNCHANGED	2,31695	UNCHANGED	1,40235
1307515	652602,3	**883288**	0,78398	UNCHANGED	1,171068	UNCHANGED	1,493746
9232,564	7619,919	**8238,343**	3,308946	UNCHANGED	0,947069	UNCHANGED	0,286215
3585,369	763,1711	**1677,276**	2,073141	UNCHANGED	0,781563	UNCHANGED	0,376995
8740,061	7663,583	**8428,748**	1,057153	UNCHANGED	1,204624	UNCHANGED	1,139497
733,3311	267,6338	**426,0576**	3,0086	UNCHANGED	1,283223	UNCHANGED	0,426518
11435,51	10080,88	**10527,64**	1,184767	UNCHANGED	1,393729	UNCHANGED	1,176374
1891,697	5554,527	**4223,283**	1,867895	UNCHANGED	1,458794	UNCHANGED	0,780982
6636,437	6379,029	**6221,818**	0,871052	UNCHANGED	0,931953	UNCHANGED	1,069917
319,7068	348,2449	**305,8419**	0,552665	UNCHANGED	0,845625	UNCHANGED	1,530086
346263,8	304068,3	**314556,5**	0,883365	UNCHANGED	0,754942	UNCHANGED	0,854621
3178,452	3999,022	**3799,919**	0,819509	UNCHANGED	0,701566	UNCHANGED	0,856081
568,5839	876,5073	**721,453**	0,590522	UNCHANGED	1,097174	UNCHANGED	1,857973
1165,688	1073,685	**1067,953**	0,809809	UNCHANGED	0,825206	UNCHANGED	1,019013
2347,372	2743,932	**2750,385**	1,472152	UNCHANGED	1,638946	UNCHANGED	1,113299
779,0722	610,4594	**684,6037**	0,661732	UNCHANGED	0,737331	UNCHANGED	1,114243
1375,054	1229,334	**1207,057**	0,995816	UNCHANGED	1,192482	UNCHANGED	1,197493
552,9889	729,6089	**622,436**	0,747829	UNCHANGED	0,794693	UNCHANGED	1,062667
1484,569	1337,959	**1432,229**	1,011937	UNCHANGED	1,327055	UNCHANGED	1,311401
481,6566	437,1161	**490,1963**	0,821873	UNCHANGED	1,052396	UNCHANGED	1,280485
361,4381	364,1242	**338,0972**	0,786308	UNCHANGED	2,105468	UNCHANGED	2,677664
3388,252	3809,022	**3778,664**	1,489378	UNCHANGED	1,095565	UNCHANGED	0,735585
278867,8	269816,4	**280600**	1,054994	UNCHANGED	1,116471	UNCHANGED	1,058272
39923,8	33924,71	**35774,85**	0,845185	UNCHANGED	0,849306	UNCHANGED	1,004875
988,2018	497,2753	**678,1776**	1,518472	UNCHANGED	0,968473	UNCHANGED	0,637795
325869,9	210104,7	**253284,6**	0,751254	UNCHANGED	0,64902	UNCHANGED	0,863915
550,4472	622,9865	**574,7417**	1,006066	UNCHANGED	0,630774	UNCHANGED	0,626971
1880,777	1920,586	**1871,872**	0,757134	UNCHANGED	0,995043	UNCHANGED	1,314223
9399,76	11663,92	**10612,61**	0,710877	UNCHANGED	0,815575	UNCHANGED	1,14728
20617,35	20688,43	**20352,61**	0,94849	UNCHANGED	0,761682	UNCHANGED	0,803046
461,0728	762,0971	**677,2531**	0,500904	UNCHANGED	0,46116	UNCHANGED	0,920654
451,3398	576,649	**483,6316**	1,260861	UNCHANGED	1,879006	UNCHANGED	1,490256
5304,238	5241,319	**5367,106**	0,805084	UNCHANGED	0,755084	UNCHANGED	0,937895
143850,7	154058,2	**153316,3**	0,914397	UNCHANGED	0,774906	UNCHANGED	0,847451
10147,33	8109,07	**8794,447**	1,015113	UNCHANGED	1,236721	UNCHANGED	1,218309
3655,654	4408,748	**4134,746**	0,703751	UNCHANGED	1,105564	UNCHANGED	1,570959
1544,353	824,3796	**1071,115**	1,367929	UNCHANGED	1,758228	UNCHANGED	1,285321

23156,87	16815,27	**18928,32**	1,20439	UNCHANGED	1,531815	UNCHANGED	1,271859
993,4862	33,34065	**1215,565**	1,04872	UNCHANGED	1,980167	UNCHANGED	1,888176
32158,31	29476,04	**31275,41**	0,806024	UNCHANGED	0,740947	UNCHANGED	0,919262
22988,89	29600,96	**27588,98**	0,837957	UNCHANGED	0,874885	UNCHANGED	1,044069
1193,692	1201,014	**1275,52**	0,860718	UNCHANGED	0,91554	UNCHANGED	1,063693
18904,49	25928,79	**23718,45**	0,778592	UNCHANGED	1,037821	UNCHANGED	1,332946
1502,949	1535,024	**1546,776**	0,654939	UNCHANGED	0,534401	UNCHANGED	0,815956
10919,17	10436,11	**10762,28**	1,353443	UNCHANGED	1,400877	UNCHANGED	1,035047
1956,433	2195,018	**2100,283**	0,999253	UNCHANGED	1,205791	UNCHANGED	1,206692
91683,6	87382,7	**89848,7**	0,845401	UNCHANGED	1,148898	UNCHANGED	1,358998
964,2408	1397,484	**1151,329**	0,831628	UNCHANGED	0,658465	UNCHANGED	0,791778
538,7639	573,2544	**539,3698**	1,144223	UNCHANGED	0,854697	UNCHANGED	0,746967
1235,264	1687,097	**1450,117**	0,809628	UNCHANGED	0,683484	UNCHANGED	0,844195
43876,4	46606,49	**44087,75**	0,930138	UNCHANGED	1,093778	UNCHANGED	1,175931
2472,599	2427,727	**2438,649**	1,337591	UNCHANGED	0,987443	UNCHANGED	0,738225
4191,853	3645,584	**3743,167**	1,308708	UNCHANGED	1,03156	UNCHANGED	0,788228
5509,525	7154,896	**6586,424**	0,943535	UNCHANGED	1,094199	UNCHANGED	1,15968
13475,59	20643,67	**16440,47**	0,578545	UNCHANGED	0,695299	UNCHANGED	1,201807
2852,552	2836,982	**3054,556**	0,968097	UNCHANGED	1,22827	UNCHANGED	1,268747
2052,772	1585,977	**1759,279**	0,685277	UNCHANGED	0,862673	UNCHANGED	1,258868
10754,74	9509,725	**10080,44**	0,837514	UNCHANGED	1,118625	UNCHANGED	1,335648
10687,34	13295,04	**12008,85**	1,280085	UNCHANGED	1,360321	UNCHANGED	1,06268
6381,385	6937,526	**6469,034**	0,572086	UNCHANGED	0,691077	UNCHANGED	1,207995
11896,85	11571,49	**12045,65**	0,729499	UNCHANGED	0,716231	UNCHANGED	0,981812
4185,374	2384,506	**3006,095**	0,904902	UNCHANGED	2,015104	UNCHANGED	2,226875
468,1261	600,8083	**544,4204**	0,947506	UNCHANGED	3,320345	UNCHANGED	3,504301
572,9326	303,0994	**345,9778**	2,154209	UNCHANGED	1,611292	UNCHANGED	0,747974
227,6677	430,9723	**308,1199**	12,26886	UNCHANGED	1,732928	UNCHANGED	0,141246
188,4094	104,8141	**131,446**	0,969318	UNCHANGED	0,560164	UNCHANGED	0,577894
21671,53	28137,97	**24583,43**	0,639007	UNCHANGED	1,79935	UNCHANGED	2,815855
5038,217	5597,461	**5185,735**	0,857634	UNCHANGED	1,151318	UNCHANGED	1,342436
9301,01	8504,675	**8920,608**	1,070212	UNCHANGED	1,654997	UNCHANGED	1,54642
85585,04	31762,11	**49715,21**	1,1429	UNCHANGED	1,811695	UNCHANGED	1,585174
407,1105	556,2866	**512,3401**	0,809963	UNCHANGED	1,996093	UNCHANGED	2,464425
7741,682	6736,799	**7134,063**	0,828724	UNCHANGED	1,395556	UNCHANGED	1,683981
1471,687	559,8531	**842,6607**	0,550124	UNCHANGED	0,546498	UNCHANGED	0,993409
472,8905	567,0988	**514,0838**	0,592122	UNCHANGED	1,772872	UNCHANGED	2,9941
332,5019	203,363	**245,9637**	2,061026	UNCHANGED	2,359337	UNCHANGED	1,144739
10761,28	7311,579	**9608,512**	1,248419	UNCHANGED	1,210599	UNCHANGED	0,969705
2886,96	1979,329	**3182,845**	0,576068	UNCHANGED	0,583691	UNCHANGED	1,013232
1882,661	1357,423	**1529,608**	0,832434	UNCHANGED	1,136478	UNCHANGED	1,365246
23119,12	24329,21	**23466,59**	0,596582	UNCHANGED	0,682557	UNCHANGED	1,144112
3513,769	4248,442	**3985,816**	0,9481	UNCHANGED	1,266068	UNCHANGED	1,335374
4044,81	4319,388	**4247,156**	0,74322	UNCHANGED	0,990652	UNCHANGED	1,332919
4562,412	4558,462	**4514,289**	0,917669	UNCHANGED	0,859186	UNCHANGED	0,93627
53537,06	68391,91	**63637,73**	0,772691	UNCHANGED	0,857814	UNCHANGED	1,110164
25456,98	29105,59	**27501,09**	0,812886	UNCHANGED	0,873056	UNCHANGED	1,074021
6718,155	7687,973	**7005,951**	1,129452	UNCHANGED	1,004619	UNCHANGED	0,889474
355,1775	515,4011	**455,9143**	1,334497	UNCHANGED	0,578607	UNCHANGED	0,433577
3097,721	2899,782	**2956,176**	1,238553	UNCHANGED	1,595665	UNCHANGED	1,288331

7549,494	7708,834	**7477,511**	0,771919	UNCHANGED	0,542206	UNCHANGED	0,702413
8957,951	9972,953	**9399,758**	0,931816	UNCHANGED	1,089049	UNCHANGED	1,168738
65338,9	55968,88	**68284,98**	1,174932	UNCHANGED	1,191735	UNCHANGED	1,014302
14909,59	15986,47	**15630,91**	0,974767	UNCHANGED	0,851422	UNCHANGED	0,873463
27416,66	29351,25	**29066,57**	1,126063	UNCHANGED	1,14183	UNCHANGED	1,014001
70,19507	246,9501	**201,4403**	1,535705	UNCHANGED	1,780904	UNCHANGED	1,159665
92,32476	148,0677	**88,76935**	1,591468	UNCHANGED	3,310846	UNCHANGED	2,080372
576,2119	667,8322	**640,1437**	1,156577	UNCHANGED	1,352709	UNCHANGED	1,16958
21023,55	24659,3	**22598,05**	1,272898	UNCHANGED	0,651291	UNCHANGED	0,51166
4489,524	4181,486	**4435,627**	0,933432	UNCHANGED	1,086086	UNCHANGED	1,16354
10127,07	11884,25	**10656,27**	0,91519	UNCHANGED	0,815204	UNCHANGED	0,890749
40779,4	27323,79	**31861,77**	1,021083	UNCHANGED	1,211089	UNCHANGED	1,186082
71,62969	99,4951	**85,7311**	2,075995	UNCHANGED	0,777536	UNCHANGED	0,374537
160,6705	24,49062	**63,68784**	0,041871	UNCHANGED	0,763445	UNCHANGED	18,23321
19757,89	23285,85	**22143,37**	0,99303	UNCHANGED	0,870076	UNCHANGED	0,876183
4156,711	3710,058	**3813,16**	0,856084	UNCHANGED	0,787434	UNCHANGED	0,919809
25549,29	26663,56	**25932,64**	1,040238	UNCHANGED	0,913998	UNCHANGED	0,878642
7874,786	10972,09	**10201,44**	0,929692	UNCHANGED	0,790123	UNCHANGED	0,849876
7274,706	7077,895	**7059,902**	0,990074	UNCHANGED	0,91897	UNCHANGED	0,928183
1368,764	1089,986	**1849,724**	2,111433	UNCHANGED	3,114306	UNCHANGED	1,474972
9297,341	8535,354	**9105,817**	1,329514	UNCHANGED	0,991862	UNCHANGED	0,746033
4070,656	4963,263	**4775,694**	1,055146	UNCHANGED	0,823653	UNCHANGED	0,780606
9676,47	10007	**10034,54**	0,884038	UNCHANGED	0,988062	UNCHANGED	1,117669
1910,987	2314,831	**2218,577**	0,610129	UNCHANGED	0,586917	UNCHANGED	0,961956
28387,31	22054,93	**24606,27**	0,926815	UNCHANGED	0,818397	UNCHANGED	0,883021
2094,87	911,235	**1296,868**	1,567835	UNCHANGED	1,192614	UNCHANGED	0,760676
2308,72	1934,056	**2046,208**	0,840323	UNCHANGED	0,741825	UNCHANGED	0,882786
33978,17	26809,97	**29434,45**	1,178256	UNCHANGED	0,990561	UNCHANGED	0,840701
702,3709	483,935	**585,6065**	1,537366	UNCHANGED	1,114151	UNCHANGED	0,724714
26146,91	26278,39	**26148,01**	0,904653	UNCHANGED	0,919767	UNCHANGED	1,016707
36176,83	24101,29	**27817,86**	1,286014	UNCHANGED	1,248379	UNCHANGED	0,970735
66561,25	141569,7	**109405,3**	0,601062	UNCHANGED	0,8827	UNCHANGED	1,468568
591,6906	656,5335	**565,0301**	3,079881	UNCHANGED	1,04306	UNCHANGED	0,338669
3846,309	3739,648	**3709,698**	0,5848	UNCHANGED	0,721776	UNCHANGED	1,234228
2958,373	3615,146	**3109,254**	0,692373	UNCHANGED	0,7545	UNCHANGED	1,08973
140,3983	842,4258	**564,4935**	0,508573	UNCHANGED	0,328883	UNCHANGED	0,646678
47468,99	49254,05	**48351,12**	0,891343	UNCHANGED	1,063019	UNCHANGED	1,192603
3689,572	1418,433	**2539,59**	1,23967	UNCHANGED	1,351056	UNCHANGED	1,089851
12098,21	13279,07	**12666,84**	1,068421	UNCHANGED	0,947876	UNCHANGED	0,887174
740,8299	1183,633	**1018,069**	1,487999	UNCHANGED	1,049371	UNCHANGED	0,705223
71,18416	89,71604	**59,62011**	0,515614	UNCHANGED	0,466874	UNCHANGED	0,905472
25331,46	13570,71	**16925,05**	0,919868	UNCHANGED	1,508806	UNCHANGED	1,640241
13822,48	14161,24	**14053,77**	0,920585	UNCHANGED	1,039047	UNCHANGED	1,128681
1764,703	2587,625	**2372,435**	0,448516	UNCHANGED	0,540385	UNCHANGED	1,204829
8481,721	7878,291	**7900,978**	1,109838	UNCHANGED	0,870612	UNCHANGED	0,784449
1980,433	1907,18	**1875,016**	0,982674	UNCHANGED	1,31902	UNCHANGED	1,342276
1898,11	2197,526	**2072,845**	1,037925	UNCHANGED	1,12611	UNCHANGED	1,084963
7193,964	6320,251	**6690,161**	1,100636	UNCHANGED	1,049922	UNCHANGED	0,953923
49620,22	40224,2	**42938,99**	1,020481	UNCHANGED	1,11124	UNCHANGED	1,088938
32350,42	40334,65	**37760,77**	1,127715	UNCHANGED	1,01706	UNCHANGED	0,901877

4211,134	4214,207	**4226,224**	0,849477	UNCHANGED	1,162589	UNCHANGED	1,368593
107961	116365,9	**115535**	0,887389	UNCHANGED	0,963846	UNCHANGED	1,086159
570,7599	608,9195	**573,6527**	0,557721	UNCHANGED	0,335634	UNCHANGED	0,601796
101,4843	176,3273	**152,5595**	0,739988	UNCHANGED	0,500359	UNCHANGED	0,676172
30807,58	40392,99	**36979,48**	0,896362	UNCHANGED	1,082872	UNCHANGED	1,208074
1682,71	2566,844	**2339,119**	0,859796	UNCHANGED	0,765688	UNCHANGED	0,890547
4118,302	7895,598	**6577,335**	0,723745	UNCHANGED	0,684453	UNCHANGED	0,94571
1383,606	2573,72	**2123,389**	0,738038	UNCHANGED	1,051744	UNCHANGED	1,425055
25839,43	23290,96	**23612,26**	0,869787	UNCHANGED	0,867032	UNCHANGED	0,996832
50421,48	54938,87	**55148,65**	0,984851	UNCHANGED	1,149696	UNCHANGED	1,167381
1165,478	1724,319	**1547,79**	1,316458	UNCHANGED	0,757273	UNCHANGED	0,575235
1883,986	1553,169	**1617,3**	1,496715	UNCHANGED	2,250534	UNCHANGED	1,503649
41721,66	44874,2	**44388,28**	1,083044	UNCHANGED	0,980585	UNCHANGED	0,905398
3261,146	3604,63	**3527,354**	0,796735	UNCHANGED	0,779229	UNCHANGED	0,978027
4962,515	7900,933	**6614,007**	0,725859	UNCHANGED	0,88407	UNCHANGED	1,217963
417,6359	87,06673	**239,9218**	0,726229	UNCHANGED	1,143533	UNCHANGED	1,574617
22077,99	16772,24	**18613,99**	1,115538	UNCHANGED	0,996056	UNCHANGED	0,892893
1457,868	2650,749	**2283,456**	0,675441	UNCHANGED	0,985389	UNCHANGED	1,458881
497,719	619,1073	**571,0095**	1,304596	UNCHANGED	1,450259	UNCHANGED	1,111654
14570	13336,05	**13580,9**	1,010777	UNCHANGED	1,149064	UNCHANGED	1,136813
1689,88	1178,008	**1279,371**	0,636758	UNCHANGED	0,546119	UNCHANGED	0,857656
163086,3	185469,6	**180840,5**	1,006757	UNCHANGED	1,074979	UNCHANGED	1,067765
10290,58	11707,17	**11182,56**	0,870505	UNCHANGED	0,958812	UNCHANGED	1,101444
3957,273	4283,27	**4508,406**	1,086283	UNCHANGED	1,417776	UNCHANGED	1,305162
4363,633	4050,525	**4427,837**	0,921893	UNCHANGED	1,156807	UNCHANGED	1,254816
137083,1	128057,1	**134483,4**	0,830147	UNCHANGED	0,953575	UNCHANGED	1,148681
238,2755	306,4517	**260,3448**	0,52066	UNCHANGED	0,295614	UNCHANGED	0,567769
440,3155	483,667	**451,8445**	0,729388	UNCHANGED	0,637281	UNCHANGED	0,87372
8187,501	9646,934	**9320,862**	0,934212	UNCHANGED	0,836263	UNCHANGED	0,895153
68143,25	39905,58	**50010,6**	1,163962	UNCHANGED	0,909114	UNCHANGED	0,781051
56858,6	61283,71	**59992,23**	0,885051	UNCHANGED	1,033555	UNCHANGED	1,167792
18296,06	17813,05	**17967,98**	0,916643	UNCHANGED	1,020322	UNCHANGED	1,113107
17769,3	19021,31	**18159,44**	0,940352	UNCHANGED	0,902653	UNCHANGED	0,95991
4354,244	3602,338	**3927,67**	1,055771	UNCHANGED	1,228409	UNCHANGED	1,163519
51812,04	53592,51	**54198,97**	0,987359	UNCHANGED	0,920613	UNCHANGED	0,9324
846,1117	611,4345	**679,1485**	0,484702	UNCHANGED	0,64663	UNCHANGED	1,334077
1676,008	1269,71	**1446,941**	1,312277	UNCHANGED	1,828487	UNCHANGED	1,393369
7483,006	6162,095	**6780,833**	1,124097	UNCHANGED	1,13594	UNCHANGED	1,010535
8957,733	10918,31	**10568,13**	1,195651	UNCHANGED	1,421578	UNCHANGED	1,188957
2100,958	1026,534	**1439,161**	1,463365	UNCHANGED	0,934858	UNCHANGED	0,638841
217,8445	1033,834	**649,1794**	0,647731	UNCHANGED	0,429848	UNCHANGED	0,66362
10275,09	10778,11	**9755,942**	0,887372	UNCHANGED	1,071991	UNCHANGED	1,208051
129,4861	168,1066	**150,5036**	0,399857	UNCHANGED	0,744471	UNCHANGED	1,861845
38229,88	32029,79	**34639,99**	1,153601	UNCHANGED	1,063359	UNCHANGED	0,921773
6318,977	4521,982	**5387,948**	1,507674	UNCHANGED	1,202775	UNCHANGED	0,797769
6089,962	7287,269	**7074,371**	1,250758	UNCHANGED	1,399088	UNCHANGED	1,118592
1793,832	2431,037	**2159,81**	0,791578	UNCHANGED	1,146223	UNCHANGED	1,448022
11258,37	12954,13	**12229,61**	1,108141	UNCHANGED	0,88544	UNCHANGED	0,799032
202,7414	159,6784	**156,4959**	0,44165	UNCHANGED	0,91853	UNCHANGED	2,079768
1171,684	1708,769	**1573,31**	0,778691	UNCHANGED	0,740895	UNCHANGED	0,951462

84877,27	89897,61	**88967,96**	0,774852	UNCHANGED	0,858664	UNCHANGED	1,108165
1572,5	907,2887	**1128,164**	0,887457	UNCHANGED	1,348205	UNCHANGED	1,519177
75962,61	80457,61	**78408,71**	0,796817	UNCHANGED	1,038794	UNCHANGED	1,30368
13720,07	11654,11	**12223,1**	0,849323	UNCHANGED	1,251368	UNCHANGED	1,473371
2054,742	1959,74	**2065,452**	1,209452	UNCHANGED	1,440381	UNCHANGED	1,190937
8486,528	8260,727	**8640,992**	1,141766	UNCHANGED	1,116573	UNCHANGED	0,977936
3457,175	4371,365	**5437,869**	1,06385	UNCHANGED	1,309456	UNCHANGED	1,230865
70458,22	68461,89	**69112,56**	0,874805	UNCHANGED	0,941767	UNCHANGED	1,076546
19819,92	17392,4	**18122,14**	1,119605	UNCHANGED	1,088896	UNCHANGED	0,972572
846,1619	718,466	**796,9406**	1,479191	UNCHANGED	0,796052	UNCHANGED	0,538167
7994,556	6668,123	**6687,112**	0,692157	UNCHANGED	0,851725	UNCHANGED	1,230537
736,6245	1363,219	**1016,563**	0,869577	UNCHANGED	0,709063	UNCHANGED	0,815411
2112,188	2213,422	**2126,97**	0,7596	UNCHANGED	0,807973	UNCHANGED	1,063682
33920,37	41184,86	**39075,88**	1,126142	UNCHANGED	1,04209	UNCHANGED	0,925363
21465,8	22381,62	**21517,12**	1,324014	UNCHANGED	0,735841	UNCHANGED	0,555765
2546,322	2529,102	**2521,795**	1,266865	UNCHANGED	1,722988	UNCHANGED	1,36004
410,2884	491,0949	**407,9856**	1,592747	UNCHANGED	2,59827	UNCHANGED	1,631314
150113,8	176865,6	**166003,1**	0,76471	UNCHANGED	0,789143	UNCHANGED	1,03195
275,8313	127,0223	**206,6362**	1,867392	UNCHANGED	0,763058	UNCHANGED	0,408622
21683,11	14603,61	**17819,12**	1,075731	UNCHANGED	1,257113	UNCHANGED	1,168613
2146,952	2346,926	**2288,122**	0,779541	UNCHANGED	0,515199	UNCHANGED	0,6609
704,4504	765,9263	**680,2428**	0,681468	UNCHANGED	0,981063	UNCHANGED	1,439631
8322,64	7373,285	**7633,32**	1,034253	UNCHANGED	1,521915	UNCHANGED	1,471511
35198	31394,07	**31755,56**	0,795098	UNCHANGED	0,82718	UNCHANGED	1,040349
753078,2	822680,4	**808932**	0,844694	UNCHANGED	0,902645	UNCHANGED	1,068605
8048,769	5796,389	**6615,638**	1,099137	UNCHANGED	1,230087	UNCHANGED	1,119139
155,0607	226,9981	**161,7701**	1,338537	UNCHANGED	0,780452	UNCHANGED	0,583063
442,6532	238,6772	**451,8822**	0,525693	UNCHANGED	0,610095	UNCHANGED	1,160553
283,5444	414,452	**374,2762**	0,871767	UNCHANGED	0,701733	UNCHANGED	0,804955
299,1046	521,7323	**442,3264**	0,838469	UNCHANGED	1,282824	UNCHANGED	1,52996
5714,606	2809,983	**3921,857**	0,707052	UNCHANGED	0,663765	UNCHANGED	0,938779
2295,25	2317,89	**2126,446**	0,803812	UNCHANGED	1,304951	UNCHANGED	1,623453
1895,264	2299,596	**2171,504**	1,026959	UNCHANGED	0,8245	UNCHANGED	0,802856
252,9379	46,7161	**336,0789**	0,628248	UNCHANGED	1,093294	UNCHANGED	1,740226
4325,61	2601,838	**3257,562**	1,169325	UNCHANGED	1,229689	UNCHANGED	1,051623
7191,761	12352,92	**10782,4**	0,660106	UNCHANGED	0,722671	UNCHANGED	1,09478
10376,77	5014,839	**9163,176**	0,702472	UNCHANGED	0,60958	UNCHANGED	0,867763
5251,36	5449,447	**5376,046**	0,884475	UNCHANGED	0,78126	UNCHANGED	0,883303
5798,489	5738,513	**5481,055**	1,138155	UNCHANGED	0,958268	UNCHANGED	0,841949
5192,229	4545,751	**4873,944**	1,107382	UNCHANGED	1,20601	UNCHANGED	1,089064
2650,302	1504,997	**1806,82**	0,795252	UNCHANGED	0,698587	UNCHANGED	0,878447
6590,34	17514,34	**12205,04**	1,857694	UNCHANGED	1,098307	UNCHANGED	0,59122
12333,5	8846,205	**10129,61**	1,706924	UNCHANGED	1,452639	UNCHANGED	0,851027
27630,88	33086,36	**31233,91**	0,868807	UNCHANGED	0,868676	UNCHANGED	0,999849
17324,82	13307,23	**14497,96**	0,665454	UNCHANGED	0,970033	UNCHANGED	1,457702
20657,89	25479,98	**24124,07**	1,218505	UNCHANGED	1,567955	UNCHANGED	1,286785
3364,682	4571,128	**4144,696**	0,754588	UNCHANGED	0,497926	UNCHANGED	0,659865
4628,696	5489,637	**5058,819**	0,929117	UNCHANGED	0,826905	UNCHANGED	0,88999
1760,352	1824,104	**1776,558**	0,807234	UNCHANGED	0,935621	UNCHANGED	1,159046
3639,732	1311,879	**1906,202**	0,437396	UNCHANGED	0,698779	UNCHANGED	1,59759

2004,368	2490,208	**2399,991**	0,998794	UNCHANGED	0,810142	UNCHANGED	0,81112
4502,561	3485,864	**3370,294**	0,747088	UNCHANGED	0,732129	UNCHANGED	0,979977
4715,208	5253,939	**5811,865**	0,825878	UNCHANGED	0,704929	UNCHANGED	0,853551
32125,15	27372,84	**28326,78**	0,814911	UNCHANGED	0,931065	UNCHANGED	1,142535
1613,173	1570,313	**1628,193**	1,335171	UNCHANGED	1,172891	UNCHANGED	0,878457
1330,975	1516,499	**1659,882**	0,545375	UNCHANGED	0,76886	UNCHANGED	1,409781
102364,3	131893,9	**121785,1**	0,783519	UNCHANGED	0,958647	UNCHANGED	1,223516
5073,067	8197,929	**7386,474**	1,122559	UNCHANGED	0,705672	UNCHANGED	0,628628
17030,96	15478,46	**16428,49**	1,165714	UNCHANGED	1,159235	UNCHANGED	0,994441
1871,596	2207,284	**2079,96**	1,118566	UNCHANGED	0,780632	UNCHANGED	0,697886
22533,93	8750,166	**13201,74**	0,539298	UNCHANGED	0,717248	UNCHANGED	1,329966
70216,91	71229,56	**71117,91**	0,985731	UNCHANGED	0,931064	UNCHANGED	0,944541
7206,291	8989,982	**8430,032**	0,867672	UNCHANGED	1,030069	UNCHANGED	1,187164
761,1941	642,85	**668,825**	0,919264	UNCHANGED	1,181425	UNCHANGED	1,285186
1011,193	1133,487	**1131,431**	1,043536	UNCHANGED	1,222132	UNCHANGED	1,171145
125417	161919,1	**149181,7**	0,839154	UNCHANGED	0,867779	UNCHANGED	1,034112
13674,43	6960,718	**8834,535**	0,63672	UNCHANGED	0,769503	UNCHANGED	1,208542
29758,76	40182,34	**37142,37**	0,862754	UNCHANGED	0,846136	UNCHANGED	0,980737
39528,31	40044,18	**41789,5**	1,094386	UNCHANGED	1,056631	UNCHANGED	0,965502
32340,1	25184,39	**28026,06**	0,949977	UNCHANGED	1,045841	UNCHANGED	1,100911
20291,76	20390,11	**20543,96**	1,257602	UNCHANGED	1,153548	UNCHANGED	0,91726
16128,03	22946,59	**20491,56**	1,023526	UNCHANGED	0,868553	UNCHANGED	0,848589
10999,08	8265,491	**9409,02**	0,846885	UNCHANGED	1,074021	UNCHANGED	1,268202
226,1026	105,595	**214,4722**	0,546825	UNCHANGED	1,121167	UNCHANGED	2,05032
43572,76	24684,75	**31317,88**	1,164652	UNCHANGED	1,224708	UNCHANGED	1,051565
666,0287	748,5831	**653,1747**	0,889854	UNCHANGED	1,374549	UNCHANGED	1,544691
1068,461	876,9494	**1012,011**	0,923633	UNCHANGED	1,137635	UNCHANGED	1,231695
84,62397	71,73516	**80,06859**	1,782932	UNCHANGED	1,174232	UNCHANGED	0,658596
11407,18	11343,09	**11238,58**	0,960522	UNCHANGED	0,826601	UNCHANGED	0,860574
79,37421	160,0966	**127,3646**	1,539237	UNCHANGED	1,642309	UNCHANGED	1,066963
780,6962	1008,446	**803,7914**	0,888141	UNCHANGED	1,673672	UNCHANGED	1,884466
2118,353	1802,586	**1960,49**	1,265877	UNCHANGED	1,093177	UNCHANGED	0,863572
757,2766	410,7128	**508,175**	1,059962	UNCHANGED	0,542152	UNCHANGED	0,511482
42953,93	41803,16	**42669,25**	0,922706	UNCHANGED	0,843623	UNCHANGED	0,914292
2198,781	1734,511	**1927,479**	0,850982	UNCHANGED	1,116296	UNCHANGED	1,311774
5098,116	5118,584	**5222,973**	1,135315	UNCHANGED	1,029827	UNCHANGED	0,907085
392,5379	421,6296	**347,905**	0,767645	UNCHANGED	1,076269	UNCHANGED	1,40204
34707,13	37719,99	**36639,14**	1,109801	UNCHANGED	1,107351	UNCHANGED	0,997792
2085,043	2415,122	**2258,257**	0,888283	UNCHANGED	0,757225	UNCHANGED	0,852459
11406,74	18870,09	**15074,49**	0,712442	UNCHANGED	0,662797	UNCHANGED	0,930318
9622,368	10100,46	**10142,98**	0,887006	UNCHANGED	1,045674	UNCHANGED	1,17888
53022,03	61503,91	**58523,5**	0,790965	UNCHANGED	0,913248	UNCHANGED	1,154599
8930,781	4468,641	**5824,531**	0,674914	UNCHANGED	1,033833	UNCHANGED	1,531798
5559,261	4101,154	**4662,075**	1,250237	UNCHANGED	1,405199	UNCHANGED	1,123946
626011,7	434566	**503256,2**	0,802356	UNCHANGED	1,060161	UNCHANGED	1,321311
13308,01	12800,37	**13228,59**	0,836239	UNCHANGED	0,87327	UNCHANGED	1,044283
54686,66	58116,98	**57621,35**	1,052638	UNCHANGED	1,051549	UNCHANGED	0,998965
36648,13	38846,21	**37506,56**	0,846396	UNCHANGED	0,965643	UNCHANGED	1,140888
2463,32	2106,762	**2300,879**	1,389215	UNCHANGED	1,854639	UNCHANGED	1,335026
3291,967	2100,119	**2477,935**	1,091677	UNCHANGED	1,751709	UNCHANGED	1,604604

1177,123	746,5526	**798,9775**	0,468537	UNCHANGED	0,774005	UNCHANGED	1,651961
10103,78	5920,801	**7429,695**	1,219828	UNCHANGED	1,200167	UNCHANGED	0,983882
24506,86	27555,67	**26347,42**	1,150289	UNCHANGED	1,079768	UNCHANGED	0,938693
79907,86	92559,66	**84513,71**	0,86991	UNCHANGED	0,938774	UNCHANGED	1,079163
10735,73	10054,82	**9990,567**	1,060023	UNCHANGED	1,158901	UNCHANGED	1,093279
2790,277	4720,207	**3702,69**	0,821541	UNCHANGED	0,656741	UNCHANGED	0,799401
24217,95	24917,2	**24612,92**	0,738892	UNCHANGED	0,80125	UNCHANGED	1,084394
1508,754	1743,22	**1676,754**	0,822716	UNCHANGED	1,118437	UNCHANGED	1,359445
42615,94	45990,05	**45147,44**	0,807141	UNCHANGED	0,80093	UNCHANGED	0,992305
6495,966	7806,966	**7443,618**	0,894744	UNCHANGED	0,986371	UNCHANGED	1,102406
3180,446	2835,081	**3037,702**	0,756365	UNCHANGED	1,036868	UNCHANGED	1,370856
93465	99858,68	**72309,15**	0,287678	UNCHANGED	0,362786	UNCHANGED	1,261084
5508,524	8718,631	**7622,984**	0,772655	UNCHANGED	0,821985	UNCHANGED	1,063844
1835,309	2098,636	**1918,285**	0,865478	UNCHANGED	1,162562	UNCHANGED	1,343261
52482,65	61338,05	**57585,22**	0,848063	UNCHANGED	0,878144	UNCHANGED	1,03547
18477,08	21341,51	**20331,28**	0,994057	UNCHANGED	1,189213	UNCHANGED	1,196323
1472,187	1193,589	**1289,488**	1,130591	UNCHANGED	1,535598	UNCHANGED	1,358226
15951,87	16970,24	**16346,42**	1,221018	UNCHANGED	0,784507	UNCHANGED	0,642503
623,3896	627,5681	**574,7399**	0,941455	UNCHANGED	0,630661	UNCHANGED	0,669878
3521,722	1836,44	**2472,654**	1,226348	UNCHANGED	1,351767	UNCHANGED	1,10227
6916,905	6687,478	**6819,612**	1,039217	UNCHANGED	1,103545	UNCHANGED	1,061901
135,2827	743,1333	**528,6776**	0,743601	UNCHANGED	0,357715	UNCHANGED	0,481058
404,0505	368,9404	**331,8128**	1,452519	UNCHANGED	1,452613	UNCHANGED	1,000065
10207,7	12512,24	**11759,32**	1,146891	UNCHANGED	0,941729	UNCHANGED	0,821114
38977,58	38792,19	**39121,05**	0,822592	UNCHANGED	0,88637	UNCHANGED	1,077533
23476,39	21187,38	**21994,18**	1,073385	UNCHANGED	1,465632	UNCHANGED	1,365431
22887,1	30212,66	**28235,19**	1,269806	UNCHANGED	0,845818	UNCHANGED	0,666101
1756,814	957,7015	**1265,62**	1,226025	UNCHANGED	0,864365	UNCHANGED	0,705014
3795,089	3796,466	**3696,576**	0,954389	UNCHANGED	1,203473	UNCHANGED	1,260988
672,9898	1114,019	**959,4694**	1,355763	UNCHANGED	1,16773	UNCHANGED	0,861309
7080,174	9311,586	**7687,106**	0,85418	UNCHANGED	0,870708	UNCHANGED	1,019349
7047,529	5661,579	**6067,773**	0,99816	UNCHANGED	0,818514	UNCHANGED	0,820023
11020,78	9428,953	**9775,842**	0,888762	UNCHANGED	0,791521	UNCHANGED	0,890589
138,661	104,3019	**114,3846**	1,024914	UNCHANGED	0,780412	UNCHANGED	0,761441
3437,968	3446,396	**3379,669**	1,120655	UNCHANGED	0,949542	UNCHANGED	0,84731
3389,253	3673,028	**3700,44**	0,689799	UNCHANGED	0,798764	UNCHANGED	1,157966
55192,83	48092,97	**50350,92**	1,071072	UNCHANGED	1,28702	UNCHANGED	1,201619
167,4711	167,2515	**176,1917**	2,459911	UNCHANGED	1,099983	UNCHANGED	0,447164
3146,92	2666,933	**3403,836**	0,620199	UNCHANGED	0,697153	UNCHANGED	1,124079
666,3847	688,7106	**676,6578**	1,17793	UNCHANGED	0,976228	UNCHANGED	0,828766
510,5253	1712,492	**1354,345**	0,753995	UNCHANGED	0,512647	UNCHANGED	0,679908
1333,904	1441,66	**1363,506**	0,934701	UNCHANGED	0,783391	UNCHANGED	0,838119
391,878	313,1112	**304,7467**	2,365959	UNCHANGED	2,397003	UNCHANGED	1,013121
97595,06	120653,1	**111355,4**	1,111032	UNCHANGED	1,073408	UNCHANGED	0,966136
12930,57	16919,57	**15613,18**	0,960135	UNCHANGED	1,106609	UNCHANGED	1,152556
5104,224	5406,584	**5194,311**	1,011497	UNCHANGED	0,851014	UNCHANGED	0,841341
135287,1	96894,78	**110289,2**	1,042383	UNCHANGED	1,136895	UNCHANGED	1,090669
17090,81	19029,35	**18220,33**	0,941747	UNCHANGED	0,916417	UNCHANGED	0,973103
1246,388	237,7602	**1436,04**	0,331324	UNCHANGED	0,618929	UNCHANGED	1,868048
778,5922	681,5818	**760,9761**	1,057723	UNCHANGED	0,80344	UNCHANGED	0,759594

156,9807	65,64008	**85,57942**	1,13006	UNCHANGED	1,590235	UNCHANGED	1,407212
5251,131	6143,205	**5757,721**	1,037385	UNCHANGED	1,104642	UNCHANGED	1,064833
46360,54	43805,87	**46144,93**	1,124532	UNCHANGED	1,078705	UNCHANGED	0,959248
71,38791	63,58666	**68,52482**	0,164805	UNCHANGED	0,503411	UNCHANGED	3,05459
89922,34	75021,99	**80149,76**	0,874641	UNCHANGED	0,912961	UNCHANGED	1,043813
4225,949	4078,521	**4249,086**	1,143248	UNCHANGED	1,164432	UNCHANGED	1,01853
6498,416	3088,358	**4178,02**	1,114315	UNCHANGED	0,789104	UNCHANGED	0,708152
1857,196	1874,351	**1810,297**	0,803013	UNCHANGED	1,145836	UNCHANGED	1,426921
950,1049	855,7768	**919,7306**	0,896009	UNCHANGED	0,875568	UNCHANGED	0,977187
247,5906	356,3522	**315,6046**	0,641383	UNCHANGED	0,818846	UNCHANGED	1,276688
51281,56	51741,34	**51790,53**	1,167092	UNCHANGED	1,066661	UNCHANGED	0,913948
4015,74	3601,115	**3871,941**	0,750646	UNCHANGED	1,077476	UNCHANGED	1,435399
79568,71	91637,23	**84910,3**	0,943139	UNCHANGED	1,10582	UNCHANGED	1,172489
780,6665	939,8578	**925,7778**	0,949652	UNCHANGED	1,10623	UNCHANGED	1,164878
1635,403	1690,715	**1613,96**	0,625476	UNCHANGED	0,80385	UNCHANGED	1,285182
996,4809	1193,074	**1144,043**	0,837248	UNCHANGED	0,879587	UNCHANGED	1,05057
514,1847	522,2661	**350,5543**	2,274395	UNCHANGED	2,059879	UNCHANGED	0,905682
48328	64323,14	**61012,05**	0,839731	UNCHANGED	0,897988	UNCHANGED	1,069375
75139,49	72192,09	**71722,03**	1,033305	UNCHANGED	0,908372	UNCHANGED	0,879094
444689,3	405227,8	**413716,5**	0,852207	UNCHANGED	0,827898	UNCHANGED	0,971475
3516,802	4041,113	**3845,774**	0,896718	UNCHANGED	0,838313	UNCHANGED	0,934868
2567,726	2378,465	**2232,592**	0,846188	UNCHANGED	1,089026	UNCHANGED	1,286978
5416,718	914,7166	**2450,231**	0,500629	UNCHANGED	0,708565	UNCHANGED	1,41535
19456,57	20606,53	**19612,68**	0,839179	UNCHANGED	0,862993	UNCHANGED	1,028378
1306,735	380,2053	**722,1279**	0,893722	UNCHANGED	1,253181	UNCHANGED	1,402205
52976,94	60157,31	**57246,69**	0,82045	UNCHANGED	0,918859	UNCHANGED	1,119945
1156,058	885,804	**987,7803**	1,137877	UNCHANGED	0,767309	UNCHANGED	0,674334
4009,124	3288,597	**3506,635**	1,27552	UNCHANGED	1,349393	UNCHANGED	1,057916
5410,707	2337,965	**3341,158**	0,575616	UNCHANGED	0,784336	UNCHANGED	1,362603
23256,88	28461,6	**27663,44**	0,959718	UNCHANGED	0,90511	UNCHANGED	0,9431
14497,53	13997,17	**14188,64**	1,168663	UNCHANGED	1,125407	UNCHANGED	0,962986
22527,87	17151,37	**18503,06**	0,837678	UNCHANGED	0,700246	UNCHANGED	0,835937
10640,11	12564,15	**12106,01**	0,914349	UNCHANGED	1,051304	UNCHANGED	1,149784
337,6588	1048,656	**707,4237**	0,589983	UNCHANGED	0,763064	UNCHANGED	1,293365
14552,19	9069,151	**10356,57**	1,247871	UNCHANGED	1,250671	UNCHANGED	1,002244
21162,38	25694,49	**23332,59**	0,842312	UNCHANGED	0,815372	UNCHANGED	0,968017
212,479	141,1678	**153,077**	1,651211	UNCHANGED	1,348125	UNCHANGED	0,816446
5501,206	5978,636	**5966,114**	1,108312	UNCHANGED	1,020273	UNCHANGED	0,920565
332,6544	186,2342	**234,5085**	1,283426	UNCHANGED	1,000444	UNCHANGED	0,77951
421,1843	102,808	**177,154**	0,341942	UNCHANGED	0,312877	UNCHANGED	0,915001
27382,95	25517,77	**26345,62**	0,863169	UNCHANGED	0,976216	UNCHANGED	1,130968
1210,005	1306,819	**1267,75**	0,89948	UNCHANGED	1,20407	UNCHANGED	1,338629
3972,956	3878,142	**4088,219**	0,894896	UNCHANGED	0,9382	UNCHANGED	1,04839
7384,696	7073,113	**7213,808**	1,311857	UNCHANGED	0,852634	UNCHANGED	0,649944
345362,1	168301	**229958,2**	1,102801	UNCHANGED	1,204871	UNCHANGED	1,092555
3977,823	7088,207	**3977,32**	1,055061	UNCHANGED	1,301678	UNCHANGED	1,233747
787222,3	928361	**891366,6**	0,852502	UNCHANGED	0,84959	UNCHANGED	0,996584
13785,29	16967,84	**16183,73**	0,831956	UNCHANGED	0,908088	UNCHANGED	1,09151
1375,961	349,7418	**646,8505**	0,457019	UNCHANGED	0,470009	UNCHANGED	1,028422
2940,058	1903,282	**2412,664**	0,818062	UNCHANGED	0,830865	UNCHANGED	1,015651

5335,447	3594,065	**4104,991**	1,139807	UNCHANGED	0,956543	UNCHANGED	0,839215
662,7993	1305,304	**1014,604**	0,867757	UNCHANGED	1,185968	UNCHANGED	1,366705
135919,7	147361,4	**145052,5**	0,904037	UNCHANGED	0,93868	UNCHANGED	1,03832
49425,65	44347,42	**43473,87**	0,816842	UNCHANGED	0,809328	UNCHANGED	0,990801
70904,82	75473,5	**72368,21**	0,791661	UNCHANGED	1,008751	UNCHANGED	1,274222
8501,367	9688,8	**9549,595**	1,23458	UNCHANGED	1,31992	UNCHANGED	1,069125
18319,15	21963,24	**21391,72**	0,919865	UNCHANGED	1,032029	UNCHANGED	1,121935
4513,823	5218,816	**4850,476**	0,901854	UNCHANGED	1,026563	UNCHANGED	1,138281
15282,95	16936,55	**16533,87**	0,765704	UNCHANGED	0,898653	UNCHANGED	1,17363
20955,93	23259,13	**22495,87**	1,022357	UNCHANGED	1,132116	UNCHANGED	1,107359
1937,679	1548,92	**1655,055**	0,927506	UNCHANGED	0,783397	UNCHANGED	0,844628
1894,868	2167,658	**2120,869**	1,206845	UNCHANGED	1,084157	UNCHANGED	0,898341
57766,4	39524,54	**45551,58**	1,162099	UNCHANGED	1,182525	UNCHANGED	1,017577
98245,82	90910,99	**93934,45**	1,338253	UNCHANGED	0,983466	UNCHANGED	0,734888
3228,741	954,8104	**1861,246**	0,459512	UNCHANGED	0,890602	UNCHANGED	1,938146
6994,849	7989,383	**7251,403**	1,218024	UNCHANGED	1,20366	UNCHANGED	0,988208
1338,22	597,1654	**843,4211**	1,405258	UNCHANGED	1,128399	UNCHANGED	0,802983
3605,592	1664,776	**2465,862**	0,980842	UNCHANGED	1,610519	UNCHANGED	1,641976
966,3821	2209,484	**1568,838**	1,471027	UNCHANGED	1,192589	UNCHANGED	0,810718
168326,1	172383,9	**174207,3**	0,963365	UNCHANGED	0,916432	UNCHANGED	0,951282
327108,3	384125,1	**359725,1**	0,888648	UNCHANGED	0,864055	UNCHANGED	0,972326
55138,93	21510,54	**32891,78**	0,767288	UNCHANGED	1,210186	UNCHANGED	1,577226
5449,231	5333,817	**5371,629**	1,144603	UNCHANGED	0,887479	UNCHANGED	0,77536
3643,987	2722,223	**3050,299**	0,961923	UNCHANGED	1,245858	UNCHANGED	1,295174
2248,184	2607,18	**2436,789**	0,853813	UNCHANGED	0,776319	UNCHANGED	0,909237
10627,39	10874,92	**10616,18**	0,817605	UNCHANGED	0,971181	UNCHANGED	1,187837
5213,554	5784,177	**5438,634**	1,287773	UNCHANGED	1,155649	UNCHANGED	0,897401
2626,29	888,9262	**1394,287**	1,219771	UNCHANGED	0,669789	UNCHANGED	0,549111
6830,308	8782,362	**8038,275**	1,133084	UNCHANGED	1,131067	UNCHANGED	0,998219
31520,14	29216,26	**29446,94**	0,931978	UNCHANGED	1,028237	UNCHANGED	1,103284
3338,206	3850,585	**3773,472**	1,070157	UNCHANGED	0,987415	UNCHANGED	0,922683
1586,393	1443,687	**1549,984**	1,116877	UNCHANGED	1,208035	UNCHANGED	1,081618
114,4516	123,64	**128,9147**	0,54022	UNCHANGED	2,202354	UNCHANGED	4,07677
15242,54	14550,68	**14980,06**	0,94232	UNCHANGED	1,038708	UNCHANGED	1,102288
25745,55	25318,79	**25313,13**	1,219979	UNCHANGED	1,131264	UNCHANGED	0,927282
5968,951	5350,669	**5450,447**	0,796598	UNCHANGED	0,860112	UNCHANGED	1,079732
3793,21	3810,581	**3830,953**	1,079014	UNCHANGED	0,987347	UNCHANGED	0,915046
26368,56	33387,49	**30941,9**	0,945564	UNCHANGED	0,888196	UNCHANGED	0,93933
10768,3	8531,017	**9252,074**	1,158773	UNCHANGED	1,038019	UNCHANGED	0,895792
403,9297	376,0008	**365,8066**	1,071478	UNCHANGED	0,935375	UNCHANGED	0,872977
63595,01	57572,98	**59731,95**	0,895194	UNCHANGED	1,110881	UNCHANGED	1,240939
32340,41	36145,12	**35214,28**	1,059795	UNCHANGED	1,037149	UNCHANGED	0,978632
29308,85	21785,15	**24312,31**	0,95116	UNCHANGED	1,112387	UNCHANGED	1,169505
48857,53	37919	**41579,91**	1,076429	UNCHANGED	0,956347	UNCHANGED	0,888444
1567,911	1422,434	**1464,327**	1,249029	UNCHANGED	1,151193	UNCHANGED	0,921671
7988,65	4875,768	**6158,068**	0,930661	UNCHANGED	0,791828	UNCHANGED	0,850823
17540,45	28609,35	**25579,84**	0,864052	UNCHANGED	1,019098	UNCHANGED	1,17944
1454	1901,606	**2094,798**	0,889082	UNCHANGED	1,118776	UNCHANGED	1,258349
232,2433	364,0339	**296,9458**	0,735827	UNCHANGED	1,013515	UNCHANGED	1,377382
730,9053	1342,794	**1140,445**	1,15235	UNCHANGED	0,867721	UNCHANGED	0,753002

351682,7	444075,4	**407475,5**	0,818419	UNCHANGED	0,87938	UNCHANGED	1,074485
2517,577	2881,079	**2681,875**	1,05972	UNCHANGED	1,221407	UNCHANGED	1,152576
30730,14	50119,46	**42712,48**	0,841831	UNCHANGED	0,813753	UNCHANGED	0,966647
43522,85	56355,1	**50982,84**	0,855449	UNCHANGED	0,793907	UNCHANGED	0,92806
65605,88	58628,29	**61829,69**	1,008589	UNCHANGED	0,87795	UNCHANGED	0,870474
5450,197	5188,749	**5141,93**	0,895176	UNCHANGED	1,073718	UNCHANGED	1,19945
37,63588	26,95214	**49,78111**	0,741981	UNCHANGED	11,4348	UNCHANGED	15,41117
1032,111	1285,868	**1199,908**	0,918109	UNCHANGED	0,798443	UNCHANGED	0,869661
1656,712	7260,534	**5564,283**	0,839921	UNCHANGED	0,606667	UNCHANGED	0,72229
3093,066	3251,945	**3060,059**	1,016628	UNCHANGED	0,839972	UNCHANGED	0,826234
190,9523	168,1332	**157,3386**	1,217926	UNCHANGED	0,940511	UNCHANGED	0,772224
3040,949	2399,112	**2696,769**	0,853942	UNCHANGED	0,969947	UNCHANGED	1,135846
14,42286	84,45455	**38,98863**	1,082557	UNCHANGED	1,561707	UNCHANGED	1,442609
7763,988	5100,407	**5814,027**	0,947464	UNCHANGED	0,796647	UNCHANGED	0,84082
1308,118	1411,928	**1430,584**	0,933862	UNCHANGED	0,803562	UNCHANGED	0,860472
2423,981	879,713	**1341,029**	0,954331	UNCHANGED	2,346755	UNCHANGED	2,459057
47159,92	45304,1	**46479,23**	1,178269	UNCHANGED	1,128785	UNCHANGED	0,958003
12053,71	10884,67	**11398,26**	1,081884	UNCHANGED	1,19254	UNCHANGED	1,102281
81382,99	84011,56	**83675,39**	1,087133	UNCHANGED	1,018361	UNCHANGED	0,93674
1474,175	1537,062	**1473,149**	0,798904	UNCHANGED	0,655422	UNCHANGED	0,820401
7757,451	9626,647	**9080,886**	1,053873	UNCHANGED	1,107886	UNCHANGED	1,051252
8793,282	8880,784	**9417,752**	1,127394	UNCHANGED	1,314049	UNCHANGED	1,165564
2212,641	803,2447	**1279,166**	0,566314	UNCHANGED	0,637454	UNCHANGED	1,125619
12618,78	14035,6	**13683,42**	0,79953	UNCHANGED	0,829207	UNCHANGED	1,037118
22184,33	21588,11	**22200,64**	0,962973	UNCHANGED	0,894963	UNCHANGED	0,929374
31878,69	27356,38	**29276,71**	0,893469	UNCHANGED	0,960304	UNCHANGED	1,074804
1963,91	1397,312	**1630,857**	0,849314	UNCHANGED	0,784689	UNCHANGED	0,923909
11047,96	18469,2	**15605,04**	0,766014	UNCHANGED	0,884573	UNCHANGED	1,154774
1935,59	1635,102	**1672,886**	0,728179	UNCHANGED	0,892203	UNCHANGED	1,225253
2869,423	2957,457	**2990,747**	1,058002	UNCHANGED	1,122295	UNCHANGED	1,060767
1987,309	809,5261	**1188,309**	0,988752	UNCHANGED	1,161832	UNCHANGED	1,175049
447,9438	65,9752	**182,3243**	1,366	UNCHANGED	1,29836	UNCHANGED	0,950483
15748,15	15808,95	**15228,89**	0,954086	UNCHANGED	0,910462	UNCHANGED	0,954276
3495,927	2071,951	**2587,537**	1,126903	UNCHANGED	0,905483	UNCHANGED	0,803514
3070,998	3906,862	**3767,584**	0,979217	UNCHANGED	0,853135	UNCHANGED	0,871242
5170,776	5214,516	**5237,236**	0,982421	UNCHANGED	1,047659	UNCHANGED	1,066405
13377,24	11230,48	**12487,29**	0,944041	UNCHANGED	1,011829	UNCHANGED	1,071806
22403,77	21202,46	**21375,71**	0,909865	UNCHANGED	1,045863	UNCHANGED	1,149471
3599,153	2659,169	**2599,97**	0,79288	UNCHANGED	0,907129	UNCHANGED	1,144094
4765,9	3920,795	**4156,89**	1,090518	UNCHANGED	1,131729	UNCHANGED	1,03779
2525,654	2385,079	**2442,15**	1,123303	UNCHANGED	1,290386	UNCHANGED	1,148742
761,1395	966,1925	**843,7611**	0,834777	UNCHANGED	0,917154	UNCHANGED	1,098681
5204,928	14000,32	**10431,07**	0,602568	UNCHANGED	0,601941	UNCHANGED	0,998959
27206,47	32985,72	**31715,99**	0,95868	UNCHANGED	1,124218	UNCHANGED	1,172672
18845	20014,79	**19912,7**	1,024052	UNCHANGED	0,93735	UNCHANGED	0,915335
27412,82	25084,67	**25971,73**	0,879879	UNCHANGED	0,953846	UNCHANGED	1,084064
481362,4	583290,3	**549033,5**	0,904155	UNCHANGED	1,016085	UNCHANGED	1,123795
23875,77	28341,69	**26726,59**	0,868716	UNCHANGED	0,973991	UNCHANGED	1,121185
623,9781	971,4226	**824,9445**	0,884864	UNCHANGED	1,009653	UNCHANGED	1,141026
1971,946	2769,74	**2322,295**	0,775081	UNCHANGED	1,040305	UNCHANGED	1,342188

2017,169	1277,269	**1496,108**	0,757037	UNCHANGED	1,013543	UNCHANGED	1,33883
3531,957	3199,627	**3248,858**	0,759245	UNCHANGED	0,985626	UNCHANGED	1,298166
3690,616	3787,29	**3791,836**	1,090741	UNCHANGED	1,066518	UNCHANGED	0,977792
3426,016	3381,306	**3317,06**	0,898874	UNCHANGED	1,172721	UNCHANGED	1,304656
10262,35	10693,24	**10092,1**	1,093361	UNCHANGED	1,145254	UNCHANGED	1,047462
20445,84	17448,38	**18216,49**	1,040597	UNCHANGED	1,343301	UNCHANGED	1,290894
10146,12	5104,669	**6719,736**	0,989686	UNCHANGED	0,778973	UNCHANGED	0,787091
79538,16	92788,49	**89032,01**	1,082164	UNCHANGED	1,082188	UNCHANGED	1,000022
60292,53	73100,38	**69337,8**	0,979737	UNCHANGED	0,920921	UNCHANGED	0,939967
26384,05	19344,62	**21852,75**	1,155602	UNCHANGED	0,979677	UNCHANGED	0,847764
167,4273	1138,003	**849,6217**	1,011146	UNCHANGED	0,620209	UNCHANGED	0,613372
131714,8	135401,6	**136077,5**	0,968122	UNCHANGED	1,059038	UNCHANGED	1,09391
11034,61	5959,956	**7671,578**	1,142779	UNCHANGED	0,886328	UNCHANGED	0,77559
10734,63	8642,348	**9160,35**	1,074411	UNCHANGED	1,527761	UNCHANGED	1,421953
986,2056	4234,849	**3035,222**	1,022324	UNCHANGED	0,643993	UNCHANGED	0,629931
24170,69	27137,27	**25585,43**	0,963428	UNCHANGED	1,074535	UNCHANGED	1,115324
54097,65	59964,95	**59976,14**	0,919902	UNCHANGED	1,047952	UNCHANGED	1,1392
34835,36	26170,57	**29623,34**	0,866298	UNCHANGED	1,062303	UNCHANGED	1,226256
4933,919	4831,477	**4827,389**	0,867707	UNCHANGED	0,949164	UNCHANGED	1,093876
2182,672	2127,439	**2146,936**	0,847943	UNCHANGED	1,033047	UNCHANGED	1,218297
5573,889	4643,002	**4903,91**	0,86297	UNCHANGED	0,845514	UNCHANGED	0,979772
14164,35	15446,21	**15222,48**	1,023489	UNCHANGED	0,92984	UNCHANGED	0,9085
997,5875	588,6586	**704,8779**	1,11427	UNCHANGED	1,57652	UNCHANGED	1,414846
1017,637	990,4266	**930,9793**	0,841194	UNCHANGED	0,84764	UNCHANGED	1,007663
4915,96	6648,354	**5667,798**	0,939305	UNCHANGED	0,888045	UNCHANGED	0,945429
35770,29	28015,09	**30462,52**	1,014755	UNCHANGED	1,075065	UNCHANGED	1,059432
494,473	446,1001	**435,3408**	0,962713	UNCHANGED	1,608041	UNCHANGED	1,670322
377,6715	224,6913	**388,4024**	1,267366	UNCHANGED	1,18523	UNCHANGED	0,935192
5912,837	8258,377	**7581,338**	0,910846	UNCHANGED	1,079349	UNCHANGED	1,184997
2922,247	2280,858	**2471,59**	0,816594	UNCHANGED	0,830221	UNCHANGED	1,016688
2119,674	2554,855	**2413,975**	1,098328	UNCHANGED	1,011441	UNCHANGED	0,920891
5298,35	5946,688	**5633,118**	0,969246	UNCHANGED	1,052701	UNCHANGED	1,086103
3814,382	2859,614	**3118,425**	0,894367	UNCHANGED	1,023024	UNCHANGED	1,143852
102,2674	128,9105	**103,4677**	1,131547	UNCHANGED	1,935069	UNCHANGED	1,710109
5992,875	8201,599	**7704,813**	0,85982	UNCHANGED	0,849022	UNCHANGED	0,987442
9478,308	10181,7	**10129,44**	1,008189	UNCHANGED	1,048564	UNCHANGED	1,040047
11432,49	9089,239	**9967,325**	0,94635	UNCHANGED	0,938524	UNCHANGED	0,99173
974578,4	890245,6	**913004,5**	0,879051	UNCHANGED	0,91841	UNCHANGED	1,044775
28952,67	29376,02	**30059,16**	1,030402	UNCHANGED	0,952281	UNCHANGED	0,924184
939,7852	1844,291	**1481,807**	0,843158	UNCHANGED	0,81532	UNCHANGED	0,966984
13106,59	7396,961	**9320,166**	1,168191	UNCHANGED	1,09451	UNCHANGED	0,936927
4441,885	4179,593	**4303,626**	1,027229	UNCHANGED	0,950686	UNCHANGED	0,925486
4506,454	7124,787	**5984,862**	0,958907	UNCHANGED	1,090385	UNCHANGED	1,137113
4900,292	4729,525	**4638,289**	0,95039	UNCHANGED	0,892528	UNCHANGED	0,939118
3025,895	4017,657	**3567,693**	1,075707	UNCHANGED	1,077833	UNCHANGED	1,001976
64542,54	81769,41	**76971,83**	0,928043	UNCHANGED	0,873875	UNCHANGED	0,941632
4368,118	5275,475	**5081,56**	1,148343	UNCHANGED	1,094238	UNCHANGED	0,952884
5081,806	4855,95	**4821,591**	0,932829	UNCHANGED	0,962742	UNCHANGED	1,032067
5549,682	4574,54	**4875,896**	1,167994	UNCHANGED	1,141495	UNCHANGED	0,977312
3305,692	3370,958	**3184,011**	1,095077	UNCHANGED	1,058577	UNCHANGED	0,966668

939,8285	784,8661	**812,8796**	1,218308	UNCHANGED	2,15819	UNCHANGED	1,771465
2649,675	1899,246	**2036,817**	1,127271	UNCHANGED	0,967796	UNCHANGED	0,858529
159775,2	150992,1	**158835,7**	0,925151	UNCHANGED	0,915918	UNCHANGED	0,990019
5013,96	5585,883	**5351,875**	1,103657	UNCHANGED	1,077922	UNCHANGED	0,976682
3284,304	2850,149	**2746,271**	0,894059	UNCHANGED	0,820949	UNCHANGED	0,918227
9583,215	3557,623	**5682,548**	0,792277	UNCHANGED	0,655048	UNCHANGED	0,826791
127243,3	141396,2	**139709,4**	0,836055	UNCHANGED	0,884333	UNCHANGED	1,057746
4586,303	3067,186	**3798,839**	0,828491	UNCHANGED	0,980906	UNCHANGED	1,183967
1117,688	1732,145	**1864,025**	0,849588	UNCHANGED	0,683	UNCHANGED	0,803919
5685,808	5607,452	**5683,529**	0,892655	UNCHANGED	0,912226	UNCHANGED	1,021925
12851,76	11946,32	**11636,71**	1,000014	UNCHANGED	0,873648	UNCHANGED	0,873636
6426,603	7047,631	**7033,173**	1,004869	UNCHANGED	1,084196	UNCHANGED	1,078943
8744,074	10776,19	**9676,359**	0,939861	UNCHANGED	0,949136	UNCHANGED	1,009868
4714,531	5777,597	**5541,776**	0,99583	UNCHANGED	1,146447	UNCHANGED	1,151248
7146,961	5870,673	**6257,165**	0,612777	UNCHANGED	0,615994	UNCHANGED	1,00525
3601,596	2859,056	**3141,886**	0,931511	UNCHANGED	0,975326	UNCHANGED	1,047036
182670,7	144644,1	**158628,9**	1,006934	UNCHANGED	1,180139	UNCHANGED	1,172012
2904,257	2334,713	**2470,495**	1,03509	UNCHANGED	0,94109	UNCHANGED	0,909187
5092,562	5198,246	**4966,49**	0,994239	UNCHANGED	0,897682	UNCHANGED	0,902884
88,74776	228,8446	**127,2639**	0,642574	UNCHANGED	0,989375	UNCHANGED	1,539706
2211,521	2975,398	**2746,349**	1,094258	UNCHANGED	0,948898	UNCHANGED	0,867161
97,95453	69,30982	**75,40535**	0,614887	UNCHANGED	1,033976	UNCHANGED	1,68157
716,6632	760,7604	**782,5828**	1,494877	UNCHANGED	1,141873	UNCHANGED	0,763858
3805,078	3449,63	**3611,829**	1,068949	UNCHANGED	1,031503	UNCHANGED	0,964969
15054	11036,51	**13212,29**	0,901217	UNCHANGED	0,929801	UNCHANGED	1,031718
136,7762	86,29361	**112,8313**	1,425102	UNCHANGED	1,192613	UNCHANGED	0,836862
9763,837	11911,04	**11219,37**	1,023822	UNCHANGED	0,937952	UNCHANGED	0,916128
444,3215	448,674	**464,4015**	0,833551	UNCHANGED	1,131256	UNCHANGED	1,357152
5571,154	4728,997	**5032,092**	0,874258	UNCHANGED	0,958374	UNCHANGED	1,096214
117789,8	67221,44	**108457,2**	0,794303	UNCHANGED	0,794027	UNCHANGED	0,999653
20287,79	20034,97	**20752,03**	0,970231	UNCHANGED	1,05516	UNCHANGED	1,087535
107342,2	100902	**104021,9**	0,918857	UNCHANGED	0,918252	UNCHANGED	0,999341
4553,12	4550,961	**4520,841**	0,97563	UNCHANGED	0,995109	UNCHANGED	1,019965
59693,88	111242,5	**95069,02**	0,858113	UNCHANGED	0,923448	UNCHANGED	1,076138
11473,34	12783,41	**12638,47**	0,85787	UNCHANGED	0,873298	UNCHANGED	1,017984
131760,3	143326,1	**139479,4**	1,026725	UNCHANGED	0,951626	UNCHANGED	0,926856
3453,771	2431,297	**2680,992**	0,748735	UNCHANGED	0,948536	UNCHANGED	1,266852
2217,348	390,8283	**1062,516**	1,455554	UNCHANGED	0,792456	UNCHANGED	0,544436
156400,4	68390,23	**96120,13**	0,98229	UNCHANGED	1,103996	UNCHANGED	1,123901
9566,483	9772,624	**10454,06**	1,02902	UNCHANGED	1,053781	UNCHANGED	1,024063
2002,458	2911,205	**2471,796**	0,849657	UNCHANGED	1,077333	UNCHANGED	1,267963
427,5372	949,2742	**728,4534**	1,425454	UNCHANGED	1,289348	UNCHANGED	0,904518
915,1777	904,8915	**916,5381**	1,192354	UNCHANGED	1,042026	UNCHANGED	0,873923
281,8009	485,3057	**419,3863**	1,003291	UNCHANGED	1,203527	UNCHANGED	1,19958
9995,716	7585,552	**8575,368**	1,074346	UNCHANGED	1,047226	UNCHANGED	0,974756
141,1997	182,9962	**165,8733**	0,848842	UNCHANGED	0,857768	UNCHANGED	1,010516
100549,2	66667,19	**79745,41**	0,966178	UNCHANGED	1,080553	UNCHANGED	1,118378
25775,07	29675,51	**28171,12**	0,944679	UNCHANGED	0,956944	UNCHANGED	1,012983
101281,8	106377	**104207,3**	0,975237	UNCHANGED	1,095019	UNCHANGED	1,122823
47258,67	47891,54	**46781,21**	0,932765	UNCHANGED	0,999015	UNCHANGED	1,071025

2366,092	2887,876	**2782,518**	1,003939	UNCHANGED	0,901602	UNCHANGED	0,898065
311,2155	239,9908	**388,7173**	1,377574	UNCHANGED	1,612345	UNCHANGED	1,170423
16986,08	15130,63	**15612,66**	0,95878	UNCHANGED	0,91101	UNCHANGED	0,950176
5437,436	3742,196	**3977,778**	0,868118	UNCHANGED	0,894642	UNCHANGED	1,030553
16934,01	13866,48	**15406,04**	1,011267	UNCHANGED	1,099299	UNCHANGED	1,087051
2572,786	3791,409	**3583,678**	1,075873	UNCHANGED	0,919887	UNCHANGED	0,855014
7108,498	5003,228	**5786,266**	0,9452	UNCHANGED	0,861814	UNCHANGED	0,91178
4647,564	4439,912	**4544,574**	1,025332	UNCHANGED	0,949322	UNCHANGED	0,925868
55365,33	61649,22	**59370,77**	0,906432	UNCHANGED	1,054378	UNCHANGED	1,163218
11693,11	10062,45	**10636,8**	0,967531	UNCHANGED	1,086759	UNCHANGED	1,123229
4386,318	4131,34	**5031,704**	0,843472	UNCHANGED	0,828377	UNCHANGED	0,982104
301,8235	237,542	**248,3765**	1,272461	UNCHANGED	1,381358	UNCHANGED	1,08558
47077,91	30703,4	**35458,84**	1,050522	UNCHANGED	0,911465	UNCHANGED	0,867631
1368,433	1348,093	**1428,283**	0,962415	UNCHANGED	0,921239	UNCHANGED	0,957215
8931,256	10107,12	**9372,849**	0,953725	UNCHANGED	0,878097	UNCHANGED	0,920702
17,09756	247,2622	**167,5199**	0,907314	UNCHANGED	0,410568	UNCHANGED	0,452509
6637,765	6378,896	**6524,566**	0,995496	UNCHANGED	0,893936	UNCHANGED	0,89798
9913,655	5509,188	**6880,486**	0,812572	UNCHANGED	0,965464	UNCHANGED	1,188157
7909,107	5623,625	**6410,977**	0,829463	UNCHANGED	0,819887	UNCHANGED	0,988455
330,1248	44,18328	**147,7543**	0,729845	UNCHANGED	0,696603	UNCHANGED	0,954454
28587,55	28078,58	**28498,86**	1,017963	UNCHANGED	0,978884	UNCHANGED	0,96161
9533,076	10551,62	**10101,94**	1,063471	UNCHANGED	0,981425	UNCHANGED	0,922851
19695,59	17918,29	**18315,53**	1,053293	UNCHANGED	1,022084	UNCHANGED	0,97037
240092,9	174184,8	**197008,8**	1,062331	UNCHANGED	1,093616	UNCHANGED	1,02945
1399,843	2066,052	**1785,714**	1,215235	UNCHANGED	1,157318	UNCHANGED	0,952341
5684,511	8240,26	**7364,314**	1,007412	UNCHANGED	0,890359	UNCHANGED	0,883809
1238,233	1570,411	**1483,151**	0,802503	UNCHANGED	0,966804	UNCHANGED	1,204736
11575,98	11330,81	**11708,08**	0,926398	UNCHANGED	0,943838	UNCHANGED	1,018826
12473,96	11412,38	**11990,7**	0,931585	UNCHANGED	0,949212	UNCHANGED	1,018922
9489,039	8652,797	**9078,771**	0,886711	UNCHANGED	0,870072	UNCHANGED	0,981234
32007,51	29783,16	**30734,93**	0,916852	UNCHANGED	0,955919	UNCHANGED	1,04261
50698,26	49425,75	**51544,13**	0,880373	UNCHANGED	0,905949	UNCHANGED	1,029051
16847,41	16522,06	**16243,78**	0,976539	UNCHANGED	1,088158	UNCHANGED	1,1143
603976,9	660556,4	**646265,2**	0,970151	UNCHANGED	0,925248	UNCHANGED	0,953716
94164,26	79769,55	**85804**	0,936728	UNCHANGED	0,975035	UNCHANGED	1,040894
19757,96	27058,04	**24331,5**	0,933393	UNCHANGED	0,894173	UNCHANGED	0,957981
3775,452	4482,74	**4280,434**	0,80432	UNCHANGED	0,811149	UNCHANGED	1,008489
1608,043	1753,567	**1704,775**	0,90149	UNCHANGED	0,93591	UNCHANGED	1,038181
8167,511	6107,059	**6760,262**	1,054674	UNCHANGED	0,967681	UNCHANGED	0,917517
2269,333	2502,237	**2400,724**	0,89438	UNCHANGED	0,961952	UNCHANGED	1,075551
31052,23	29154,23	**29744,62**	0,931808	UNCHANGED	1,007449	UNCHANGED	1,081177
593,3614	560,1984	**657,9714**	1,239483	UNCHANGED	1,171147	UNCHANGED	0,944868
111,8339	8,726131	**44,2625**	0,737397	UNCHANGED	0,96775	UNCHANGED	1,312387
14457,17	11862,04	**13064,46**	1,128096	UNCHANGED	1,334342	UNCHANGED	1,182827
3929,724	6410,045	**5498,084**	0,95403	UNCHANGED	1,068005	UNCHANGED	1,119467
29665,13	30881,92	**30230,24**	1,062625	UNCHANGED	1,035481	UNCHANGED	0,974455
245,1389	211,8917	**260,9938**	0,970059	UNCHANGED	0,858349	UNCHANGED	0,884841
7878,855	8576,889	**8150,739**	0,951797	UNCHANGED	1,022869	UNCHANGED	1,074671
4824,277	4292,075	**4451,808**	0,902763	UNCHANGED	0,932793	UNCHANGED	1,033264
1455,938	1469,888	**1044,936**	2,678682	UNCHANGED	5,971769	UNCHANGED	2,229368

3964,642	4516,416	**4258,49**	1,051806	UNCHANGED	0,973986	UNCHANGED	0,926013
42374,69	49043,63	**47343,04**	0,981162	UNCHANGED	1,056332	UNCHANGED	1,076613
10798,4	15292,1	**14324,98**	0,982766	UNCHANGED	1,076427	UNCHANGED	1,095303
6071,618	2936,51	**4120,141**	1,079503	UNCHANGED	0,970511	UNCHANGED	0,899035
19104,24	5403,188	**10326,67**	0,899592	UNCHANGED	1,335082	UNCHANGED	1,484098
676,4987	309,4768	**355,8431**	1,735854	UNCHANGED	2,416865	UNCHANGED	1,39232
7601,532	7576,46	**7620,331**	0,970373	UNCHANGED	1,092026	UNCHANGED	1,125367
396,0767	345,0962	**347,2084**	1,072499	UNCHANGED	0,928683	UNCHANGED	0,865906
1083,361	1382,693	**1268,536**	1,100519	UNCHANGED	1,200137	UNCHANGED	1,090519
1637,634	1739,963	**1652,553**	1,036787	UNCHANGED	0,961971	UNCHANGED	0,927838
2736,86	4166,985	**3522,024**	0,92663	UNCHANGED	1,023605	UNCHANGED	1,104654
1831,998	3305,835	**2643,357**	0,899341	UNCHANGED	0,837594	UNCHANGED	0,931342
27756,6	27924,72	**27032,61**	1,008313	UNCHANGED	1,047177	UNCHANGED	1,038544
108,5247	125,2601	**118,8653**	0,919064	UNCHANGED	1,113599	UNCHANGED	1,211667
33791,6	40536,06	**38283,97**	0,964891	UNCHANGED	0,975485	UNCHANGED	1,01098
2811,175	3273,792	**3110,5**	0,995299	UNCHANGED	0,935134	UNCHANGED	0,939551
2724,688	2421,275	**2517,791**	1,028384	UNCHANGED	0,923722	UNCHANGED	0,898227
346,6589	344,8511	**357,3636**	1,012576	UNCHANGED	0,939676	UNCHANGED	0,928005
6398,595	7814,15	**7245,46**	1,015607	UNCHANGED	1,052343	UNCHANGED	1,036172
1709,695	1786,259	**1805,897**	0,988916	UNCHANGED	1,028645	UNCHANGED	1,040175
65540,37	69037,96	**68536,12**	1,038163	UNCHANGED	1,050682	UNCHANGED	1,012058
40895,87	37081,64	**37673,35**	0,968429	UNCHANGED	0,947447	UNCHANGED	0,978334
9294,332	6819,407	**7560,072**	1,136221	UNCHANGED	1,169243	UNCHANGED	1,029063
10625,3	7493,394	**8309,01**	0,815905	UNCHANGED	0,91278	UNCHANGED	1,118733
1387,26	1426,619	**1405,105**	0,906402	UNCHANGED	0,987244	UNCHANGED	1,089191
1741,302	2469,128	**2261,719**	0,905906	UNCHANGED	0,948755	UNCHANGED	1,047299
3734,427	3447,928	**3741,115**	1,066622	UNCHANGED	1,055485	UNCHANGED	0,989558
3251,449	3388,358	**3318,868**	1,030277	UNCHANGED	1,035118	UNCHANGED	1,004698
3627,006	3038,617	**3753,573**	1,119918	UNCHANGED	1,261492	UNCHANGED	1,126415
39,26924	16,56103	**39,54369**	0,357589	UNCHANGED	0,759201	UNCHANGED	2,123111
1443,235	1880,792	**1678,004**	0,981425	UNCHANGED	1,157466	UNCHANGED	1,179373
138243,7	75646,78	**96984,54**	0,919026	UNCHANGED	1,031296	UNCHANGED	1,122163
18906,29	10104,98	**12796,07**	0,975722	UNCHANGED	0,853925	UNCHANGED	0,875172
1532,734	1380,83	**1551,383**	0,926226	UNCHANGED	1,063043	UNCHANGED	1,147715
1087,908	1220,4	**1069,903**	0,818146	UNCHANGED	0,855517	UNCHANGED	1,045679
15197,78	15682,75	**15550,05**	0,949869	UNCHANGED	1,073552	UNCHANGED	1,130211
1646,655	2348,077	**2149,008**	0,995054	UNCHANGED	1,112416	UNCHANGED	1,117945
4900,716	5835,385	**5864,207**	0,950078	UNCHANGED	0,948744	UNCHANGED	0,998596
30117,32	23330,58	**25797,75**	1,000668	UNCHANGED	1,045764	UNCHANGED	1,045066
40199,48	39249,93	**40221,7**	0,996198	UNCHANGED	0,974327	UNCHANGED	0,978045
67907,71	66604,61	**67198,77**	0,938342	UNCHANGED	0,957297	UNCHANGED	1,0202
1610,844	771,4857	**1008,218**	1,121209	UNCHANGED	0,941692	UNCHANGED	0,83989
32952,41	39418,75	**37428,54**	0,952134	UNCHANGED	0,982391	UNCHANGED	1,031778
26377,49	32381,17	**30179,81**	0,946791	UNCHANGED	1,016166	UNCHANGED	1,073274
8592,663	7123,457	**7566,354**	1,036614	UNCHANGED	0,963036	UNCHANGED	0,92902
17054,45	12686,26	**14171,6**	1,053453	UNCHANGED	0,982076	UNCHANGED	0,932245
11256,98	7066,49	**8873,288**	0,963315	UNCHANGED	1,10038	UNCHANGED	1,142285
46238,94	50244,02	**48123,95**	1,001513	UNCHANGED	1,018713	UNCHANGED	1,017174
16016,19	5016,375	**8746,554**	0,987799	UNCHANGED	1,036584	UNCHANGED	1,049387
5744,464	3410,507	**4221,447**	0,981432	UNCHANGED	0,98391	UNCHANGED	1,002525

7139,188	7669,168	**7132,695**	0,979556	UNCHANGED	1,030157	UNCHANGED	1,051657
59388,51	84238,71	**75096,42**	0,976147	UNCHANGED	0,932318	UNCHANGED	0,9551
73156,48	32401,26	**46049,51**	1,102045	UNCHANGED	0,9652	UNCHANGED	0,875826
14142,41	7380,277	**9593,308**	0,891063	UNCHANGED	0,87109	UNCHANGED	0,977585
48,66067	71,97301	**55,07419**	1,027092	UNCHANGED	0,93662	UNCHANGED	0,911915
61367,04	40099,47	**48902,4**	1,05498	UNCHANGED	1,010811	UNCHANGED	0,958134
9299,092	10041,03	**9807,702**	1,040079	UNCHANGED	1,053302	UNCHANGED	1,012714
36721,46	22063,24	**26898,77**	0,847768	UNCHANGED	0,867765	UNCHANGED	1,023588
7680,792	10243,65	**9354,35**	0,981316	UNCHANGED	0,932651	UNCHANGED	0,950409
8324,295	1847,585	**3996,535**	0,915093	UNCHANGED	0,993658	UNCHANGED	1,085855
191449,8	181730,1	**185394,9**	1,021321	UNCHANGED	0,984942	UNCHANGED	0,964381
521,2243	525,317	**504,4893**	0,998864	UNCHANGED	0,964128	UNCHANGED	0,965225
15477,27	15111,57	**15284,17**	0,975011	UNCHANGED	1,028985	UNCHANGED	1,055358
9545,804	9673,042	**9768,307**	1,038292	UNCHANGED	1,061779	UNCHANGED	1,022621
51959,88	56653,44	**54690,91**	0,956868	UNCHANGED	0,955985	UNCHANGED	0,999077
1709,7	1978,901	**1808,13**	1,030173	UNCHANGED	1,011073	UNCHANGED	0,98146
779,6833	248,0316	**441,5884**	1,010592	UNCHANGED	1,041601	UNCHANGED	1,030685
85,52643	291,9063	**231,4725**	1,48262	UNCHANGED	1,482257	UNCHANGED	0,999755
2693,226	2510,897	**2450,87**	0,968578	UNCHANGED	1,018041	UNCHANGED	1,051068
1432,716	2086,491	**1888,033**	1,010396	UNCHANGED	0,950617	UNCHANGED	0,940836
198780,8	70415,99	**116570,5**	1,033228	UNCHANGED	0,96971	UNCHANGED	0,938524
290069,6	265623,5	**273727,6**	0,859457	UNCHANGED	1,119374	UNCHANGED	1,302421
3180,317	3098,614	**3179,081**	0,918148	UNCHANGED	1,039616	UNCHANGED	1,132297
34682,12	28649,75	**30353,62**	1,016231	UNCHANGED	1,070777	UNCHANGED	1,053674
4731,757	3646,033	**4294,286**	1,008083	UNCHANGED	0,960767	UNCHANGED	0,953064
9818,389	9514,628	**9763,857**	1,036075	UNCHANGED	1,017028	UNCHANGED	0,981616
20361,04	26262	**24907,91**	0,964769	UNCHANGED	0,947215	UNCHANGED	0,981804
8991,628	9594,284	**8802,409**	1,023647	UNCHANGED	1,041446	UNCHANGED	1,017388
63156,94	62337,23	**64670,96**	1,008438	UNCHANGED	0,969004	UNCHANGED	0,960896
77,54002	155,6108	**108,8849**	0,800416	UNCHANGED	1,216265	UNCHANGED	1,519541
4843,981	4676,52	**4728,384**	1,046986	UNCHANGED	1,071295	UNCHANGED	1,023218
48641,9	29010,81	**35644,11**	1,015322	UNCHANGED	0,936122	UNCHANGED	0,921995
1188,24	598,3892	**781,728**	0,897266	UNCHANGED	1,036788	UNCHANGED	1,155496
289,5171	2505,13	**1866,068**	0,721384	UNCHANGED	0,55927	UNCHANGED	0,775273
36095,29	35258,99	**35713,62**	1,038639	UNCHANGED	1,002602	UNCHANGED	0,965304
19779,82	19926,14	**20208,49**	0,97051	UNCHANGED	0,972031	UNCHANGED	1,001567
361968	387488,5	**378668,8**	0,974565	UNCHANGED	0,964186	UNCHANGED	0,98935
31126,98	23378,7	**26022,34**	1,002409	UNCHANGED	1,040562	UNCHANGED	1,038061
2178,065	3445,607	**2935,69**	1,300983	UNCHANGED	1,137254	UNCHANGED	0,87415
48644,95	57795,52	**56736,86**	0,987439	UNCHANGED	0,956698	UNCHANGED	0,968868
23699,97	7821,257	**12920,28**	0,849981	UNCHANGED	0,841931	UNCHANGED	0,99053
25922,29	15800,92	**19601,24**	0,943619	UNCHANGED	0,983235	UNCHANGED	1,041982
41741,39	43136,41	**42835,85**	0,990033	UNCHANGED	0,974397	UNCHANGED	0,984207
7001,192	5025,056	**5596,445**	1,003774	UNCHANGED	1,005247	UNCHANGED	1,001468
145,9269	56,65476	**90,24658**	0,848852	UNCHANGED	0,835689	UNCHANGED	0,984493
10344,54	8301,641	**9292,285**	0,986169	UNCHANGED	0,979741	UNCHANGED	0,993482
21787,42	19355,18	**20455,85**	1,008028	UNCHANGED	0,994307	UNCHANGED	0,986388
6803,063	6265,315	**6594,621**	1,01405	UNCHANGED	0,991497	UNCHANGED	0,977759
1372,07	1412,521	**1482,359**	1,057645	UNCHANGED	1,0672	UNCHANGED	1,009034
180,4695	126,2012	**134,6899**	0,98533	UNCHANGED	0,973282	UNCHANGED	0,987773

2007,839	3588,915	**2643,068**	0,959576	UNCHANGED	0,928207	UNCHANGED		0,967309
1696,746	801,3392	**1096,819**	0,861854	UNCHANGED	0,888081	UNCHANGED		1,030431
2159,992	2282,627	**2255,391**	0,946388	UNCHANGED	1,05294	UNCHANGED		1,112588
5300,08	1243,924	**2616,094**	0,914071	UNCHANGED	0,864251	UNCHANGED		0,945496
1897,161	1999,014	**1982,481**	0,937462	UNCHANGED	0,960234	UNCHANGED		1,024292
6921,161	6494,458	**6194,557**	0,972907	UNCHANGED	1,003819	UNCHANGED		1,031772
46230,3	26829,79	**33686,37**	0,783718	UNCHANGED	0,92242	UNCHANGED		1,176979
366621,9	352635,7	**359609**	0,989394	UNCHANGED	1,01115	UNCHANGED		1,02199
20562,91	23945,94	**23101,83**	0,980695	UNCHANGED	0,994548	UNCHANGED		1,014126
1760,213	1803,889	**1803,194**	1,098555	UNCHANGED	1,063282	UNCHANGED		0,967892
2559,751	3325,585	**3022,943**	0,96246	UNCHANGED	0,990329	UNCHANGED		1,028955
24684,36	23850,07	**24949,87**	0,963691	UNCHANGED	1,014824	UNCHANGED		1,053059
1293,225	1372,484	**1356,533**	1,048622	UNCHANGED	1,056863	UNCHANGED		1,007859
11141,77	16620,88	**15151,7**	1,001405	UNCHANGED	0,972954	UNCHANGED		0,971589
18558,69	16345,18	**17486,02**	0,990348	UNCHANGED	1,000019	UNCHANGED		1,009765
643,7235	1423,693	**1151,92**	0,902356	UNCHANGED	0,996916	UNCHANGED		1,104793
1246,231	628,8909	**818,9072**	1,047136	UNCHANGED	1,082427	UNCHANGED		1,033703
7242,315	7377,112	**7430,321**	0,997976	UNCHANGED	0,994358	UNCHANGED		0,996374
2066,858	2327,647	**2267,321**	0,990489	UNCHANGED	1,010297	UNCHANGED		1,019998
189,3792	174,0725	**180,8262**	1,617503	UNCHANGED	1,559357	UNCHANGED		0,964052
362,69	678,0808	**610,5954**	0,957546	UNCHANGED	0,967961	UNCHANGED		1,010876
28537,12	42001,62	**36721,18**	1,006797	UNCHANGED	0,984644	UNCHANGED		0,977997
1804,513	1935,198	**1970,425**	0,992723	UNCHANGED	1,007285	UNCHANGED		1,014668
24721,4	28587,43	**27551,25**	1,002631	UNCHANGED	0,998564	UNCHANGED		0,995944
6730,227	3054,933	**4210,051**	0,846889	UNCHANGED	0,956409	UNCHANGED		1,129319
12330,54	15421,05	**14416,29**	0,956769	UNCHANGED	0,992086	UNCHANGED		1,036912

TAG_DMSO/1mg
UP
UP
DOWN
UP
UNCHANGED
UP
-
UP
UP
UP
UP
UP
UP
UP
DOWN
UP
UP
UP
UNCHANGED
UP
UP
UP
UP
UNCHANGED
UP
UNCHANGED
UP
UNCHANGED
UP
UNCHANGED
DOWN
UNCHANGED
UP
UNCHANGED
UP
UP
UP
DOWN

UP
UNCHANGED
UP
UP
UP
UNCHANGED
UP
UP
UP
UNCHANGED
UP
UNCHANGED
UNCHANGED
UP
DOWN
UNCHANGED
UP
UP
UNCHANGED
UP
-
UNCHANGED
UNCHANGED
DOWN
UP
UNCHANGED
DOWN
UP
UP
UNCHANGED
UP
UNCHANGED
UP
UP
DOWN
UNCHANGED
UP
UNCHANGED
DOWN
DOWN
UNCHANGED
UP
UNCHANGED
UNCHANGED
UNCHANGED
UP
UNCHANGED
DOWN
UP
UNCHANGED

UNCHANGED
UNCHANGED
UNCHANGED
UP
UNCHANGED
UP
UNCHANGED
UP
UP
UNCHANGED
UP
UP
UNCHANGED
UP
UP
UNCHANGED
UNCHANGED
UP
DOWN
UP
UP
UNCHANGED
DOWN
UNCHANGED
UNCHANGED
UP
UNCHANGED
UNCHANGED
UNCHANGED
UNCHANGED
DOWN
UNCHANGED
UP
UP
UNCHANGED
UNCHANGED
UP
UP
UP
UNCHANGED
UNCHANGED
DOWN
UP
UNCHANGED
UP
UNCHANGED
DOWN
UP
UP
UNCHANGED

UP
DOWN
UNCHANGED
UNCHANGED
UNCHANGED
UP
UP
UNCHANGED
UNCHANGED
UP
UNCHANGED
UNCHANGED
UP
UP
DOWN
UP
DOWN
UP
UNCHANGED
UNCHANGED
UNCHANGED
UP
UNCHANGED
UNCHANGED
UNCHANGED
UP
DOWN
UP
UP
UNCHANGED
UNCHANGED
UP
UNCHANGED
UP
UP
UNCHANGED
UP
UP
UP
UP
UP
UNCHANGED
DOWN
UP
DOWN
UNCHANGED
UNCHANGED
UNCHANGED
UNCHANGED
UNCHANGED

UP
UNCHANGED
UNCHANGED
UP
UNCHANGED
UNCHANGED
UP
UNCHANGED
UNCHANGED
UNCHANGED
UP
UP
UNCHANGED
UP
UNCHANGED
UP
UNCHANGED
UNCHANGED
UP
UP
UNCHANGED
UNCHANGED
UNCHANGED
UP
UP
UNCHANGED
DOWN
UNCHANGED
UP
DOWN
UP
UP
DOWN
UP
DOWN
DOWN
UNCHANGED
DOWN
UNCHANGED
UP
UNCHANGED
UNCHANGED
UP
UNCHANGED
UNCHANGED
UP
UP
DOWN
UNCHANGED
UNCHANGED

UNCHANGED
UP
UP
DOWN
UNCHANGED
UNCHANGED
UP
UP
UP
UNCHANGED
UNCHANGED
UNCHANGED
UNCHANGED
UNCHANGED
UP
UP
UNCHANGED
UNCHANGED
DOWN
UP
UNCHANGED
DOWN
UNCHANGED
UP
UNCHANGED
UP
UNCHANGED
UNCHANGED
UNCHANGED
UNCHANGED
UNCHANGED
UP
UP
UNCHANGED
UNCHANGED
UP
UP
UNCHANGED
UNCHANGED
UNCHANGED
UP
DOWN
UP
UNCHANGED
UP
UNCHANGED
UNCHANGED
UP
DOWN
UNCHANGED

UP
UP
UP
UP
UNCHANGED
UNCHANGED
UP
UNCHANGED
UNCHANGED
UNCHANGED
DOWN
UNCHANGED
UP
UNCHANGED
UP
UNCHANGED
UNCHANGED
UP
DOWN
UNCHANGED
UNCHANGED
UNCHANGED
UNCHANGED
DOWN
UNCHANGED
UNCHANGED
UP
UNCHANGED
DOWN
UP
UNCHANGED
UNCHANGED
UP
UNCHANGED
UP
UNCHANGED
DOWN
UNCHANGED
UNCHANGED
UNCHANGED
UNCHANGED
UP
DOWN
UP
UNCHANGED
DOWN
DOWN
UNCHANGED
UNCHANGED
UNCHANGED

UNCHANGED
UNCHANGED
UNCHANGED
UP
UNCHANGED
UNCHANGED
UNCHANGED
UP
UNCHANGED
UNCHANGED
UNCHANGED
UNCHANGED
UNCHANGED
UNCHANGED
UNCHANGED
UNCHANGED
UNCHANGED
UNCHANGED
UNCHANGED
UNCHANGED
UNCHANGED
UNCHANGED
UNCHANGED
UNCHANGED
UNCHANGED
UNCHANGED
UNCHANGED
UNCHANGED
UNCHANGED
UNCHANGED
UNCHANGED
UNCHANGED
UNCHANGED
UNCHANGED
UNCHANGED
UNCHANGED
UNCHANGED
UNCHANGED
UNCHANGED
UNCHANGED
UNCHANGED
UNCHANGED
UNCHANGED
UNCHANGED
UNCHANGED
UNCHANGED
UNCHANGED
UNCHANGED
UNCHANGED
UNCHANGED

UNCHANGED
UNCHANGED
UNCHANGED
UNCHANGED
UNCHANGED
UNCHANGED
UNCHANGED
UNCHANGED
UNCHANGED
UNCHANGED
UNCHANGED
UNCHANGED
UNCHANGED
UNCHANGED
UNCHANGED
UNCHANGED
UNCHANGED
UNCHANGED
UNCHANGED
UNCHANGED
UNCHANGED
UNCHANGED
UNCHANGED
UNCHANGED
UNCHANGED
UNCHANGED
UNCHANGED
UNCHANGED
UNCHANGED
UNCHANGED
UNCHANGED
UNCHANGED
UNCHANGED
UNCHANGED
UNCHANGED
UNCHANGED
UNCHANGED
UNCHANGED
UNCHANGED
UNCHANGED
UNCHANGED
UNCHANGED
UNCHANGED
UNCHANGED
UNCHANGED
UNCHANGED
UNCHANGED
UNCHANGED
UNCHANGED
UNCHANGED

UNCHANGED
UNCHANGED
UNCHANGED
UNCHANGED
UNCHANGED
UNCHANGED
UNCHANGED
UNCHANGED
UNCHANGED
UNCHANGED
UNCHANGED
UNCHANGED
UNCHANGED
UNCHANGED
UNCHANGED
UNCHANGED
UNCHANGED
UNCHANGED
UNCHANGED
UNCHANGED
UNCHANGED
UNCHANGED
UNCHANGED
UNCHANGED
UNCHANGED
UNCHANGED
UNCHANGED
UNCHANGED
UNCHANGED
UNCHANGED
UNCHANGED
UNCHANGED
UNCHANGED
UNCHANGED
UNCHANGED
UNCHANGED
UNCHANGED
UNCHANGED
UNCHANGED
UNCHANGED
UNCHANGED
UNCHANGED
UNCHANGED
UNCHANGED
UNCHANGED
UNCHANGED
UNCHANGED
UNCHANGED
UNCHANGED
UNCHANGED

UNCHANGED
UNCHANGED
UNCHANGED
UNCHANGED
UNCHANGED
UNCHANGED
UNCHANGED
UNCHANGED
UNCHANGED
UNCHANGED
UNCHANGED
UNCHANGED
UNCHANGED
UNCHANGED
UNCHANGED
UNCHANGED
UNCHANGED
UNCHANGED
UNCHANGED
UNCHANGED
UNCHANGED
UNCHANGED
UNCHANGED
UNCHANGED
UNCHANGED
UNCHANGED
UNCHANGED
UNCHANGED
UNCHANGED
UNCHANGED
UNCHANGED
UNCHANGED
UNCHANGED
UNCHANGED
UNCHANGED
UNCHANGED
UNCHANGED
UNCHANGED
UNCHANGED
UNCHANGED
UNCHANGED
UNCHANGED
UNCHANGED
UNCHANGED
UNCHANGED
UNCHANGED
UNCHANGED
UNCHANGED
UNCHANGED
UNCHANGED

UNCHANGED
UNCHANGED
UNCHANGED
UNCHANGED
UNCHANGED
UNCHANGED
UNCHANGED
UNCHANGED
UNCHANGED
UNCHANGED
UNCHANGED
UNCHANGED
UNCHANGED
UNCHANGED
UNCHANGED
UNCHANGED
UNCHANGED
UNCHANGED
UNCHANGED
UNCHANGED
UNCHANGED
UNCHANGED
UNCHANGED
UNCHANGED
UNCHANGED
UNCHANGED
UNCHANGED
UNCHANGED
UNCHANGED
UNCHANGED
UNCHANGED
UNCHANGED
UNCHANGED
UNCHANGED
UNCHANGED
UNCHANGED
UNCHANGED
UNCHANGED
UNCHANGED
UNCHANGED
UNCHANGED
UNCHANGED
UNCHANGED
UNCHANGED
UNCHANGED
UNCHANGED
UNCHANGED
UNCHANGED
UNCHANGED
UNCHANGED

UNCHANGED
UNCHANGED
UNCHANGED
UNCHANGED
UNCHANGED
UNCHANGED
UNCHANGED
UNCHANGED
UNCHANGED
UNCHANGED
UNCHANGED
UNCHANGED
UNCHANGED
UNCHANGED
UNCHANGED
UNCHANGED
UNCHANGED
UNCHANGED
UNCHANGED
UNCHANGED
UNCHANGED
UNCHANGED
UNCHANGED
UNCHANGED
UNCHANGED
UNCHANGED
UNCHANGED
UNCHANGED
UNCHANGED
UNCHANGED
UNCHANGED
UNCHANGED
UNCHANGED
UNCHANGED
UNCHANGED
UNCHANGED
UNCHANGED
UNCHANGED
UNCHANGED
UNCHANGED
UNCHANGED
UNCHANGED
UNCHANGED
UNCHANGED
UNCHANGED
UNCHANGED
UNCHANGED
UNCHANGED
UNCHANGED
UNCHANGED

UNCHANGED
UNCHANGED
UNCHANGED
UNCHANGED
UNCHANGED
UNCHANGED
UNCHANGED
UNCHANGED
UNCHANGED
UNCHANGED
UNCHANGED
UNCHANGED
UNCHANGED
UNCHANGED
UNCHANGED
UNCHANGED
UNCHANGED
UNCHANGED
UNCHANGED
UNCHANGED
UNCHANGED
UNCHANGED
UNCHANGED
UNCHANGED
UNCHANGED
UNCHANGED
UNCHANGED
UNCHANGED
UNCHANGED
UNCHANGED
UNCHANGED
UNCHANGED
UNCHANGED
UNCHANGED
UNCHANGED
UNCHANGED
UNCHANGED
UNCHANGED
UNCHANGED
UNCHANGED
UNCHANGED
UNCHANGED
UNCHANGED
UNCHANGED
UNCHANGED
UNCHANGED
UNCHANGED
UNCHANGED
UNCHANGED
UNCHANGED

UNCHANGED
UNCHANGED
UNCHANGED
UNCHANGED
UNCHANGED
UNCHANGED
UNCHANGED
UNCHANGED
UNCHANGED
UNCHANGED
UNCHANGED
UNCHANGED
UNCHANGED
UNCHANGED
UNCHANGED
UNCHANGED
UNCHANGED
UNCHANGED
UNCHANGED
UNCHANGED
UNCHANGED
UNCHANGED
UNCHANGED
UNCHANGED
UNCHANGED
UNCHANGED
UNCHANGED
UNCHANGED
UNCHANGED
UNCHANGED
UNCHANGED
UNCHANGED
UNCHANGED
UNCHANGED
UNCHANGED
UNCHANGED
UNCHANGED
UNCHANGED
UNCHANGED
UNCHANGED
UNCHANGED
UNCHANGED
UNCHANGED
UNCHANGED
UNCHANGED
UNCHANGED
UNCHANGED
UNCHANGED
UNCHANGED
UNCHANGED

UNCHANGED
UNCHANGED
UNCHANGED
UNCHANGED
UNCHANGED
UNCHANGED
UNCHANGED
UNCHANGED
UNCHANGED
UNCHANGED
UNCHANGED
UNCHANGED
UNCHANGED
UNCHANGED
UNCHANGED
UNCHANGED
UNCHANGED
UNCHANGED
UNCHANGED
UNCHANGED
UNCHANGED
UNCHANGED
UNCHANGED
UNCHANGED
UNCHANGED
UNCHANGED
UNCHANGED
UNCHANGED
UNCHANGED
UNCHANGED
UNCHANGED
UNCHANGED
UNCHANGED
UNCHANGED
UNCHANGED
UNCHANGED
UNCHANGED
UNCHANGED
UNCHANGED
UNCHANGED
UNCHANGED
UNCHANGED
UNCHANGED
UNCHANGED
UNCHANGED
UNCHANGED
UNCHANGED
UNCHANGED
UNCHANGED
UNCHANGED

UNCHANGED
UNCHANGED
UNCHANGED
UNCHANGED
UNCHANGED
UNCHANGED
UNCHANGED
UNCHANGED
UNCHANGED
UNCHANGED
UNCHANGED
UNCHANGED
UNCHANGED
UNCHANGED
UNCHANGED
UNCHANGED
UNCHANGED
UNCHANGED
UNCHANGED
UNCHANGED
UNCHANGED
UNCHANGED
UNCHANGED
UNCHANGED
UNCHANGED
UNCHANGED
UNCHANGED
UNCHANGED
UNCHANGED
UNCHANGED
UNCHANGED
UNCHANGED
UNCHANGED
UNCHANGED
UNCHANGED
UNCHANGED
UNCHANGED
UNCHANGED
UNCHANGED
UNCHANGED
UNCHANGED
UNCHANGED
UNCHANGED
UNCHANGED
UNCHANGED
UNCHANGED
UNCHANGED
UNCHANGED
UNCHANGED
UNCHANGED

UNCHANGED
UNCHANGED
UNCHANGED
UNCHANGED
UNCHANGED
UNCHANGED
UNCHANGED
UNCHANGED
UNCHANGED
UNCHANGED
UNCHANGED
UNCHANGED
UNCHANGED
UNCHANGED
UNCHANGED
UNCHANGED
UNCHANGED
UNCHANGED
UNCHANGED
UNCHANGED
UNCHANGED
UNCHANGED
UNCHANGED
UNCHANGED
UNCHANGED
UNCHANGED
UNCHANGED
UNCHANGED
UNCHANGED
UNCHANGED
UNCHANGED
UNCHANGED
UNCHANGED
UNCHANGED
UNCHANGED
UNCHANGED
UNCHANGED
UNCHANGED
UNCHANGED
UNCHANGED
UNCHANGED
UNCHANGED
UNCHANGED
UNCHANGED
UNCHANGED
UNCHANGED
UNCHANGED
UNCHANGED
UNCHANGED
UNCHANGED

UNCHANGED
UNCHANGED
UNCHANGED
UNCHANGED
UNCHANGED
UNCHANGED
UNCHANGED
UNCHANGED
UNCHANGED
UNCHANGED
UNCHANGED
UNCHANGED
UNCHANGED
UNCHANGED
UNCHANGED
UNCHANGED
UNCHANGED
UNCHANGED
UNCHANGED
UNCHANGED
UNCHANGED
UNCHANGED
UNCHANGED
UNCHANGED
UNCHANGED
UNCHANGED
UNCHANGED
UNCHANGED
UNCHANGED
UNCHANGED
UNCHANGED
UNCHANGED
UNCHANGED
UNCHANGED
UNCHANGED
UNCHANGED
UNCHANGED
UNCHANGED
UNCHANGED
UNCHANGED
UNCHANGED
UNCHANGED
UNCHANGED
UNCHANGED
UNCHANGED
UNCHANGED
UNCHANGED
UNCHANGED
UNCHANGED
UNCHANGED

UNCHANGED
UNCHANGED
UNCHANGED
UNCHANGED
UNCHANGED
UNCHANGED
UNCHANGED
UNCHANGED
UNCHANGED
UNCHANGED
UNCHANGED
UNCHANGED
UNCHANGED
UNCHANGED
UNCHANGED
UNCHANGED
UNCHANGED
UNCHANGED
UNCHANGED
UNCHANGED
UNCHANGED
UNCHANGED
UNCHANGED
UNCHANGED
UNCHANGED
UNCHANGED
UNCHANGED
UNCHANGED
UNCHANGED
UNCHANGED
UNCHANGED
UNCHANGED
UNCHANGED
UNCHANGED
UNCHANGED
UNCHANGED
UNCHANGED
UNCHANGED
UNCHANGED
UNCHANGED
UNCHANGED
UNCHANGED
UNCHANGED
UNCHANGED
UNCHANGED
UNCHANGED
UNCHANGED
UNCHANGED
UNCHANGED
UNCHANGED

UNCHANGED
UNCHANGED
UNCHANGED
UNCHANGED
UNCHANGED
UNCHANGED
UNCHANGED
UNCHANGED
UNCHANGED
UNCHANGED
UNCHANGED
UNCHANGED
UNCHANGED
UNCHANGED
UNCHANGED
UNCHANGED
UNCHANGED
UNCHANGED
UNCHANGED
UNCHANGED
UNCHANGED
UNCHANGED
UNCHANGED
UNCHANGED
UNCHANGED
UNCHANGED
UNCHANGED
UNCHANGED
UNCHANGED
UNCHANGED
UNCHANGED
UNCHANGED
UNCHANGED
UNCHANGED
UNCHANGED
UNCHANGED
UNCHANGED
UNCHANGED
UNCHANGED
UNCHANGED
UNCHANGED
UNCHANGED
UNCHANGED
UNCHANGED
UNCHANGED
UNCHANGED
UNCHANGED
UNCHANGED
UNCHANGED
UNCHANGED

UNCHANGED
UNCHANGED
UNCHANGED
UNCHANGED
UNCHANGED
UNCHANGED
UNCHANGED
UNCHANGED
UNCHANGED
UNCHANGED
UNCHANGED
UNCHANGED
UNCHANGED
UNCHANGED
UNCHANGED
UNCHANGED
UNCHANGED
UNCHANGED
UNCHANGED
UNCHANGED
UNCHANGED
UNCHANGED
UNCHANGED
UNCHANGED
UNCHANGED
UNCHANGED
UNCHANGED
UNCHANGED
UNCHANGED
UNCHANGED
UNCHANGED
UNCHANGED
UNCHANGED
UNCHANGED
UNCHANGED
UNCHANGED
UNCHANGED
UNCHANGED
UNCHANGED
UNCHANGED
UNCHANGED
UNCHANGED
UNCHANGED
UNCHANGED
UNCHANGED
UNCHANGED
UNCHANGED
UNCHANGED
UNCHANGED
UNCHANGED

UNCHANGED
UNCHANGED
UNCHANGED
UNCHANGED
UNCHANGED
UNCHANGED
UNCHANGED
UNCHANGED
UNCHANGED
UNCHANGED
UNCHANGED
UNCHANGED
UNCHANGED
UNCHANGED
UNCHANGED
UNCHANGED
UNCHANGED
UNCHANGED
UNCHANGED
UNCHANGED
UNCHANGED
UNCHANGED
UNCHANGED
UNCHANGED
UNCHANGED
UNCHANGED
UNCHANGED
UNCHANGED
UNCHANGED
UNCHANGED
UNCHANGED
UNCHANGED
UNCHANGED
UNCHANGED
UNCHANGED
UNCHANGED
UNCHANGED
UNCHANGED
UNCHANGED
UNCHANGED
UNCHANGED
UNCHANGED
UNCHANGED
UNCHANGED
UNCHANGED
UNCHANGED
UNCHANGED
UNCHANGED
UNCHANGED
UNCHANGED

UNCHANGED
UNCHANGED
UNCHANGED
UNCHANGED
UNCHANGED
UNCHANGED
UNCHANGED
UNCHANGED
UNCHANGED
UNCHANGED
UNCHANGED
UNCHANGED
UNCHANGED
UNCHANGED
UNCHANGED
UNCHANGED
UNCHANGED
UNCHANGED
UNCHANGED
UNCHANGED
UNCHANGED
UNCHANGED
UNCHANGED
UNCHANGED
UNCHANGED
UNCHANGED
UNCHANGED
UNCHANGED
UNCHANGED
UNCHANGED
UNCHANGED
UNCHANGED
UNCHANGED
UNCHANGED
UNCHANGED
UNCHANGED
UNCHANGED
UNCHANGED
UNCHANGED
UNCHANGED
UNCHANGED
UNCHANGED
UNCHANGED
UNCHANGED
UNCHANGED
UNCHANGED
UNCHANGED
UNCHANGED
UNCHANGED
UNCHANGED

UNCHANGED
UNCHANGED
UNCHANGED
UNCHANGED
UNCHANGED
UNCHANGED
UNCHANGED
UNCHANGED
UNCHANGED
UNCHANGED
UNCHANGED
UNCHANGED
UNCHANGED
UNCHANGED
UNCHANGED
UNCHANGED
UNCHANGED
UNCHANGED
UNCHANGED
UNCHANGED
UNCHANGED
UNCHANGED
UNCHANGED
UNCHANGED
UNCHANGED
UNCHANGED
UNCHANGED
UNCHANGED
UNCHANGED
UNCHANGED
UNCHANGED
UNCHANGED
UNCHANGED
UNCHANGED
UNCHANGED
UNCHANGED
UNCHANGED
UNCHANGED
UNCHANGED
UNCHANGED
UNCHANGED
UNCHANGED
UNCHANGED
UNCHANGED
UNCHANGED
UNCHANGED
UNCHANGED
UNCHANGED
UNCHANGED
UNCHANGED

UNCHANGED
UNCHANGED
UNCHANGED
UNCHANGED
UNCHANGED
UNCHANGED
UNCHANGED
UNCHANGED
UNCHANGED
UNCHANGED
UNCHANGED
UNCHANGED
UNCHANGED
UNCHANGED
UNCHANGED
UNCHANGED
UNCHANGED
UNCHANGED
UNCHANGED
UNCHANGED
UNCHANGED
UNCHANGED
UNCHANGED
UNCHANGED
UNCHANGED
UNCHANGED
UNCHANGED
UNCHANGED
UNCHANGED
UNCHANGED
UNCHANGED
UNCHANGED
UNCHANGED
UNCHANGED
UNCHANGED
UNCHANGED
UNCHANGED
UNCHANGED
UNCHANGED
UNCHANGED
UNCHANGED
UNCHANGED
UNCHANGED
UNCHANGED
UNCHANGED
UNCHANGED
UNCHANGED
UNCHANGED
UNCHANGED
UNCHANGED

UNCHANGED
UNCHANGED
UNCHANGED
UNCHANGED
UNCHANGED
UNCHANGED
UNCHANGED
UNCHANGED
UNCHANGED
UNCHANGED
UNCHANGED
UNCHANGED
UNCHANGED
UNCHANGED
UNCHANGED
UNCHANGED
UNCHANGED
UNCHANGED
UNCHANGED
UNCHANGED
UNCHANGED
UNCHANGED
UNCHANGED
UNCHANGED
UNCHANGED
UNCHANGED
UNCHANGED
UNCHANGED
UNCHANGED
UNCHANGED
UNCHANGED
UNCHANGED
UNCHANGED
UNCHANGED
UNCHANGED
UNCHANGED
UNCHANGED
UNCHANGED
UNCHANGED
UNCHANGED
UNCHANGED
UNCHANGED
UNCHANGED
UNCHANGED
UNCHANGED
UNCHANGED
UNCHANGED
UNCHANGED
UNCHANGED
UNCHANGED

UNCHANGED
UNCHANGED
UNCHANGED
UNCHANGED
UNCHANGED
UNCHANGED
UNCHANGED
UNCHANGED
UNCHANGED
UNCHANGED
UNCHANGED
UNCHANGED
UNCHANGED
UNCHANGED
UNCHANGED
UNCHANGED
UNCHANGED
UNCHANGED
UNCHANGED
UNCHANGED
UNCHANGED
UNCHANGED
UNCHANGED
UNCHANGED
UNCHANGED
UNCHANGED
UNCHANGED
UNCHANGED
UNCHANGED
UNCHANGED
UNCHANGED
UNCHANGED
UNCHANGED
UNCHANGED
UNCHANGED
UNCHANGED
UNCHANGED
UNCHANGED
UNCHANGED
UNCHANGED
UNCHANGED
UNCHANGED
UNCHANGED
UNCHANGED
UNCHANGED
UNCHANGED
UNCHANGED
UNCHANGED
UNCHANGED
UNCHANGED

UNCHANGED
UNCHANGED
UNCHANGED
UNCHANGED
UNCHANGED
UNCHANGED
UNCHANGED
UNCHANGED
UNCHANGED
UNCHANGED
UNCHANGED
UNCHANGED
UNCHANGED
UNCHANGED
UNCHANGED
UNCHANGED
UNCHANGED
UNCHANGED
UNCHANGED
UNCHANGED
UNCHANGED
UNCHANGED
UNCHANGED
UNCHANGED
UNCHANGED
UNCHANGED
UNCHANGED
UNCHANGED
UNCHANGED
UNCHANGED
UNCHANGED
UNCHANGED
UNCHANGED
UNCHANGED
UNCHANGED
UNCHANGED
UNCHANGED
UNCHANGED
UNCHANGED
UNCHANGED
UNCHANGED
UNCHANGED
UNCHANGED
UNCHANGED
UNCHANGED
UNCHANGED
UNCHANGED
UNCHANGED
UNCHANGED
UNCHANGED

UNCHANGED
UNCHANGED
UNCHANGED
UNCHANGED
UNCHANGED
UNCHANGED
UNCHANGED
UNCHANGED
UNCHANGED
UNCHANGED
UNCHANGED
UNCHANGED
UNCHANGED
UNCHANGED
UNCHANGED
UNCHANGED
UNCHANGED
UNCHANGED
UNCHANGED
UNCHANGED
UNCHANGED
UNCHANGED
UNCHANGED
UNCHANGED
UNCHANGED
UNCHANGED
UNCHANGED
UNCHANGED
UNCHANGED
UNCHANGED
UNCHANGED
UNCHANGED
UNCHANGED
UNCHANGED
UNCHANGED
UNCHANGED
UNCHANGED
UNCHANGED
UNCHANGED
UNCHANGED
UNCHANGED
UNCHANGED
UNCHANGED
UNCHANGED
UNCHANGED
UNCHANGED
UNCHANGED
UNCHANGED
UNCHANGED
UNCHANGED

UNCHANGED
UNCHANGED
UNCHANGED
UNCHANGED
UNCHANGED
UNCHANGED
UNCHANGED
UNCHANGED
UNCHANGED
UNCHANGED
UNCHANGED
UNCHANGED
UNCHANGED
UNCHANGED
UNCHANGED
UNCHANGED
UNCHANGED
UNCHANGED
UNCHANGED
UNCHANGED
UNCHANGED
UNCHANGED
UNCHANGED
UNCHANGED
UNCHANGED
UNCHANGED
UNCHANGED
UNCHANGED
UNCHANGED
UNCHANGED
UNCHANGED
UNCHANGED
UNCHANGED
UNCHANGED
UNCHANGED
UNCHANGED
UNCHANGED
UNCHANGED
UNCHANGED
UNCHANGED
UNCHANGED
UNCHANGED
UNCHANGED
UNCHANGED
UNCHANGED
UNCHANGED
UNCHANGED
UNCHANGED
UNCHANGED
UNCHANGED

UNCHANGED
UNCHANGED
UNCHANGED
UNCHANGED
UNCHANGED
UNCHANGED
UNCHANGED
UNCHANGED
UNCHANGED
UNCHANGED
UNCHANGED
UNCHANGED
UNCHANGED
UNCHANGED
UNCHANGED
UNCHANGED
UNCHANGED
UNCHANGED
UNCHANGED
UNCHANGED
UNCHANGED
UNCHANGED
UNCHANGED
UNCHANGED
UNCHANGED
UNCHANGED
UNCHANGED
UNCHANGED
UNCHANGED
UNCHANGED
UNCHANGED
UNCHANGED
UNCHANGED
UNCHANGED
UNCHANGED
UNCHANGED
UNCHANGED
UNCHANGED
UNCHANGED
UNCHANGED
UNCHANGED
UNCHANGED
UNCHANGED
UNCHANGED
UNCHANGED
UNCHANGED
UNCHANGED
UNCHANGED
UNCHANGED
UNCHANGED

UNCHANGED
UNCHANGED
UNCHANGED
UNCHANGED
UNCHANGED
UNCHANGED
UNCHANGED
UNCHANGED
UNCHANGED
UNCHANGED
UNCHANGED
UNCHANGED
UNCHANGED
UNCHANGED
UNCHANGED
UNCHANGED
UNCHANGED
UNCHANGED
UNCHANGED
UNCHANGED
UNCHANGED
UNCHANGED
UNCHANGED
UNCHANGED
UNCHANGED
UNCHANGED
UNCHANGED
UNCHANGED
UNCHANGED
UNCHANGED
UNCHANGED
UNCHANGED
UNCHANGED
UNCHANGED
UNCHANGED
UNCHANGED
UNCHANGED
UNCHANGED
UNCHANGED
UNCHANGED
UNCHANGED
UNCHANGED
UNCHANGED
UNCHANGED
UNCHANGED
UNCHANGED
UNCHANGED
UNCHANGED
UNCHANGED
UNCHANGED

UNCHANGED
UNCHANGED
UNCHANGED
UNCHANGED
UNCHANGED
UNCHANGED
UNCHANGED
UNCHANGED
UNCHANGED
UNCHANGED
UNCHANGED
UNCHANGED
UNCHANGED
UNCHANGED
UNCHANGED
UNCHANGED
UNCHANGED
UNCHANGED
UNCHANGED
UNCHANGED
UNCHANGED
UNCHANGED
UNCHANGED
UNCHANGED
UNCHANGED
UNCHANGED
